# Asymmetric Total Synthesis of (−)-Phaeocaulisin A

**DOI:** 10.1021/jacs.2c02188

**Published:** 2022-04-13

**Authors:** Áron Péter, Giacomo E. M. Crisenza, David J. Procter

**Affiliations:** Department of Chemistry, The University of Manchester, Oxford Road, Manchester M13 9PL, UK

## Abstract

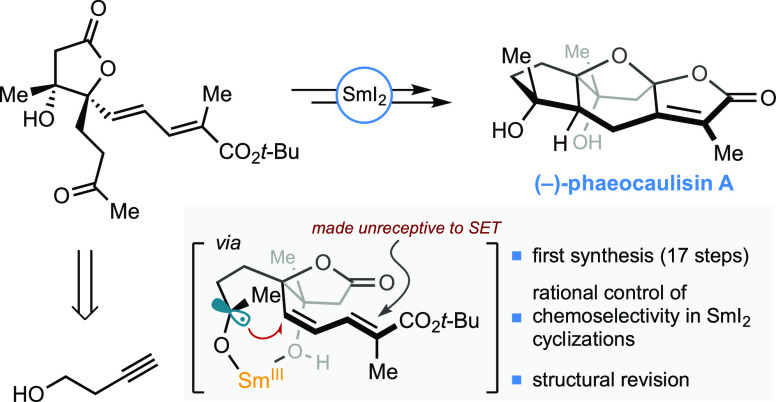

The therapeutic properties of *Curcuma* (ginger and turmeric’s family) have long been known in traditional medicine. However, only recently have guaiane-type sesquiterpenes extracted from *Curcuma phaeocaulis* been submitted to biological testing, and their enhanced bioactivity was highlighted. Among these compounds, phaeocaulisin A has shown remarkable anti-inflammatory and anticancer activity, which appears to be tied to the unique bridged acetal moiety embedded in its tetracyclic framework. Prompted by the promising biological profile of phaeocaulisin A and by the absence of a synthetic route for its provision, we have implemented the first enantioselective total synthesis of phaeocaulisin A in 17 steps with 2% overall yield. Our route design builds on the identification of an enantioenriched lactone intermediate, tailored with both a ketone moiety and a conjugated alkene system. Taking advantage of the umpolung carbonyl-olefin coupling reactivity enabled by the archetypal single-electron transfer (SET) reductant samarium diiodide (SmI_2_), the lactone intermediate was submitted to two sequential SmI_2_-mediated cyclizations to stereoselectively construct the polycyclic core of the natural product. Crucially, by exploiting the innate inner-sphere nature of carbonyl reduction using SmI_2_, we have used a steric blocking strategy to render sites SET-unreceptive and thus achieve chemoselective reduction in a complex substrate. Our asymmetric route enabled elucidation of the naturally occurring isomer of phaeocaulisin A and provides a synthetic platform to access other guaiane-type sesquiterpenes from *C. phaeocaulis*—as well as their synthetic derivatives—for medicinal chemistry and drug design.

## Introduction

**Figure 1:**
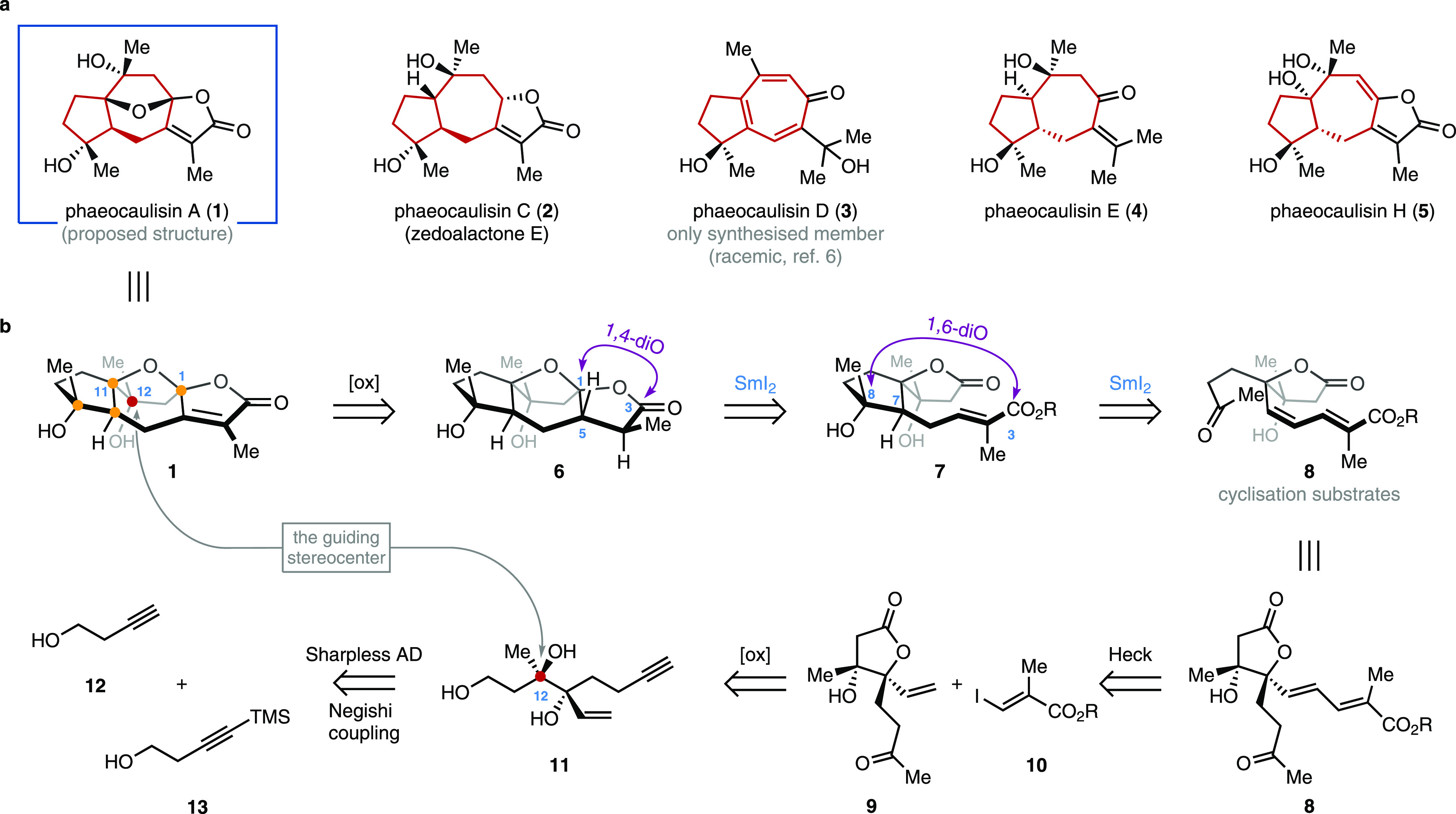
Guaiane-type sesquiterpenes from *C. phaeocaulis* and our strategy for the enantioselective synthesis of phaeocaulisin A. (a) Structurally diverse terpenoids, isolated from the rhizomes of *C. phaeocaulis*, exhibit enhanced anti-inflammatory activity. (b) Retrosynthetic analysis of one of the most biologically active and structurally intriguing members of the above class of guaiane-type sesquiterpene: phaeocaulisin A. The retrosynthesis builds on two SmI_2_-mediated cyclizations to forge the key 1,4-diO (C3-C1) and 1,6-diO (C3-C8) patterns through an umpolung strategy. The stereocenters are highlighted; the guiding stereocenter (C12) is established in triol **11** (highlighted in red).

Natural products extracted from the rhizomes of the widely distributed plant genus *Curcuma* (e.g., common turmeric) have long been known for their therapeutic properties, as exemplified by the use of these plants in traditional Indian and Chinese medicine.^1^ A recent report by the European Medicines Agency also highlights their potential societal worth.^2^ Among these compounds, guaiane-type sesquiterpenes—featuring the characteristic 5,7-fused carbocyclic skeleton and pendant methyl groups—have gained significant traction as privileged scaffolds due to their antitumor, anti-inflammatory, antioxidant, and antibacterial activity.^3^ In particular, phaeocaulisins—obtained from *Curcuma phaeocaulis* and *Curcuma wenyujin* (Figure 1a)^4^—have demonstrated, most importantly, the ability to inhibit lipopolysaccharide (LPS)-induced nitric oxide (NO) production in RAW 264.7 macrophages. Phaeocaulisin A (**1**), first isolated in 2013 from *C. phaeocaulis*, shows noteworthy inhibitory activity against NO production and, compared to other guaiane-type sesquiterpenes from its family, has a low IC_50_ value of 1.5 μM, thus making it a promising non-cytotoxic anti-inflammatory agent.^4^ Interestingly, preliminary structure–activity relationship studies indicated that its bioactive properties are tied to its characteristic acetal C1–C11 oxygen bridge, which is a unicum in guaiane-type sesquiterpenes.^4^ In addition, cell counting experiments and methyl thiazolyl tetrazolium (MTT) assays have highlighted the ability of phaeocaulisin A to potently suppress both growth and proliferation in A375 human melanoma cells.^5^ Its importance has been recognized by two patents describing its use as a targeted therapy drug for melanoma and as a treatment for autoimmune diseases associated with metabolic disorder of nitric oxide production.^5^ Despite these promising biological features and the rising relevance of guaiane-type sesquiterpenes in pharma, asymmetric synthetic routes to guaiane-type sesquiterpene lactones, phaeocaulisins,^6^ and, in particular, to phaeocaulisin A (**1**) are yet to be developed.

Intrigued by both its potential biological application as a targeted therapy for melanoma and its unique structure featuring a peculiar bridged acetal moiety, we set out to synthesize phaeocaulisin A (**1**). To this end, we took into consideration the following synthetic challenges: (i) the enantioselective construction of five stereocenters, four of which are contiguous and four are tetrasubstituted, and (ii) the formation of an acetal functionality, whose oxygen atoms are part of a bridged heterocycle and an unsaturated lactone ring. Our retrosynthetic analysis of **1** builds on the identification of the 1,4- and 1,6-dioxygenated patterns defined by the substituents at the C3–C1 and C3–C8 carbons embedded within structures **6** and **7**, respectively (Figure 1b).^7^ Taking advantage of the umpolung reactivity offered by the single-electron reduction of carbonyl compounds using the archetypal single-electron transfer (SET) reductant samarium(II) iodide (SmI_2_, Kagan’s reagent),^8^ we envisaged that both motifs can be built by two sequential SmI_2_-mediated couplings between the lactone and the ketone carbonyls of intermediates **7** and **8**, respectively, and a pendant-conjugated electron-deficient olefinic system—thus forging the characteristic 5,7-fused skeleton of the natural product (i.e., construction of the C1–C5 and C7–C8 bonds).^9^ These disconnections led to the identification of stereodefined lactone **8** as the key intermediate in our synthesis, whose three-dimensional arrangement would guide the diastereoselective formation of the three stereocenters generated during the SmI_2_-mediated cyclizations. Another synthetic design feature was the identification of an initially set stereocenter (C12, red) whose absolute stereochemistry would guide the construction of all other stereocenters. Therefore, for the synthesis of **8**, we aimed to design a short route featuring a single, established, enantioselective reaction; we anticipated that the Sharpless asymmetric dihydroxylation^10^ of a Negishi adduct,^11^ obtained from commercially available alkynes **12** and **13**, would allow the enantioselective synthesis of intermediate **8** in a few steps (Figure 1b). Our synthetic design not only provides, for the first time, an asymmetric route to phaeocaulisin A (**1**) but also potentially paves the way to the synthesis of other members of the guaiane-type sesquiterpene family extracted from *C. phaeocaulis* and their derivatives, thus enabling their study and application in medicinal chemistry.

## Results and Discussion

### Synthesis of the Cyclization Substrates

**Scheme 1:**
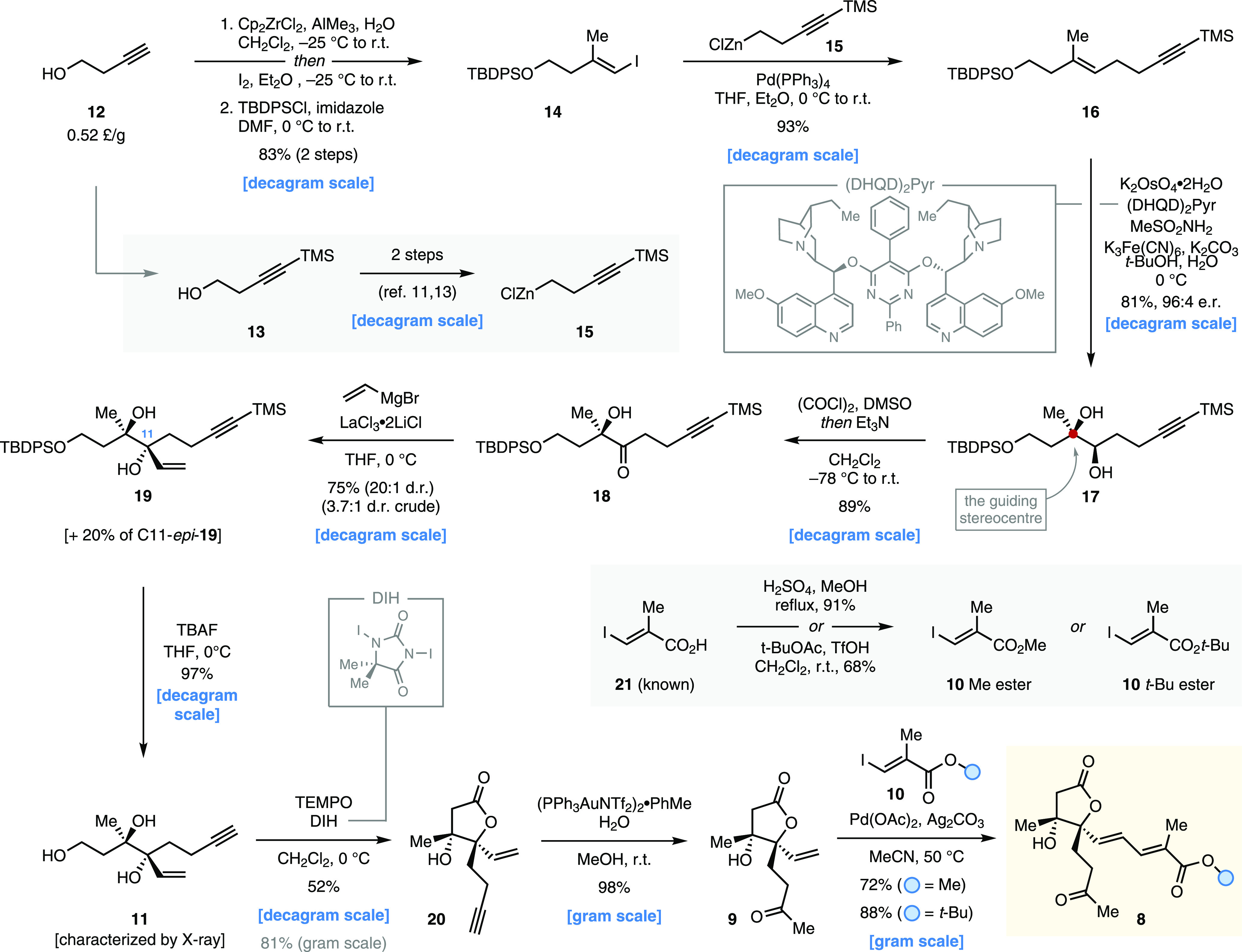
Enantioselective Synthesis of Key Intermediates **8** Synthetic route, summarizing reagents and conditions, to enantioenriched dienoates **8**: substrates for the SmI_2_-mediated cyclization reactions. Cp, cyclopentadienyl; DMF, dimethylformamide; TBDPS, *tert*-butyldiphenylsilyl; THF, tetrahydrofuran; TMS, trimethylsilyl; DMSO, dimethyl sulfoxide; TBAF, tetrabutylammonium fluoride; DIH, 1,3-diiodo-5,5-dimethylhydantoin.

Our synthetic endeavors commenced with the synthesis of lactone **8**, the substrate for the first proposed key SmI_2_-mediated reductive cyclization. Commercially available 3-butyn-1-ol **12** (∼0.50 £/g) was submitted to sequential *E*-selective alkyne carboalumination and in situ iodination,^12^ and then to the *tert*-butyldiphenylsilyl (TBDPS) protection of its primary alcohol functionality. This provided straightforward access to vinyl iodide derivative **14** in decagram quantities (Scheme 1). The latter was used as a coupling partner in a palladium-catalyzed Negishi coupling^11^ with trimethylsilyl-protected alkyl zinc reagent **15**, prepared in two steps from **13** following a previously reported procedure,^13^ thus affording 1,5-eneyne **16** in excellent yield. This set the stage for the introduction of the guiding stereocenter at C12 (phaeocaulisin A numbering) via Sharpless asymmetric alkene dihydroxylation.^10^ In an initial attempt, **16** was subjected to the standard enantioselective dihydroxylation conditions, using AD-mix-β and running the reaction at 0 °C. This furnished the desired *syn*-diol **17** in good yield, albeit in a moderate enantiomeric ratio (75% yield, 85:15 e.r.; see the SI). A survey of the most commonly adopted commercially available hydroquinidine-based ligands for the process identified (DHQD)_2_Pyr as the ligand of choice. Under these conditions, the desired mono-TBDPS-protected triol **17** was isolated in 81% yield and 96:4 e.r. on a decagram scale. The TBDPS-protecting group is vital to achieve high enantioselectivity since a smaller *tert*-butyldimethylsilyl (TBS) group failed to effectively shield one of the enantiotopic faces of the alkene, and the corresponding mono-protected triol was obtained with low levels of enantioinduction (60:40 e.r.). Crucially, this transformation defines the configuration of the tertiary alcohol stereocenter at C12 (c.f. numbering in phaeocaulisin A (**1**), Figure 1b, red), which dictates the diastereoselective installation of all of the other stereocenters in the natural product. The absolute stereochemistry of **17** was initially inferred based on the Sharpless mnemonic device^10^ and later corroborated by single-crystal X-ray crystallographic analysis of a more advanced intermediate (i.e., **7** Me ester, vide infra). Next, we sought to set the adjacent C11 stereocenter and, at the same time, install a vinyl handle; this serves as a strategic precursor for the dienoate functionality. Preliminary optimization studies performed on the TBS-protected analogue of **17** (see the SI) showed the oxidation of the secondary alcohol moiety to be challenging: even mild oxidants—including Dess–Martin periodinane (DMP),^14^ 2-iodoxybenzoic acid (IBX),^15^ SO_3_·py/DMSO (Parikh–Doering oxidation),^16^ and tetrapropylammonium perruthenate/NMO (TPAP, Ley oxidation)^17^—failed to give the desired hydroxyketone **18** in a synthetically useful yield, and oxidative cleavage of the 1,2-diol functionality was in all cases the preferred pathway (see the SI). To overcome this issue, we envisaged employing oxidation conditions in which the alcohol activator and the base are not present simultaneously, and the tertiary alcohol moiety is masked *in situ*. Based on this rationale, the TBS-protected analogue of **17** was submitted to classic Swern conditions using 2.0 equivalents of activated sulfoxide to give the desired mono-TBS-protected dihydroxyketone in excellent yield (86% yield; see the SI). Pleasingly, when these conditions were tested on the enantioenriched mono-TBDPS-protected triol **17**, the reactivity translated smoothly to afford decagram quantities of the corresponding dihydroxyketone **18** (89% yield). To ensure the diastereoselective installation of the stereocenter at C11, we envisioned a chelate-controlled Lewis acid-mediated Grignard addition to the ketone moiety of **18**. This was realized using the commercially available LaCl_3_·2LiCl complex,^18^ which served a dual role in (i) granting chelation between the carbonyl and the vicinal alcohol oxygen at C12—despite the bulky TBDPS group—and (ii) suppressing the detrimental enolization of the ketone functionality in the presence of excess vinylmagnesium bromide. The reaction was reliably carried out on the decagram scale to obtain the desired diastereomer **19** in 75% yield, together with 20% of the undesired isomer C11-*epi*-**19** (both diastereoisomers can be isolated separately by column chromatography) without erosion of the diastereoselectivity (3.7:1 d.r.) seen on a smaller scale. When other Lewis acids were trialed in this protocol, the Grignard addition reaction suffered from low diastereoselectivity or unproductive enolization was the dominant pathway, and starting material **18** was returned upon quenching. Simultaneous deprotection of both the primary alcohol and the alkyne moiety of **19** was achieved by treatment with tetrabutylammonium fluoride (TBAF) in THF. The reaction provided crystalline triol **11**, which was submitted to single-crystal X-ray analysis to establish its relative stereochemistry—this being in agreement with the Cram-chelate model for nucleophilic addition to ketones bearing α-stereocenters. The absolute configuration of **11** was later confirmed by the analysis of more advanced intermediates (i.e., **7** Me ester and **6**, vide infra) and is in agreement with the Sharpless mnemonic.^10^ Again, the 1,2-diol functionality within **11** proved to be sensitive toward most oxidation conditions, with oxidative cleavage of the C11–C12 bond proving facile (see the SI). Even the employment of Fetizon’s reagent (silver(I) carbonate on Celite)^19^—commonly used in oxidative lactonization protocols—failed to afford the desired lactone **20**. Extensive screening of conditions revealed that TEMPO-mediated oxidations were the most effective procedures to give **20**: using 1,3-diiodo-5,5-dimethylhydantoin (DIH) as the terminal oxidant, the desired lactone could be obtained in good yield (81% on gram scale), although the efficiency of the oxidation was lower on the decagram scale. At this stage, the pendant alkyne moiety of **20** was regioselectively hydrated under Au(I)-catalyzed conditions to provide methyl ketone **9**.^20^ Finally, building on preliminary optimization studies with a model substrate (see the SI), **9** was exposed to Ag-mediated, Pd-catalyzed Heck conditions and coupled first with known β-iodomethacrylate Me ester **10**(21) and later with its *tert*-butyl ester analogue (both prepared from known acid **21**(22)) to afford multigram quantities of both dienoates **8**. These substrates were used to test the feasibility of the key SmI_2_-mediated cyclization reactions.

### SmI_2_-Mediated Cyclizations

**Scheme 2:**
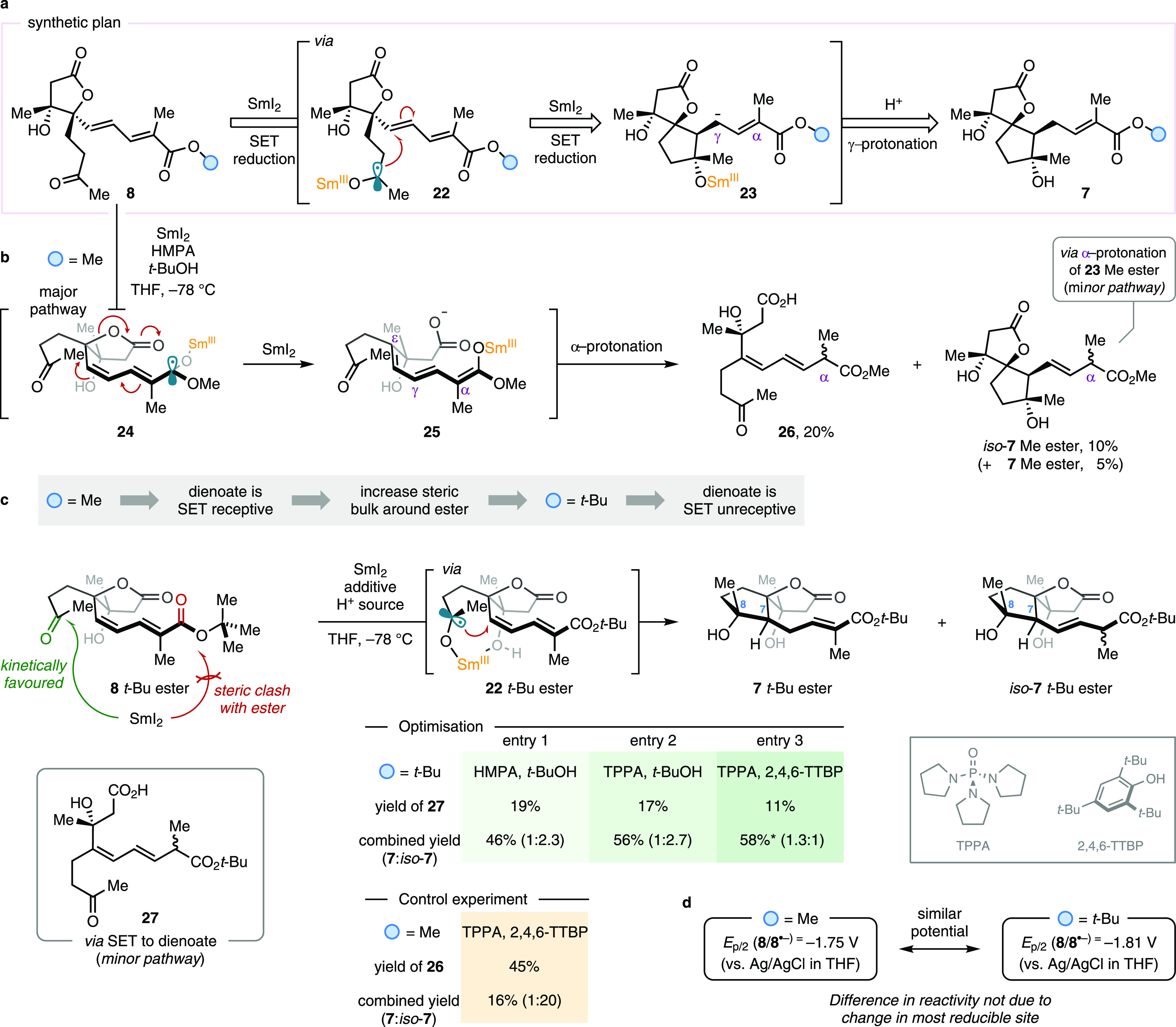
Synthetic Design and Optimization of the Key SmI_2_-Mediated Cyclization Yields determined by ^1^H NMR analysis using MeNO_2_ as the internal standard. Asterisks denote isolated yield. (a) Postulated reactivity in the SmI_2_-mediated cyclization of **8** to produce **7** through SET reduction of its ketone moiety. (b) An initial attempt using **8** Me ester afforded the undesired carboxylic acid **26** as the major product. (c) Increasing the steric bulk around the ester moiety of **8** alters the chemoselectivity of the SmI_2_ reduction: design and optimization of the key SmI_2_-promoted cyclization using **8***tert*-butyl ester as the substrate. (d) Electrochemical characterization of dienoates **8**. HMPA, hexamethylphosphoramide; THF, tetrahydrofuran; TPPA, tripyrrolidinophosphoric acid triamide; 2,4,6-TTBP, 2,4,6-tri-*tert*-butylphenol.

With an efficient route in hand to stereoselectively access enantiomerically enriched lactone dienoates **8** on the gram scale, we tackled the development of the SmI_2_-mediated cyclizations that would construct the polycyclic skeleton of phaeocaulisin A (**1**). According to the synthetic plan outlined in Figure 1, we first investigated the cyclization of **8** to deliver spirocyclic enoate **7**. It was envisaged that SET reduction of the ketone moiety of **8** by SmI_2_ would trigger 5-*exo*-trig cyclization of the resulting ketyl radical **22** onto the pendant-conjugated diene, thus forming the spirocyclic all-carbon five-membered ring of the natural product (Scheme 2a). Further reduction of the radical intermediate stemming from the cyclization event—by another equivalent of SmI_2_—and subsequent γ-protonation of the so-formed extended enolate **23** would deliver compound **7**. One anticipated challenge was the tendency of extended enolates of conjugated esters to give mixtures of α- and γ-protonated products or favoring α-protonation depending on the cation (known as the extended enolate problem).^7^ The SmI_2_-mediated cyclization reaction was initially trialed using the methyl ester analogue of dienoate **8** (Scheme 2b). Building on model studies (see the SI), preliminary experiments were performed using 2.2 equivalents of SmI_2_, in both the absence and presence of various proton sources and HMPA at −78 °C. Unfortunately, all of these attempts yielded the desired cyclization products in low amounts (≤15% NMR yield) as a mixture of alkene regioisomers **7** and *iso*-**7**, the latter (obtained via α-protonation of intermediate **23**) undesirably being the major component of the mixture. Even though the combined yield of **7** Me ester was low in all cases, the high levels of diastereoselectivity with which the two new stereocenters were generated were encouraging. Importantly, in all cases, the preferred pathway was the undesired opening of the lactone ring to form carboxylic acid by-product **26**. This unwanted reactivity arises from SET from SmI_2_ to the dienoate fragment of **8**—as opposed to its ketone moiety—thus generating radical intermediate **24** (Scheme 2b). This, through sequential lactone ring opening and a second SmI_2_-promoted SET reduction, delivers carboxylate **25**, which, upon α-protonation of its Sm-enolate functionality, gives **26**. In order to avoid this pitfall, we sought to kinetically disfavor the reduction of the dienoate system within **8** by increasing the steric bulk around the ester moiety; SmI_2_ reduces carbonyls via an inner-sphere electron transfer mechanism, which requires coordination of the metal center to the carbonyl oxygen prior to the SET event.^23^ Therefore, we proposed that the use of the more sterically encumbered *tert*-butyl ester analogue of **8**—in place of the previously employed methyl ester—would render the dienoate moiety unreceptive to SET due to unfavorable steric interactions, thus fostering the formation of ketyl radical **22** and driving the desired cyclization reaction (Scheme 2c). Pleasingly, when **8***tert*-butyl ester was submitted to the previously employed cyclization conditions (2.2 equivalents of SmI_2_, in the presence of HMPA, *t*-BuOH, and THF and at −78 °C), the desired spirocyclic product **7***tert*-butyl ester was obtained in 46% NMR yield, albeit as a 1:2.3 mixture of regioisomers **7** and *iso*-**7** (Scheme 2c, optimization, entry 1). Crucially, the reaction remained highly diastereoselective for the desired C7, C8 isomer as confirmed by single-crystal X-ray crystallographic analysis of a later intermediate (i.e., **6**, vide infra). The use of tripyrrolidinophosphoric acid triamide (TPPA)—a nontoxic alternative to the carcinogenic and mutagenic HMPA—as the Lewis basic ligand for SmI_2_, and *t*-BuOH as the proton source, boosted the efficiency of the cyclization reaction, affording the spirocyclic product in 56% NMR yield with 1:2.7 r.r., again in favor of *iso*-**7** (entry 2). To reverse the observed regioselectivity and obtain selectively the desired isomer **7**, we screened different proton sources; we envisaged that their steric properties could influence the ratio of site protonation upon quenching of the enolate intermediate (α- vs γ-protonation, c.f. structure **23** in Scheme 2a). A survey of various proton sources identified the use of bulky 2,4,6-tri-*tert*-butylphenol (2,4,6-TTBP) as optimal (see the SI); the formation of **7***tert*-butyl ester over *iso*-**7** was now favored (1.3:1 r.r.) in good isolated yield and with excellent diastereoselectivity even on a 1 mmol scale (entry 3). Under these conditions, the formation of the corresponding lactone ring-opening product **27** was limited to 11% NMR yield. The size of the proton source seems to be the main parameter influencing the protonation process, since a relationship between the p*K*_a_ of the proton sources and either the combined yield or regioselectivity of the process could not be established. To confirm the role of the *tert*-butyl ester functionality, we performed a control experiment subjecting **8** Me ester to the optimized SmI_2_-mediated conditions (using TPPA and 2,4,6-TTBP). As expected in this case, the reaction delivered prevalently ring-opened carboxylic acid **26** together with *iso*-**7**, while the desired cyclization product **7** Me ester was observed only in low yield. Interestingly, cyclic voltammetry studies showed that the most reducible site of **8** remains the dienoate system regardless of the substitution at the ester moiety, thus underlining the operation of kinetic control in the selective SET reduction of the ketone moiety (Scheme 2d). Remarkably, this study represents a rare example in which the chemoselectivity of a SmI_2_-mediated SET reduction can be altered by rationally exploiting the innate inner-sphere electron transfer mechanism of SmI_2_.^23^

**Scheme 3:**
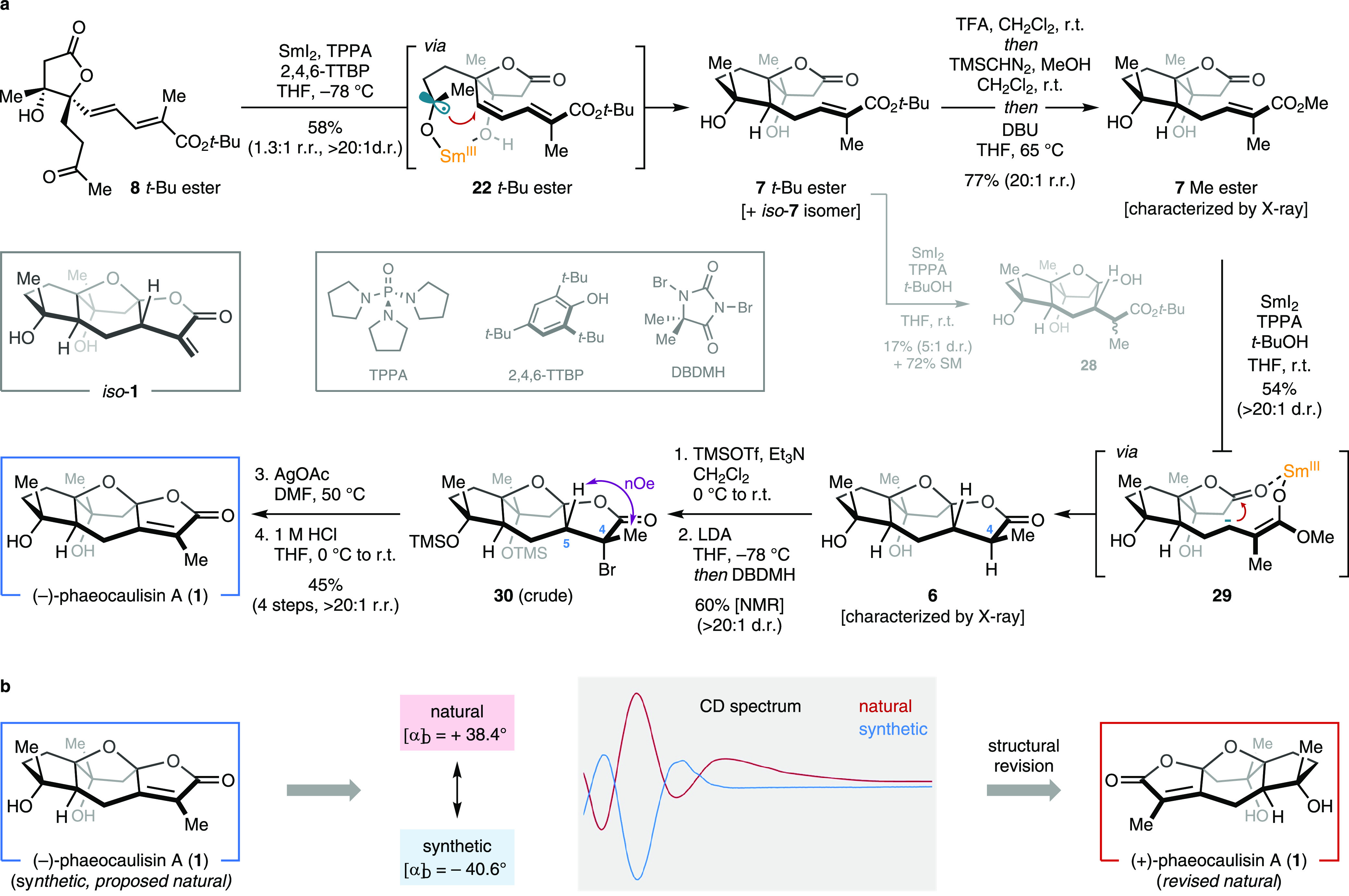
Completion of the Total Synthesis of (−)-Phaeocaulisin A and Structural Revision of Its Naturally Occurring Enantiomer (a) The final steps toward the synthesis of (−)-phaeocaulisin A, featuring the two key SmI_2_-mediated cyclizations and the installation of the endocyclic double bond. (b) Structural revision of the naturally occurring (+)-phaeocaulisin A based on the comparison of both the specific rotation and the CD spectrum of the synthetic and the natural sample. TPPA, tripyrrolidinophosphoric acid triamidetris(*N*,*N*-tetramethylene)phosphoric acid triamide; 2,4,6-TTBP, 2,4,6-tri-tripyrrolidinophosphoric acid triamide; 2,4,6-TTBP, 2,4,6-tri*-tert*-butylphenol; TFA, trifluoroacetic acid; DBU, 1,8-diazabicyclo[5.4.0]undec-7-ene; THF, tetrahydrofuran; TMSOTf, trimethylsilyl trifluoromethanesulfonate; LDA, lithium diisopropylamide; DBDMH, 1,3-dibromo-5,5-dimethylhydantoin; SM, starting material.

While the presence of the bulky *tert*-butyl ester group renders the conjugated ester moiety unreceptive to SET, thus promoting the first radical cyclization reaction (from **8** to **7**), for the same reason, it would disfavor the second planned SmI_2_-mediated cyclization (c.f. Figure 1b, from **7** to **6**); SET from SmI_2_ to the electron-deficient olefin within **7** initiates the process (Scheme 3a). Accordingly, exposure of **7***tert*-butyl ester to the SmI_2_-mediated cyclization conditions (*vide infra*) provided the tricyclic product **28** in low yield and with low diastereoselectivity, with the majority of the starting material being recovered. To prepare **7** for the reductive cyclization process, we exchanged the *tert*-butyl ester group for its Me ester analogue and converted the inseparable unconjugated *iso*-**7** isomer into **7** Me ester via base-assisted isomerization. Compound **7** Me ester was then treated with 2.2 equivalents of SmI_2_ in the presence of *t*-BuOH to afford the desired bridged seven-membered ring structure (i.e., Me ester analogue of **28**) in 30% yield. The use of other proton sources, such as H_2_O, promoted reduction of the alkene bond, with no cyclization being observed. Crucially, addition of TPPA, in conjunction with *t*-BuOH, triggered the desired 6-*exo*-trig/lactonization cascade process and allowed the direct isolation of lactone **6**—unequivocally characterized by single-crystal X-ray crystallography—from **7** Me ester with high diastereocontrol (>20:1 d.r.), thus completing the assembly of the tetracyclic skeleton of the natural product (Scheme 3a). Mechanistically, we believe that the seven-membered ring formation proceeds via intermediate **29**, stemming from two sequential SETs from SmI_2_ to the conjugated alkene system of **7**.

### Endgame and Structural Revision of the Natural Product

Having built the bridged tetracyclic architecture of phaeocaulisin A, we aimed to complete our synthetic route by introducing the unsaturation between the C4 and C5 carbons in the natural product (Scheme 3a). Initially, we planned to achieve this formal oxidation of **6** via a *syn*-selenoxide elimination from the corresponding C4-substituted phenyl selenide (not shown, see the SI) to deliver the endocyclic alkene product selectively. To this end, we protected the tertiary alcohol groups of **6** as trimethylsilyl (TMS) ethers to obtain its C8- and C12-OTMS derivative in almost quantitative yield. Subsequent deprotonation of the lactone moiety using lithium diisopropylamide (LDA)—forming the corresponding enolate—and quenching with phenylselenyl chloride (PhSeCl) afforded a separable mixture of two diastereomers of the targeted selenide (4:1 d.r.). Surprisingly, despite the presence of the two bulky OTMS groups, ^1^H nOe NMR experiments indicated that the *endo*-isomer—obtained by the electrophile approaching the Li-enolate on the bottom face—was the major component of the mixture. With selenium installed on the bottom face (C4-Se and C5-H in *anti*-arrangement), *syn*-elimination upon oxidation of the Se-atom could only promote the formation of the exocyclic double bond, thus giving rise to phaeocaulisin A regioisomer *iso*-**1**. Isomerization of the exocyclic double bond to deliver **1** could not be achieved under a variety of conditions (see the SI). Therefore, we sought to exploit the *endo*-selectivity seen in the lactone α-functionalization process to install a leaving group at C4 that could undergo elimination via an *anti*-mechanism and provide access to the endocyclic double bond of **1**. α-Bromination was chosen as previous reports have shown that *anti*-elimination can be promoted in kindred systems by the silver acetate (AgOAc)-assisted cleavage of C-Br bonds. Pleasingly, protection of the tertiary alcohols of **6** (as above) followed by α-deprotonation of the lactone moiety using LDA and quenching of the Li-enolate with 1,3-dibromo-5,5-dimethylhydantoin (DBDMH) yielded the desired *endo*-bromide **30** in 60% NMR yield and with excellent diastereoselectivity (>20:1 d.r.). *Anti*-elimination was then accomplished by treating crude **30** with AgOAc in DMF at 50 °C to afford the endocyclic unsaturated product almost exclusively. Finally, deprotection of the silyl ethers with aqueous HCl in THF furnished phaeocaulisin A in 45% overall yield over four steps. Our total synthesis was designed based on the absolute stereochemical configuration of the naturally occurring enantiomer of phaeocaulisin A reported in the contribution describing its isolation^4^ assigned based on empirical rules using its circular dichroism (CD) spectrum.^24^ However, the specific rotation recorded for our synthetic sample was of the same magnitude, but of opposite sign, with respect to that of the isolated natural product. In addition, we found that the CD spectrum of our sample was opposite to that of the isolated sample (Scheme 3b). This indicates that the original absolute stereochemistry of phaeocaulisin A was misassigned and the natural occurring sesquiterpene extracted from *C. phaeocaulis* is actually the enantiomer of the synthesized compound (−)-**1**. Interestingly, this example provides an exception to the empirical rule used in CD to assign the absolute stereochemistry of α,β-unsaturated γ-lactones based on their characteristic Cotton effect at specific wavelengths.^24^ More importantly, this study underlines once again the paramount role of total synthesis in confirming the structure of natural products.

## Conclusions

We have developed an asymmetric route to the guaiane-type sesquiterpene phaeocaulisin A: a 17-step sequence delivers the target compound in 2% overall yield. Our strategy builds on the enantioselective synthesis of a lactone dienoate intermediate, which is then “folded” using two sequential diastereoselective SmI_2_-mediated cyclizations to construct the unique tetracyclic framework of the natural product. Crucially, we have used a steric blocking strategy to render sites in a complex substrate SET-unreceptive, overriding natural reactivity and achieving chemoselectivity in a reductive cyclization—a strategy that is unprecedented in SmI_2_ chemistry. Through our synthesis, we have identified and amended the absolute stereochemical configuration assigned to the naturally occurring enantiomer of (+)-phaeocaulisin A. Our route prepares the ground for the synthesis of other sesquiterpenes from *C. phaeocaulis* as well as their synthetic derivatives. Given the biological activity of phaeocaulisin A and guaiane-type sesquiterpenes in general, we believe that synthetic studies, such as ours, will promote their evaluation and exploitation in biology and medicine.

## Supporting Information Available

Materials and methods; experimental procedures; optimization studies; useful information; ^1^H NMR spectra; ^13^C NMR spectra; CD spectra; mass spectrometry data; specific rotations (PDF)

## Accession Codes

CCDC 2123220–2123222 and 2125350 contain the supplementary crystallographic data for this paper. These data can be obtained free of charge via www.ccdc.cam.ac.uk/data_request/cif, or by emailing data_request@ccdc.cam.ac.uk, or by contacting The Cambridge Crystallographic Data Centre, 12 Union Road, Cambridge CB2 1EZ, UK; fax: +44 1223 336033.
