# Supplementary material for: Asymmetric Total Synthesis of (−)-Phaeocaulisin A
Source: J Am Chem Soc. 2022 Apr 13;144(16):7457–64. doi: 10.1021/jacs.2c02188 (PMC9490872; doi:10.1021/jacs.2c02188)
Supplement: Supplementary file 1 — ja2c02188_si_001.pdf [file ja2c02188_si_001.pdf]

Asymmetric total synthesis of (–)-phaeocaulisin A

Áron Péter[1], Giacomo E. M. Crisenza[1], & David J. Procter[1]*

1 – Department of Chemistry, The University of Manchester, Oxford Road, Manchester, M13 9PL (UK).
*email: david.j.procter@manchester.ac.uk.

Supporting information

Table of contents

1. General information 3
2. Summary of our synthesis of (–)-phaeocaulisin A 4
3. Synthetic Procedures and Characterisations 5
4. Unsuccessful approaches 48
5. Model studies towards (–)-phaeocaulisin A (1) 49
6. X-Ray Crystal structures 50
7. Cyclic voltammetry (CV) studies 55
8. Circular dichroism (CD) spectrum of (–)-phaeocaulisin A (1) 65
9. ^{1}H and ^{13}C NMR spectra 66
10. References 110

1. General information

All air and/or moisture sensitive reactions were performed under an atmosphere of dry nitrogen using anhydrous solvents and standard Schlenk techniques. The glassware used for such reactions was flame dried and cooled under a flow of nitrogen. Other reactions were carried out open to air as noted in each case.

SmI_2 solution (0.1 M in THF) was prepared according to a literature procedure and used within 3 days[1]. Freshly distilled THF (Na/benzophenone) was used for all SmI_2 reactions. All other solvents and reagents were purchased from commercial sources at the highest available grade and used as supplied unless otherwise noted.

Flash column chromatography was carried out using 35 – 70 μm, 60 Å silica gel, and dry column vacuum chromatography (DCVC)[2,3] using 20 – 45 μm, 60 Å silica gel (Merck catalogue number: 1.15111.1000). Preparative TLCs were carried out using 20 × 20 cm glass plates 500 μm or 2000 μm thickness. Routine TLC analysis was carried out on aluminium sheets coated with silica gel 60 F254, viewed under 254 nm ultraviolet lamp and/or visualized by anisaldehyde, $KMnO_4$, vanillin, or cerium ammonium molybdate.

^{1}H, ^{13}C NMR spectra were recorded using Bruker Avance III 600, Bruker AVIII HD 500, Bruker AVIII HD 400 or Bruker AVIII 400 spectrometers, with chemical shift values being reported in ppm relative to the corresponding residual solvent signal ($CDCl_3$ – 7.26 ppm ^{1}H NMR, 77.16 ppm ^{13}C NMR, CD_3OD – 3.31 ppm ^{1}H NMR, 49.00 ppm ^{13}C NMR). All coupling constants (*J*) are reported in Hertz (Hz). Splitting patterns are assigned as s = singlet, bs = broad singlet, d = doublet, t = triplet, q = quartet, p = pentet, sx = sextet, m = multiplet, and app = apparent.

Accurate mass spectra were obtained using positive and/or negative electrospray ($ES^{\pm}$) or atmospheric-pressure chemical ionization ($APCI^{\pm}$) techniques. Measurements were carried out by the Mass spectrometry Service, Department of Chemistry, University of Manchester.

IR spectra were recorded on an ATR FTIR spectrometer as evaporated films (from acetone, CH_2Cl_2 or MeOH) or using neat samples and are reported in wavenumbers.

Enantiomeric excess of chiral samples was determined by HPLC-UV or GC-FID using a chiral stationary phase.

Specific rotations were measured on a Rudolph Research Analytical Autopol I Automatic Polarimeter.

Melting points were measured of solids as obtained after column chromatography using a Stuart Scientific capillary melting point apparatus and are uncorrected.

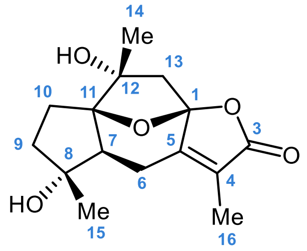

Figure S1. Carbon numbering. The carbon numbers are adopted from the IUPAC name of (–)-phaeocaulisin A (**1**) [(1S,7S,8R,11R,12R)-8,12-Dihydroxy-4,8,12-trimethyl-2,14-dioxatetracyclo [9.2.1.0^{1,5}.0^{7,11}]tetradec-4-en-3-one]. Consequently, there is no C2 in our carbon numbering system. Our numbering is different to that used in the isolation paper[4]. The numbering of all carbons in the intermediates is based on our numbering of (–)-phaeocaulisin A (**1**).

2. Summary of our synthesis of (–)-phaeocaulisin A

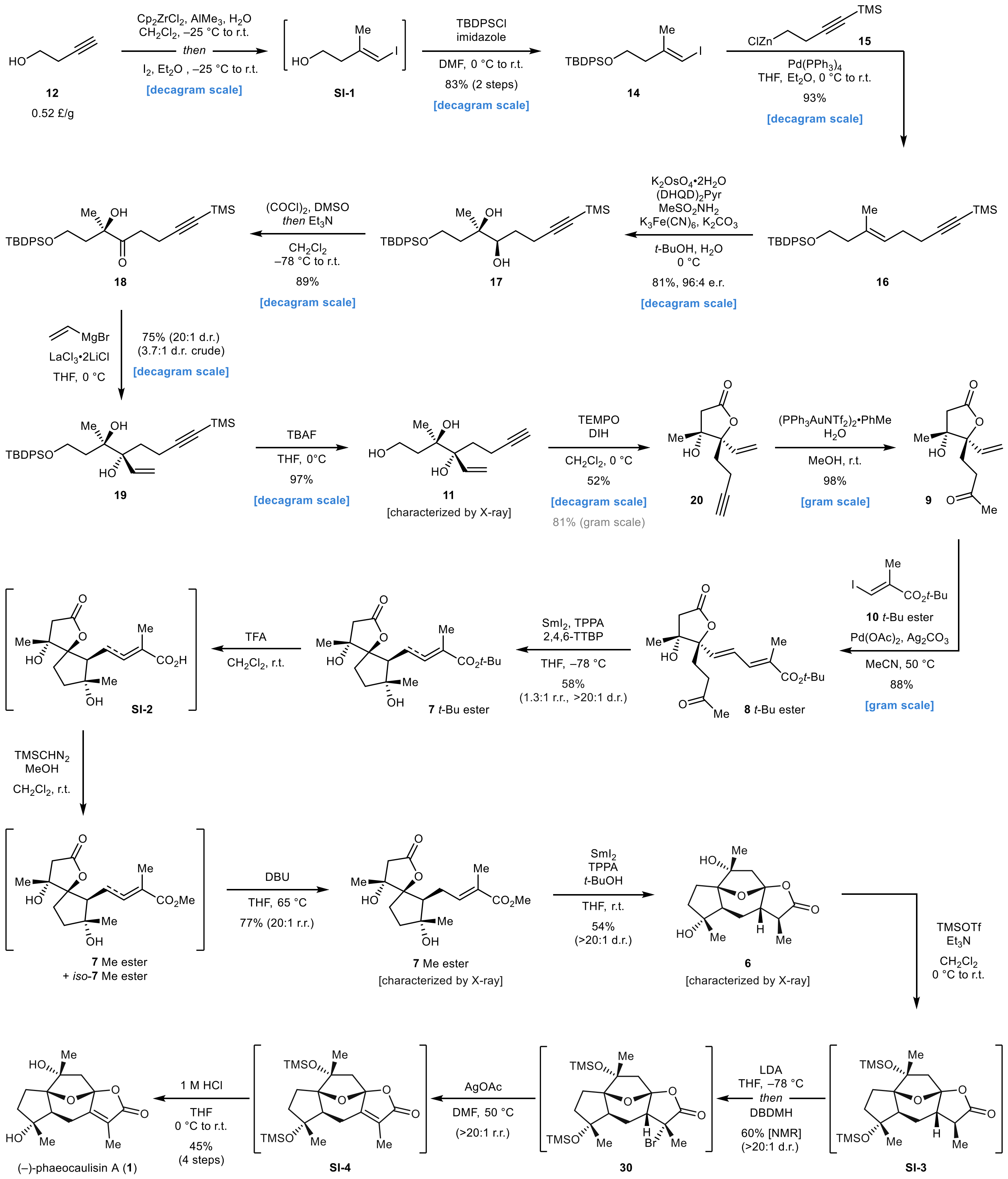

Scheme S1. Summary of our 17-step synthesis of (–)-phaeocaulisin A.

Each reaction step is defined as one in which a substrate is converted to a product in a single reaction flask (irrespective of the number of transformations) without intermediate workup in a separate flask or purification. Compounds in brackets are not isolated as pure compounds (even though they could be) but carried forward as crude products.

3. Synthetic Procedures and Characterisations

Preparation of vinyl iodide **14**: carboalumination and TBDPS protection of 3-butynol (**12**)

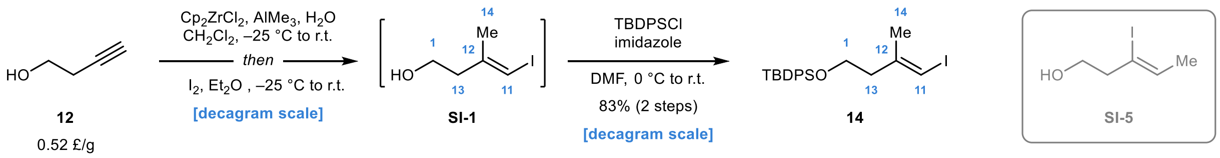

Step 1: the following is a modification of a literature procedure[5]. A flame-dried tri-neck 2 litre round-bottom flask was equipped with a magnetic stirrer and charged with Cp_2ZrCl_2 (0.22 eq., 31.4 mmol, 9.17 g). The flask was evacuated and refilled with dry N_2 three times. Dry CH_2Cl_2 (615 ml) was added *via* cannula and the solution was cooled to –25 °C, then $AlMe_3$ solution (2 M in hexanes, 3.1 eq., 442 mmol, 221 ml) was added dropwise *via* cannula. The resulting yellow solution was stirred for 15 min at –25 °C before H_2O (1.55 eq., 221 mmol, 3.98 ml) was carefully added dropwise. ***Note:*** *the addition of water is exothermic and produces a large amount of gas! The mixture should not be cooled below –30 °C as water freezes in the mixture resulting in inferior yields.* After an additional 15 min stirring at –25 °C, 3-butyn-1-ol (**12**, 1.0 eq., 142.7 mmol, 10.0 g, 10.8 ml), pre-treated with $AlMe_3$ (2 M in hexanes, 0.31 eq., 44.2 mmol, 22.1 ml) in dry CH_2Cl_2 (120 mL) at 0 °C for 10 min, was added dropwise *via* cannula at –25 °C. The reaction mixture was warmed to room temperature and stirred for 2.5 h. Then, the mixture was cooled to –25 °C again, and a solution of I_2 (1.5 eq., 214 mmol, 54.3 g) in dry Et_2O (230 ml) was added dropwise *via* cannula. The reaction was allowed to warm to room temperature and stirred for 3 h. After this, the reaction was cooled to 0 °C and slowly quenched with saturated aq. Rochelle's salt solution (1200 ml) and Et_2O (700 ml). The mixture was stirred for 48 h before the phases separated. The aqueous phase was extracted with Et_2O (3 × 500 ml), the combined organic layers were dried over $MgSO_4$, filtered and concentrated *in vacuo*. The product was used without further purification in the next step.

Note: The product **SI-1** is moderately volatile and contains some of the regioisomeric product **SI-5** (**SI-1**:**SI-5** = 15.6:1). However, this side-product, **SI-5**, and derivatives thereof, are separated after the Sharpless Asymmetric Dihydroxylation step (*vide infra*) by flash column chromatography.

Analytical sample of **SI-1** was obtained by flash column chromatography on silica gel (50% Et_2O in hexanes).

^{1}H NMR (400 MHz, $CDCl_3$) δ 6.02 (sx, J = 1.1 Hz, 1H, C11*H*), 3.72 (q, J = 5.9 Hz, 2H, C1H_2), 2.48 (td, J = 6.2, 1.1 Hz, 2H, C13H_2), 1.87 (d, J = 1.1 Hz, 3H, C14H_3), 1.41 (t, J = 5.5 Hz, 1H, O*H*).
^{13}C NMR (101 MHz, $CDCl_3$) δ 144.7 (C12), 77.0 (C11), 60.3 (C1), 42.6 (C13), 23.9 (C14).
HRMS ($APCI^+$) calculated for $C_5H_{10}OI$ $[M+H]^+$ 212.9771, found 212.9764 (–3.29 ppm).
IR (ν_{max}/cm^{-1}, neat) 3326, 2912, 1376, 1271, 1141, 1040.
TLC (30% EtOAc in hexanes): R_f = 0.44 (anisaldehyde).
Physical state: yellow oil.

Data is in agreement with literature[6].

Step 2: crude alcohol **SI-1** (around 125 mmol, assumed yield for step 1: 90%) was dissolved in dry DMF (100 ml) under dry N_2. The solution was cooled to 0 °C, then imidazole (1.4 eq., 175 mmol, 11.9 g) and TBDPSCl (1.23 eq., 154 mmol, 42.3 g, 39.4 ml) were added consecutively. The resulting solution was stirred at 0 °C for 30 min before warming to r.t. and stirring for an additional 16 h. TLC analysis of the reaction mixture indicated complete consumption of the starting material (50% Et_2O in hexanes). The reaction was then quenched with saturated aq. $NaHCO_3$ solution (150 ml) and pentane (150 ml). The phases were separated, the aqueous layer was extracted with pentane (5 × 150 ml) and the combined organic layers were dried over $MgSO_4$, filtered and concentrated *in vacuo*. The crude product was purified on a short silica gel pad (1% Et_2O in petroleum ether 40-60) to give the title compound **14** (53.4 g, 118 mmol, 83% over 2 steps) as a colourless oil.

^{1}H NMR (400 MHz, $CDCl_3$) δ 7.74 – 7.59 (m, 4H, Ar*H*), 7.47 – 7.33 (m, 6H, Ar*H*), 5.93 (q, *J* = 1.1 Hz, 1H, C11*H*), 3.73 (t, *J* = 6.4 Hz, 2H, C1H_2), 2.44 (td, *J* = 6.4, 1.0 Hz, 2H, C13H_2), 1.76 (d, *J* = 1.2 Hz, 3H, C14H_3), 1.05 (s, 9H, C*Me*$_3$).
^{13}C NMR (126 MHz, $CDCl_3$) δ 145.2 (C12), 135.7 (Ar*C*H), 133.8 (Ar*C*), 129.8 (Ar*C*H), 127.8 (Ar*C*H), 76.7 (C11), 62.0 (C1), 42.4 (C13), 27.0 (C*Me*$_3$), 24.2 (C14), 19.3 (*C*Me$_3$).
HRMS (APCI$^+$) calculated for $C_{21}H_{28}OISi$ $[M+H]^+$ 451.0949, found 451.0949 (0.00 ppm).
TLC (1% Et_2O in hexanes): R_f = 0.51 (anisaldehyde)
Physical state: colourless oil.

Preparation of alkyl iodide **SI-6**: Appel reaction of 4-(trimethylsilyl)but-3-yn-1-ol (**13**)

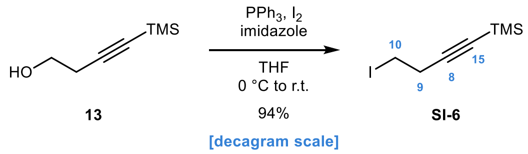

Following a modified literature procedure[7], 4-(trimethylsilyl)but-3-yn-1-ol (**13**, 1.0 eq., 165 mmol, 23.5 g, 27.49 mmol) was dissolved in dry THF (660 ml) under N_2. After cooling to 0 °C, PPh_3 (1.0 eq., 165 mmol, 43.3 g), imidazole (1.0 eq., 165 mmol, 11.2 g), and I_2 (1.0 eq., 165 mmol, 41.8 g) were added consecutively *via* a powder funnel under air after the previous reagent was completely dissolved. The ice-bath was removed after 5 min and the reaction was stirred at room temperature for 2 h. After this, an additional portion of PPh_3 (0.25 eq., 41.3 mmol, 10.8 g), imidazole (0.25 eq., 41.3 mmol, 2.81 g), and I_2 (0.25 eq., 41.3 mmol, 8.36 g) were added consecutively *via* a powder funnel under air after the previous reagent was completely dissolved. The mixture was stirred for another hour at r.t, TLC indicated complete consumption of the alcohol starting material (30% EtOAc in hexanes). After this, the mixture was diluted with Et_2O (450 ml) and quenched with 10% aq. $Na_2S_2O_3$ (450 ml). The phases were separated, and the aqueous layer was washed with Et_2O (2 × 150 ml). The combined organic layers were dried over $MgSO_4$, filtered and concentrated *in vacuo*. The crude product was purified by Kugelrohr distillation (95 °C, 1 mbar) to give the title compound (39.1 g, 155 mmol, 94%) as a colourless oil.

Note: The product is moderately unstable towards flash column chromatography. It can be purified by eluting with petroleum ether 40-60 on a short silica pad. The resulting pink oil was then dissolved in hexanes and washed with 10% aq. $Na_2S_2O_3$ to give the title compound, after evaporation, as a colourless oil. On large scale, Kugelrohr distillation is more convenient.

^{1}H NMR (400 MHz, $CDCl_3$) δ 3.22 (t, *J* = 7.5 Hz, 2H, C10*H*$_2$), 2.79 (t, *J* = 7.5 Hz, 2H, C9*H*$_2$), 0.16 (s, 9H, Si*Me*$_3$).
^{13}C NMR (101 MHz, $CDCl_3$) δ 105.2 (C8), 86.9 (C15), 25.2 (C9), 1.2 (C10), 0.1 (Si*Me*$_3$).
HRMS (APCI$^+$) calculated for $C_7H_{14}SiI$ $[M+H]^+$ 252.9904, found 252.9896 (–3.2 ppm).
IR (ν_{max}/cm^{-1}, neat) 2957, 2174, 1420, 1247, 1170, 992, 836.
TLC (30% EtOAc in hexanes) R_f = 0.66 (anisaldehyde).
Physical state: colourless oil.

Data is in agreement with literature[7].

Preparation of 1,5-enyne **16**: Negishi coupling of alkyl idodide **SI-6** and vinyl iodide **14** *via* **15**

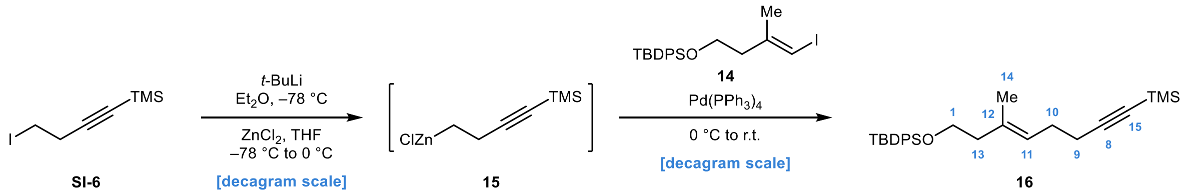

Following a modified literature procedure[8], *t*-BuLi (3.0 eq., 193 mmol, 113 ml, 1.7 M in pentane) was added to dry Et_2O (200 ml) under dry N_2, then the solution was cooled to –78 °C. Alkyl iodide **SI-6** (1.5 eq. 96.5 mmol, 24.3 g) was dissolved in dry Et_2O (290 ml), cooled to –78 °C, then added dropwise to the cold *t*-BuLi solution *via* cannula. The resulting reaction mixture was stirred for 1.5 h at –78 °C, following this $ZnCl_2$ solution (2.0 eq., 128.6 mmol, 184 ml, 0.7 M in THF) was added dropwise *via* cannula. The resulting mixture was allowed to warm to 0 °C over 1 h. In the meantime, a separate 3-neck flask was charged with vinyl iodide **14** (1.0 eq., 64.3 mmol, 28.9 g) and $Pd(PPh_3)_4$ (0.035 eq., 2.27 mmol, 2.54 g) then cooled to 0 °C. The previously prepared zinc reagent **15** was transferred to the flask containing the mixture of vinyl iodide and $Pd(PPh_3)_4$ *via* cannula. The resulting reaction mixture was allowed to warm to r.t. overnight. To quench the reaction, water (600 ml) was added, then the pH was adjusted with HCl (1 M) to dissolve the zinc by-products. The phases were separated, the aqueous phase was washed with Et_2O (2 × 600 ml) and the combined organic layers were dried over $MgSO_4$, filtered and concentrated *in vacuo*. The crude product was purified by column chromatography on silica gel (1 to 2% Et_2O in petroleum ether 40-60) to give the title compound **16** (27.3 g, 60.9 mmol, 95%) as a colourless oil.

^{1}H NMR (500 MHz, $CDCl_3$) δ 7.74 – 7.57 (m, 4H, Ar*H*), 7.43 – 7.36 (m, 6H, Ar*H*), 5.18 (s, 1H, C11*H*), 3.73 (t, *J* = 6.9 Hz, 2H, C1H_2), 2.25 (t, *J* = 6.9 Hz, 2H, C13H_2), 2.21 (m, 4H, C10H_2 and C9H_2), 1.56 (d, *J* = 1.8 Hz, 3H, C14H_3), 1.05 (s, 9H, C*Me*$_3$), 0.14 (s, 9H, Si*Me*$_3$).

^{13}C NMR (126 MHz, $CDCl_3$) δ 135.7 (Ar*C*H), 134.2 (Ar*C*), 133.7 (C12), 129.7 (Ar*C*H), 127.7 (Ar*C*H), 124.8 (C11), 107.5 (C8), 84.4 (C15), 63.0 (C1), 42.9 (C13), 27.6 (C10), 27.0 (C*Me*$_3$), 20.4 (C9), 19.3 (*C*Me$_3$), 16.5 (C14), 0.3 (Si*Me*$_3$).

HRMS (ES^+) calculated for $C_{28}H_{40}OSi_2Na$ $[M+Na]^+$ 471.2510, found 471.2499 (–2.3 ppm).

TLC (1% Et_2O in hexanes): R_f = 0.32.

Physical state: colourless oil.

Preparation of (±)-**SI-8**: dihydroxylation of 1,5-enyne **SI-7**

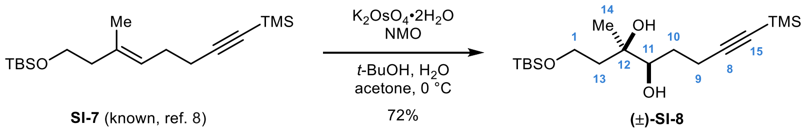

(±)-**SI-8** was synthesised analogously to (±)-**17** (*vide infra*) from **SI-7**[8] and isolated in 72% yield (258 mg, 0.719 mmol).

Note: ^{1}H NMR, ^{13}C NMR, and TLC data matches that of **SI-8**.

HRMS (APCI$^+$) calculated for $C_{18}H_{39}O_3Si_2$ [*M+H*]$^+$ 359.2432, found 359.2435 (+0.84 ppm).
Physical state: pale-yellow oil.

Preparation of **SI-8**: Sharpless asymmetric dihydroxylation of 1,5-enyne **SI-7**

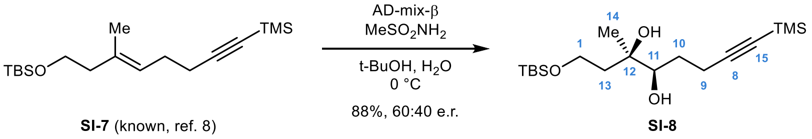

SI-8 was synthesised following the general recommendations outlined by Sharpless *et al.*[9] from **SI-8** and isolated in 88% yield (315 mg, 0.878 mmol).

Note: The enantioselectivity of the reaction was determined after Swern oxidation of **SI-8** (*vide infra*). The low levels of enantioselectivity prompted us to use a more bulky silyl protecting group than TBS. The absolute stereochemistry of SI-8 was assumed based on the Sharpless mnemonic[9].

^{1}H NMR (400 MHz, $CDCl_3$) δ 3.96 (s, 1H, C12O*H*), 3.95 – 3.83 (m, 2H, C1*H$_2$*), 3.58 (ddd, *J* = 10.4, 3.6, 2.2 Hz, 1H, C11*H*), 2.96 (dd, *J* = 3.5, 1.3 Hz, 1H, C11O*H*), 2.48 (ddd, *J* = 16.9, 7.5, 5.3 Hz, 1H, C9*H$_a$*H$_b$), 2.36 (dt, *J* = 16.9, 7.8 Hz, 1H, C9H$_a$*H$_b$*), 1.80 (ddd, *J* = 14.7, 7.5, 4.2 Hz, 1H, C13*H$_a$*H$_b$), 1.74 – 1.69 (m, 1H, C13H$_a$*H$_b$*), 1.69 – 1.51 (m, 2H, C10*H$_2$*), 1.15 (s, 3H, C14*H$_3$*), 0.90 (s, 9H, C*Me$_3$*), 0.14 (s, 9H, Si*Me$_3$*), 0.10 (s, 6H, Si*Me$_2$*).
^{13}C NMR (101 MHz, $CDCl_3$) δ 107.6 (C8), 84.7 (C15), 75.7 (C11), 74.8 (C12), 60.4 (C1), 39.6 (C13), 30.5 (C10), 25.9 (C*Me$_3$*), 21.8 (C14), 18.2 (*C*Me$_3$), 17.3 (C9), 0.3 (Si*Me$_3$*), -5.4 (Si*Me$_a$*Me$_b$), -5.5 (SiMe$_a$*Me$_b$*).
HRMS (ESI$^-$) calculated for $C_{18}H_{37}O_3Si_2$ [*M-H*]$^-$ 357.2287, found 357.2290 (+0.84 ppm).
Physical state: colourless oil.

Preparation of (±)-**SI-9**: Swern oxidation of (±)-**SI-8**

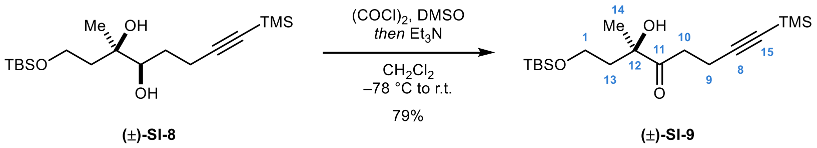

(±)-**SI-9** was synthesised analogously to (+)-**18** (*vide infra*) from (±)-**SI-8** and isolated in 79% yield (38 mg, 0.107 mmol).

Note: ^{1}H NMR, ^{13}C NMR, and TLC data matches that of **SI-9**.

HRMS (ESI$^+$) calculated for $C_{18}H_{36}O_3Si_2Na$ $[M+Na]^+$ 379.2095, found 379.2081 (–3.74 ppm).
Physical state: yellow oil.

Preparation of **SI-9**: Swern oxidation of **SI-8**

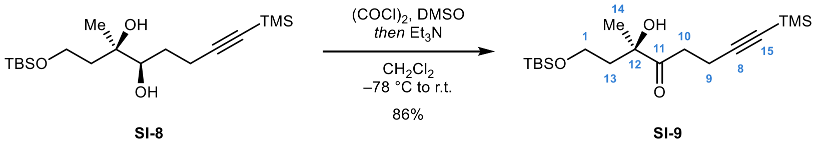

SI-9 was synthesised analogously to (+)-**18** (*vide infra*) from **SI-8** and isolated in 86% yield (153 mg, 0.429 mmol).

^{1}**H NMR** (400 MHz, $CDCl_3$) δ 4.55 (s, 1H, C12O*H*), 3.80 (ddd, *J* = 10.4, 7.6, 3.8 Hz, 1H, C1H_aH$_b$), 3.67 (ddd, *J* = 10.5, 6.5, 4.0 Hz, 1H, C1H$_a$$H_b$), 3.00 (ddd, *J* = 18.4, 8.1, 6.5 Hz, 1H, C10H_aH$_b$), 2.87 (ddd, *J* = 18.5, 8.4, 6.7 Hz, 1H, C10H$_a$$H_b$), 2.60 – 2.38 (m, 2H, C9H_2), 2.11 (ddd, *J* = 14.4, 7.5, 4.0 Hz, 1H, C13H_aH$_b$), 1.83 (ddd, *J* = 14.5, 6.6, 3.8 Hz, 1H, C13H$_a$$H_b$), 1.31 (s, 3H, C14$H_3$), 0.87 (s, 9H, C$Me_3$), 0.12 (s, 9H, Si$Me_3$), 0.04 (s, 3H, Si$Me_aMe_b$), 0.03 (s, 3H, SiMe$_a$$Me_b$).
^{13}**C NMR** (101 MHz, $CDCl_3$) δ 213.5 (C11), 106.1 (C8), 84.9 (C15), 78.7 (C12), 60.0 (C1), 40.4 (C13), 36.4 (C10), 26.6 (C14), 26.0 (CMe_3), 18.3 (*C*Me$_3$), 14.4 (C9), 0.2 (SiMe_3), -5.6 (SiMe_aMe$_b$), -5.6 (SiMe$_a$$Me_b$).
HRMS (ESI$^+$) calculated for $C_{18}H_{37}O_3Si_2$ $[M+H]^+$ 357.2276, found 357.2275 (–0.21 ppm).
TLC (10% EtOAc in hexanes): R_f = 0.28.
Physical state: yellow oil.
Enantiomeric ratio (GC-FID) 60:40, ChiraSil® DEX CB 25 m × 0.25 mm, injector temperature: 250 °C, 1 ml/min, 120 °C for 200 min, heat to 130 °C over 60 min, hold at 130 °C for 200 min, heat to 185 °C over 60 min then hold for 5 min.

Preparation of protected triol (±)-**17**: dihydroxylation of 1,5-enyne **16**

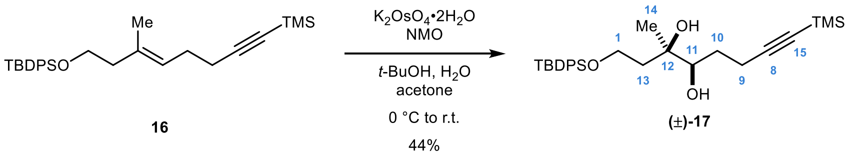

Following a modified literature procedure[10], NMO (5.0 eq., 5.00 mmol, 586 mg) and K_2OsO_4•$2H_2O$ (0.03 eq., 0.03 mmol, 11 mg) were dissolved in H_2O (1.5 ml) and *t*-BuOH (1.5 ml) under air then cooled to 0 °C. 1,5-Enyne **16** (1.0 eq., 1.00 mmol, 449 mg) was dissolved in acetone (1.5 ml) and added to the above biphasic reaction mixture at 0 °C. The reaction mixture was allowed to warm to r.t. overnight. Upon completion determined by TLC, Na_2SO_3 (1.5 g), EtOAc (10 ml) and water (10 ml) were added and the mixture was stirred at room temperature for 30 min. The phases were separated, the aqueous phase was washed with EtOAc (2 × 10 ml) and the combined organic layers were dried over $MgSO_4$, filtered and concentrated *in vacuo*. The crude product was purified by column chromatography on silica gel (17% EtOAc in Hexanes) to give the title compound (211 mg, 0.437 mmol, 44%) as a pale-yellow oil.

Note: ^{1}H NMR, ^{13}C NMR, and TLC data matches that of (+)-**17**.

HRMS (ES^+) calculated for $C_{28}H_{43}O_3Si_2$ $[M+H]^+$ 483.2745, found 483.2743 (–0.4 ppm).
Physical state: pale-yellow oil.

Preparation of protected triol (+)-**17**: Sharpless asymmetric dihydroxylation of 1,5-enyne **16**

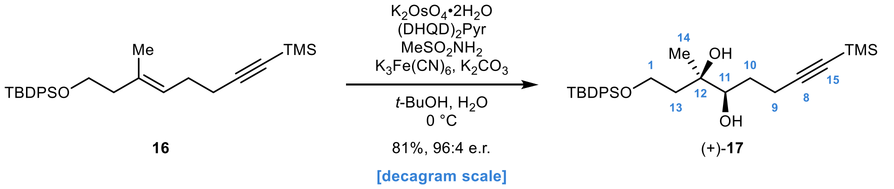

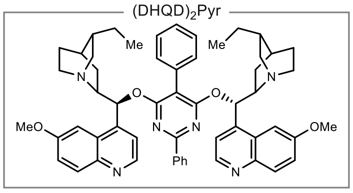

Following the general recommendations outlined by Sharpless *et al.*[9], a flask was charged with H_2O (280 ml) and *t*-BuOH (280 ml) under air. Then, $K_3Fe(CN)_6$ (3.0 eq., 180 mmol, 59.3 g), K_2CO_3 (3.0 eq., 180 mmol, 24.9 g), $(DHQD)_2PYR$ (0.025 eq., 1.50 mmol, 1.32 g), K_2OsO_4•$2H_2O$ (0.01 eq., 0.60 mmol, 221 mg) and $MeSO_2NH_2$ (1.0 eq., 60.0 mmol, 5.71 g) were added sequentially, making sure that the previous reagent was fully dissolved before adding the next. The resulting biphasic mixture was stirred at r.t. for 15 min, then cooled to 0 °C. ***Note****: A large amount of inorganic salts precipitate which makes stirring difficult, however efficient stirring is vital to achieve high enantioselectivity*. 1,5-Eneyne **16** (1.0 eq., 60.0 mmol, 26.9 g) was dissolved in a mixture of H_2O (10 ml) and *t*-BuOH (10 ml) under air then added to the above solution. The flask previously containing 1,5-eneyne **16** was rinsed with H_2O (10 ml) and *t*-BuOH (10 ml) and added to the reaction mixture. The resulting mixture was stirred vigorously at 0 °C for 18 h, then Na_2SO_3 (90 g) was added then the mixture was warmed to r.t. and stirred for 30 min. Next, EtOAc (200 ml) and water (100 ml) were added, the phases were separated, the aqueous phase was washed with EtOAc (4 × 150 ml) and the combined organic layers were dried over $MgSO_4$, filtered and concentrated *in vacuo*. The crude product was purified by column chromatography on silica gel (7% to 17% to 27% EtOAc in petroleum ether 40-60) to give the title compound (23.5 g, 48.6 mmol, 81%, 96:4 e. r.) as a yellow oil.

Note: the temperature of the reaction mixture was measured internally to ensure high enantioselectivity, see picture below.

^{1}H NMR (400 MHz, $CDCl_3$) δ 7.67 (ddd, *J* = 7.7, 3.5, 1.5 Hz, 4H, Ar*H*), 7.50 – 7.37 (m, 6H, Ar*H*), 3.99 (s, 1H, C12O*H*), 3.94 (ddd, *J* = 11.7, 8.2, 3.7 Hz, 1H, C1H_aH_b), 3.86 (ddd, *J* = 10.8, 6.3, 4.1 Hz, 1H, C1H_aH_b), 3.59 (ddd, *J* = 10.2, 3.7, 2.4 Hz, 1H, C11*H*), 2.91 (dd, *J* = 3.8, 1.1 Hz, 1H, C11O*H*), 2.49 (ddd, *J* = 17.0, 7.8, 5.4 Hz, 1H, C9H_aH_b), 2.41 – 2.27 (m, 1H, C9H_aH_b), 1.86 (ddd, *J* = 14.7, 8.2, 4.1 Hz, 1H, C13H_aH_b), 1.73 – 1.66 (m, 1H, C13H_aH_b), 1.66 – 1.52 (m, 2H, C10H_2), 1.18 (s, 3H, C14H_3), 1.06 (s, 9H, CMe_3), 0.13 (s, 9H, SiMe_3).
^{13}C NMR (101 MHz, $CDCl_3$) δ 135.7 (ArCH), 132.7 (Ar*C*), 132.6 (Ar*C*), 130.2 (ArCH), 128.0 (Ar*C*H), 107.5 (C8), 84.8 (C15), 76.2 (C11), 74.9 (C12), 61.5 (C1), 39.4 (C13), 30.6 (C10), 26.9 (CMe_3), 21.7 (C14), 19.1 (*C*Me_3), 17.4 (C9), 0.3 (SiMe_3).
HRMS (ES^+) calculated for $C_{28}H_{42}O_3Si_2Na$ $[M+Na]^+$ 505.2565, found 505.2545 (–3.95 ppm).
TLC (17% EtOAc in hexanes): R_f = 0.30.
Physical state: yellow oil.
Enantiomeric ratio (HPLC-UV) 96:4, Chiralpak IB column, hexanes:*i*-PrOH 99.5:0.5 (isocratic), 1 ml/min, 22 °C, 220 or 210 nm, compared to authentic racemic material (±)-**17**.
$[\alpha]_D^{26}$ = +26.7° (c = 0.19 g/100 ml, MeOH)

Chiral HPLC trace of (±)-**17**

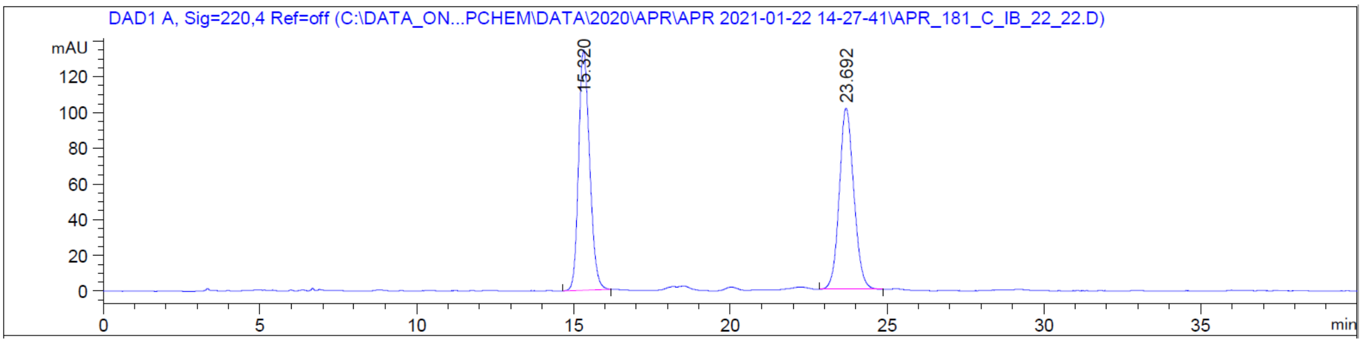

Signal 1: DAD1 A, Sig=220,4 Ref=off

Peak #	RetTime [min]	Type	Width [min]	Area [mAU*s]	Height [mAU]	Area %
1	15.320	BB	0.3803	3298.46045	134.43262	50.0500
2	23.692	BB	0.4918	3291.86475	101.65165	49.9500

Chiral HPLC trace of (+)-**17**

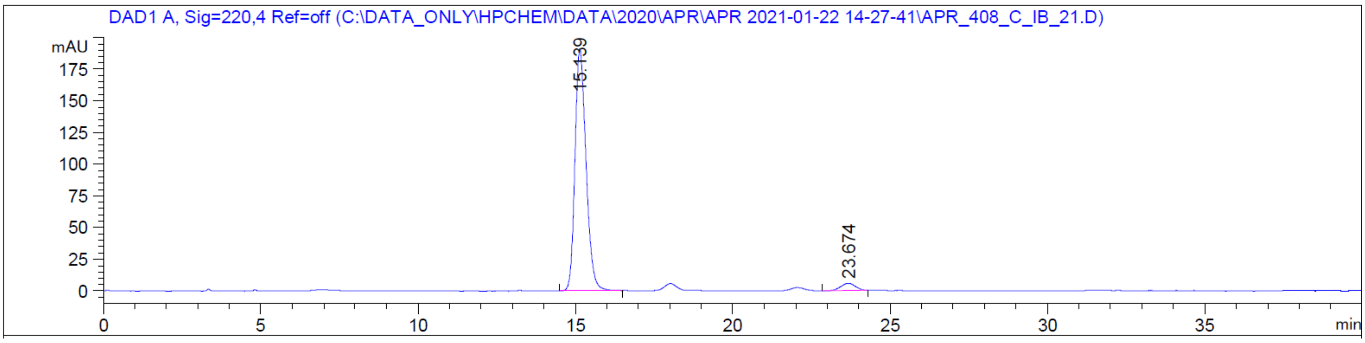

Signal 1: DAD1 A, Sig=220,4 Ref=off

Peak #	RetTime [min]	Type	Width [min]	Area [mAU*s]	Height [mAU]	Area %
1	15.139	BB	0.3769	4655.75098	190.67508	96.1918
2	23.674	BB	0.3931	184.32088	5.70259	3.8082

Table S1. Optimisation of the Sharpless asymmetric dihydroxylation[9].

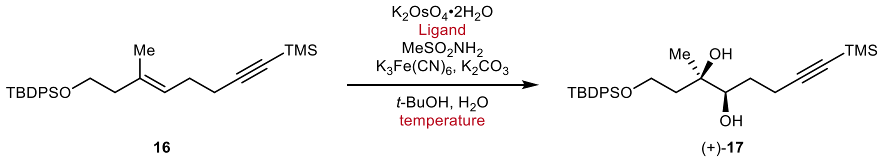

Entry	Ligand	Temperature	yield	e.r.
1	AD-mix-β	r.t.	72%	73:27
2	AD-mix-β	10 °C	n.d. (full conversion)	80:20
3	AD-mix-β	0 °C	75%	85:15
4	$(DHQD)_2$PHAL	0 °C	n.d. (low conversion)	85:15
5	$(DHQD)_2$AQN	0 °C	72%	90:10
6	$(DHQD)_2$PYR	0 °C	67%	96:4

The reactions were carried out on 1 mmol scale. n.d. – not determined. Yields refer to isolated yields.

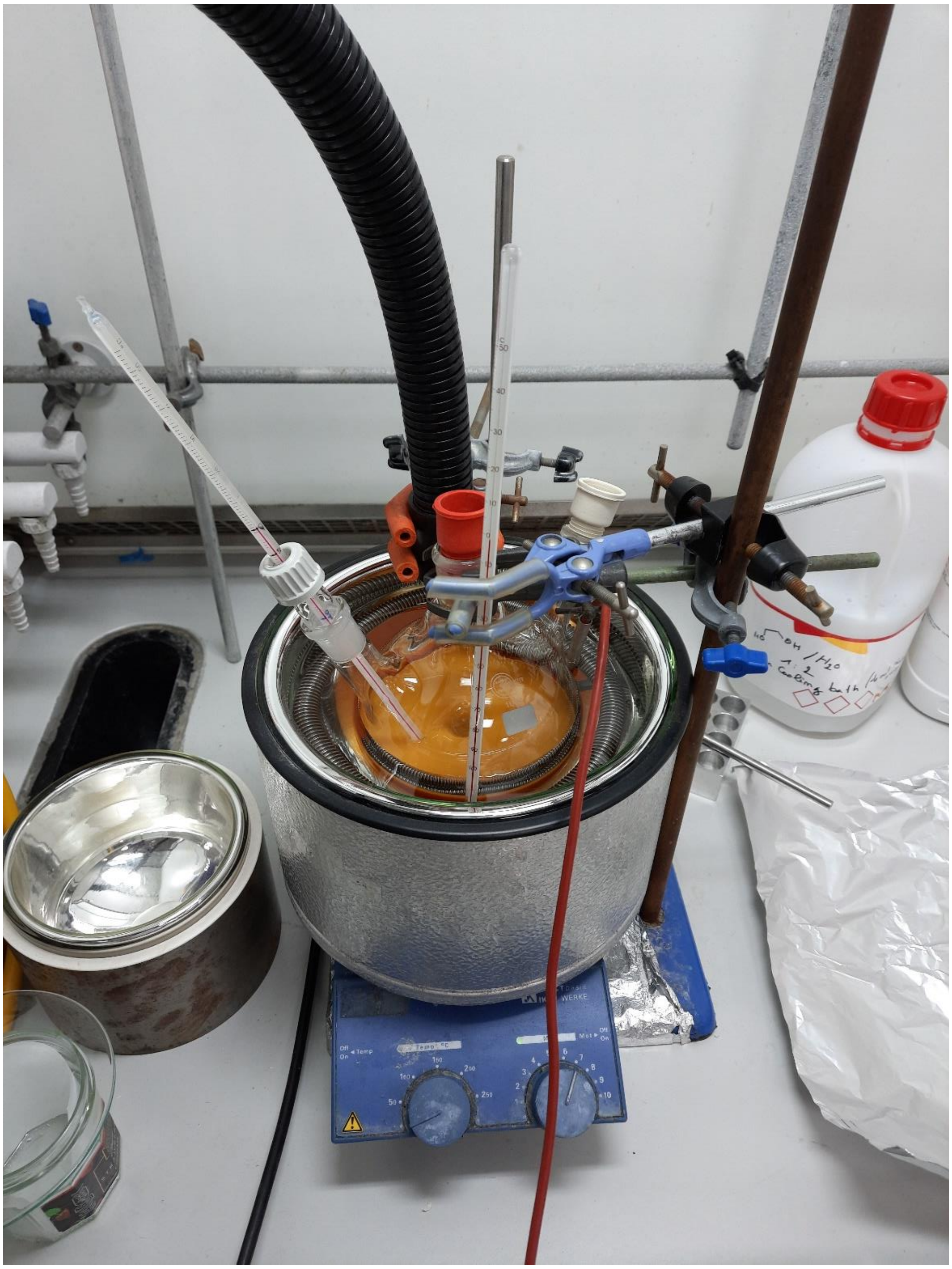

Figure S2. Setup for the decagram scale Sharpless asymmetric dihydroxylation of **16**.

Preparation of dihydroxyketone (+)-**18**: Swern oxidation of protected triol (+)-**17**

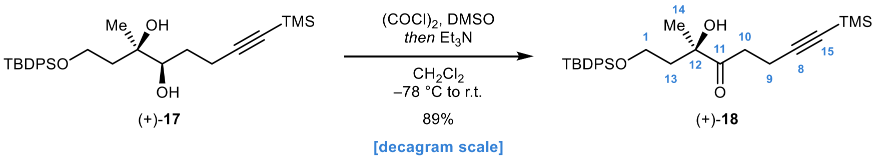

An oven-dried, 2 litre round bottom flask was equipped with a stirrer bar, then the flask was evacuated and refilled with dry N_2 three times. $(COCl)_2$ (2.0 eq., 89.1 mmol, 11.3 g, 7.54 ml) and dry CH_2Cl_2 (300 ml) were added to the flask then the solution was cooled to –78 °C. In a separate flask, DMSO (3.0 eq., 133 mmol, 10.4 g, 9.49 ml) was dissolved in dry CH_2Cl_2 (270 ml) under dry N_2, cooled to –78 °C, then added dropwise to the above-mentioned solution *via* cannula. The resulting solution was stirred for 15 min at –78 °C. In the meantime, (+)-**17** (1.0 eq., 44.6 mmol, 21.5 g) was dissolved in dry CH_2Cl_2 (165 ml) under dry N_2, cooled to –78 °C, then added dropwise to the above-mentioned solution *via* cannula. After stirring the resulting reaction mixture at –78 °C for 2 h, Et_3N (5.0 eq., 222 mmol, 22.5 g, 31.1 ml) was added dropwise. The reaction was stirred at –78 °C for another hour, then at 0 °C for 30 min, then at r.t. for 30 min. The reaction was quenched by the slow addition of water (450 ml), then the phases were separated, the aqueous phase was washed with EtOAc (3 × 100 ml) and the combined organic layers were dried over $MgSO_4$, filtered and concentrated *in vacuo*. The crude product was purified by column chromatography on silica gel (7% EtOAc in petroleum ether 40-60) to give the title compound (19.1 g, 39.7 mmol, 89%) as a yellow oil.

Note: To remove odorous by-products (undetectable by ^{1}H NMR) the pure product was dissolved in pentanes (150 ml) then concentrated *in vacuo* three times. Optimisation studies were carried out using the TBS analogue of (+)-**17**, **SI-8** (Table S2, *vide infra*).

^{1}H NMR (400 MHz, $CDCl_3$) δ 7.62 (ddd, *J* = 8.0, 5.0, 1.7 Hz, 4H, Ar*H*), 7.47 – 7.36 (m, 6H, Ar*H*), 4.49 (s, 1H, C12O*H*), 3.80 (ddd, *J* = 11.3, 7.3, 4.4 Hz, 1H, C1H_aH$_b$), 3.70 (ddd, *J* = 10.9, 6.6, 4.4 Hz, 1H, C1H$_a$$H_b$), 3.06 – 2.80 (m, 2H, C10H_2), 2.56 – 2.36 (m, 2H, C9H_2), 2.11 (ddd, *J* = 14.5, 7.3, 4.5 Hz, 1H, C13H_aH$_b$), 1.88 (ddd, *J* = 14.5, 6.6, 4.4 Hz, 1H, C13H$_a$$H_b$), 1.33 (s, 3H, C14$H_3$), 1.04 (s, 9H, C*Me*$_3$), 0.13 (s, 9H, Si*Me*$_3$).
^{13}C NMR (101 MHz, $CDCl_3$) δ 213.3 (C11), 135.7 (Ar*C*H), 135.6 (Ar*C*H), 132.9 (Ar*C*), 132.8 (Ar*C*), 130.1 (Ar*C*H), 128.0 (Ar*C*H), 106.0 (C8), 85.1 (C15), 78.8 (C12), 61.0 (C1), 40.4 (C13), 36.4 (C10), 27.0 (C*Me*$_3$), 26.5 (C14), 19.2 (*C*Me$_3$), 14.5 (C9), 0.2 (Si*Me*$_3$).
HRMS (ES$^+$) calculated for $C_{28}H_{40}O_3Si_2Na$ $[M+Na]^+$ 503.2408, found 503.2393 (–2.98 ppm).
TLC (7% EtOAc in hexanes): R_f = 0.27.
Physical state: yellow oil.
$[\alpha]_D^{26}$ = +0.8° (c = 0.22 g/100 ml, MeOH)

Table S2. Optimisation of the oxidation of the C11 secondary alcohol moiety of **SI-8**.

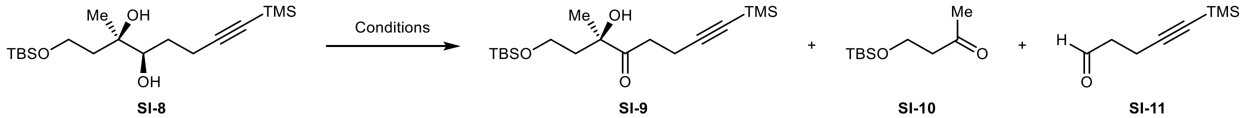

Entry	Conditions	Outcome
1[11]	DMP, $NaHCO_3$, CH_2Cl_2, 0 °C to r.t.	full conversion but low yield, 1:1 mix of **SI-9** and **SI-10**.
2[12]	TPAP, NMO, mol. sieves, CH_2Cl_2, r.t.	full conversion, **SI-10** is the major product (5:1).
3[13,14]	SO_3•py, Et_3N, DMSO, CH_2Cl_2, 0 °C to 35 °C	45% **SI-8**, 25% **SI-9**, 0% **SI-10** after 30 h.
4[15]	[Al] catalyst, pivalaldehyde, toluene, r.t.	traces of **SI-9** after 50 h (TLC).
5[16]	IBX, DMSO, r.t.	traces of **SI-9** after 50 h (TLC).
6	$(COCl)_2$, DMSO, Et_3N, CH_2Cl_2, –78 °C to r.t.	full conversion, clean, 83% (79%) **SI-9**, 0% **SI-10**.

SI-9 and **SI-10** are inseparable by column chromatography, whereas **SI-11** is separable but not isolated. Yields determined by ^{1}H NMR analysis of crude reaction mixtures, using $MeNO_2$ as internal standard, isolated yields are shown in parentheses.

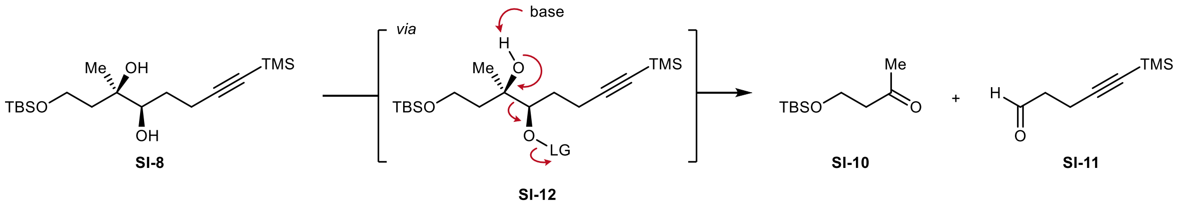

Figure S3. Proposed mechanism for the formation of **SI-10** and **SI-11** from **SI-8**. If a base is present before the tertiary alcohol can be 'capped', cleavage of the 1,2-diol moiety becomes the favoured pathway; a solution is to use superstoichiometric amounts of chloro(dimethyl)sulfonium chloride under standard Swern conditions. LG denotes an unspecified leaving group.

Preparation of vinyl diol (–)-**19**: diastereoselective, chelate-controlled Grignard addition to (+)-**18**

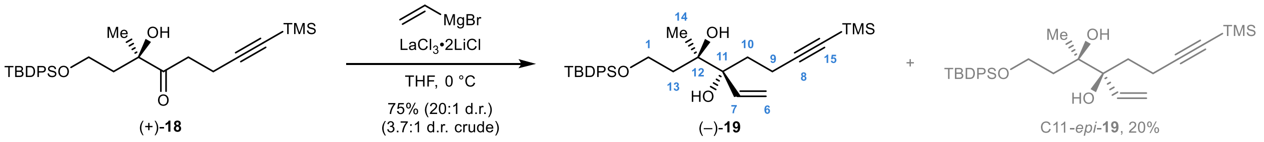

Dihydroxyketone (+)-**18** (1.0 eq., 49.5 mmol, 23.8 g) was dissolved in dry THF (495 ml) under dry N_2, then $LaCl_3$•2LiCl solution (2.5 eq., 124 mmol, 206 ml, 0.6 M in THF) was added at r.t.. The reaction mixture was stirred at r.t. for 1 h before cooling to 0 °C, then vinylmagnesium bromide solution (5.0 eq., 247 mmol, 248 ml, 1 M in THF) was added dropwise *via* cannula. The reaction mixture was stirred for 40 min at 0 °C, then water (500 ml) and EtOAc (500 ml) were added. The pH was set to 1 with 1 M HCl to dissolve the inorganic by-products. The phases were separated, the aqueous phase was washed with EtOAc (3 × 250 ml) and the combined organic layers were dried over $MgSO_4$, filtered and concentrated *in vacuo*. (An aliquot was taken to determine the diastereoselectivity of the reaction by ^{1}H NMR analysis, and showed a 3.7:1 ratio of diastereomers in favour of the desired isomer.) The crude product was first purified by column chromatography on silica gel (20% Et_2O in petroleum ether 40-60), then the mixed fractions of the two diastereomers were combined and separated by DCVC (d = 10 cm, l = 6.5 cm, V = 250 ml, 0% (3 times) to 26% Et_2O in petroleum ether 40-60 with 2% increments) to give the title compound (18.9 g, 37.1 mmol, 75%) as a yellow oil.

Note: It is important to quench the reaction after 40 min to ensure high yield.

^{1}H NMR (500 MHz, $CDCl_3$) δ 7.68 (ddd, J = 7.9, 3.8, 1.6 Hz, 4H, Ar*H*), 7.49 – 7.38 (m, 6H, Ar*H*), 5.81 (dd, J = 17.3, 10.9 Hz, 1H, C7*H*), 5.35 (dd, J = 17.3, 1.8 Hz, 1H, C6$H_{trans}H_{cis}$), 5.26 (dd, J = 10.9, 1.7 Hz, 1H, $H_{trans}H_{cis}$), 4.04 (s, 1H, C12O*H*), 4.03 – 3.94 (m, 1H, C1H_aH_b), 3.85 (dt, J = 10.8, 4.3 Hz, 1H, C1H_aH_b), 2.91 (s, 1H, C11O*H*), 2.34 (ddd, J = 16.9, 10.6, 6.0 Hz, 1H, C9H_aH_b), 2.24 – 2.15 (m, 1H, C9H_aH_b), 2.11 (ddd, J = 15.2, 10.8, 4.8 Hz, 1H, C13H_aH_b), 2.00 (ddd, J = 13.5, 10.6, 6.0 Hz, 1H, C10H_aH_b), 1.77 (ddd, J = 14.1, 10.8, 4.6 Hz, 1H, C10H_aH_b), 1.49 (dt, J = 14.6, 3.5 Hz, 1H, C13H_aH_b), 1.25 (s, 3H, C14H_3), 1.05 (s, 9H, C*Me*$_3$), 0.15 (s, 9H, Si*Me*$_3$).

^{13}C NMR (101 MHz, $CDCl_3$) δ 138.6 (C7), 135.7 (Ar*C*H), 132.7 (Ar*C*), 132.6 (Ar*C*), 130.1 (Ar*C*H), 128.0 (Ar*C*H), 116.0 (C6), 108.4 (C8), 84.6 (C15), 79.4 (C11), 76.9 (C12), 61.7 (C1), 36.6 (C13), 33.0 (C10), 26.9 (C*Me*$_3$), 21.9 (C14), 19.1 (*C*Me$_3$), 14.7 (C9), 0.3 (Si*Me*$_3$).

HRMS (APCI$^+$) calculated for $C_{30}H_{45}O_3Si_2$ $[M+H]^+$ 509.2902, found 509.2904 (0.44 ppm).

TLC (20% EtOAc in hexanes): R_f = 0.29.

Physical state: yellow oil.

$[\alpha]_D^{26}$ = –1.5° (c = 0.44 g/100 ml, MeOH)

Table S3. Initial optimisation of the chelate-controlled Grignard addition to **SI-9**.

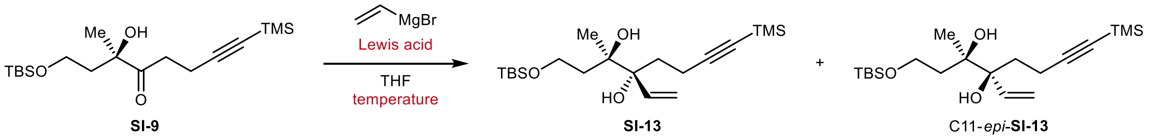

Entry	Lewis acid	Temperature	Reagent order	**SI-9**	**SI-13**	d.r.
1	–	0 °C	SM + G (+ G)	low conversion	n.d.	n/a
2[17]	$ZnCl_2$	r.t.	LA + G + SM (+ G)	n.d.	0%	n/a
3	LiCl	0 °C	LA + G + SM (+ G)	low conversion	n.d.	n/a
4[18]	$CeCl_3$	–78 °C	LA + G + SM	n.d.	(26%)	>20:1

LA refers to Lewis acid, G to vinylmagnesium bromide, SM to starting material (i.e. **SI-9**). Yields and d.r. were determined by ^1H NMR analysis of crude reaction mixtures, using $MeNO_2$ as internal standard, isolated yields are shown in parentheses. n.d. – not determined.

Table S4. Optimisation of the chelate-controlled Grignard addition to (+)-**18** – Lewis acid screening.

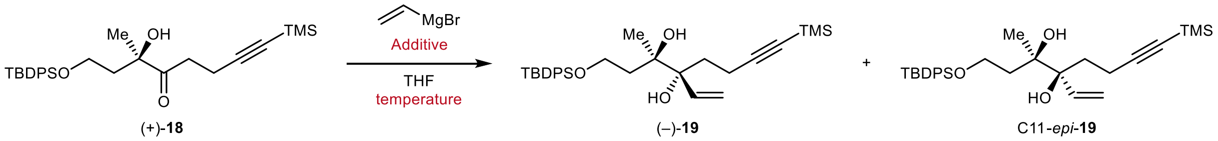

Entry	Additive	Temperature	Reagent order	**18**	**19**	d.r.
1[18]	$CeCl_3$	–78 °C	LA + G + SM	n.d.	(43%)	>20:1
2	NaH, $CeCl_3$	–78 °C	SM + A + LA + G	n.d.	0%	n/a
3	NaH, $Mg(OTf)_2$	0 °C	SM + A + LA + G	n.d.	0%	n/a
4	NaH, $Yb(OTf)_3$	0 °C	SM + A + LA + G	traces	0%	n/a
5	NaH, $ZnCl_2$	0 °C	SM + A + LA + G	26%	0%	n/a
6[19]	$CeCl_3$•2LiCl	–78 °C	SM + LA + G	50%	23%	3.8:1
7	dioxane	0 °C	G + A + SM	43%	33%	1.7:1
8[20]	TBAB, DME	0 °C	A + G + SM	46%	31%	2.6:1
9[20]	TBAB, dioxane	0 °C	A + G + SM	52%	19%	1.7:1
10[20]	diglyme	0 °C	G + A + SM	24%	29%	2.1:1
11[20]	NBu_4Cl	0 °C	G + A + SM	62%	14%	3.5:1
12[20]	NBu_4Cl, diglyme	0 °C	G + A + SM	78%	traces	n/a
13	$La(OTf)_3$	–78 °C	LA + SM + G	*messy*	0%	n/a
14	$Sm(OTf)_3$	–78 °C	LA + SM + G	*messy*	0%	n/a
15	$La(OTf)_3$	–78 °C	LA + G + SM	n.d.	– (TLC)	n/a
16	$Sm(OTf)_3$	–78 °C	LA + G + SM	n.d.	– (TLC)	n/a
17	$Yb(OTf)_3$	–78 °C	LA + G + SM	n.d.	– (TLC)	n/a
18	$CeCl_3$	–78 °C	LA + G + SM	49%	26%	5.2:1
19[19]	$CeCl_3$•2LiCl	–78 °C	LA + G + SM	69%	5%	10:1
20[19]	$LaCl_3$•2LiCl	0 °C	LA + SM + G	10%	53%	4.1:1
21	–	–78 °C	SM + G	38%	30%	2.5:1
22	sat. LiCl	–78 °C	SM + A + G	92%	– (TLC)	n/a

LA refers to Lewis acid, G to vinylmagnesium bromide, SM to starting material (i.e. (+)-**18**) and A to non-Lewis acid additives. Yields and d.r. were determined by ^1H NMR analysis of crude reaction mixtures, using $MeNO_2$ as internal standard, isolated yields are shown in parentheses. n.d. – not determined.

Table S5. Optimisation of the chelate-controlled Grignard addition to (+)-**18** using $LaCl_3$•2LiCl[19].

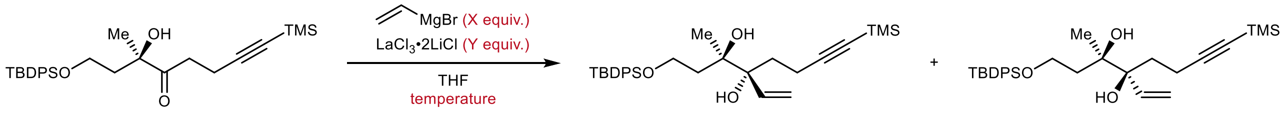

Entry	X	Y	Temperature	Reagent order	**18**	**19**	d.r.
1	1.05	1.05	r.t.	LA + SM + G	65%	11%	2.2:1
2	2.5	1.05	r.t.	LA + SM + G	traces	50%	2.6:1
3	5.0	1.05	r.t.	LA + SM + G	traces	47%	2.0:1
4	3.0	1.5	r.t.	LA + SM + G	traces	56%	3.1:1
5	5.0	1.5	r.t.	LA + SM + G	–	*messy*	n.d.
6	2.5	2.5	r.t.	LA + SM + G	–	54%	3.4:1
7	5.0	1.5	0 °C	LA + SM + G	–	59%	2.7:1
8	2.5	2.5	0 °C	LA + SM + G	10%	53%	4.1:1
9	2.5	2.5	0 °C	LA + G + SM	46%	5%	n.d.
10	5.0	2.5	0 °C	LA + SM + G	traces	65%	3.1:1
11	2.5	5.0	0 °C	LA + SM + G	22%	48%	3.0:1
12	5.0	5.0	0 °C	LA + SM + G	–	70%	2.6:1
13	10	10	0 °C	LA + SM + G	–	*messy*	n.d.
14	2.5	1.05	–78 °C	LA + SM + G	13%	46%	3.3:1
15	5.0	1.05	–78 °C	LA + SM + G	9%	51%	3.9:1
16	2.5	1.5	–78 °C	LA + SM + G	8%	49%	3.3:1
17	5.0	1.5	–78 °C	LA + SM + G	4%	54%	3.4:1
18	1.5	1.5	–78 °C	LA + SM + G	66%	11%	1.6:1
19	2.0	2.0	–78 °C	LA + SM + G	31%	37%	2.5:1
20	5.0	2.5	–78 °C	LA + SM + G	4%	60%	3.0:1
21	3.0	3.0	–78 °C	LA + SM + G	4%	61%	2.7:1
22	2.5	5.0	–78 °C	LA + SM + G	21%	46%	2.6:1
23	2.5	2.5	–78 °C	LA + SM + G	5%	62%	2.7:1
24	2.5	2.5	–78 °C	LA + G + SM	15%	54%	2.6:1
25[a]	5.0	2.5	0 °C	LA + SM + G	–	67%	3.0:1
26[b]	5.0	2.5	0 °C	LA + SM + G	–	65%	2.5:1
27[c]	5.0	2.5	0 °C	LA + SM + G	–	68%	2.1:1
28[d]	5.0	2.5	0 °C	LA + SM + G	–	64%	3.2:1

LA refers to Lewis acid, G to vinylmagnesium bromide, and SM to starting material (i.e. (+)-**18**). Yields and d.r. were determined by ^{1}H NMR analysis of crude reaction mixtures, using $MeNO_2$ as internal standard. n.d. – not determined. $LaCl_3$•2LiCl was used as a pre-made solution in THF (0.6 M). [a]Run in toluene, [b]run in Et_2O, [c]run in hexanes, [d]run in 1,4-dioxane.

Preparation of triol (–)-**11**: deprotection of (–)-**19**

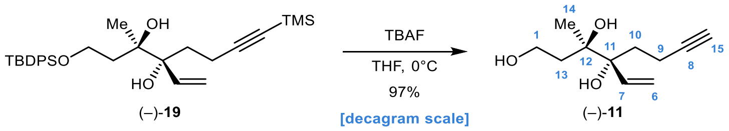

Protected triol (–)-**19** (1.0 eq., 26.7 mmol, 13.6 g) was dissolved in dry THF (265 ml) under dry N_2, then cooled to 0 °C. TBAF solution (2.1 eq., 56.0 mmol, 56 ml, 1 M in THF) was added dropwise, then the reaction was stirred at 0 °C for 2 h. To quench the reaction, saturated aq. NH_4Cl solution (250 ml), water (200 ml) and Et_2O (200 ml) were added. The phases were separated, the aqueous phase was washed with Et_2O (8 × 100 ml) and the combined organic layers were dried over $MgSO_4$, filtered and concentrated *in vacuo*. The crude product was purified by DCVC (d = 6.5 cm, l = 6 cm, V = 100 ml, 0% to 7% MeOH in CH_2Cl_2 with 0.5% increments) to give the title compound (5.12 g, 25.8 mmol, 97%) as a white solid.

^{1}H NMR (500 MHz, $CDCl_3$) δ 5.82 (dd, J = 17.2, 10.8 Hz, 1H, C7*H*), 5.38 (dt, J = 17.3, 1.6 Hz, 1H, C6$H_{trans}H_{cis}$), 5.32 (dd, J = 10.8, 1.6 Hz, 1H, C6$H_{trans}H_{cis}$), 3.99 (td, J = 10.8, 2.8 Hz, 1H, C1H_aH_b), 3.83 (dd, J = 10.1, 5.1 Hz, 1H, C1H_aH_b), 2.92 (s, 1H, C12O*H*), 2.88 (s, 1H, C11O*H*), 2.73 (s, 1H, C1O*H*), 2.35 – 2.22 (m, 1H, C9H_aH_b), 2.20 – 2.13 (m, 1H, C9H_aH_b), 2.10 (m, 1H, C13H_aH_b), 2.03 (ddd, J = 13.7, 9.5, 6.8 Hz, 1H, C10H_aH_b), 1.97 (app q, J = 2.2 Hz, 1H, C15*H*), 1.78 (ddd, J = 14.1, 9.7, 4.8 Hz, 1H, C10H_aH_b), 1.55 (dt, J = 14.7, 3.7 Hz, 1H, C13H_aH_b), 1.25 (s, 3H, C14H_3).

^{13}C NMR (126 MHz, $CDCl_3$) δ 138.5 (C7), 116.7 (C6), 85.2 (C8), 79.8 (C11), 77.6 (C12), 68.8 (C15), 59.7 (C1), 36.7 (C13), 32.8 (C10), 22.2 (C14), 13.3 (C9).

HRMS (ES^+) calculated for $C_{11}H_{18}O_3Na$ $[M+Na]^+$ 221.1148, found 221.1139 (–4.07 ppm).

TLC (5% MeOH in CH_2Cl_2): R_f = 0.32.

Physical state: white solid.

mp: 68–70 °C

$[\alpha]_D^{26}$ = –0.1° (c = 0.18 g/100 ml, MeOH)

XRD: Single crystal analysis confirms the structure drawn and the relative and absolute stereochemistry.

Preparation of lactone (+)-**20**: oxidative lactonisation of triol (–)-**11**

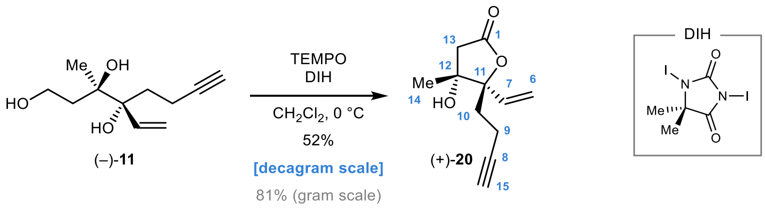

Triol (–)-**11** (1.0 eq., 7.50 mmol, 1.49 g) and TEMPO (0.4 eq., 3.0 mmol, 469 mg) were dissolved in CH_2Cl_2 (75 ml) under nitrogen then the solution was cooled to 0 °C. 1,3-Diiodo-5,5-dimethylhydantoin (DIH, 2.2 eq., 16.5 mmol, 6.27 g) was added in a single portion then the reaction mixture was stirred at 0 °C for 20 min. The reaction was quenched with saturated aq. Na_2SO_3 (70 ml) and EtOAc (75 ml), the phases were separated, the aqueous phase was washed with EtOAc (3 × 50 ml) and the combined organic layers were dried over $MgSO_4$, filtered and concentrated *in vacuo*. The crude product was purified by column chromatography on silica gel (70% Et_2O in hexanes) to give the title compound (1.19 g, 6.10 mmol, 81%) as a yellow oil.

When the reaction was repeated using 52 mmol (10.3 g) of triol (–)-**11**, lactone (+)-**20** was isolated in 52% yield after DCVC purification (d = 6.5 cm, l = 6 cm, V = 100 ml, 0% to 50% Et_2O in Hexanes with 5% increments, then from 50% to 70% Et_2O in Hexanes with 2.5% increments; after this, the column was washed with 75% Et_2O in Hexanes (three times), and with 80% Et_2O in Hexanes (three times) to elute any product left on the column).

Note: It's imperative to stop the reaction after 20 min to ensure high yield.

^{1}H NMR (500 MHz, $CDCl_3$) δ 5.78 (dd, J = 17.4, 11.1 Hz, 1H, C7H), 5.59 (d, J = 17.9 Hz, 1H, C6$H_{trans}H_{cis}$), 5.55 (d, J = 11.2 Hz, 1H, C6$H_{trans}H_{cis}$), 2.67 (s, 2H, C13H_2), 2.34 (dddd, J = 16.7, 11.5, 5.1, 2.6 Hz, 1H, C9H_aH_b), 2.22 (dddd, J = 16.8, 11.2, 5.0, 2.7 Hz, 1H, C9H_aH_b), 2.02 – 1.96 (m, 1H, C10H_aH_b), 1.96 (app d, J = 2.7 Hz, 1H, C15H), 1.95 (s, 1H, C12OH), 1.87 (dddd, J = 13.9, 11.2, 7.9, 5.1 Hz, 1H, C10H_aH_b), 1.41 (s, 3H, C14H_3).
^{13}C NMR (126 MHz, $CDCl_3$) δ 173.7 (C1), 132.1 (C7), 120.5 (C6), 92.0 (C11), 83.5 (C8), 77.3 (C12), 69.0 (C15), 42.9 (C13), 34.8 (C10), 22.0 (C14), 13.6 (C9).
HRMS (APCI$^+$) calculated for $C_{11}H_{15}O_3$ $[M+H]^+$ 195.1016, found 195.1023 (+3.59 ppm).
TLC (70% Et_2O in Hexanes): R_f = 0.25.
Physical state: yellow oil.
$[\alpha]_D^{26}$ = +4.5° (c = 0.17 g/100 ml, MeOH)

Table S6. Optimisation of the oxidative lactonisation of (–)-**11**.

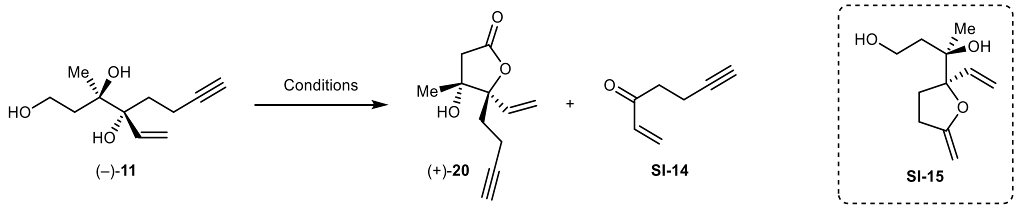

Entry	Conditions	Outcome
1	TPAP, NMO, (mol. sieves), CH_2Cl_2, r.t.	full conversion, messy, 18% **SI-14**, several side-products
2	PCC or PDC, (Celite®), CH_2Cl_2, r.t.	full conversion, messy, <10% **SI-14**, several side-products
3[21]	DMP, (pyridine), CH_2Cl_2, r.t.	full conversion, 20% **SI-14,** several side-products
4	TEMPO, PIDA, CH_2Cl_2, r.t.	>90% **SI-14**
5	$BaMnO_4$, CH_2Cl_2, r.t.	no reaction
6	SO_3•py, Et_3N, DMSO, CH_2Cl_2, 0 °C to r.t.	only **SI-14** (TLC)
7[22]	$AgCO_3$ on Celite® (Fétizon's reagent)	full conversion, clean, 85% **SI-15**
8	$(COCl)_2$, DMSO, Et_3N, CH_2Cl_2, –78 °C to r.t.	messy
9[23]	CuI, bpy, TEMPO, NMI, MeCN, r.t., air	very low conversion
10[24]	$Cu(MeCN)_4OTf$, bpy, NMI, TEMPO, MeCN, r.t., air	full conversion, clean, no (+)-**20**, no **SI-14**
11[25]	TEMPO, TCCA, CH_2Cl_2, 0 °C	messy
12	IBX, DMSO, r.t.	full conversion, clean, 86% of an unidentified product
13[26]	TEMPO, NaOCl, $NaHCO_3$, KBr, CH_2Cl_2, H_2O, 0 °C	full conversion, clean, no **SI-14**, 45% (41%) (+)-**20** – not reproducible
14	Al(O*i*-Pr)$_3$, cyclohexanone, toluene, reflux	full conversion, clean, 75% of an unidentified product
15[27]	$RuCl_3$•xH_2O, $NaBrO_3$, Aliquat 336, Na_2HPO_4, $CHCl_3$, H_2O, r.t.	full conversion, clean, 45% (+)-**20**
16[28]	TEMPO, $Ca(OCl)_2$, MeCN, r.t.	no reaction
17[29]	TEMPO, PhIO, $Yb(OTf)_3$•xH_2O, CH_2Cl_2, 0 °C	full conversion, only **SI-14** (TLC)
18[30]	TEMPO, DIH, CH_2Cl_2, 0 °C	full conversion, clean, 80% (+)-**20**
19[31]	TEMPO, Oxone®, TBAB, CH_2Cl_2, r.t.	full conversion, messy, no (+)-**20**
20	TEMPO, NIS, CH_2Cl_2, 0 °C	full conversion, clean, 63% (+)-**20**
21[32]	TEMPO, I_2 *or* NISac *or* *p*TSA, CH_2Cl_2, 0 °C	full conversion, very messy
22	TEMPO, DBDMH *or* DCDMH, CH_2Cl_2, 0 °C	full conversion, very messy
23	4-OH-TEMPO, DIH *or* DBDMH *or* DCDMH, CH_2Cl_2, 0 °C	full conversion, very messy
24	TEMPO, *N*-Iodophthalimide, CH_2Cl_2, 0 °C	full conversion, clean, 75% (+)-**20** – inseparable from phthalimide
25[33]	TEMPO, $CuCl_2$, CaH_2, MeCN, r.t.	messy, no (+)-**20** (TLC)
26[34]	HMDS, I_2, CH_2Cl_2, r.t.	no reaction

Yields were determined by ^1H NMR analysis of crude reaction mixtures, using $MeNO_2$ as internal standard; isolated yields are shown in parentheses.

Preparation of methyl ketone (+)-**9**: gold-catalysed Markovnikov hydration of (+)-**20**

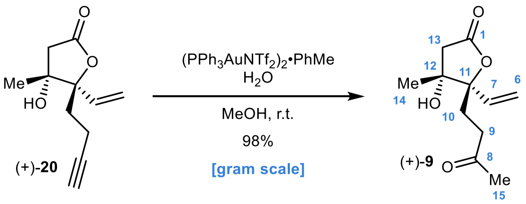

A Schlenk tube was charged with $(PPh_3AuNTf_2)_2$•PhMe (0.05 eq., 0.305 mmol, 479 mg) then evacuated and backfilled with dry N_2 three times. Alkyne (+)-**20** (1.0 eq., 6.10 mmol, 1.19 g) was dissolved in MeOH (61 ml) under N_2 then added to the Schlenk tube. Water (4.0 eq., 24.4 mmol, 439 mg, 0.44 ml) was added immediately, then the reaction mixture was stirred at room temperature for 4 h. TLC analysis indicated full conversion of the starting material. Following this, the reaction was quenched by the addition of Celite®, then the suspension was filtered through a pad of Celite®. The Celite® pad was washed with MeOH (2 × 20 ml) then the filtrate was concentrated *in vacuo*. The crude product was purified by column chromatography on silica gel (65% to 75% EtOAc in hexanes) to give the title compound (1.27 g, 5.96 mmol, 98%) as a yellow oil.

^{1}H NMR (400 MHz, $CDCl_3$) δ 5.74 (dd, J = 17.4, 10.9 Hz, 1H, C7H), 5.57 (dd, J = 17.4, 1.4 Hz, 1H, C6$H_{trans}H_{cis}$), 5.52 (dd, J = 10.9, 1.4 Hz, 1H, C6$H_{trans}H_{cis}$), 2.69 (d, J = 1.2 Hz, 1H, C13H_aH_b), 2.66 (s, 1H, C13H_aH_b), 2.70 – 2.57 (m, 1H, C9H_aH_b), 2.49 (ddd, J = 18.7, 9.1, 5.5 Hz, 1H, C9H_aH_b), 2.15 (s, 3H, C15H_3), 1.99 (ddd, J = 14.7, 9.3, 5.5 Hz, 1H, C10H_aH_b), 1.94 (d, J = 1.2 Hz, 1H, C12OH), 1.88 (ddd, J = 14.6, 9.1, 5.8 Hz, 1H, C10H_aH_b), 1.42 (s, 3H, C14H_3).

^{13}C NMR (101 MHz, $CDCl_3$) δ 207.8 (C8), 173.9 (C1), 132.6 (C7), 120.2 (C6), 92.3 (C11), 77.5 (C12), 42.9 (C13), 38.0 (C9), 30.3 (C15), 28.6 (C10), 21.9 (C14).

HRMS (APCI$^+$) calculated for $C_{11}H_{16}O_4Na$ $[M+Na]^+$ 235.0941, found 235.0931 (–4.25 ppm).

TLC (65% EtOAc in Hexanes): R_f = 0.29.

Physical state: yellow oil.

$[\alpha]_D^{26}$ = +28.6° (c = 0.10 g/100 ml, MeOH)

Table S7. Optimisation of the alkyne hydration[35].

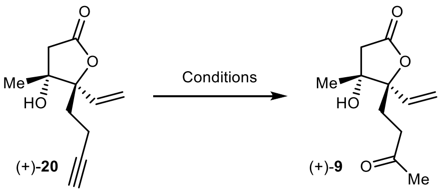

Entry	Conditions	Outcome
1	$AuCl_3$, H_2O, MeOH, r.t. to 50 °C	full conversion, low yield (<30%) – not reproducible
2	$Hg(OTf)_2(TMU)_2$, H_2O, MeCN, CH_2Cl_2, r.t.	full conversion, no (+)-**9** just decomposition
3	AuCl, H_2O, MeOH, r.t	full conversion, messy
4[36]	$(PPh_3AuNTf_2)_2$•PhMe, H_2O, MeOH, r.t	full conversion, clean, 98% (+)-**9**
5	IPrAuCl, $AgSbF_6$, H_2O, MeOH, 65 °C	very messy
6	$Au(PPh_3)Cl$, $AgSbF_6$, wet toluene, 40 °C	very messy
7	$AuCl_3$, PPh_3, $AgSbF_6$, H_2O, 1,4-dioxane, r.t.	messy, no (+)-**9**

Yields were determined by ^{1}H NMR analysis of crude reaction mixtures using $MeNO_2$ as internal standard.

Preparation of **10** Me ester: esterification of acid **21**

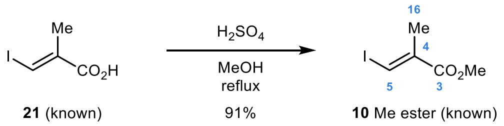

10 Me ester was synthesised according to a known procedure[37] from acid **21**[38]; and isolated in 91% yield.

Note: the product is volatile!

^{1}H NMR (400 MHz, $CDCl_3$) δ 7.80 (q, *J* = 1.2 Hz, 1H, C5*H*), 3.76 (s, 3H, O*Me*), 2.06 (d, *J* = 1.3 Hz, 3H, C16H_3).
^{13}C NMR (101 MHz, $CDCl_3$) δ 164.4 (C3), 139.6 (C4), 98.8 (C5), 52.5 (O*Me*), 20.5 (C16).
HRMS (APCI$^+$) calculated for $C_5H_8O_2I$ $[M+H]^+$ 226.9563, found 226.9555 (–3.52 ppm).
TLC (3% EtOAc in hexanes): R_f = 0.34.
Physical state: colourless oil.

Characterisation data is in agreement with the literature[37].

Preparation of **10** *t*-Bu ester: esterification of acid **21**

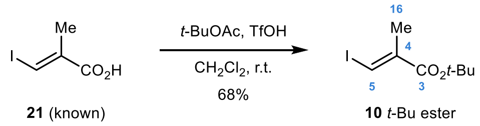

An oven-dried, 250 ml round bottom flask was equipped with a stirrer bar then cooled under vacuum and backfilled with dry N_2. Acid **21**[38] (1.0 eq., 25.0 mmol, 5.30 g) was added then the flask was evacuated and refilled with dry N_2 three times. Next, dry CH_2Cl_2 (125 ml) and *t*-BuOAc (10 eq., 250 mmol, 29.0 g, 33.7 ml) were added consecutively at r.t. and stirred until acid **21** was dissolved. TfOH (0.05 eq., 1.25 mmol, 188 mg, 0.11 ml) was added dropwise then the reaction mixture was stirred for 3 h at r.t. To quench the reaction, saturated aq. Na_2CO_3 solution (200 ml) and CH_2Cl_2 (100 ml) were added, the phases were separated, the aqueous phase was washed with CH_2Cl_2 (3 × 100 ml) and the combined organic layers were dried over $MgSO_4$, filtered and concentrated *in vacuo*. The crude product was purified by column chromatography on silica gel (1% Et_2O in pentanes) to give the title compound (4.54 g, 17.0 mmol, 68%) as a colourless oil.

Note: the product is moderately volatile.

^{1}H NMR (400 MHz, $CDCl_3$) δ 7.65 (q, *J* = 1.3 Hz, 1H, C5*H*), 2.02 (d, *J* = 1.2 Hz, 3H, C16H_3), 1.49 (s, 9H, C*Me*$_3$).
^{13}C NMR (101 MHz, $CDCl_3$) δ 163.1 (C3), 141.4 (C4), 97.5 (C5), 81.7 (*C*Me$_3$), 28.2 (C*Me*$_3$), 20.4 (C16).
HRMS (ESI$^+$) calculated for $C_8H_{13}O_2INa$ $[M+Na]^+$ 290.9852, found 290.9845 (–2.56 ppm).
TLC (1% Et_2O in pentanes): R_f = 0.35.
Physical state: colourless oil.

Preparation of **10** Ph ester: esterification of acid **21**

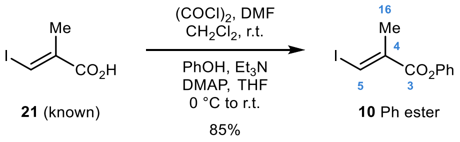

An oven-dried, 25 ml round bottom flask was equipped with a stirrer bar then cooled under vacuum then backfilled with dry N_2. Acid **21** (1.0 eq., 5.00 mmol, 1.06 g) was added then the flask was evacuated and refilled with dry N_2 three times. Next, dry CH_2Cl_2 (5 ml) was added and the mixture was stirred at r.t. until acid **21** was dissolved. Oxalyl chloride (1.2 eq., 6.00 mmol, 762 mg, 0.51 ml) was added followed by dry DMF (3 drops). ***Note**: A large amount of gas forms!* The reaction mixture was stirred for 3 h at r.t. then concentrated *in vacuo*. In the meantime, PhOH (1.5 eq., 7.50 mmol, 706 mg) and DMAP (0.1 eq., 0.50 mmol, 61.1 mg) were dissolved in dry THF (5 ml) under dry N_2 then Et_3N (2.0 eq., 10.0 mmol, 1.01 g, 1.39 ml) was added then cooled to 0 °C. The crude acid chloride was dissolved in dry THF (5 ml) under dry N_2 then added dropwise to the other flask at 0 °C then the reaction was stirred at 0 °C for 3.5 h before allowing it to warm to r.t. overnight (18 h). To quench the reaction, saturated aq. K_2CO_3 solution (20 ml), saturated aq. $NaHCO_3$ solution (20 ml), and EtOAc (25 ml) were added, the phases were separated, the aqueous phase was washed with EtOAc (3 × 25 ml) and the combined organic layers were dried over $MgSO_4$, filtered and concentrated *in vacuo*. The crude product was purified by column chromatography on silica gel (5% Et_2O in hexanes) to give the title compound (1.24 g, 4.26 mmol, 85%) as a light yellow oil.

Note: the ^{1}H NMR spectra was referenced to the water peak in $CDCl_3$ because the residual solvent signal of $CHCl_3$ overlaps with the aromatic protons.

^{1}H NMR (400 MHz, $CDCl_3$) δ 8.14 (q, J = 1.2 Hz, 1H, C5*H*), 7.46 – 7.37 (m, 2H, Ar*H*), 7.34 – 7.23 (m, 1H, Ar*H*), 7.17 – 7.07 (m, 2H, Ar*H*), 2.20 (d, J = 1.3 Hz, 3H, C16H_3).
^{13}C NMR (101 MHz, $CDCl_3$) δ 162.3 (C3), 150.9 (Ar*C*), 139.3 (C4), 129.6 (Ar*C*H), 126.1 (Ar*C*H), 121.6 (Ar*C*H), 101.0 (C5), 20.6 (C16).
HRMS (APCI$^+$) calculated for $C_{10}H_{10}O_2I$ $[M+H]^+$ 288.9720, found 288.9713 (–2.42 ppm).
TLC (5% Et_2O in hexanes): R_f = 0.28.
Physical state: light yellow oil.

Preparation of (+)-**8** *t*-Bu ester: Heck coupling of (+)-**9** and **10** *t*-Bu ester

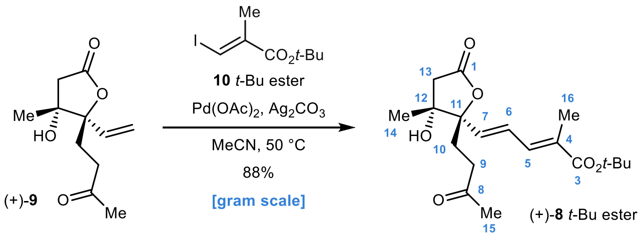

An oven-dried, 250 ml round bottom flask was equipped with a stirrer bar then cooled under vacuum then backfilled with dry N_2. Methyl ketone (+)-**9** (1.0 eq., 7.42 mmol, 1.58 g), $Pd(OAc)_2$ (0.1 eq., 0.742 mmol, 167 mg), and Ag_2CO_3 (1.3 eq., 9.64 mmol, 2.66 g) were added, the flask was evacuated and refilled with dry N_2 three times then dry MeCN (55 ml) was added. In a separate oven-dried round bottom flask, **10** *t*-Bu ester was dissolved in dry MeCN (20 ml) and the solution was added to the above mixture, then the reaction mixture was stirred at 50 °C for 15 h. TLC indicated full conversion of (+)-**9**, so the reaction mixture was cooled to r.t., then Celite® was added and the suspension was stirred at r.t. for 20 min. Next, it was filtered through a pad of Celite® and concentrated *in vacuo*. The crude product was purified by column chromatography on silica gel (60% to 80% EtOAc in hexanes) to give the title compound (2.29 g, 6.49 mmol, 88%) as an off-white solid.

Note: optimisation of the reaction was carried out using (+)-**10** Me ester (*vide infra*).

^{1}H NMR (400 MHz, $CDCl_3$) δ 7.06 (dt, *J* = 11.4, 1.2 Hz, 1H, C5*H*), 6.73 (dd, *J* = 15.3, 11.5 Hz, 1H, C6*H*), 5.84 (d, *J* = 15.3 Hz, 1H, C7*H*), 2.72 (dd, *J* = 17.3, 1.1 Hz, 1H, C13H_aH_b), 2.63 (d, *J* = 17.3 Hz, 1H, C13H_aH_b), 2.66 – 2.52 (m, 1H, C9H_aH_b), 2.44 (ddd, *J* = 18.7, 9.2, 5.4 Hz, 1H, C9H_aH_b), 2.29 – 2.23 (m, 1H, C12O*H*), 2.11 (s, 3H, C15H_3), 2.05 – 1.85 (m, 2H, C10H_2), 1.93 (d, *J* = 1.5 Hz, 3H, C16H_3), 1.48 (s, 9H, CMe_3), 1.44 (s, 3H, C14H_3).
^{13}C NMR (101 MHz, $CDCl_3$) δ 207.6 (C8), 173.8 (C1), 167.4 (C3), 135.0 (C5), 133.1 (C7), 131.7 (C4), 129.3 (C6), 92.3 (C11), 80.9 (*C*Me$_3$), 78.0 (C12), 43.0 (C13), 38.0 (C9), 30.2 (C15), 29.1 (C10), 28.2 (CMe_3), 22.0 (C14), 13.2 (C16).
HRMS (ESI$^+$) calculated for $C_{19}H_{28}O_6Na$ $[M+Na]^+$ 375.1778, found 375.1772 (–1.63 ppm).
TLC (60% EtOAc in hexanes): R_f = 0.29.
Physical state: off-white solid.
$[\alpha]_D^{26}$ = +28.4° (c = 0.13 g/100 ml, MeOH)

Preparation of (+)-**8** Me ester: Heck coupling of (+)-**9** and **10** Me ester

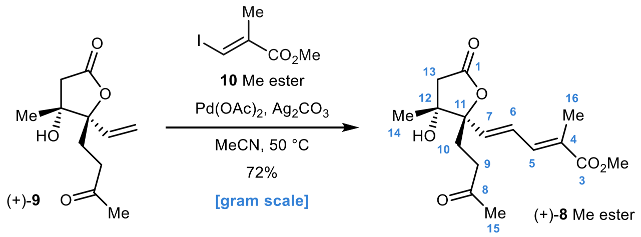

(+)-**8** Me ester was prepared analogously to (+)-**8** *t*-Bu ester (*vide supra*) using **10** Me ester and isolated in 72% yield (910 mg, 2.93 mmol) as a yellow oil after column chromatography on silica gel (50% CH_2Cl_2 in Et_2O).

^{1}H NMR (400 MHz, $CDCl_3$) δ 7.18 (dt, *J* = 11.4, 1.2 Hz, 1H, C5*H*), 6.77 (dd, *J* = 15.3, 11.5 Hz, 1H, C6*H*), 5.87 (d, *J* = 15.3 Hz, 1H, C7*H*), 3.77 (s, 3H, OCH_3), 2.74 (dd, *J* = 17.3, 1.4 Hz, 1H, C13H_aH_b), 2.65 (d, *J* = 17.3 Hz, 1H, C13H_aH_b), 2.68 – 2.55 (m, 1H, C9H_aH_b), 2.45 (ddd, *J* = 18.6, 9.1, 5.5 Hz, 1H, C9H_aH_b), 2.13 (s, 3H, C15H_3), 2.08 – 1.96 (m, 1H, C10H_aH_b), 1.99 (d, *J* = 1.4 Hz, 3H, C16H_3), 1.93 (bs, 1H, C12O*H*), 1.98 – 1.86 (m, 1H, C10H_aH_b), 1.46 (s, 3H, C14H_3).

^{13}C NMR (101 MHz, $CDCl_3$) δ 207.5 (C8), 173.6 (C1), 168.7 (C3), 136.0 (C5), 133.8 (C7), 130.0 (C4), 129.2 (C6), 92.2 (C11), 78.1 (C12), 52.2 (O*Me*), 43.0 (C13), 37.9 (C9), 30.3 (C15), 29.1 (C10), 22.1 (C14), 13.2 (C16).

HRMS (ESI$^+$) calculated for $C_{16}H_{22}O_6Na$ $[M+Na]^+$ 333.1309, found 333.1301 (–2.28 ppm).

TLC (50% MeCN in toluene): R_f = 0.63.

Physical state: yellow oil.

$[\alpha]_D^{26}$ = +45.8° (c = 0.12 g/100 ml, MeOH)

Table S8. Optimisation of the Heck reaction between (+)-**9** and **10** Me ester[39,40].

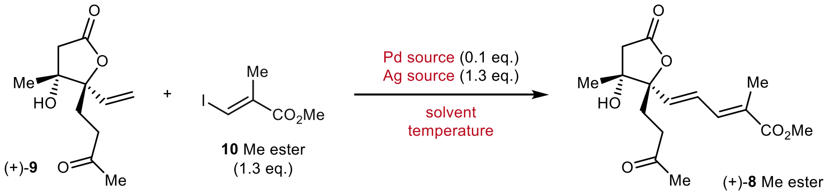

Entry	Pd source	Ag source	solvent	temperature	conversion	**8** Me ester
1	$Pd(OAc)_2$	Ag_2CO_3	DMF	50 °C	full (TLC)	45%
2	$Pd(OAc)_2$	Ag_2O	DMF	50 °C	not full (TLC)	n.d.
3	$Pd(OAc)_2$	AgOAc	DMF	50 °C	not full (TLC)	n.d.
4[a]	$Pd(OAc)_2$	–	DMF *or* MeCN	50 °C	no reaction (TLC)	n/a
5	$Pd(OAc)_2$	Ag_2CO_3	MeCN	50 °C	full (TLC)	77%
6	$Pd(OAc)_2$	Ag_2CO_3	MeCN	40 °C	7% **9**	78%
7	$Pd(OAc)_2$	Ag_2CO_3	MeCN	30 °C	20% **9**	65%
8	$Pd(OAc)_2$	AgOAc	MeCN	50 °C	10% **9**	66%
9[b]	$Pd(OAc)_2$	AgOAc	MeCN	50 °C	no reaction (TLC)	n/a
10	$Pd_2(dba)_3$	Ag_2CO_3	MeCN	50 °C	full (TLC)	66%

Yields were determined by ^{1}H NMR analysis of crude reaction mixtures using $MeNO_2$ as internal standard. Methyl ketone (+)-**9** is inseparable from **10** Me ester, therefore it's pivotal to fully consume (+)-**9** during the reaction. n.d. – not determined. [a]1.3 eq. of Et_3N was used. [b]0.2 eq. of P(*o*-tol)$_3$ was added.

Preparation of (+)-**8** Ph ester: Heck coupling of (+)-**9** and **10** Ph ester

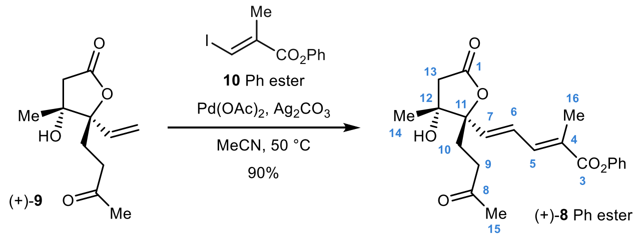

(+)-**8** Ph ester was prepared analogously to (+)-**8** *t*-Bu ester (*vide supra*) using **10** Ph ester and isolated in 90% yield (502 mg, 1.35 mmol) as an off-white solid after column chromatography on silica gel (60% EtOAc in hexanes).

^{1}H NMR (400 MHz, $CDCl_3$) δ 7.44 – 7.34 (m, 3H, C5*H* and 2 × ArC*H*), 7.26 – 7.21 (m, 1H, ArC*H*), 7.14 – 7.05 (m, 2H, ArC*H*), 6.81 (dd, *J* = 15.3, 11.5 Hz, 1H, C6*H*), 5.96 (d, *J* = 15.2 Hz, 1H, C7*H*), 2.73 (d, *J* = 17.4 Hz, 1H, C13H_aH$_b$), 2.64 (d, *J* = 17.3 Hz, 1H, C13H$_a$$H_b$), 2.61 (td, *J* = 9.5, 5.9 Hz, 1H, C9H_aH$_b$), 2.46 (ddd, *J* = 18.6, 9.0, 5.6 Hz, 1H, C9H$_a$$H_b$), 2.25 (s, 1H, C12O*H*), 2.13 (s, 3H, C15H_3), 2.10 (d, *J* = 1.4 Hz, 3H, C16H_3), 2.05 – 1.86 (m, 2H, C10H_2), 1.44 (s, 3H, C14H_3).

^{13}C NMR (101 MHz, $CDCl_3$) δ 207.6 (C8), 173.7 (C1), 166.8 (C3), 151.0 (Ar*C*), 137.8 (C5), 135.3 (C7), 129.6 (Ar*C*H), 129.0 (C4), 128.6 (C6), 125.9 (Ar*C*H), 121.7 (Ar*C*H), 92.3 (C11), 78.1 (C12), 43.0 (C13), 37.9 (C9), 30.2 (C15), 29.0 (C10), 22.0 (C14), 13.3 (C16).

HRMS (ESI$^+$) calculated for $C_{21}H_{24}O_6Na$ $[M+Na]^+$ 395.1465, found 395.1460 (–1.29 ppm).

TLC (60% EtOAc in hexanes): R_f = 0.24.

Physical state: off-white solid.

$[\alpha]_D^{26}$ = +35.8° (c = 0.10 g/100 ml, MeOH)

Preparation of **7** *t*-Bu ester and *iso*-**7** *t*-Bu ester: SmI_2-mediated cyclisation of (+)-**8** *t*-Bu ester

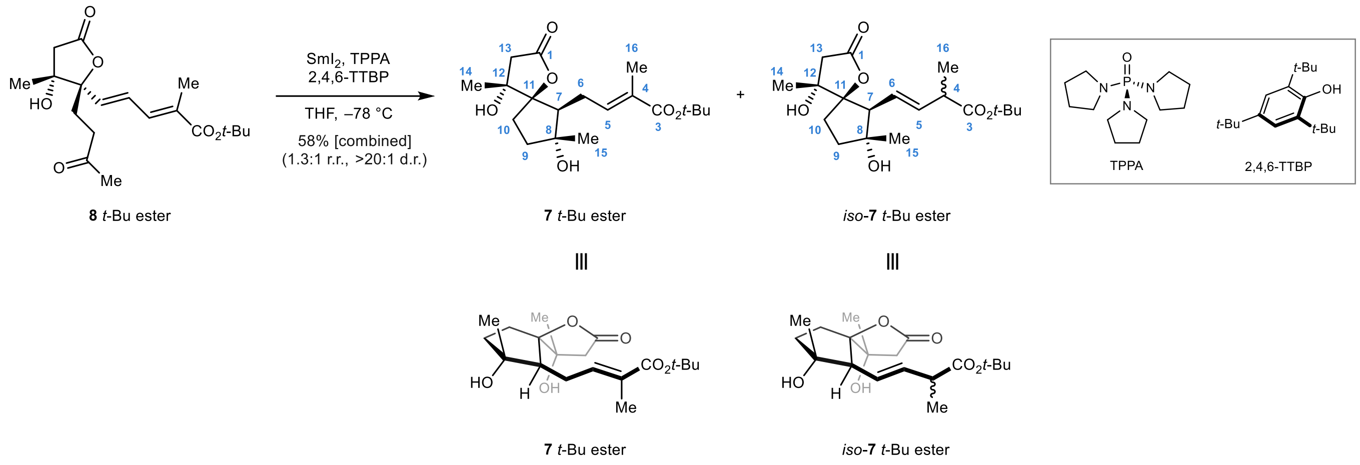

SmI_2 solution (0.1 M in THF, 30 ml) was prepared according to a literature procedure[1]. Commercial tripyrrolidinophosphoric acid triamide (TPPA) was vacuum distilled from CaH_2 at 168 °C (0.1 mbar) and stored under dry N_2; the reagent was degassed by 3 cycles of freeze-pump-thaw before use. 2,4,6-Tri-*tert*-butylphenol (2,4,6-TTBP) was used as received. THF was freshly distilled from Na/benzophenone and degassed by bubbling dry N_2 before use.

A flame-dried, 3-neck 100 ml round bottom flask was equipped with a stirrer bar and a jacketed addition funnel, cooled under vacuum then backfilled with dry N_2. The setup was evacuated and refilled with dry N_2 three times (Fig. S4A, *vide infra*), then a solution of SmI_2 (2.2 eq., 2.20 mmol, 22.0 ml, 0.1 M in THF) and TPPA (8.8 eq., 8.80 mmol, 2.26 g, 2.02 ml) were added to the round bottom flask *via* syringe – colour change from blue to deep purple. The mixture was cooled to –78 °C; meanwhile in a separate flame-dried round bottom flask 2,4,6-TTBP (2.0 eq., 2.00 mmol, 525 mg) was dissolved in dry, degassed THF (5 ml) under dry N_2 then added to the SmI_2/TPPA mixture at –78 °C *via* syringe (Fig. S4B). The jacketed addition funnel was cooled to –78 °C. In a separate flame-dried flask, (+)-**8** *t*-Bu ester (1.0 eq., 1.00 mmol, 352 mg) was dissolved in dry, degassed THF (5 ml) under dry N_2 then cooled to –78 °C. The cold solution of **8** *t*-Bu ester in THF was transferred to the cold jacketed addition funnel *via* cannula. ***Note****: it is imperative that the solution of* **8** *t-Bu ester is at –78 °C before adding to the reaction mixture to ensure high yield and regioselectivity.* Next, the cold solution of (+)-**8** *t*-Bu ester was added *via* the jacketed addition funnel to the reaction mixture (Fig. S4C) which turned deep brown in colour within a minute of addition but not immediately (Fig. S4D). The reaction was stirred at –78 °C for 10 min, then slowly warmed to r.t. by removing the dry ice/acetone bath. At r.t., the reaction was quenched by opening it to air and adding EtOAc (5 ml), saturated aq. Rochelle's salt solution (45 ml), and EtOAc (45 ml), sequentially. The phases were separated, the aqueous phase was washed with EtOAc (3 × 50 ml) and the combined organic layers were dried over $MgSO_4$, filtered and concentrated *in vacuo*. The crude product was purified by column chromatography on silica gel (25% to 40% to 60% MeCN in CH_2Cl_2) to give the title compound (204 mg, 0.575 mmol, 58%, 1.3: r.r. [inseparable], >20:1 d.r.) as a yellow foam.

Note: due to the complexity of the mixture only a handful of the peaks can be assigned to either **7** *t*-Bu ester or *iso*-**7** *t*-Bu ester in the mixed ^{1}H and ^{13}C NMR spectra. The other peaks are indistinguishable using standard 2D NMR techniques, such as ^{1}H -^{1}H COSY, ^{1}H -^{13}C HSQC, and ^{1}H -^{13}C HMBC. The major diastereomer of *iso*-**7** *t*-Bu ester was not assigned; and their ratio was not determined, because both are inconsequential.

NMR data for **7** *t*-Bu ester

^{1}H NMR (400 MHz, $CDCl_3$) δ 6.81 (ddq, *J* = 6.2, 4.4, 1.5 Hz, 1H, C5*H*), 2.64 (d, *J* = 17.1 Hz, 1H, C13H_aH$_b$), 2.53 (d, *J* = 17.1 Hz, 1H, C13H$_a$$H_b$), 2.39 (app d, *J* = 5.5 Hz, 1H, C7*H*), 1.82 (d, *J* = 1.3 Hz, 3H, C16H_3), 1.47 (s, 9H, CMe_3).

^{13}C NMR (101 MHz, $CDCl_3$) δ 174.7 (C1), 167.9 (C3), 140.3 (C5), 127.0 (C4), 51.9 (C7), 28.3 (CMe_3), 12.9 (C16).

NMR data for *iso*-**7** *t*-Bu ester (major dia)

^{1}H NMR (400 MHz, $CDCl_3$) δ 5.71 (dd, *J* = 15.5, 7.8 Hz, 1H, C5*H*), 5.58 (ddd, *J* = 15.4, 9.9, 0.9 Hz, 1H, C6*H*), 3.07 (app h, *J* = 7.1 Hz, 1H, C4*H*), 2.94 (d, *J* = 7.3 Hz, 1H, C7*H*).

^{13}C NMR (101 MHz, $CDCl_3$) δ 135.5 (C5), 127.0 (C6), 58.0 (C7), 43.8 (C4), 28.1 (CMe_3).

Analytical data of a 1.3:1 mixture of **7** *t*-Bu ester:*iso*-**7** *t*-Bu ester

HRMS (APCI^{-}) calculated for $C_{19}H_{29}O_6$ [*M-H*]$^{-}$ 353.1970, found 353.1953 (–4.71 ppm).

TLC (25% MeCN in CH_2Cl_2): R_f = 0.26.

Physical state: yellow foam.

$[\alpha]_D^{26}$ = +0.2° (c = 0.05 g/100 ml, MeOH)

Note on side-products: the major side-product is **27** which can form a cyclic structure **27'** under the work up conditions, however, these compounds are removed by column chromatography. **26** and **SI-17** also cyclises under work up conditions to form compounds similar to **27'**. However, side-product **SI-16**, or its Me or Ph ester analogue, was not observed as expected based on model studies[41].

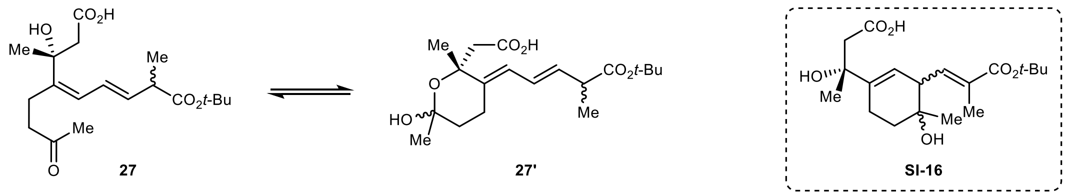

Figure S4. Structure of the side products observed (**27** and **27'**) and not observed, but expected (**SI-16**) during the key SmI_2-mediated cyclisation of (+)-**8** *t*-Bu ester.

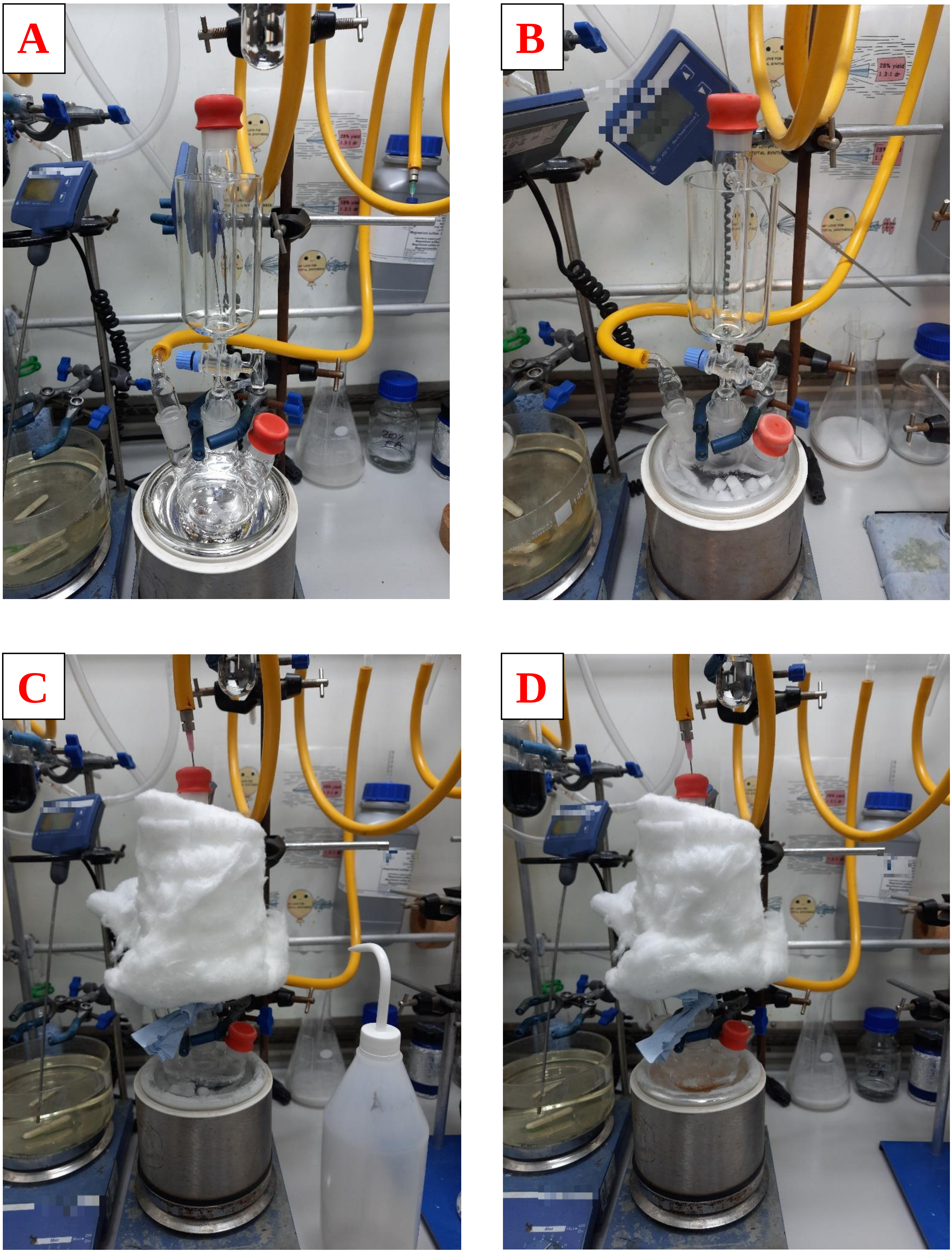

Figure S5. Setup for the SmI_2-mediated cyclisation of (+)-**8** *t*-Bu ester. **A**, the setup under dry N_2; **B**, the mixture of SmI_2, TPPA, and 2,4,6-TTBP in THF at –78 °C; **C**, during the addition of (+)-**8** *t*-Bu ester the jacket was covered to avoid condensation; **D**, at the end of the reaction the mixture turned deep brown in colour.

Table S9. Initial optimisation of the SmI_2-mediated ketyl-olefin coupling using (+)-**8** Me ester.

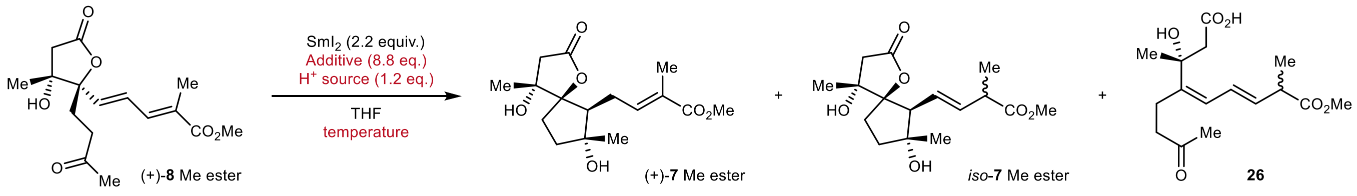

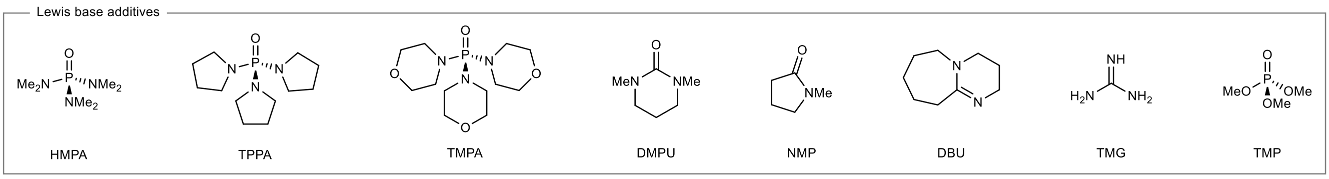

Entry	Additive	H^+ source	temperature	(+)-**8** Me ester	(+)-**7** Me ester	*iso*-**7** Me ester	**26**
1	–	–	r.t.	–	–	traces	39%
2	–	–	–78 °C	5%	–	traces	50%
3	–	MeOH	–78 °C	–	–	–	88%
4	–	*t*-BuOH	–78 °C	traces	–	–	10%
5	–	H_2O	–78 °C	8%	–	–	65%
6	HMPA	–	–78 °C	60%	–	–	5%
7	HMPA	MeOH	–78 °C	30%	–	3%	50%
8	HMPA	*t*-BuOH	–78 °C	35%	5%	10%	20%
9	Lewis bases[a]	MeOH	–78 °C	10% to 37%	–	<10%	36% to 65%
10	LiCl or LiBr	MeOH	–78 °C	–	–	–	60%, 12%
11	TPPA	–	–78 °C	–	–	10%	15%
12	TPPA	MeOH	–78 °C	–	2%	25%	40%
13[b]	TPPA	MeOH	–78 °C	–	12%	25%	40%
14	TPPA	*t*-BuOH	–78 °C	4%	5%	10%	35%
15	TPPA	H_2O	–78 °C	traces	–	20%	40%
16	TPPA	various[c]	–78 °C	0% to 30%	traces	5% to 22%	30% to 50%
17	TPPA (2.2 eq.)	MeOH	–78 °C	traces	–	10%	58%
18	TPPA (4.4 eq.)	MeOH	–78 °C	4%	–	11%	70%
19	TPPA (22 eq.)	MeOH	–78 °C	12%	traces	16%	36%
20	TPPA	MeOH (5.0 eq.)	–78 °C	13%	traces	16%	15%
21	TPPA	TFE	–78 °C	20%	–	23%	42%
22[b]	TPPA	TFE	–78 °C	–	–	20%	40%
23	TPPA	TFE	0 °C	50%	–	6%	25%
24	TPPA	TFE	r.t.	40%	–	9%	23%
25[d]	–	MeOH	–78 °C	traces	–	–	traces
26[d]	TPPA	MeOH	–78 °C	20%	–	–	30%
27[d,e]	–	MeOH	–30 °C	traces	–	traces	20%
28[d,e]	TPPA	MeOH	–30 °C	36%	–	traces	25%

The reactions were carried out on 0.05 mmol scale. Reagent order: if the additive or the proton source (or both) is solid they were added first to a vial strictly under dry N_2. Next, a solution of SmI_2 was added, followed by any liquid additives or proton sources. The mixture was cooled, then a room temperature solution of (+)-**8** Me ester in THF (0.1 M) was added dropwise. The order of addition is important since the reaction is usually over within 10 sec of addition of (+)-**8** Me ester. Yields were determined by ^{1}H NMR analysis of crude reaction mixtures using $MeNO_2$ as internal standard. [a]Lewis bases: TMPA, DMPU, NMP, Et_3N, DBU, TMG, TMP. [b]Reverse order of addition: (+)-**8** Me ester in THF cooled to –78 °C then the premixed SmI_2/TPPA/H^+ source mixture was added dropwise. [c]Proton sources: glycol, PhSH, PivOH, PhOH, HFIP. [d]$Sm(OTf)_2$ was used[42]. [e]Reactions were run in MeCN[43].

Table S10. Optimisation of the SmI_2-mediated ketyl-olefin coupling using (+)-**8** Ph ester.

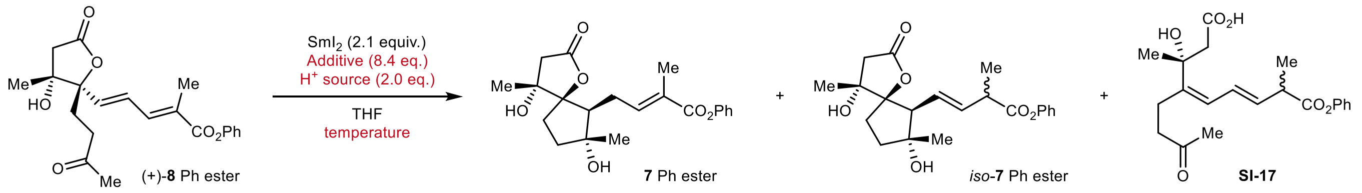

Entry	Additive	H^+ source	temperature	(+)-**8** Ph ester	**7** Ph ester	*iso*-**7** Ph ester	**SI-17**
1	–	MeOH	r.t.	13%	–	–	40%
2	–	*t*-BuOH	r.t.	15%	–	–	52%
3	–	MeOH	–78 °C	–	–	–	46%
4	–	*t*-BuOH	–78 °C	10%	–	–	23%
5	–	H_2O (10 eq.)	–78 °C	3%	–	–	53%
6	TPPA	–	–78 °C	–	–	35%	26%
7	TPPA	MeOH (1.2 eq.)	–78 °C	–	–	38%	16%
8	TPPA	*t*-BuOH	–78 °C	4%	–	38%	29%
9	TPPA	H_2O (10 eq.)	–78 °C	29%	–	19%	28%

The reactions were carried out on 0.05 mmol scale. Reagent order and conditions: see Table S9. Yields were determined by ^1H NMR analysis of crude reaction mixtures using $MeNO_2$ as internal standard.

Table S11. Initial optimisation of the SmI_2-mediated ketyl-olefin coupling using (+)-**8** *t*-Bu ester.

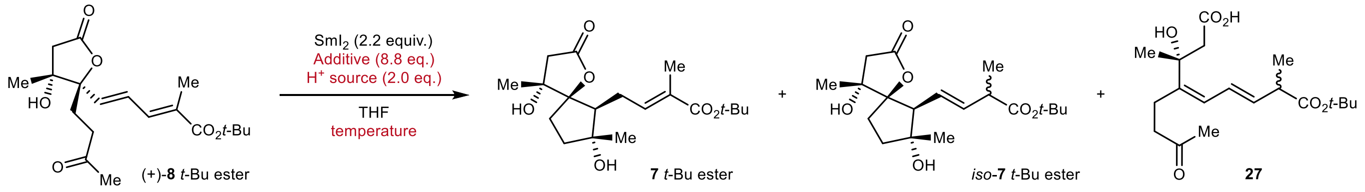

Entry	Additive	H^+ source	temperature	(+)-**8** *t*-Bu ester	**7** *t*-Bu ester	*iso*-**7** *t*-Bu ester	**27**
1	–	MeOH	r.t.	13%	0%	7%	41%
2	–	*t*-BuOH	r.t.	12%	0%	5%	31%
3	–	MeOH	–78 °C	20%	0%	0%	25%
4	–	*t*-BuOH	–78 °C	17%	0%	1%	20%
5	–	H_2O (10 eq.)	–78 °C	7%	0%	0%	51%
6	HMPA	–	–78 °C	10%	12%	24%	5%
7	HMPA	MeOH	–78 °C	–	4%	56%	5%
8	HMPA	*t*-BuOH	–78 °C	8%	14%	32%	19%
9	TPPA	*t*-BuOH	r.t.	54%	2%	22%	5%
10	TPPA	–	–78 °C	5%	12%	35%	15%
11	TPPA	MeOH	–78 °C	0%	9%	43%	10%
12	TPPA	*t*-BuOH	–78 °C	5%	15%	41%	17%
13	TPPA	H_2O (10 eq.)	–78 °C	26%	0%	35%	26%
14	TMPA	*t*-BuOH	–78 °C	0%	3%	36%	30%

The reactions were carried out on 0.05 mmol scale. Reagent order and conditions: see Table S9. Yields were determined by ^1H NMR analysis of crude reaction mixtures using $MeNO_2$ as internal standard. Diastereomers of **7** *t*-Bu ester and *iso*-**7** *t*-Bu ester other than the ones drawn were not observed.

Table S12. Optimisation of the SmI_2-mediated ketyl-olefin coupling using (+)-**8** *t*-Bu ester.

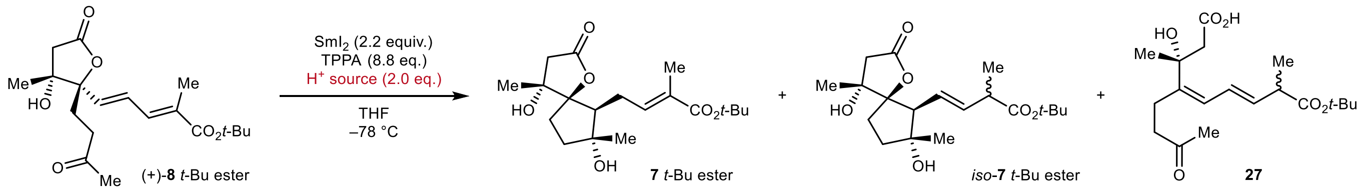

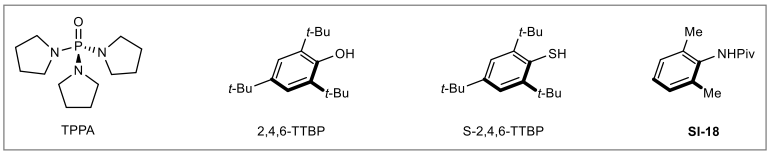

Entry	H^+ source	(+)-**8** *t*-Bu ester	**7** *t*-Bu ester	*iso*-**7** *t*-Bu ester	combined yield[a]	ratio[b]	**27**
1	3-Ethyl-3-pentanol	5%	17%	36%	53%	1:2.1	10%
2	2,3-Dimethyl-2-butanol	4%	17%	39%	56%	1:2.2	10%
3	F_9-*t*-BuOH	2%	traces	61%	69%	1:20	13%
4	TFE	14%	–	47%	47%	n/a	15%
5	HFIP	50%	–	34%	34%	n/a	20%
6	F_5-Phenol	53%	–	28%	28%	n/a	6%
7	$CF_3SO_2NH_2$	10%	–	58%	58%	n/a	10%
8	Ph_3COH	8%	17%	32%	49%	1:1.9	5%
9	2,6-Di-*tert*-butylphenol	3%	24%	overlaps	24%	n/a	messy
10	BHT	85%	–	–	–	n/a	2%
11	**SI-18**	5%	21%	26%	47%	1:1.2	13%
12	$(TMS)_3SiOH$	10%	14%	48%	62%	1:3.4	35%
13	imidazole	13%	5%	55%	57%	1:11	2%
14	benzimidazole	14%	–	68%	68%	n/a	5%
15	2-Me-benzimidazole	24%	5%	60%	65%	1:12	10%
16	2,4,6-TTBP	4%	30%	35%	65%	1:1.2	12%
17[c]	2,4,6-TTBP	5%	27%	27%	54%	1:1.0	15%
18[d]	2,4,6-TTBP	45%	3%	16%	19%	1:5.3	2%
19	2,4,6-TTBP (1.0 eq.)	7%	20%	33%	53%	1:1.7	13%
20	2,4,6-TTBP (5.0 eq.)	15%	25%	23%	48%	1.1:1	7%
21	S-2,4,6-TTBP[44,45]	22%	–	48%	48%	n/a	12%
22[e]	F_9-*t*-BuOH	50%	–	–	–	n/a	16%
23[e,f]	F_9-*t*-BuOH	50%	–	–	–	n/a	30%
24[e]	2,4,6-TTBP	50%	–	–	–	n/a	13%
25[e,f]	2,4,6-TTBP	67%	–	–	–	n/a	20%
26[g]	2,4,6-TTBP	–	traces	16%	16%	1:20	45%

The reactions were carried out on 0.05 mmol scale. Reagent order and conditions: see Table S9. Yields were determined by ^1H NMR analysis of crude reaction mixtures using $MeNO_2$ as internal standard. Diastereomers of **7** *t*-Bu ester and *iso*-**7** *t*-Bu ester other than the ones drawn were not observed. [a]Combined yield of **7** *t*-Bu ester and iso-**7** *t*-Bu ester. [b]Ratio of **7** *t*-Bu ester to iso-**7** *t*-Bu ester. [c]Reverse order of addition (see Table S9). [d]Reactions were run at r.t. [e]$Sm(OTf)_2$ was used[42]. [f]Reactions were run in MeCN[43]. [g]The control experiment, 0.20 mmol (+)-**8** Me ester was used.

Table S13. Optimisation of the SmI_2-mediated ketyl-olefin coupling using (+)-**8** *t*-Bu ester on larger scale.

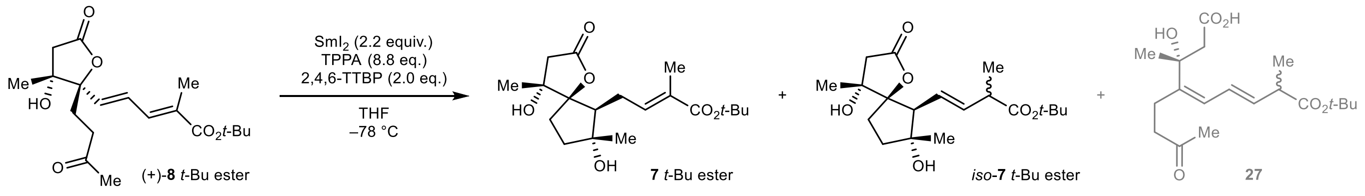

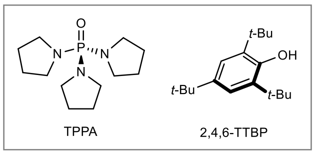

Entry	Scale	combined yield[a]	ratio[b]	Conditions
1	0.20 mmol	n/a	n/a	Reverse order[c]
2	0.20 mmol	51%	0.7:1	SM was cooled to –40 °C, then added *via* syringe
3	0.20 mmol	54%	0.7:1	SM was cooled to –78 °C, then added *via* syringe
4	1.00 mmol	46%	1.0:1	SM was cooled to –78 °C, then added *via* a cooled cannula
5	1.00 mmol	58%	1.3:1	SM was cooled to –78 °C, then added *via* the jacketed addition funnel
6[d]	0.20 mmol	16%	1:20	SM was cooled to –78 °C, then added *via* syringe

Reagent order and conditions: SmI_2 was added to a flame-dried flask under dry N_2 followed by TPPA. The mixture cooled to –78 °C, then a THF solution of 2,4,6-TTBP was added. The mixture was stirred for 5 min before the cooled solution of (+)-**8** *t*-Bu ester (SM) in THF (0.1 M) was added. [a]Combined yield of **7** *t*-Bu ester and iso-**7** *t*-Bu ester. [b]Ratio of **7** *t*-Bu ester to iso-**7** *t*-Bu ester. [c]Reverse order: 2,4,6-TTBP was added to the flask first followed by SmI_2 which almost immediately decolourised. [d]The control experiment, 0.20 mmol (+)-**8** Me ester was used.

Preparation of (+)-**7** Me ester: ester exchange and isomerisation of *iso*-**7** *t*-Bu ester

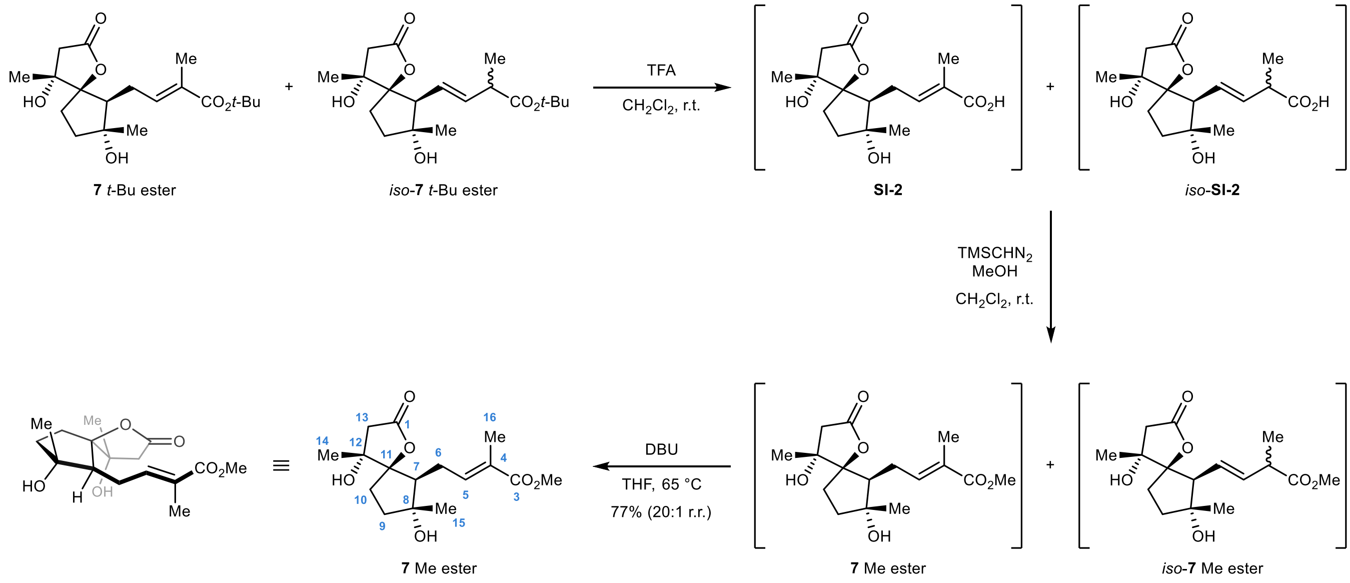

A flame-dried 25 ml round bottom flask was equipped with a stirrer bar and cooled under vacuum then backfilled with dry N_2. A 1.3:1 mixture of **7** *t*-Bu ester and *iso*-**7** *t*-Bu ester (1.0 eq., 0.547 mmol, 194 mg) was added then the flask was evacuated and refilled with dry N_2 three times. Dry CH_2Cl_2 (11 ml) was added followed by the dropwise addition of TFA (50.0 eq., 27.4 mmol, 3.12 g, 2.10 ml). The reaction mixture was stirred at r.t. for 1 h (TLC: full conversion); during which time it turned deep purple. Toluene (10 ml) was added then the volatiles (CH_2Cl_2 and TFA), but not toluene, were removed *in vacuo*. CH_2Cl_2 (15 ml) was added and the volatiles were removed once again leaving a toluene solution behind. CH_2Cl_2 (15 ml) was added, then the mixture was concentrated *in vacuo* yielding crude **SI-2** and *iso*-**SI-2**. CH_2Cl_2 (11 ml) and MeOH (2.1 ml) were added followed by the dropwise addition of a $TMSCHN_2$ solution (3.0 eq., 1.64 mmol, 0.82 ml, 2 M in hexanes). The reaction mixture was stirred at r.t. for 1 h (TLC: full conversion); then the mixture was concentrated *in vacuo* yielding crude **7** Me ester and *iso*-**7** Me ester. The flask was evacuated and refilled with dry N_2 three times. Dry THF (6 ml) and DBU (1.5 eq., 0.821 mmol, 125 mg, 0.12 ml) were added then the reaction was stirred at 65 °C for 4 h then allowed to cool to r.t. and stirred at r.t. overnight. The reaction was quenched with EtOAc (20 ml) and 1 M HCl (20 ml), the phases were separated then the organic phase was washed with 1 M HCl (3 × 20 ml). The aqueous phases were not combined but labelled AQ1 to AQ-4. AQ1 was washed with EtOAc (20 ml) then this organic phase was used to wash AQ2 to AQ4 as well. This process was repeated two more times to remove any trace of the product from the aqueous phases. The organic phases were combined and washed with saturated aq. $NaHCO_3$ solution (50 ml); the aqueous phase was washed with EtOAc (20 ml) then the combined organic layers were dried over $MgSO_4$, filtered and concentrated *in vacuo*. The crude product was purified by column chromatography on silica gel (40% MeCN in CH_2Cl_2) to give the title compound (131 mg, 0.420 mmol, 77%, 20:1 r.r.) as an off-white foam.

Note: it is necessary to use 50 eq. of TFA to remove the *t*-Bu ester, as fewer equivalents do not achieve full conversion even at 35 °C. The unsuccessful attempt to isomerise and cyclise **7** *t*-Bu ester is shown in Figure S7.

^{1}H NMR (400 MHz, $CDCl_3$) δ 6.95 (app ddd, J = 8.0, 6.2, 1.6 Hz, 1H, C5*H*), 3.72 (s, 3H, O*Me*), 2.91 (s, 1H, OH), 2.64 (d, J = 17.1 Hz, 1H, C13H_aH$_b$), 2.66 – 2.50 (m, 1H, C6H_aH$_b$), 2.53 (d, J = 17.1 Hz, 1H, C13H$_a$$H_b$), 2.47 – 2.32 (m, 1H, C6H$_a$$H_b$), 2.40 (app d, J = 1.6 Hz, 1H, C7*H*), 2.19 – 2.06 (m, 1H, C10H_aH$_b$), 1.86 (d, J = 1.4 Hz, 3H, C16H_3), 1.85 – 1.78 (m, 2H, C9H_aH$_b$ and OH), 1.74 – 1.59 (m, 2H, C9H$_a$$H_b$ and C10H$_a$$H_b$), 1.47 (s, 3H, C14$H_3$), 1.29 (s, 3H, C15$H_3$).

^{13}C NMR (101 MHz, $CDCl_3$) δ 174.8 (C1), 169.2 (C3), 142.3 (C5), 127.8 (C4), 97.5 (C11), 80.3 (C8), 79.1 (C12), 52.1 (O*Me*), 51.8 (C7), 43.6 (C13), 39.6 (C9), 33.7 (C10), 26.3 (C6), 23.8 (C15), 21.7 (C14), 12.7 (16).

HRMS (ESI^+) calculated for $C_{16}H_{24}O_6Na$ $[M+Na]^+$ 335.1465, found 335.1456 (–2.71 ppm).

TLC (40% MeCN in CH_2Cl_2): R_f = 0.28.

Physical state: off-white foam.

$[\alpha]_D^{26}$ = +5.8° (c = 0.15 g/100 ml, MeOH)

XRD: Single crystal analysis confirms the structure drawn and the relative and absolute stereochemistry.

Note on side-products: extended reaction time during isomerisation at 65 °C favours the formation of **SI-19** (Fig. S6). Analogous isomerisation of a mixture of **7** *t*-Bu ester and *iso*-**7** *t*-Bu ester to **7** *t*-Bu ester (with at least 5.5:1 r.r.) requires long reaction time (>2 days) therefore the formation of **SI-19** *t*-Bu ester becomes significant.

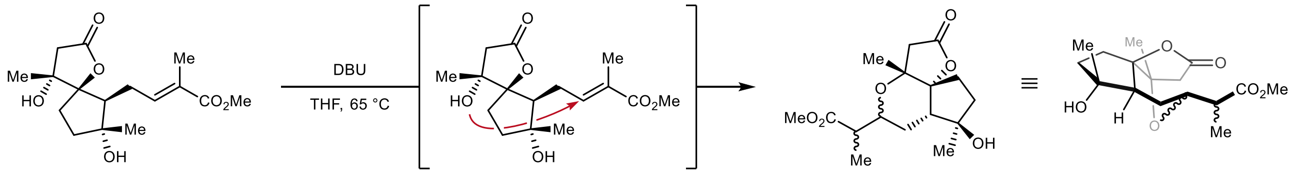

7 Me ester

SI-19

Figure S6. Formation of **SI-19** during the DBU mediated isomerisation of *iso*-**7** Me ester to **7** Me ester.

Table S14. Optimisation of the ester exchange of **7** *t*-Bu ester and *iso*-**7** *t*-Bu ester.

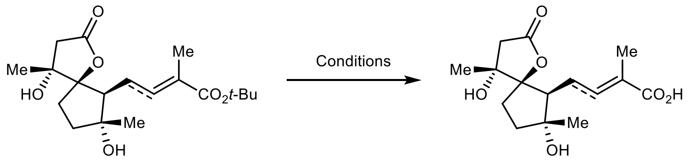

7 *t*-Bu ester
+ *iso*-**7** *t*-Bu ester

SI-2
+ *iso*-**SI-2**

Entry	Conditions	Outcome
1[46]	$ZnBr_2$, CH_2Cl_2, r.t.	messy, not full conversion (TLC)
2[47]	NaI, $CeCl_3$•$7H_2O$, MeCN, 80 °C	very messy, almost full conversion (TLC)
3	1 M aq. HCl – EtOAc (biphasic), r.t.	no conversion (TLC)
4	HCl (in Et_2O), THF, 65 °C	low conversion (TLC)
5[48]	AcCl, MeOH, r.t. or 40 °C or 50 °C	full conversion, moderate yield of **7** Me ester and *iso*-**7** Me ester (65% isolated)
6	TFA, CH_2Cl_2, r.t.	clean, full conversion, high yield of **SI-2** and *iso*-**SI-2** (>90% NMR)

Preparation of lactone (–)-**6**: second SmI_2-mediated ketyl-olefin cyclisation

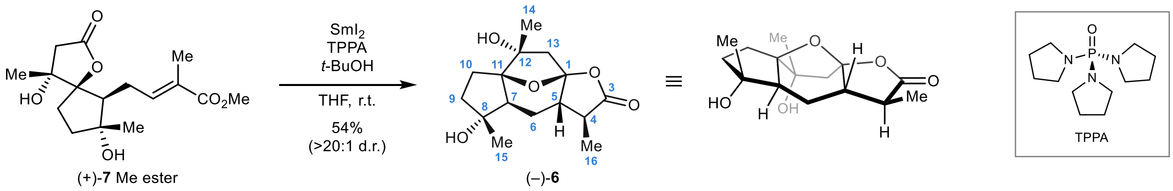

SmI_2 solution (0.1 M in THF, 30 ml) was prepared according to a literature procedure[1]. Commercial tripyrrolidinophosphoric acid triamide (TPPA) was vacuum distilled from CaH_2 at 168 °C (0.1 mbar) and stored under dry N_2; the reagent was degassed by 3 cycles of freeze-pump-thaw before use. Dry *tert*-butanol was used as received. THF was freshly distilled from Na/benzophenone and degassed by bubbling dry N_2 before use.

A flame-dried vial was equipped with a stirrer bar and cooled under vacuum then backfilled with dry N_2. The vial was evacuated and refilled with dry N_2 three times before a solution of SmI_2 (4.4 eq., 0.22 mmol, 2.20 mmol, 0.1 M in THF), TPPA (17.6 eq., 0.88 mmol, 226 mg, 0.20 ml), and dry *t*-BuOH (10 eq., 0.50 mmol, 37.1 mg, 48 µl) were added. In a separate flame-dried vial (+)-**7** Me ester (1.0 eq., 0.05 mmol, 15.6 mg) was dissolved in dry THF (0.5 ml) under dry N_2 then added to the above mixture dropwise at r.t., and stirred for 10 min before quenching it by opening it to air (deep purple to colourless). EtOAc (5 ml) and saturated aq. Rochelle's salt solution (5 ml) were added, the phases were separated, the aqueous phase was washed with EtOAc (2 × 3 ml) and the combined organic layers were dried over $MgSO_4$, filtered and concentrated *in vacuo*. TPPA was removed by passing the mixture through a short silica pad eluting with EtOAc. The crude product was purified by column chromatography on silica gel (35% MeCN in CH_2Cl_2) to give the title compound (7.6 mg, 0.027 mmol, 54%, >20:1 d.r.) as a white powder.

Note: It's imperative to use an excess of SmI_2 to favour the formation of (–)-**6** over **SI-20** (*vide infra*).

^{1}H NMR (400 MHz, $CDCl_3$) δ 2.42 (d, *J* = 7.6 Hz, 1H, C7*H*), 2.31 (d, *J* = 13.9 Hz, 1H, C13H_aH_b), 2.33 – 2.21 (m, 1H, C4*H*), 2.18 – 2.07 (m, 2H, C5*H* and C6H_aH_b), 2.00 (d, *J* = 13.7 Hz, 1H, C13H_aH_b), 2.07 – 1.94 (m, 2H, C9H_aH_b and C10H_aH_b), 1.89 – 1.68 (m, 3H, C6H_aH_b, C9H_aH_b and C10H_aH_b), 1.45 (s, 3H, C14H_3), 1.44 (s, 3H, C15H_3), 1.24 (d, *J* = 6.8 Hz, 3H, C16H_3).

^{13}C NMR (101 MHz, $CDCl_3$) δ 175.5 (C3), 108.0 (C1), 96.1 (C11), 80.2 (C8), 75.4 (C12), 48.4 (C5), 46.7 (C7), 44.6 (C13), 39.9 (C9), 39.8 (C4), 27.6 (C14), 26.6 (C10), 26.4 (C15), 21.2 (C6), 12.6 (C16).

HRMS (ESI^+) calculated for $C_{15}H_{22}O_5Na$ $[M+Na]^+$ 305.1359, found 305.1354 (–1.64 ppm).

TLC (10% EtOH in CH_2Cl_2): R_f = 0.31.

Physical state: white powder.

$[\alpha]_D^{26}$ = –11.8° (c = 0.15 g/100 ml, MeOH)

XRD: Single crystal analysis confirms the structure drawn and the relative and absolute stereochemistry.

Table S15. Optimisation of the second SmI_2-mediated ketyl-olefin coupling using (+)-**7** Me ester.

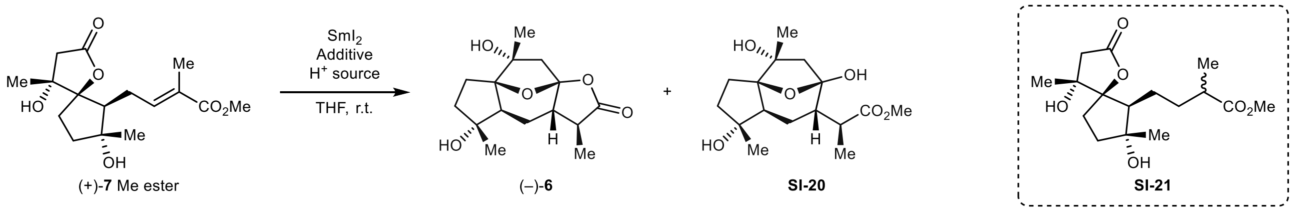

Entry	Equiv. of SmI_2	Additive (equiv.)	H^+ source (equiv.)	(+)-**7** Me ester	(–)-**6**	**SI-20**
1	2.2	–	MeOH (3.0)	–	–	50%
2	2.1	–	*t*-BuOH (1.1)	29%	–	50%
3[a]	2.1	–	H_2O (21)	–	–	–
4[b]	2.4	–	–	2%	–	33%
5	2.4	TPPA (9.6)	–	6%	7%	40%
6[b]	2.2	TPPA (8.8)	–	24%	15%	31%
7	2.2	TPPA (8.8)	MeOH (2.0)	5%	traces	55%
8	2.2	TPPA (8.8)	*t*-BuOH (2.0)	5%	10%	60%
9[c]	2.2	TPPA (8.8)	*t*-BuOH (2.0)	5%	traces	50%
10[b]	2.4	TPPA (9.6)	*t*-BuOH (2.0)	–	–	57%
11	2.4	TPPA (9.6)	*t*-BuOH (1.0)	–	–	65%
12	2.4	TPPA (9.6)	*t*-BuOH (3.0)	–	–	65%
13	2.4	TPPA (9.6)	*t*-BuOH (10)	–	15%	55%
14	2.4	HMPA (9.6)	*t*-BuOH (2.0)	–	–	37%
15	2.2	TPPA (8.8)	*t*-BuOH (2.0), H_2O (1.0)	12%	5%	63%
16[d]	2.4	TPPA (9.6)	–	–	15%	50%
17[e]	2.2	TPPA (8.8)	–	–	traces	50%
18[e]	2.2	TPPA (8.8)	*t*-BuOH (2.0)	–	traces	70%
19	2.2	LiBr (26.4)	–	–	–	50%[f]
20	2.2	LiBr (26.4)	*t*-BuOH (2.0)	–	–	63%[f]
21	2.2	TPPA (8.8), LiBr (8.8)	*t*-BuOH (2.0)	–	–	94%[f]
22	2.2	TPPA (4.4)	–	–	–	60%
23	4.4	TPPA (17.6)	–	–	40%	17%
24	4.4	TPPA (17.6)	*t*-BuOH (2.0)	–	45%	11%
25	4.4	TPPA (17.6)	*t*-BuOH (10)	–	62%	7%
26	6.6	TPPA (26.4)	*t*-BuOH (2.0)	–	53%	10%
27[e]	4.4	TPPA (17.6)	*t*-BuOH (10)	–	53%	32%
28	4.4	TPPA (17.6)	*t*-BuOH (50)	–	30%	45%

The reactions were carried out on 0.05 mmol scale. Reagent order: if the additive is solid it was added first to a vial strictly under dry N_2. Next, a solution of SmI_2 was added, then any liquid additives or proton sources. The temperature was adjusted then a room temperature solution of (+)-**7** Me ester in THF (0.1 M) was added dropwise. Yields were determined by ^1H NMR analysis of crude reaction mixtures using $MeNO_2$ as internal standard. [a]The only observed product is **SI-21** (75%). [b]The reaction was carried out at 35 °C. [c]The reaction was carried out at 0 °C. [d]Reverse order of addition: (+)-**7** Me ester in THF then the premixed SmI_2/TPPA/H^+ source mixture was added dropwise. [e](+)-**7** Me was added quickly, not dropwise. [f]Combined yield of **SI-20** and **SI-21**.

Synthesis of lactone (–)-**6** from **SI-20**.

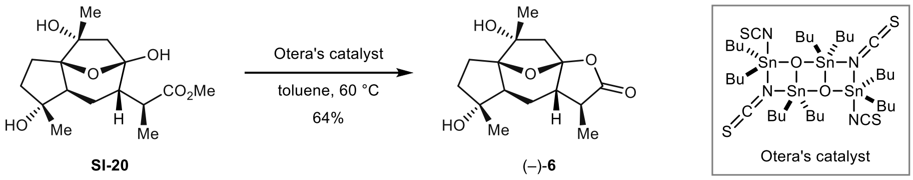

In a flame-dried microwave vial, **SI-20** (1.0 eq., 0.3 mmol, 94 mg) was suspended in dry toluene (6 ml) under dry N_2. Otera's catalyst (0.25 eq., 0.075 mmol, 90 mg) was added, then the reaction mixture was stirred at 60 °C overnight. The reaction mixture was concentrated *in vacuo* then the crude product was purified by column chromatography on silica gel (10% EtOH in CH_2Cl_2) to give the title compound (55 mg, 0.193 mmol, 64%) as a white powder.

Note: Strong base (KHMDS) decomposes **SI-20**; whereas acids, weak or strong (PPTS, *p*TSA) appear to form elimination products.

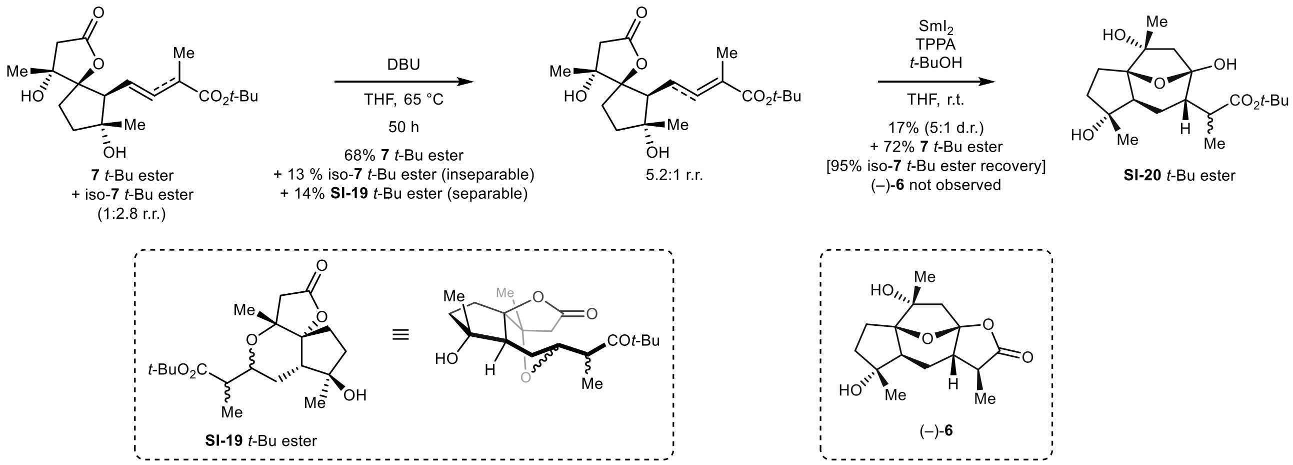

Figure S7. An attempt to isomerise and cyclise (+)-**7** *t*-Bu ester to form (–)-**6** directly without an ester exchange. As mentioned above, isomerisation forms significant amounts of **SI-19** *t*-Bu ester. The SmI_2-mediated cyclisation was unsuccessful due to the steric hindrance of the *t*-Bu ester group (*vide supra*).

Preparation of (–)-phaeocaulisin A (**1**): formal oxidation of lactone (–)-**6**

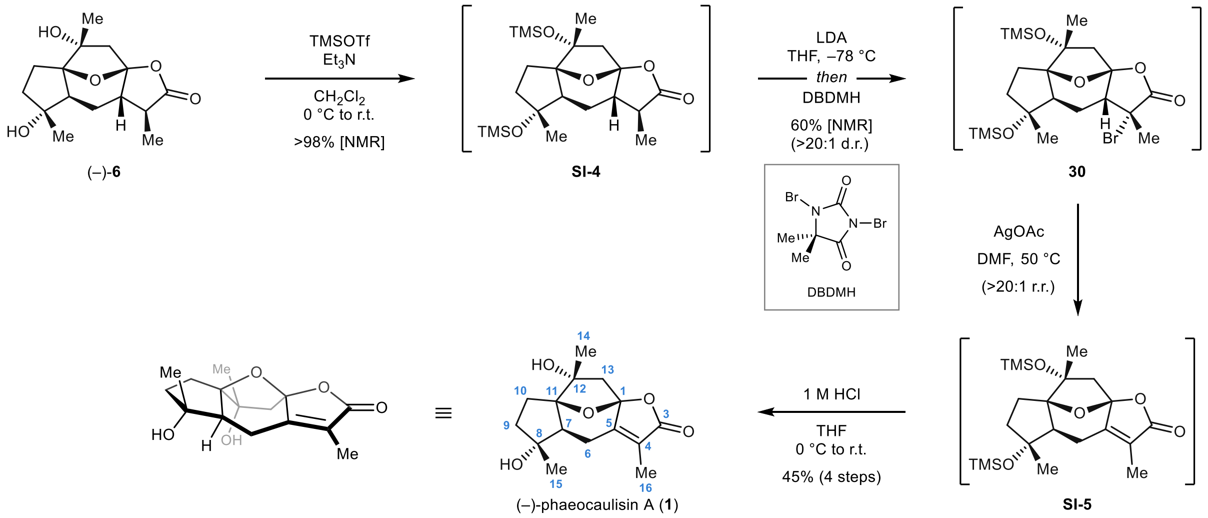

Step 1: An oven-dried mass spec vial was equipped with a stirrer bar and cooled under vacuum then backfilled with dry N_2. (–)-**6** (1.0 eq., 0.025 mmol, 7.1 mg) was added then the vial was evacuated and refilled with dry N_2 three times. Dry CH_2Cl_2 (0.5 ml) and dry Et_3N (7.0 eq., 0.175 mmol, 17.7 mg, 25 µl) were added then the solution was cooled to 0 °C before TMSOTf (4.0 eq., 0.10 mmol, 22.2 mg, 18 µl) was added. The reaction mixture was stirred at 0 °C for 30 min, then it was allowed to warm to r.t. and stirred for 30 min, after which TLC analysis indicated full consumption of (–)-**6**. 10% aq. $Na_2S_2O_3$ solution (2 ml) and EtOAc (2 ml) were added then the phases were separated, the aqueous phase was washed with EtOAc (3 × 3 ml) and the combined organic layers were dried over $MgSO_4$, filtered and concentrated *in vacuo*. The crude product was passed through a very short silica pad eluting with CH_2Cl_2 yielding a highly crystalline solid which was used in the next step without further purification.

Note: saturated aq. $NaHCO_3$ doesn't fully remove Et_3NH^+ from the organic phase, whereas Na_2CO_3 deprotects, presumably, the C12OTMS group; therefore it's important to use 10% aq. $Na_2S_2O_3$ solution during the work up.

Step 2: A lithium diisopropylamide stock solution was freshly prepared; a flame-dried vial was equipped with a stirrer bar and cooled under vacuum then backfilled with dry N_2. The vial was evacuated and refilled with dry N_2 three times before freshly distilled THF (4.72 ml) and dry diisopropylamine (0.50 mmol, 50.6 mg, 70 µl) were added. The solution was cooled to 0 °C, a solution of *n*-BuLi (0.50 mmol, 0.21 ml, 2.4 M in hexanes) was added dropwise, and then the mixture was stirred at 0 °C for 15 min before warming to r.t. The stock solution was not titrated; its concentration was calculated assuming additivity of volumes (assumed V_{total} = 5 ml).

A flame-dried vial was equipped with a stirrer bar and cooled under vacuum then backfilled with dry N_2. The vial was evacuated and refilled with dry N_2 three times before the above LDA stock solution (2.0 eq., 0.050 mmol, 0.5 ml) was added. The solution was cooled to –78 °C before **SI-4** was added dropwise in dry THF (0.5 ml), then the reaction mixture was stirred at –78 °C for 1 h. DBDMH (2.5 eq., 0.063 mmol, 17.9 mg) was added dropwise at –78 °C as a solution in dry THF (0.25 ml) and the reaction was stirred for 1 h at –78 °C. Next, the solution was slowly warmed to r.t. then stirred for 30 min before it was quenched with saturated aq. NH_4Cl solution (3 ml) and EtOAc (3 ml). The phases were separated, the aqueous phase was washed with EtOAc (3 × 3 ml) and the combined organic

layers were dried over $MgSO_4$, filtered and concentrated *in vacuo*. The crude product was purified by column chromatography on silica gel (7% Et_2O in hexanes) to give semi-pure bromolactone **30**.

Note: it's imperative to achieve full conversion, because (–)-**6** and (–)-phaeocaulisin A (**1**) are inseparable. Other brominating agents, such as NBS, CBr_4, CCl_3Br, 1,2-dibromotetrachloroethane, gave lower yields of **30**.

Step 3: Bromolactone **30** was dissolved in dry DMF (0.5 ml) under dry N_2 before AgOAc (3.0 eq., 0.045 mmol, 7.5 mg) was added, then the suspension was stirred at 50 °C for 5 h. Next, the mixture was passed through a short pad of Celite® and silica gel eluting with CH_2Cl_2, before concentration *in vacuo*. This process was repeated 3 more times to achieve full conversion of bromolactone **30** to **SI-5**. After the final concentration *in vacuo,* crude **SI-5** was used in the next step without further purification.

Step 4: Crude **SI-5** was dissolved in THF (1 ml) under air then cooled to 0 °C before 1 M HCl (0.1 ml) was added dropwise, then the reaction was stirred at 0 °C for 30 min. ***Note****: at this point mono-deprotection of, presumably, the C12OTMS group was achieved.* Next, the mixture was warmed to r.t., stirred for 30 min, then 1 M HCl (0.1 ml) was added and the mixture was stirred for 1 h at r.t. after which TLC analysis indicated full conversion. The reaction was quenched by adding saturated aq. $NaHCO_3$ (3 ml) and EtOAc (3 ml), then the phases were separated, the aqueous phase was washed with EtOAc (3 × 3 ml) and the combined organic layers were dried over $MgSO_4$, filtered and concentrated *in vacuo*. The crude product was purified by column chromatography on silica gel (40% MeCN in CH_2Cl_2) to give (–)-phaeocaulisin A (**1**, 3.2 mg, 0.0113 mmol, 45%, 4 steps) as a white solid. The yield was confirmed by quantitative ^1H NMR spectroscopic analysis.

Note: We propose that the naturally occurring enantiomer of phaeocaulisin A can be obtained following the same route but by a subtle variation of the enantioselective Sharpless dihydroxylation conditions (i.e. using the pseudo-enantiomer of $(DHQD)_2Pyr$, $(DHQ)_2Pyr$, to obtain *ent*-**17** from **16**).

^{1}H NMR (500 MHz, CD_3OD) δ 2.93 (dd, *J* = 16.9, 1.0 Hz, 1H, C6H_aH_b), 2.83 (ddq, *J* = 16.9, 8.8, 2.1 Hz, 1H, C6H_aH_b), 2.62 (broad d, *J* = 8.7 Hz, 1H, C7*H*), 2.27 (d, *J* = 13.7 Hz, 1H, C13H_aH_b), 2.16 (d, *J* = 13.8 Hz, 1H, C13H_aH_b), 2.08 (ddd, *J* = 14.1, 11.2, 7.5 Hz, 1H, C10H_aH_b), 1.88 (ddd, *J* = 13.4, 9.1, 7.5 Hz, 1H, C9H_aH_b), 1.85 – 1.74 (m, 1H, C9H_aH_b), 1.79 (d, *J* = 2.1 Hz, 3H, C16H_3), 1.64 (ddd, *J* = 13.9, 9.1, 4.6 Hz, 1H, C10H_aH_b), 1.40 (s, 3H, C14H_3), 1.17 (s, 3H, C15H_3).
^{13}C NMR (151 MHz, CD_3OD) δ 174.1 (C3), 162.3 (C5), 118.7 (C4), 106.9 (C1), 96.2 (C11), 80.7 (C8), 76.8 (C12), 50.3 (C7), 46.9 (C13), 39.6 (C9), 28.3 (C10), 27.7 (C14), 25.8 (C15), 21.0 (C6), 7.7 (C16).
HRMS (ESI^+) calculated for $C_{15}H_{20}O_5Na$ $[M+Na]^+$ 303.1203, found 303.1194 (–2.95 ppm).
($APCI^+$) calculated for $C_{15}H_{21}O_5$ $[M+H]^+$ 281.1384, found 281.1381 (–0.89 ppm).
TLC (40% MeCN in CH_2Cl_2): R_f = 0.23.
Physical state: white solid.
$[\alpha]_D^{26}$ = –40.6° (c = 0.13 g/100 ml, MeOH)
XRD: Single crystal analysis confirms the structure drawn and the relative and absolute stereochemistry.

Table S16. Comparison of ^{1}H and ^{13}C NMR data for natural and synthetic phaeocaulisin A.

Comparison of ^{1}H NMR data[a]

Proton number	(+)-phaeocaulisin A (natural)	(–)-phaeocaulisin A (synthetic)
C6H_a	2.87 (d, J = 16.8 Hz)	2.93 (dd, J = 16.9, 1.0 Hz)
C6H_b	2.78 (ddd, J = 16.8, 8.8, 2.0 Hz)	2.83 (ddq, J = 16.9, 8.8, 2.1 Hz)
C7H	2.56 (d, J = 8.8 Hz)	2.62 (broad d, J = 8.7 Hz)
C9H_a	1.82 (m)	1.88 (ddd, J = 13.4, 9.1, 7.5 Hz)
C9H_b	1.76 (m)	1.85 – 1.74 (m)
C10H_a	2.02 (m)	2.08 (ddd, J = 14.1, 11.2, 7.5 Hz)
C10H_b	1.58 (m)	1.64 (ddd, J = 13.9, 9.1, 4.6 Hz)
C13H_a	2.22 (d, J = 13.8 Hz)	2.27 (d, J = 13.7 Hz)
C13H_b	2.12 (d, J = 13.8 Hz)	2.16 (d, J = 13.8 Hz)
C14H_3	1.34 (s)	1.40 (s)
C15H_3	1.11 (s)	1.17 (s)
C16H_3	1.74 (d, J = 1.9 Hz)	1.79 (d, J = 2.1 Hz)

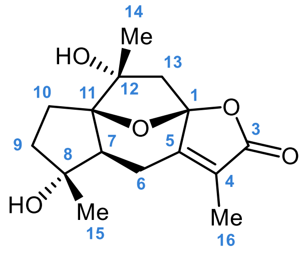

(–)-phaeocaulisin A
(*synthetic*)

Comparison of ^{13}C NMR data[a]

Carbon number	(+)-phaeocaulisin A (natural)	(–)-phaeocaulisin A (synthetic)	Shift difference, Δδ
1	107.1	106.9	0.2
3	174.3	174.1	0.2
4	118.8	118.7	0.1
5	162.4	162.3	0.1
6	21.1	21.0	0.1
7	50.4	50.3	0.1
8	80.8	80.7	0.1
9	39.8	39.6	0.2
10	28.5	28.3	0.2
11	96.3	96.2	0.1
12	76.9	76.8	0.1
13	47.1	46.9	0.2
14	27.9	27.7	0.2
15	25.9	25.8	0.1
16	7.9	7.7	0.2

[a]Important note: in the isolation paper, the authors claim that they have reported the shifts relative to TMS, however there is no TMS peak in either the ^{1}H or ^{13}C spectra they have provided[4]. Our spectra are referenced to the residual solvent peak.

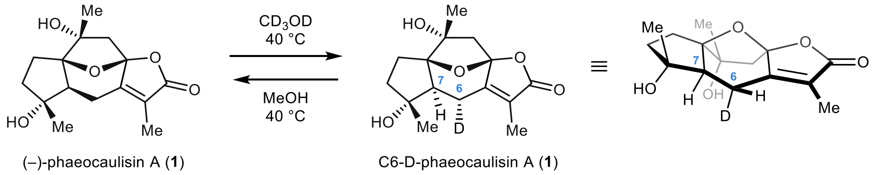

Figure S8. Unexpected deuteration of (–)-phaeocaulisin A (**1**) at C6. (–)-phaeocaulisin A was dissolved in CD_3OD, then concentrated *in vacuo*; this process was repeated three times before the NMR measurements were carried out. Deuteration was apparent as the C6 appeared as a triplet in the ^{13}C spectrum. The stereochemistry at C6 in C6-D-phaeocaulisin A was assigned based on the disappearance of the coupling between the remaining proton at C6 and the proton at C7 – both signals became apparent singlets – combined with single crystal analysis of the natural product; XRD analysis shows that the proton pointing 'up' at C6 sits at almost 90° to the proton at C7 therefore their coupling constant is too small to be observed providing the apparent singlets. Thus the deuterium must have replaced the proton pointing 'down'. The deuterium can be replaced by a proton by repeating the process with MeOH.

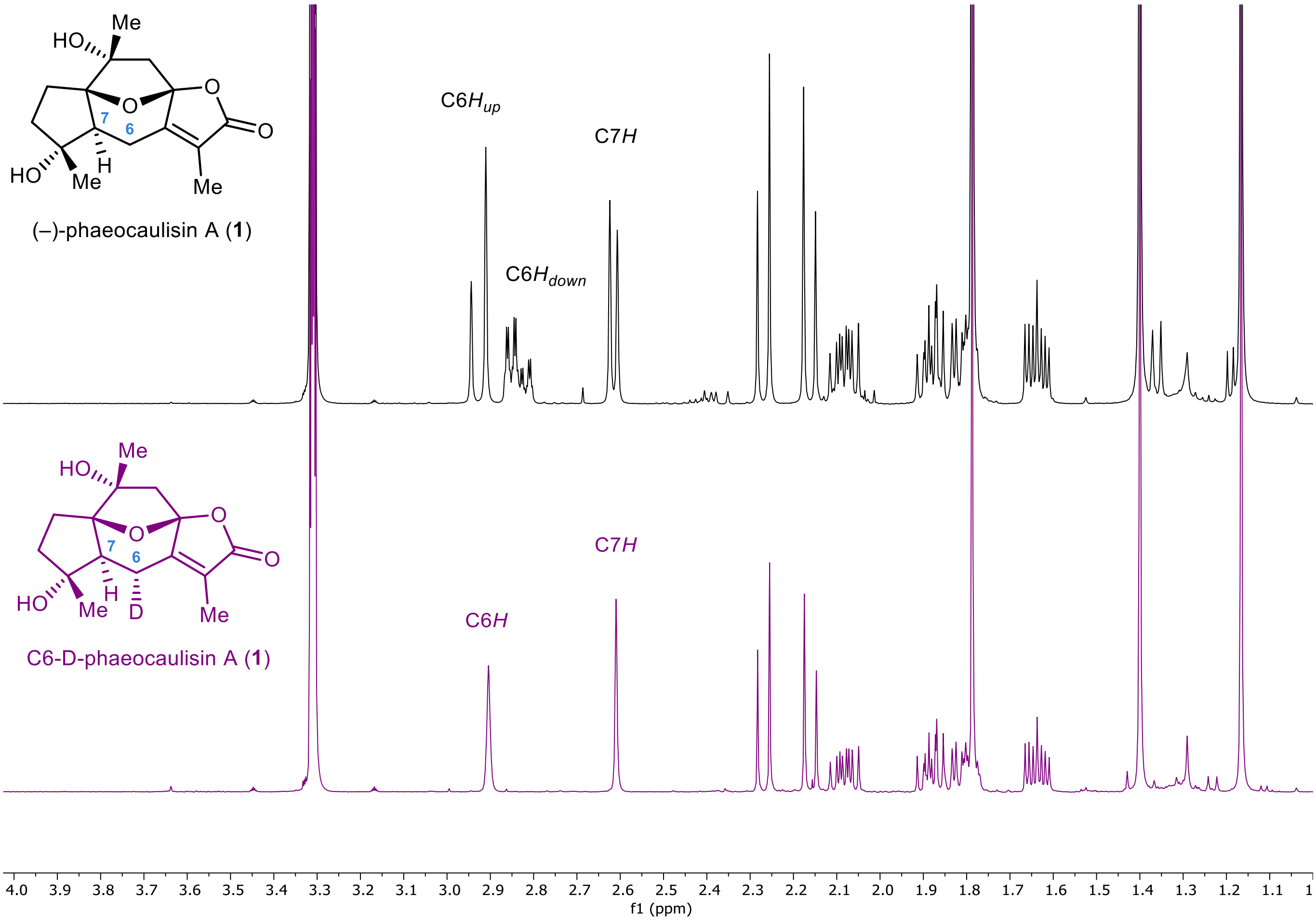

Figure S9. Comparison of the ^1H NMR spectra of (–)-phaeocaulisin A (**1**) and C6-D-phaeocaulisin A.

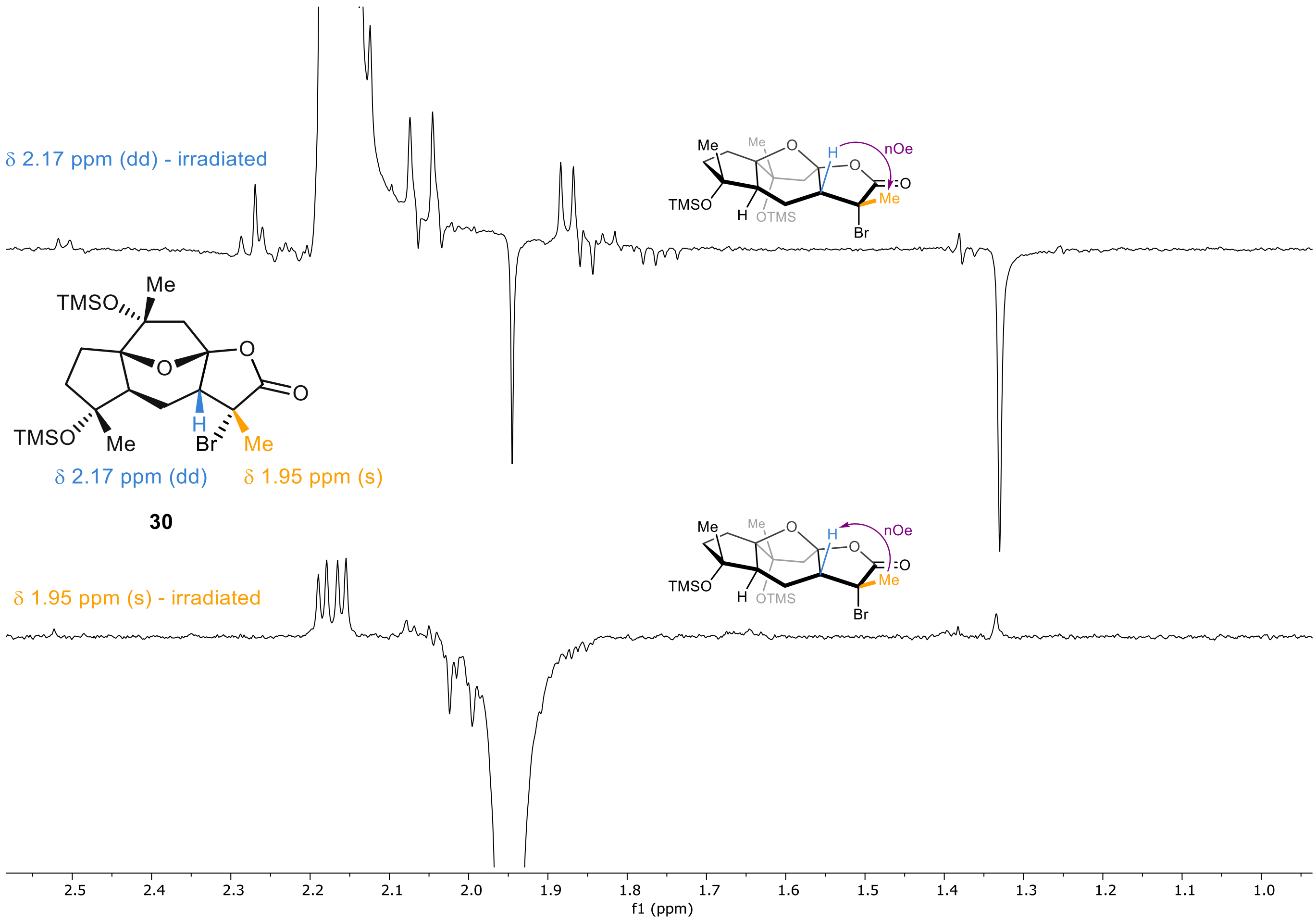

Figure S10. Selective ^{1}H-^{1}H nOe analysis of semi-pure bromolactone **30**. The peaks at δ 2.17 ppm and 1.95 ppm were separately and selectively irradiated. Irradiation of each resulted in a positive correlation to the other therefore confirming the relative stereochemistry of bromolactone **30**.

Unsuccessful formal oxidation of (–)-**6**: preparation of **SI-22** and **SI-23**

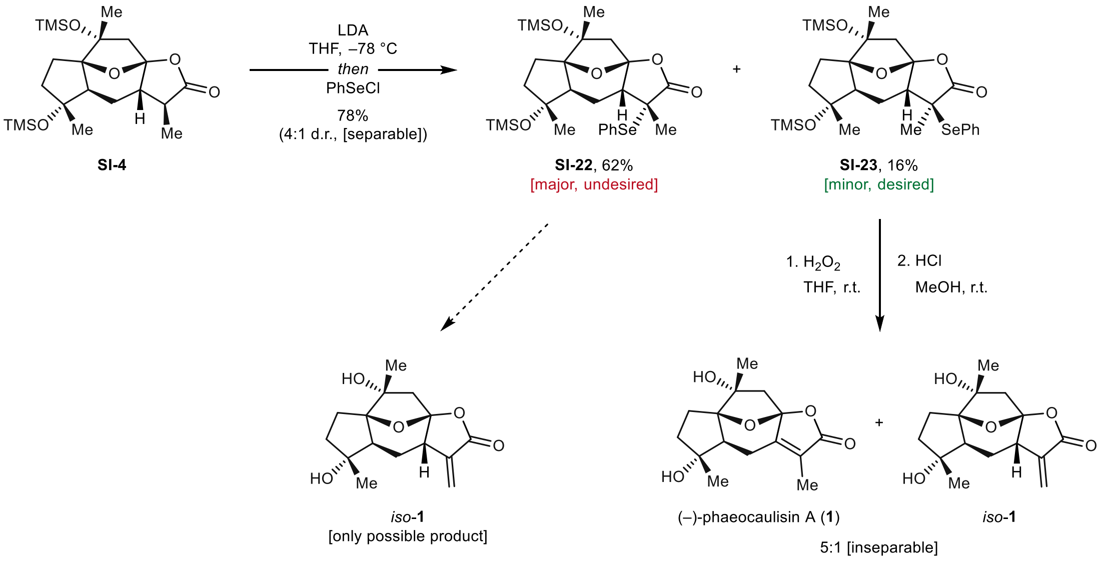

Figure S11. Initial attempt at the formal oxidation of (–)-**6** *via* selenenylation and selenoxide elimination. The relative stereochemistry of **SI-22** and **SI-23** were established by selective ^{1}H-^{1}H nOe analysis.

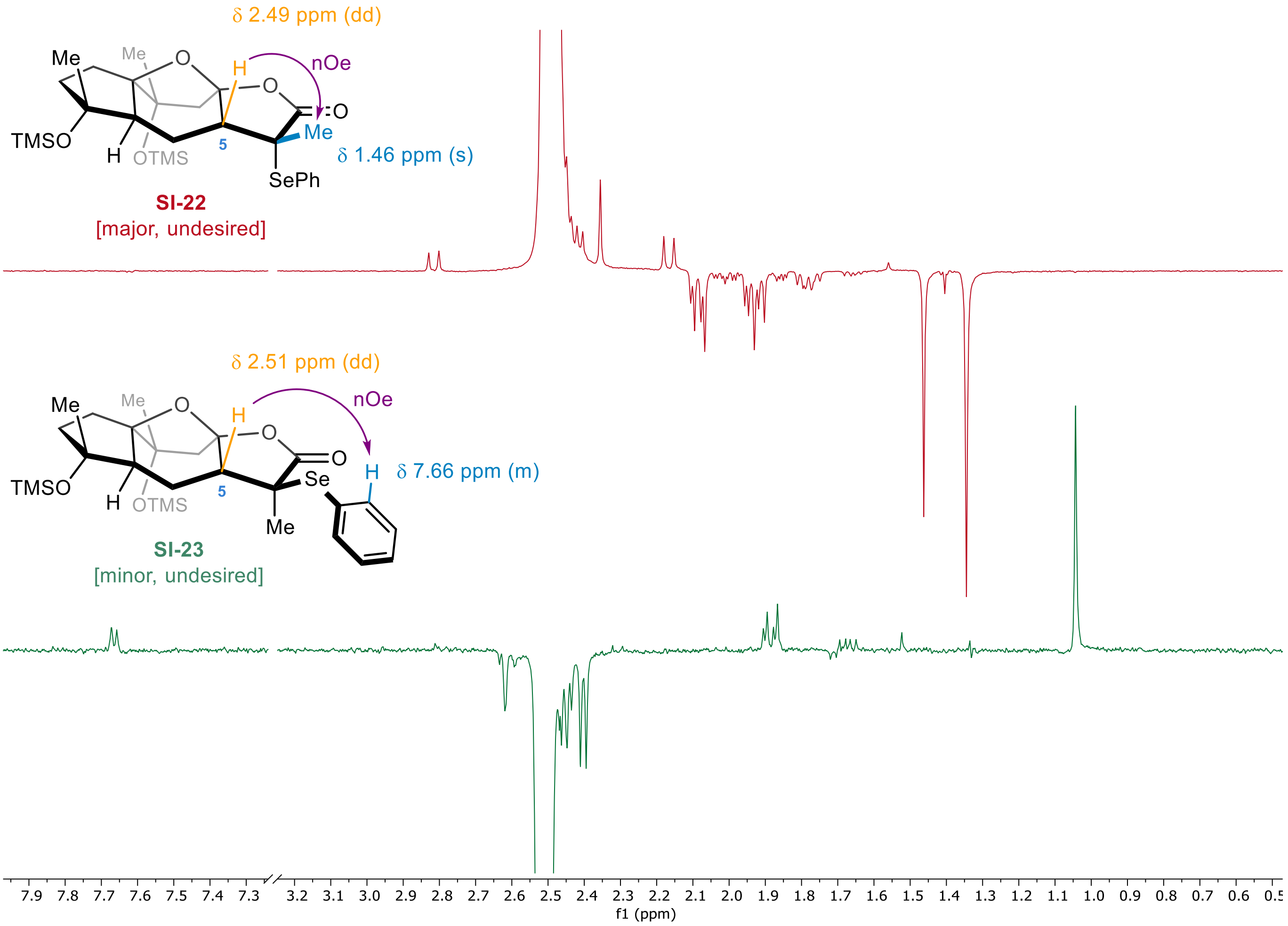

Figure S12. Selective ^{1}H-^{1}H nOe analysis of **SI-22** (undesired, red) and **SI-23** (desired, green) to establish their relative stereochemistry. The C5*H* was irradiated in both diastereomers.

Table S17. Attempted isomerisation of *iso*-**1** to (–)-phaeocaulisin A (**1**).

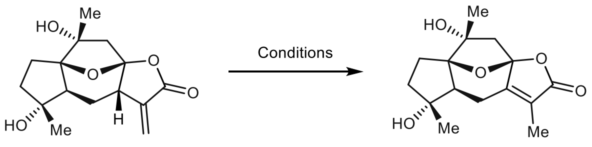

iso-**1** (–)-phaeocaulisin A (**1**)

Entry	Conditions	Outcome
1[51]	$RhCl_3$, EtOH, 60 °C	no isomerisation, but lactone ring is opened by EtOH
2[52]	Co($Sal^{t\text{-Bu},t\text{-Bu}}$)Cl, $PhSiH_3$, benzene, 60 °C	decomposition
3[53]	$HRh(PPh_3)_4$, toluene, 120 °C	no reaction
4[54]	$Pd(OAc)_2$, DBU, toluene, 110 °C	decomposition
5[53]	$HRh(PPh_3)_3CO$, 1,4-dioxane, r.t.	no isomerisation, 80% recovered *iso*-**1**

Discussion: *syn*-elimination upon oxidation of the Se-atom in the major diastereomer of the selenenylation reaction (Fig. S12), **SI-22**, could only promote the formation of the exocyclic double bond, thus giving rise to phaeocaulisin A (**1**) regioisomer, *iso*-**1**, after deprotection. On the other hand, oxidation of the minor diastereomer, **SI-23**, followed by deprotection, lead to an inseparable 5:1 mixture of (–)-phaeocaulisin A (**1**) and *iso*-**1**. Furthermore, because the isomerisation of *iso*-**1** to (–)-phaeocaulisin A (**1**) was unsuccessful and they are inseparable (Table S17), the selenenylation route was abandoned in favour of the bromination route. The bromination route exploits the tendency of the enolate to react with electrophiles on the bottom face. A subsequent anti-elimination of bromide **30** then yields (–)-phaeocaulisin A (**1**).

4. Unsuccessful approaches

Heck coupling between **9** and **SI-24**

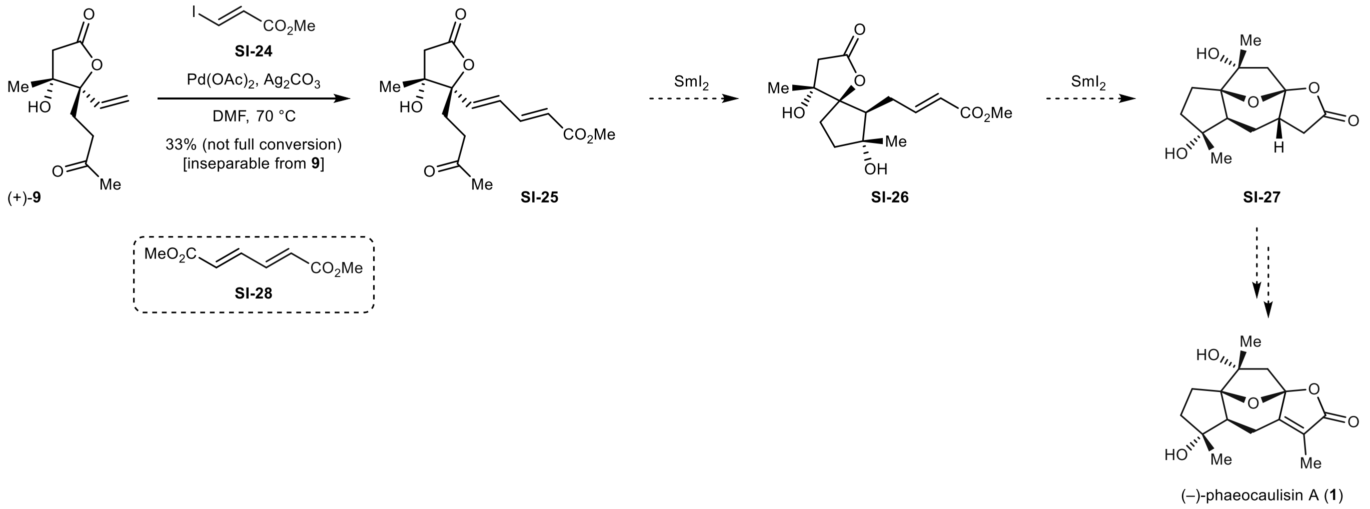

Figure S13. Attempted Heck cross coupling between ketone **9** and **SI-24**. The cross coupling was plagued by low yield under a variety of conditions due to prevalent homo-coupling of **SI-24** to form **SI-28**. Furthermore, ketone **9** could not be separated from **SI-25**.

Grubbs cross-metathesis between ketone **9** and **SI-29**.

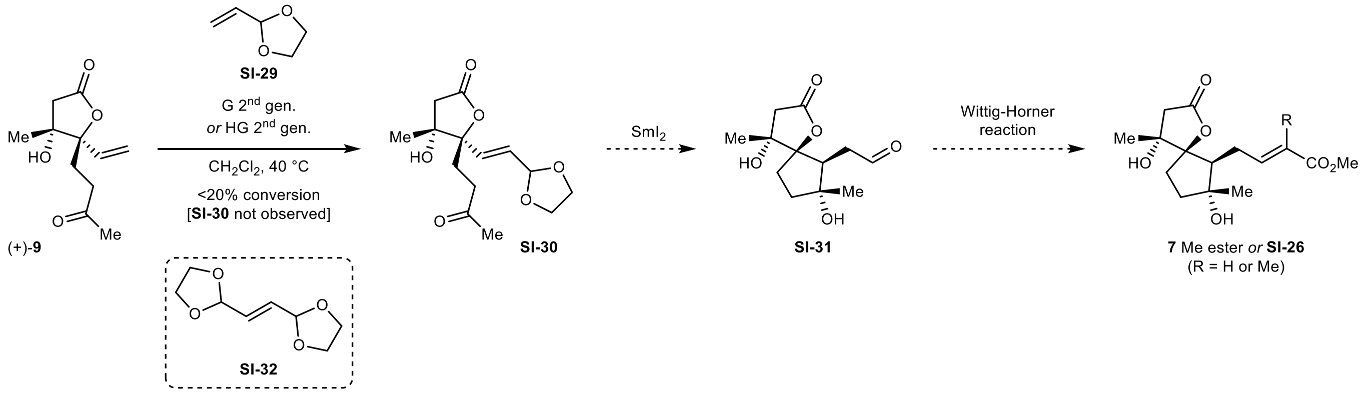

Figure S14. Attempted Grubbs cross-metathesis to form **SI-30**. The desired product was not observed under a variety of conditions, however, **SI-32** was formed under the reaction conditions. When the tertiary hydroxyl group in **9** was protected as its TMS ether the cross-metathesis proceeded with <5% conversion and, again, **SI-32** was the major product.

5. Model studies towards (–)-phaeocaulisin A (1)

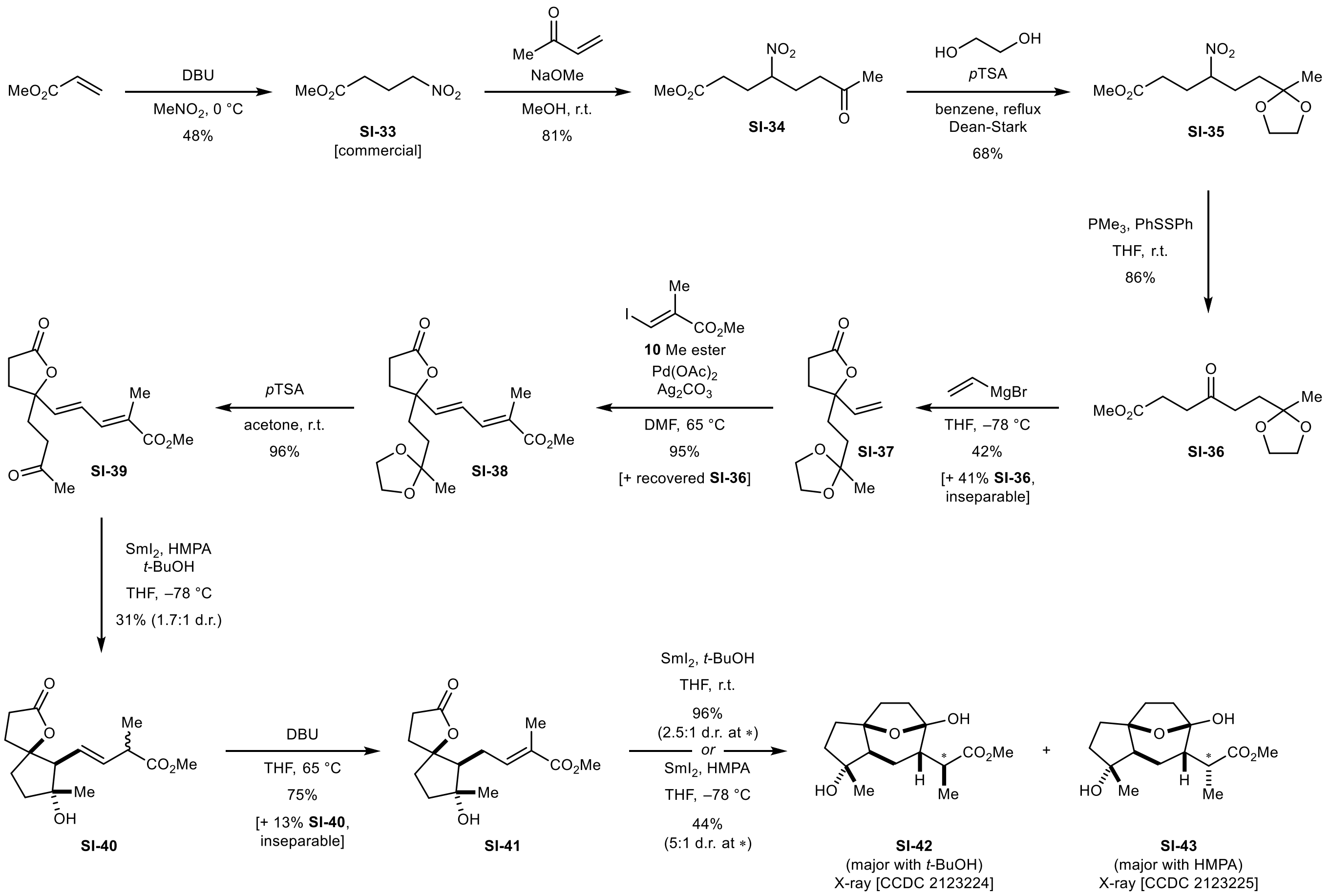

Figure S15. Summary of model studies[41]. Further experimental procedures and characterisation data are available upon request.

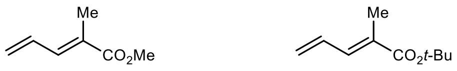

SI-44 (ref. 55) SI-45 (ref. 56)

Figure S16. Model substrates for CV measurements[55,56].

6. X-Ray Crystal structures

All data collections, crystal structure determinations and refinements were done by the X-ray crystallography service (Dr George Whitehead, Dr Inigo J. Vitorica-Yrezabal) at The University of Manchester.

Data collection

X-ray data was collected at a temperature of 100 K on a Rigaku FR-X DW rotating anode diffractometer using CuKα radiation, (λ = 1.54184 Å) with an AFC-11 RINC goniometer and a Rigaku Hypix 6000 HE photon counting detector. The diffractometer was equipped with an Oxford Cryosystems Cryostream 800 plus nitrogen flow gas system.

Crystal structure determinations and refinements

X-ray data were processed and reduced using CrysAlisPro suite of programs by. The crystal structures were solved and refined against all F2 values using the SHELX and Olex 2 suite of programs[57,58]. All the non-hydrogen atoms were refined anisotropically. Hydrogen atoms were placed in a calculated position refined using idealised geometries (riding model) and assigned fixed isotropic displacement parameters. Some carbon atoms were found disordered and modelled over two positions were possible. In such cases, C-C bond distances were restrained using FIX and SADI commands. The atomic displacement parameters (adp) of the disordered atoms have been restrained using RIGU command.

Data availability

Crystallographic data for **1**, **6**, **7** Me ester, **11** have been deposited in the Cambridge Crystallographic Data Centre, with deposition numbers CCDC 2125350 (**1**), 2123221 (**6**), 2123220 (**7** Me ester), 2123222 (**11**), and are available free of charge via www.ccdc.cam.ac.uk/data_request/cif.

Triol (–)-**11** – **CCDC 2123222**

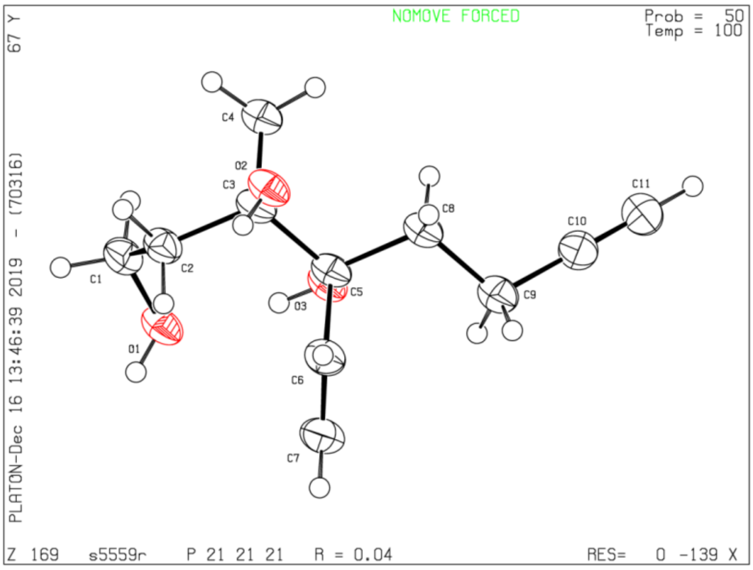

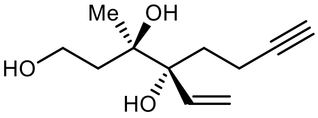

11

Table S18. Crystal data and structure refinement for (–)-**11**.

Empirical Formula	C11 H18 O3
Temperature/K	100
Crystal system	orthorhombic
Space group	P 21 21 21
a/Å	5.36272(15)
b/Å	12.0607(3)
c/Å	17.3174(6)
α/°	90
β/°	90
γ/°	90
Volume/Å^3	1120.06(6)
Z	4
F(000)	432
Radiation	Cu K\a
2Θ range for data collection/°	8.936 to 133.168
Index ranges	-6 ≤ h ≤ 4, -14 ≤ k ≤ 14, -19 ≤ l ≤ 20
Goodness-of-fit on F2	1.042
Final R indexes [I>=2σ (I)]	R1 = 0.0376 wR2 = 0.0947
Final R indexes [all data]	R1 = 0.0430 wR2 = 0.0981
Absolute structure parameter	0.07(18)

7 Me ester – **CCDC 2123220**

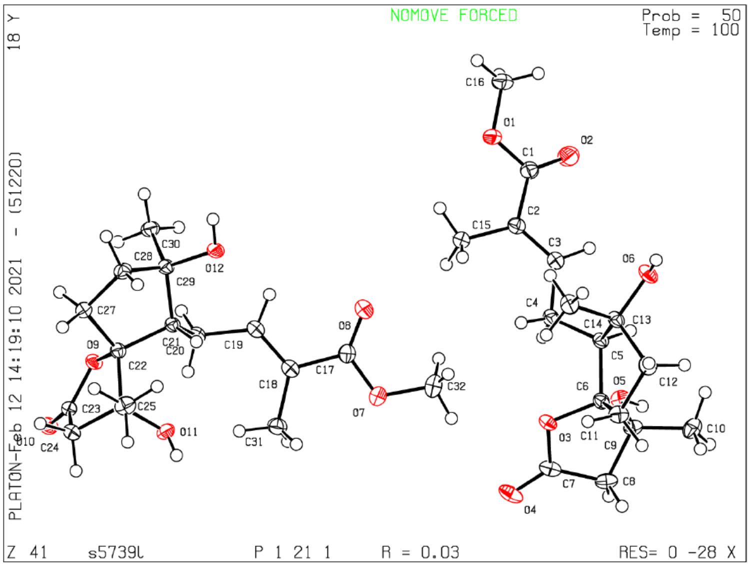

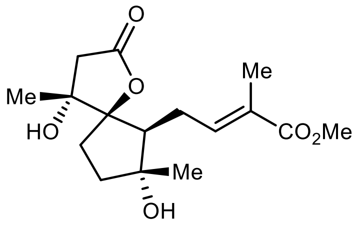

7 Me ester

Table S19. Crystal data and structure refinement for (+)-**7** Me ester.

Empirical Formula	C16 H24 O6
Temperature/K	100
Crystal system	monoclinic
Space group	P 1 21 1
a/Å	10.25367(5)
b/Å	9.37771(5)
c/Å	17.28846(10)
α/°	90
β/°	102.6834(6)
γ/°	90
Volume/Å^3	1621.824(15)
Z	4
F(000)	672
Radiation	Cu K\a
2Θ range for data collection/°	5.240 to 152.238
Index ranges	-12 ≤ h ≤ 12, -11 ≤ k ≤ 11, -21 ≤ l ≤ 21
Goodness-of-fit on F2	1.053
Final R indexes [I>=2σ (I)]	R1 = 0.0254 wR2 = 0.0664
Final R indexes [all data]	R1 = 0.0255 wR2 = 0.0664
Absolute structure parameter	0.00(2)

Lactone **6** – **CCDC 2123221**

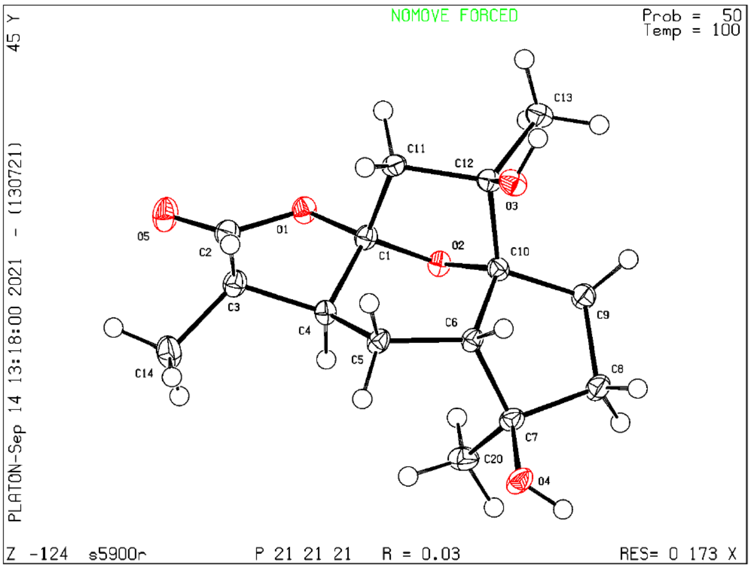

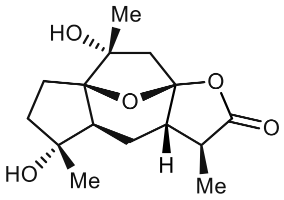

6

Table S20. Crystal data and structure refinement for lactone **6**.

Empirical Formula	C15 H22 O5
Temperature/K	100
Crystal system	orthorhombic
Space group	P 21 21 21
a/Å	7.30554(12)
b/Å	10.57545(18)
c/Å	18.1068(3)
α/°	90
β/°	90
γ/°	90
Volume/Å^3	1398.92(4)
Z	4
F(000)	608
Radiation	Cu K\a
2Θ range for data collection/°	9.686 to 151.158
Index ranges	-9 ≤ h ≤ 9, -11 ≤ k ≤ 13, -22 ≤ l ≤ 16
Goodness-of-fit on F2	1.051
Final R indexes [I>=2σ (I)]	R1 = 0.0284 wR2 = 0.0733
Final R indexes [all data]	R1 = 0.0292 wR2 = 0.0738
Absolute structure parameter	-0.01(5)

(–)-phaeocaulisin A (**1**) – **CCDC 2125350**

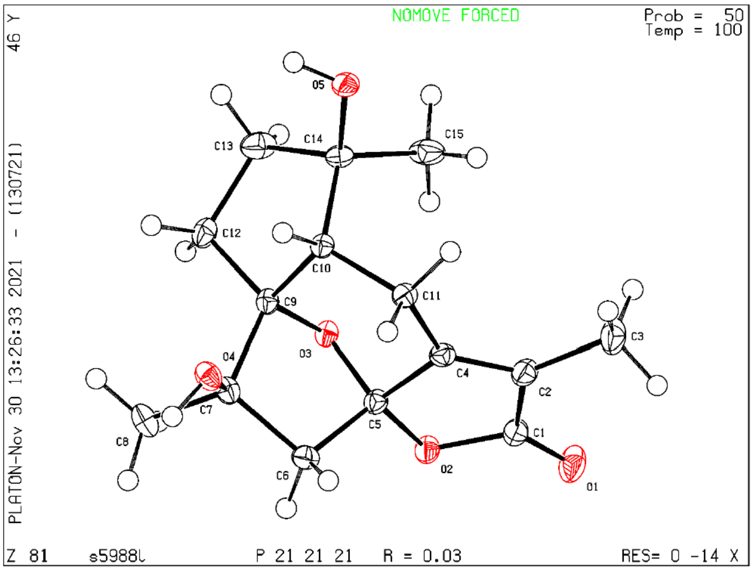

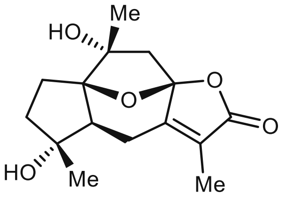

(–)-phaeocaulisin A (**1**)

Table S21. Crystal data and structure refinement for lactone (–)-phaeocaulisin A (**1**).

Empirical Formula	C15 H20 O5
Temperature/K	100
Crystal system	orthorhombic
Space group	P 21 21 21
a/Å	7.2314(2)
b/Å	10.6297(3)
c/Å	18.0371(5)
α/°	90
β/°	90
γ/°	90
Volume/Å^3	1386.47(7)
Z	4
F(000)	600
Radiation	Cu K\a
2Θ range for data collection/°	9.658 to 152.522
Index ranges	-8 ≤ h ≤ 9, -9 ≤ k ≤ 12, -22 ≤ l ≤ 21
Goodness-of-fit on F2	1.063
Final R indexes [I>=2σ (I)]	R1 = 0.0285 wR2 = 0.0711
Final R indexes [all data]	R1 = 0.0315 wR2 = 0.0723
Absolute structure parameter	0.00(8)

7. Cyclic voltammetry (CV) studies

Cyclic voltammetry measurements were conducted on an EmStat4s (PalmSens) potentiostat using a three electrode cell configuration (Fig. S17). A glassy carbon working electrode (red) was employed alongside a platinum wire counter electrode (black) and a Ag/AgCl reference electrode (blue). All the solutions were degassed by bubbling dry N_2 prior to measurements for 5 min (green needle). 5 mM solutions of the desired compounds were freshly prepared in dry THF along with 0.1 M of *n*-Bu_4NPF_6 as supporting electrolyte and were examined at a scan rate of 0.1 V s^{-1}.

Irreversible waves were obtained in all cases; therefore, the potentials reported are half-peak potentials ($E_{p/2}$).

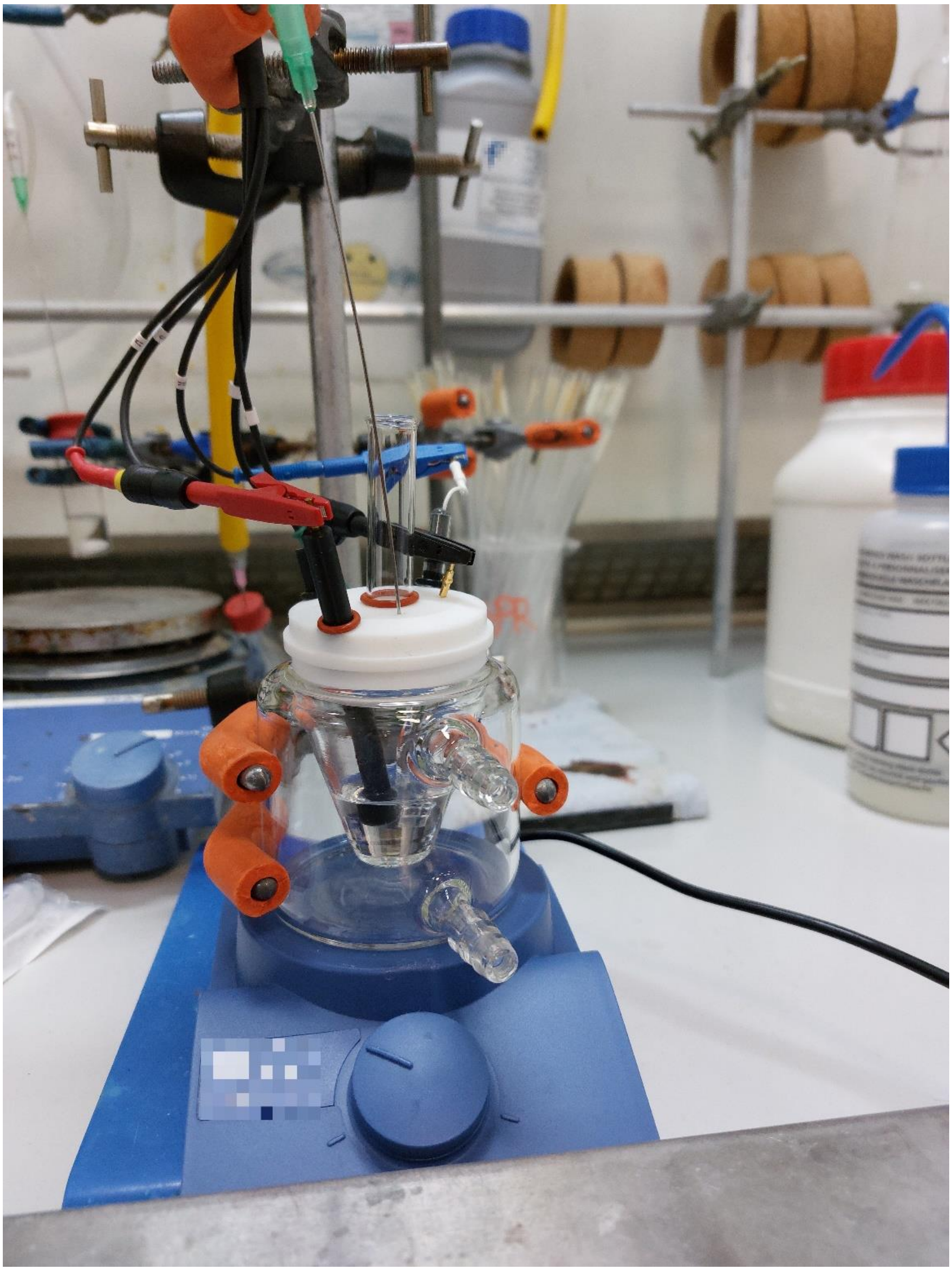

Figure S17. Our cyclic voltammetry measurements setup.

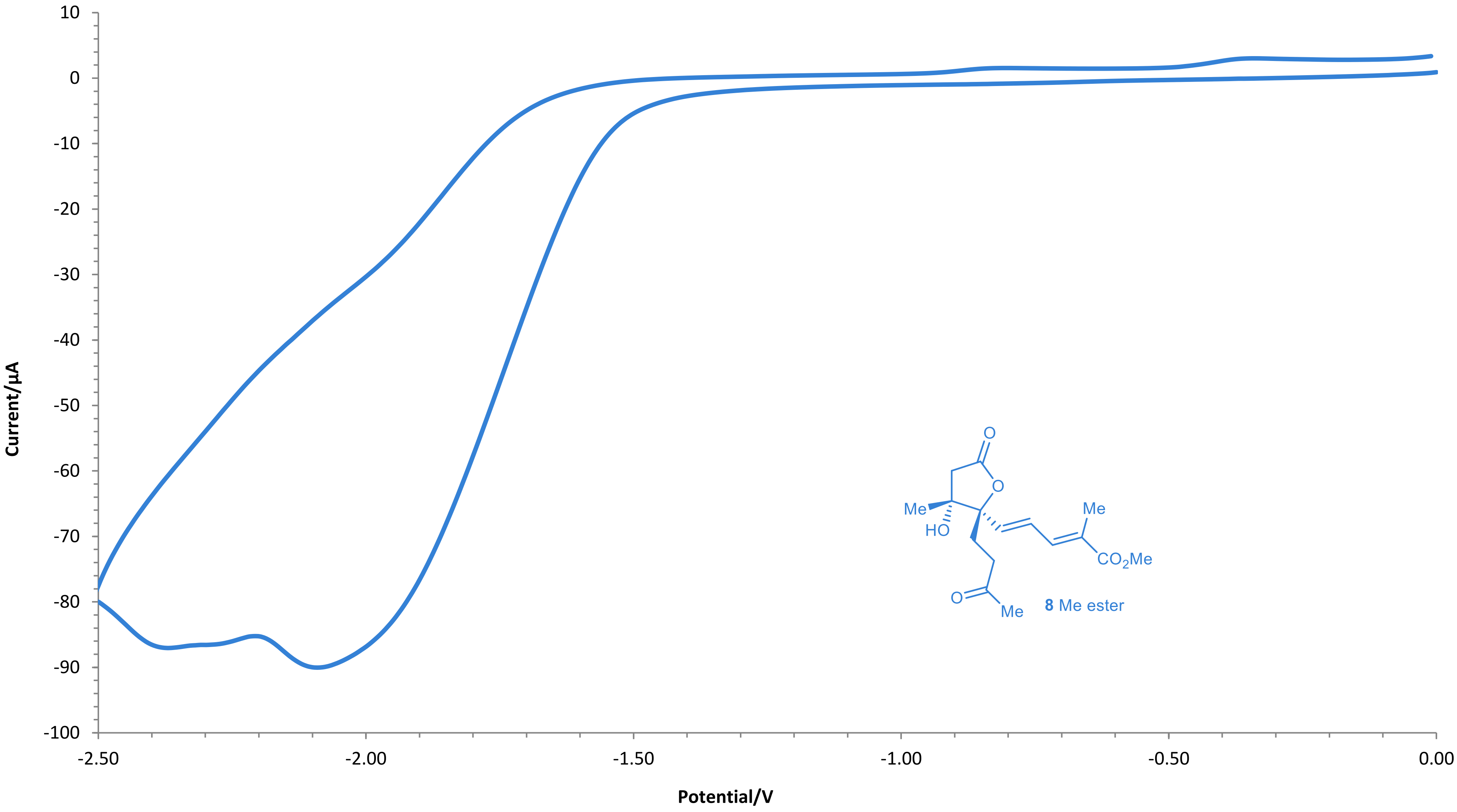

Figure S18. Cyclic voltammogram of **8** Me ester. $E_{p/2}$ (**8** Me ester/**8** Me ester$^{\bullet -}$) = –1.75 V *vs*. Ag/AgCl in THF.

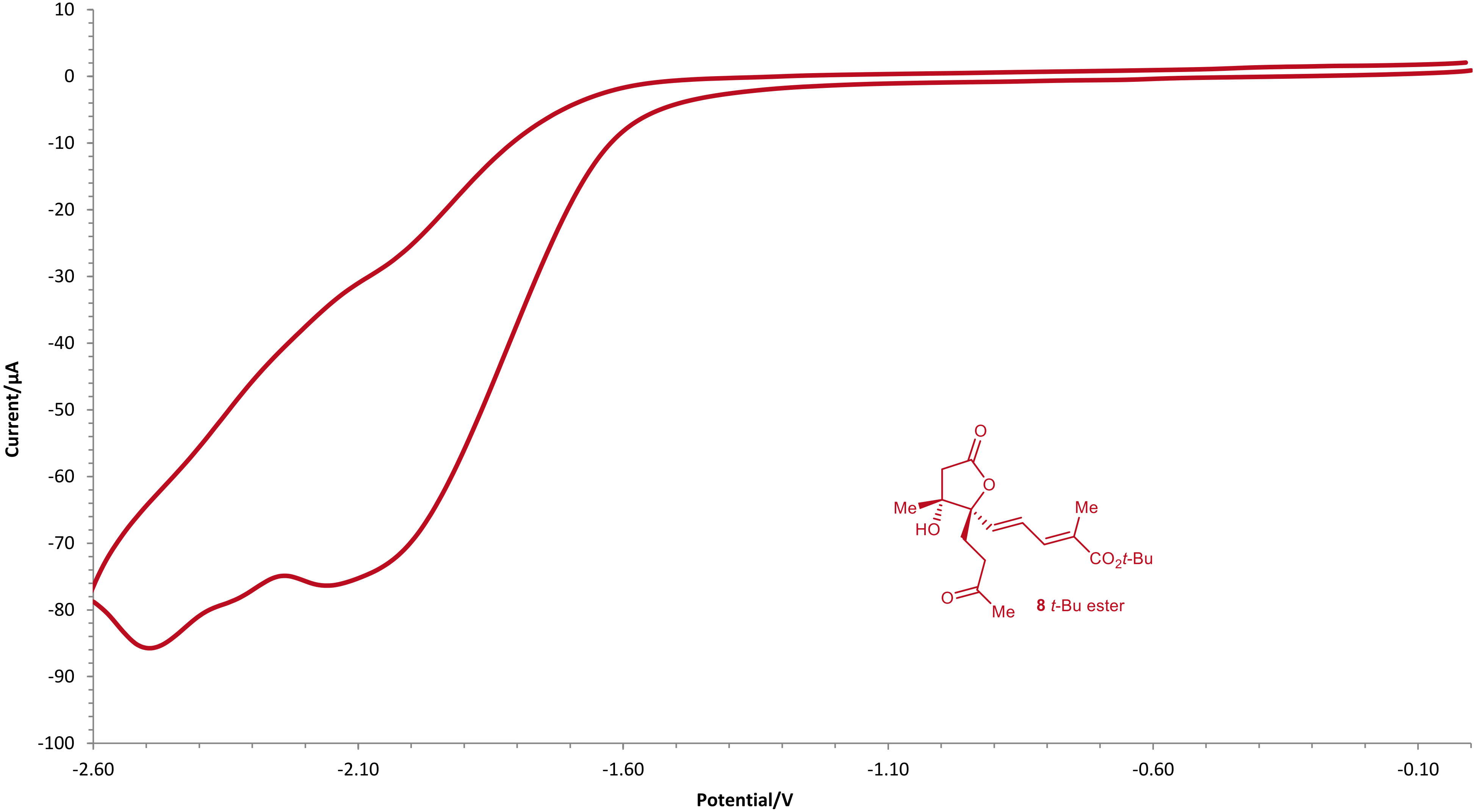

Figure S19. Cyclic voltammogram of **8** *t*-Bu ester. $E_{p/2}$ (**8** *t*-Bu ester/**8** *t*-Bu ester$^{\bullet-}$) = –1.81 V *vs*. Ag/AgCl in THF.

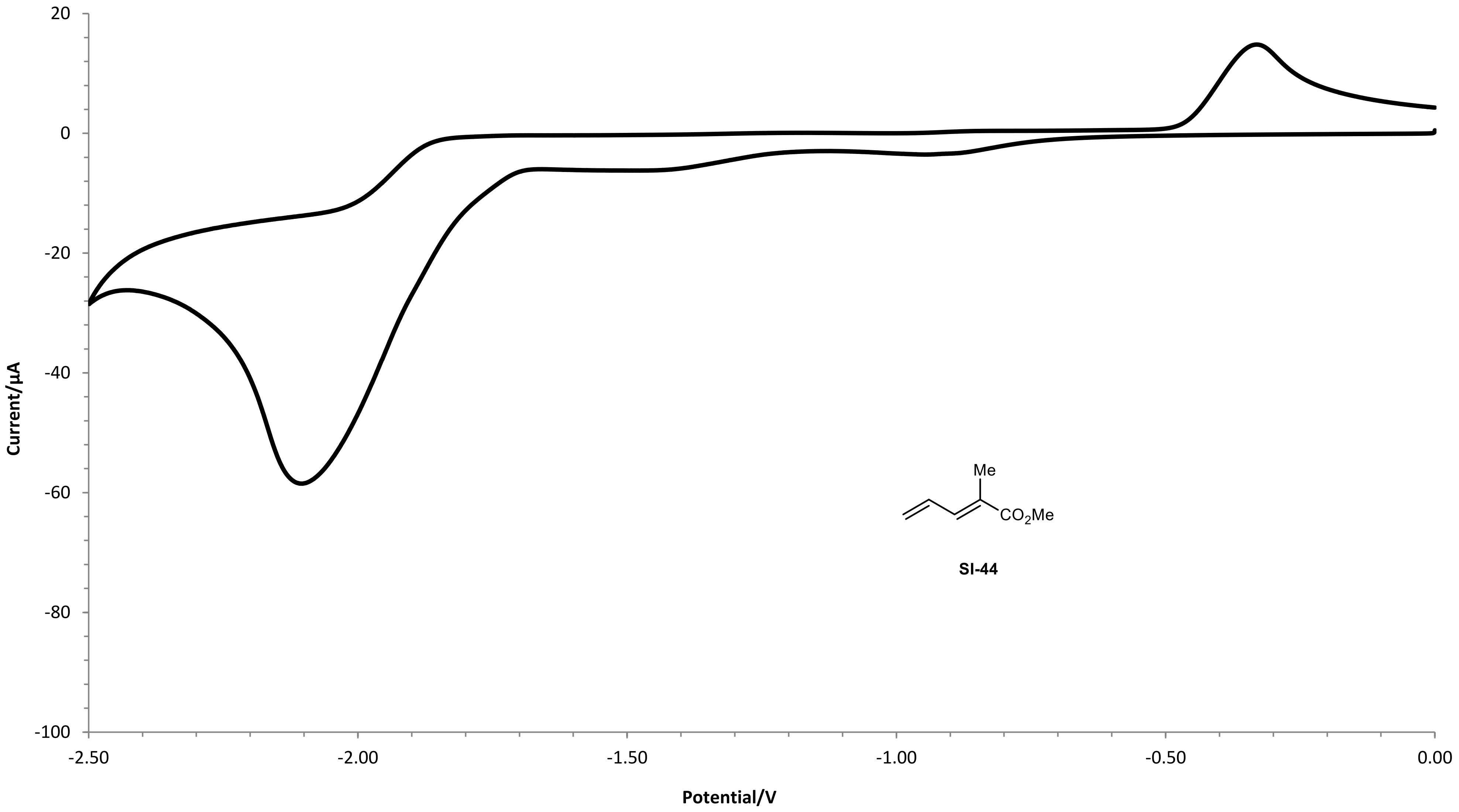

Figure S20. Cyclic voltammogram of **SI-44**. $E_{p/2}$ (**SI-44**/**SI-44**$^{\bullet-}$) = –1.91 V *vs*. Ag/AgCl in THF.

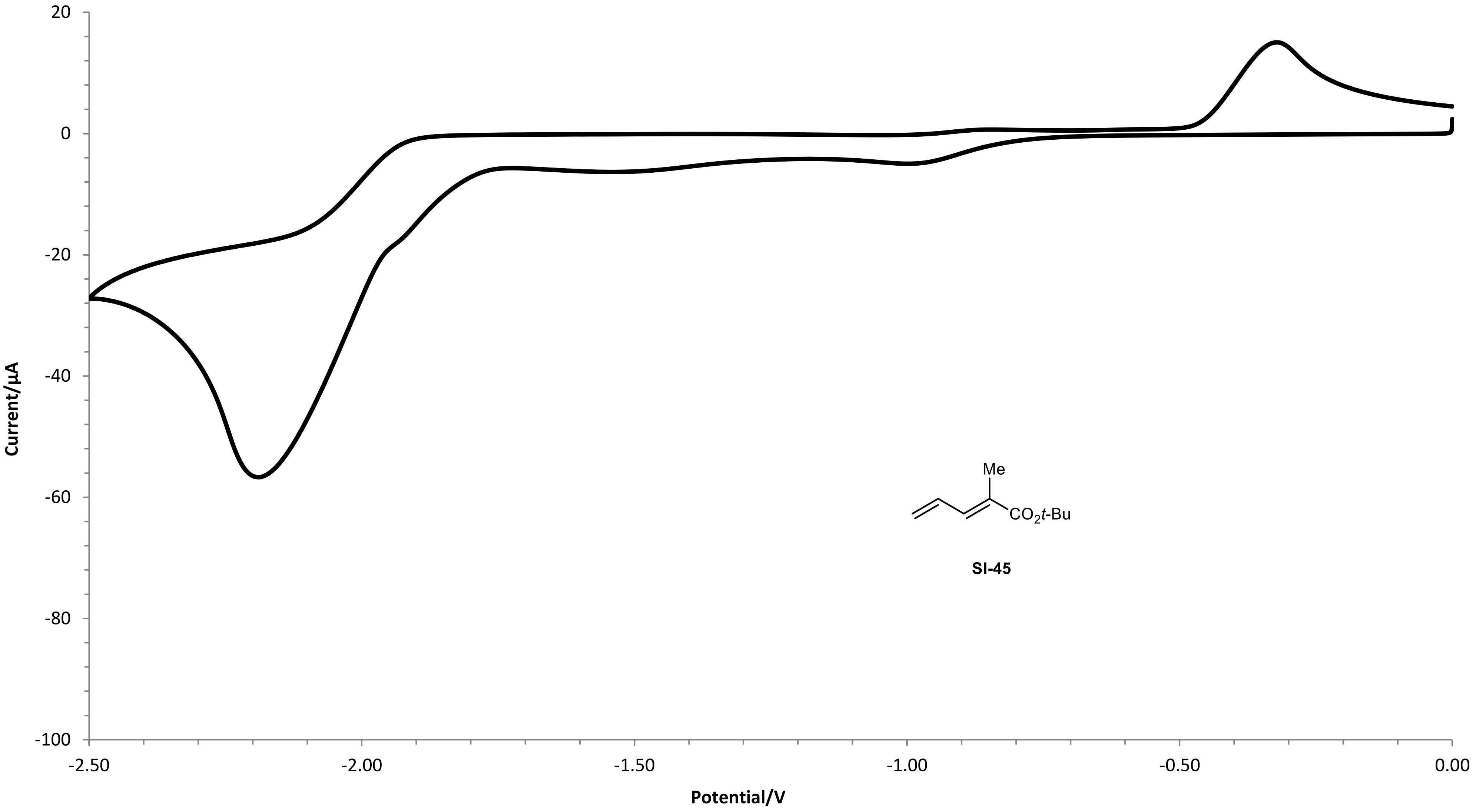

Figure S21. Cyclic voltammogram of **SI-45**. $E_{p/2}$ (**SI-45**/**SI-45**$^{•–}$) = –2.00 V *vs*. Ag/AgCl in THF.

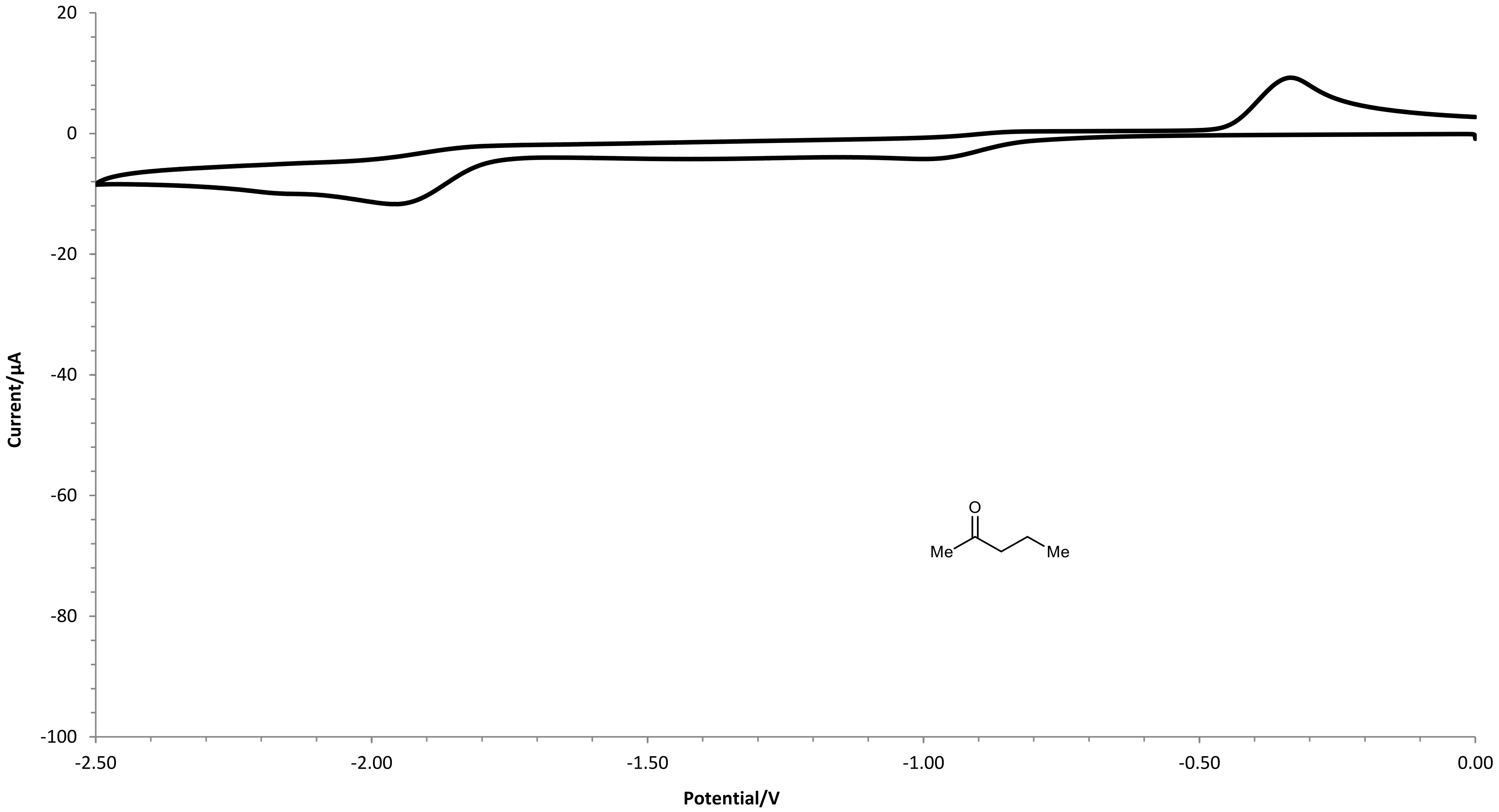

Figure S22. Cyclic voltammogram of 2-pentanone.

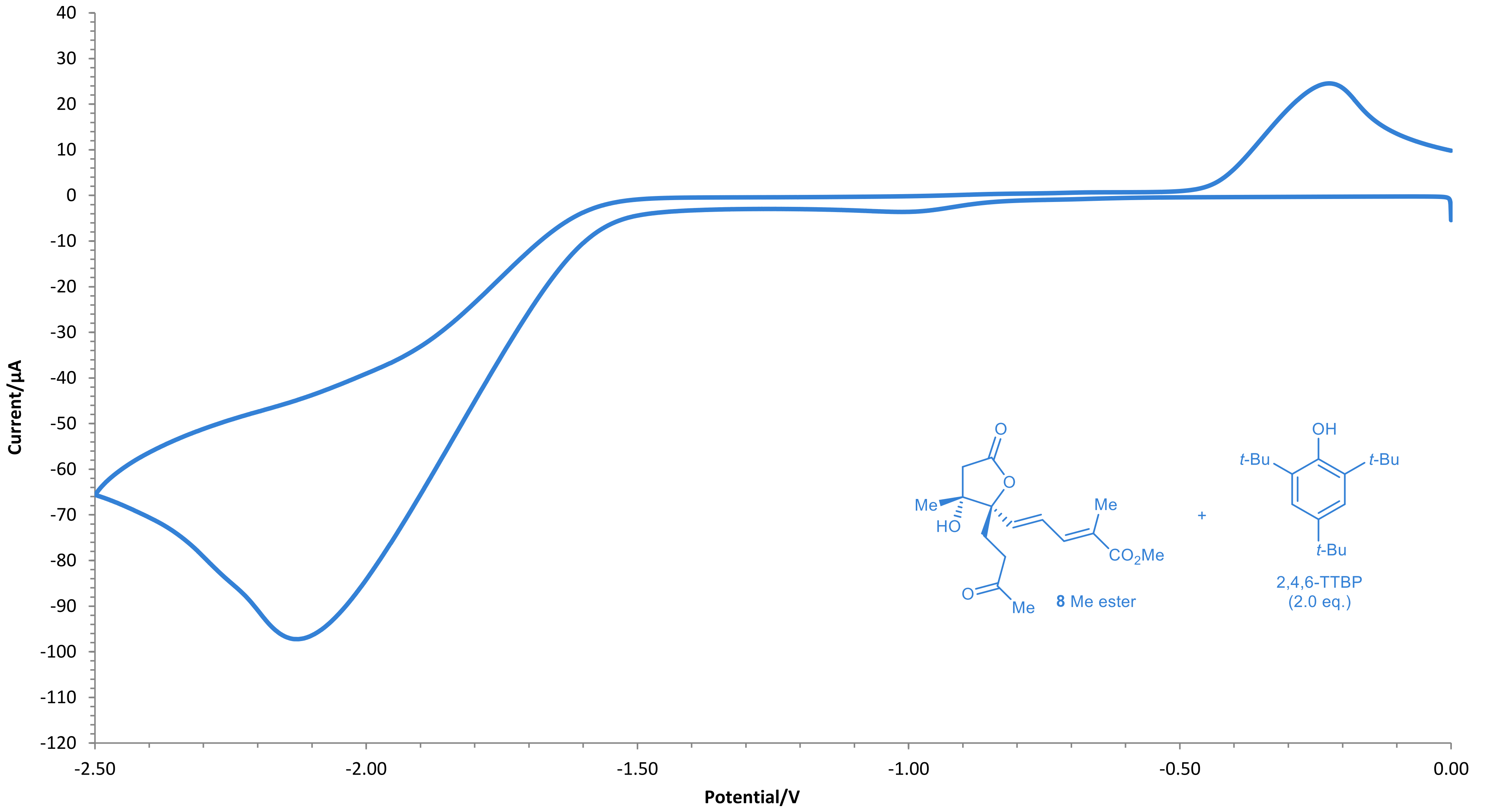

Figure S23. Cyclic voltammogram of **8** Me ester in the presence of 2 equiv. of 2,4,6-TTBP. $E_{p/2}$ (**8** Me ester/**8** Me ester$^{\bullet-}$) = –1.82 V *vs*. Ag/AgCl in THF (2 equiv. of 2,4,6-TTBP was added).

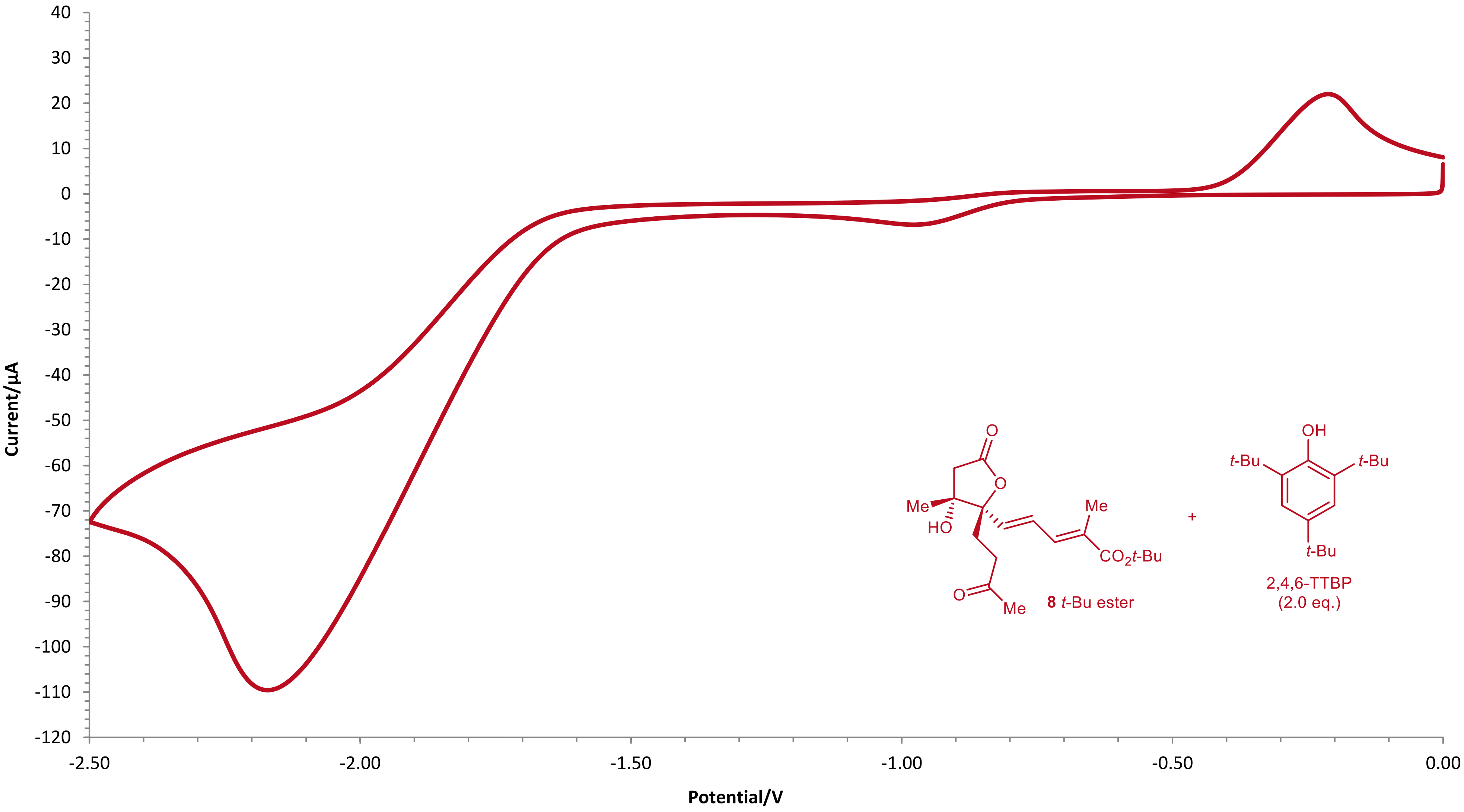

Figure S24. Cyclic voltammogram of **8** *t*-Bu ester in the presence of 2 equiv. of 2,4,6-TTBP. $E_{p/2}$ (**8** *t*-Bu ester/**8** *t*-Bu ester$^{\bullet-}$) = –1.87 V *vs*. Ag/AgCl in THF (2 equiv. of 2,4,6-TTBP was added).

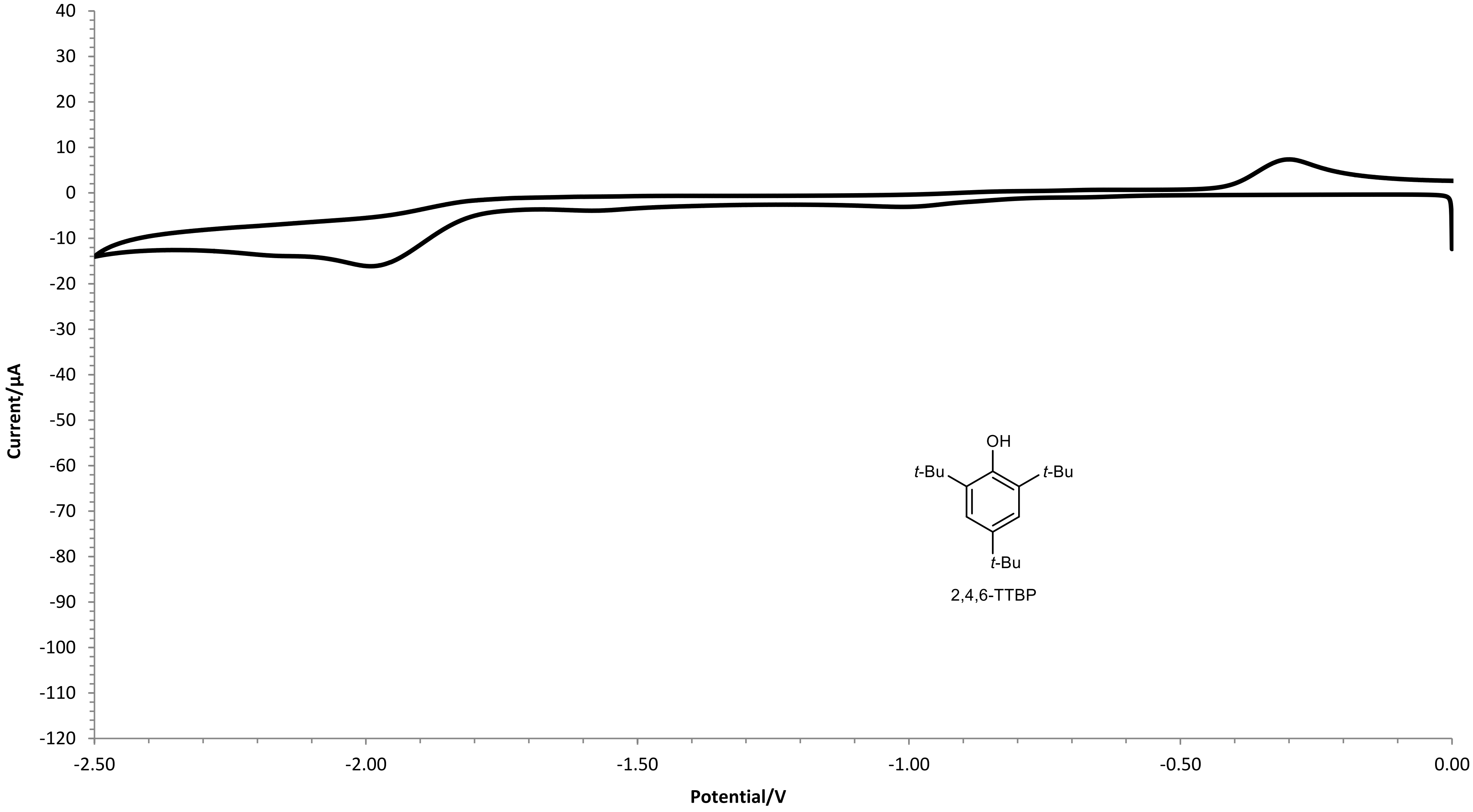

Figure S25. Cyclic voltammogram of 2,4,6-TTBP in THF.

Interpretation of cyclic voltammetry measurements

Table S22. Summary of half-peak potentials of **8** Me ester and **8** *t*-Bu ester in the absence and presence of 2.0 equiv. of 2,4,6-TTBP in THF. The $E_{p/2}$ values reported are *vs*. Ag/AgCl in THF.

$E_{p/2}$	**8** Me ester/**8** Me ester$^{•-}$	**8** *t*-Bu ester/**8** *t*-Bu ester$^{•-}$	$\lvert \Delta E_{p/2} \rvert$
0.0 equiv. 2,4,6-TTBP	–1.75 V	–1.81 V	0.06 V
2.0 equiv. 2,4,6-TTBP	–1.82 V	–1.87 V	0.05 V

Discussion: the half-peak potentials shift to more negative values upon addition of 2.0 equiv. of 2,4,6-TTBP. However, the difference between the half-peak potentials of **8** Me ester and **8** *t*-Bu ester remains the same.

Table S23. Summary of half-peak potentials of **8** Me ester, **8** *t*-Bu ester, and model substrates – **SI-44** and **SI-45** – (Fig. S16) in THF. The $E_{p/2}$ values reported are *vs*. Ag/AgCl in THF.

**8** Me ester/**8** Me ester$^{•-}$	**SI-44**/**SI-44**$^{•-}$	**8** *t*-Bu ester/**8** *t*-Bu ester$^{•-}$	**SI-45**/**SI-45**$^{•-}$
–1.75 V	–1.91 V	–1.81 V	–2.00 V

Discussion: Comparing the half-peak potentials and cyclic voltammograms of the model substrates (**SI-44** and **SI-45**), ketone model 2-pentanone, **8** Me ester, and **8** *t*-Bu ester we concluded that the most reducible site in **8** Me ester and **8** *t*-Bu ester is the conjugated diene system. This supports the fact that in the key cyclisation step SmI_2 kinetically disfavours the reduction of the diene system upon changing the ester for Me to *t*-Bu, even though it remains the most reducible functionality (i.e. more reducible than the ketone moiety present).

8. Circular dichroism (CD) spectrum of (–)-phaeocaulisin A (1)

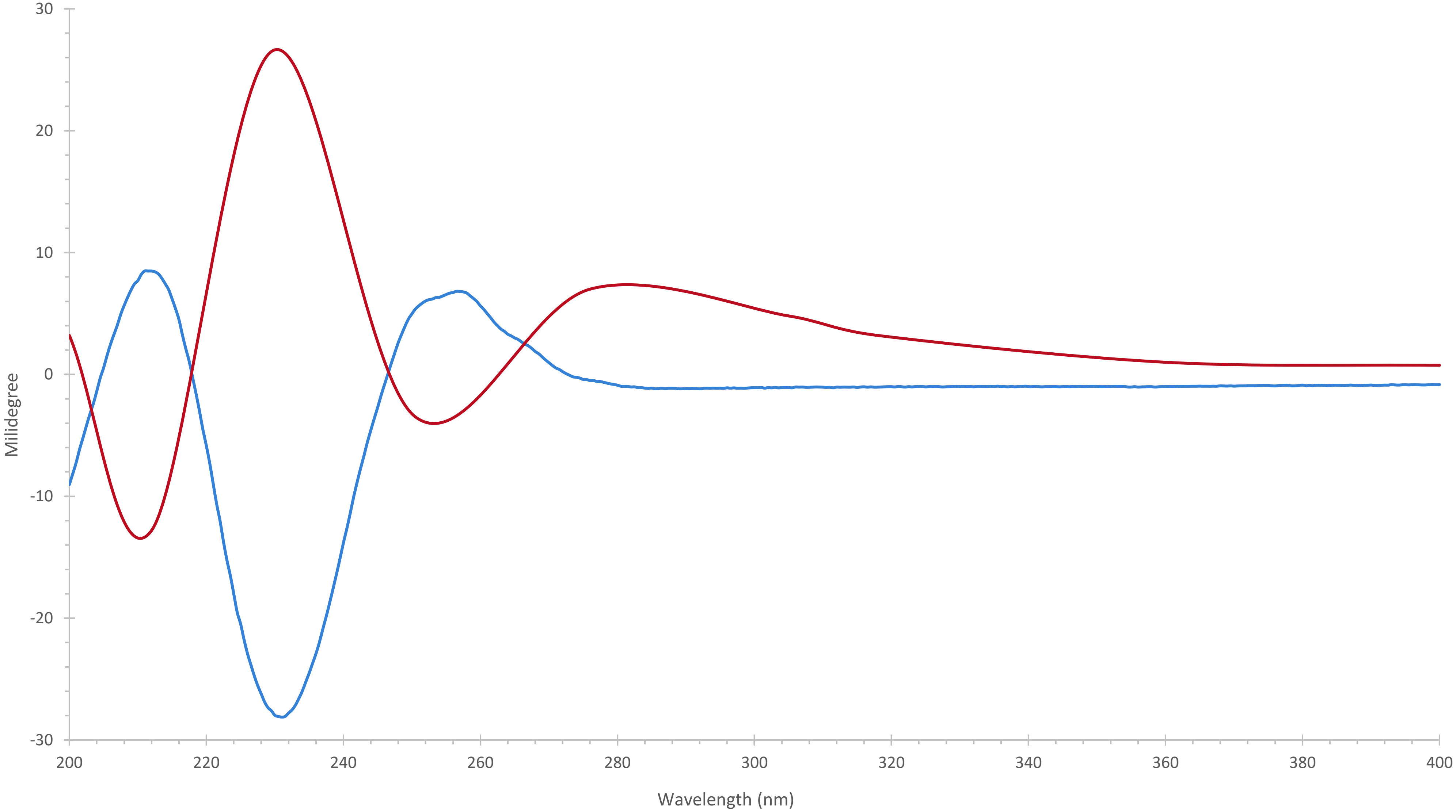

Figure S26. Circular dichroism (CD) spectrum of (–)-phaeocaulisin A (**1**, blue) which is the opposite of that of the isolated sample, (+)-phaeocaulisin A (red, the parameters were manually adjusted so the curve fits that of the isolated sample's)[4].

9. ^{1}H and ^{13}C NMR spectra

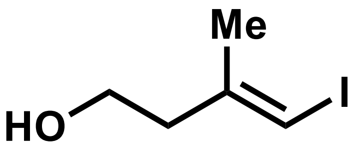

SI-1

^{1}H NMR, 400 MHz, $CDCl_3$

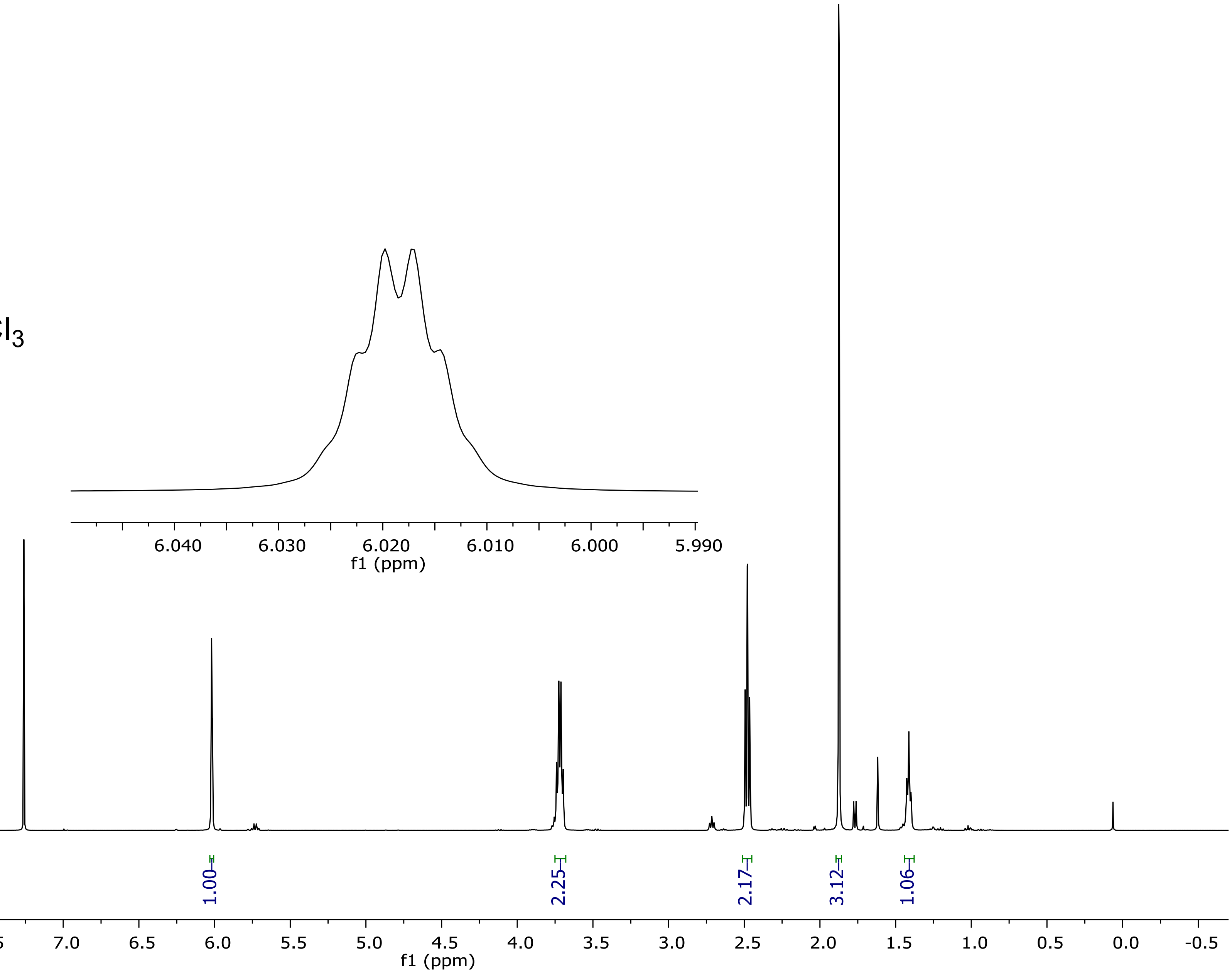

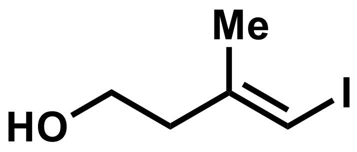

SI-1

^{13}C NMR, 101 MHz, $CDCl_3$

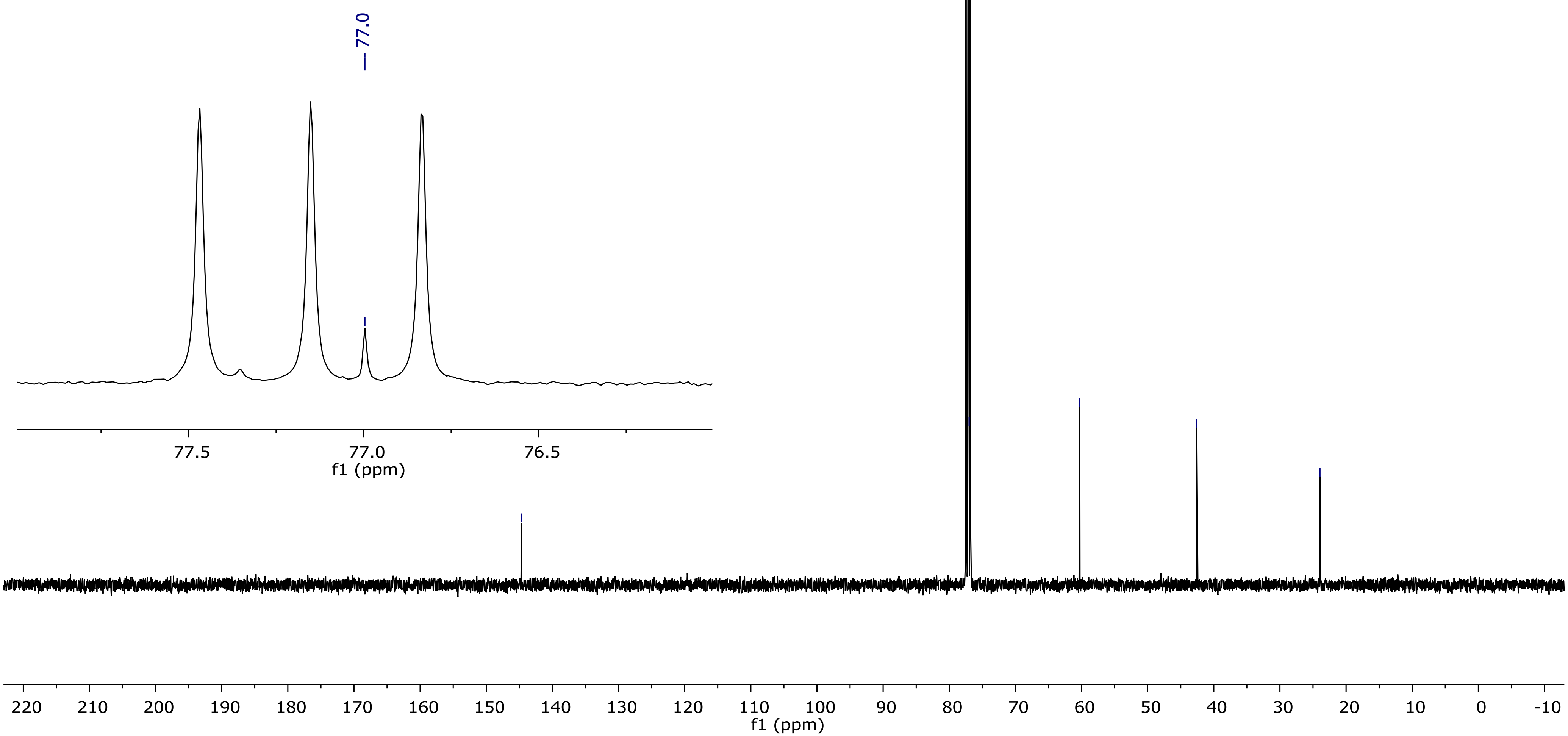

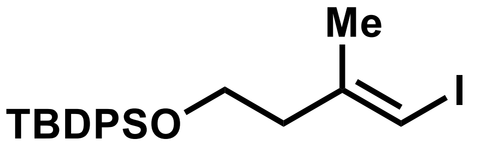

14

^{1}H NMR, 400 MHz, $CDCl_3$

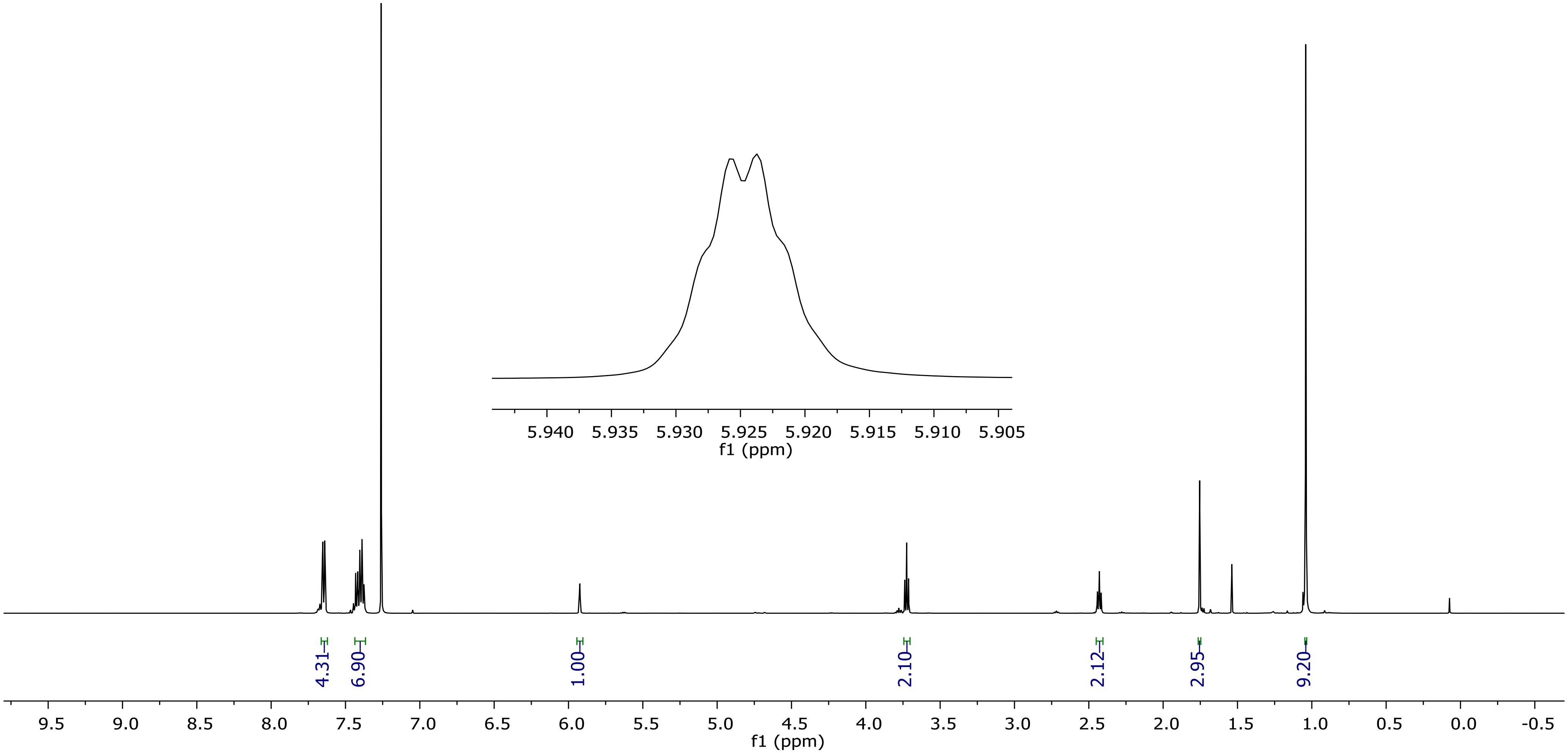

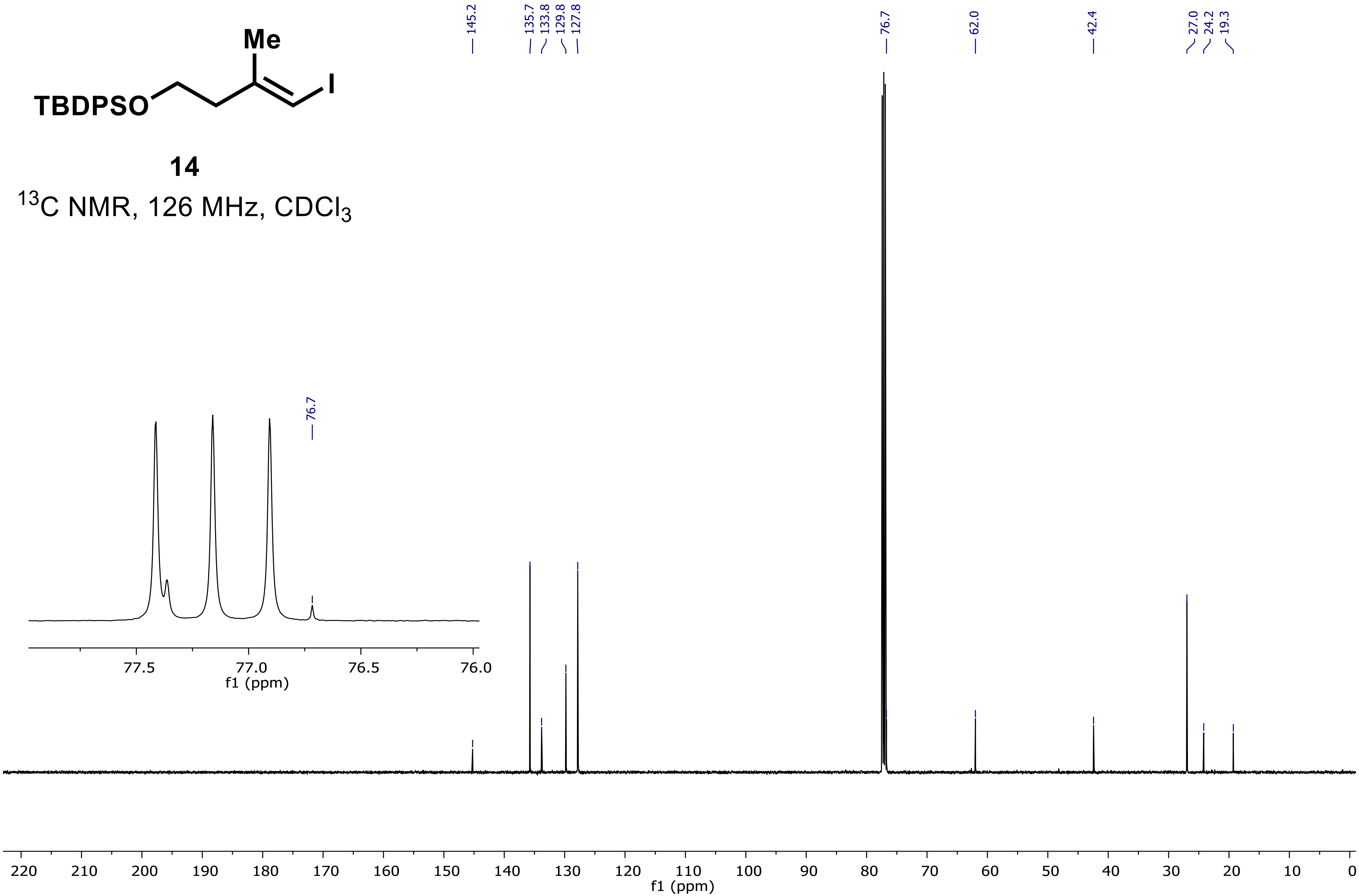
Me
TBDPSO
I
14
^{13}C NMR, 126 MHz, $CDCl_3$
145.2
135.7
133.8
129.8
127.8
76.7
62.0
42.4
27.0
24.2
19.3
77.5
77.0
76.5
76.0
f1 (ppm)
220 210 200 190 180 170 160 150 140 130 120 110 100 90 80 70 60 50 40 30 20 10 0
f1 (ppm)

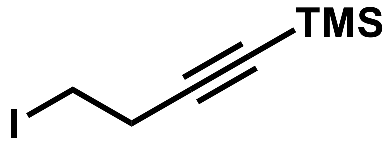

SI-6

^{1}H NMR, 400 MHz, $CDCl_3$

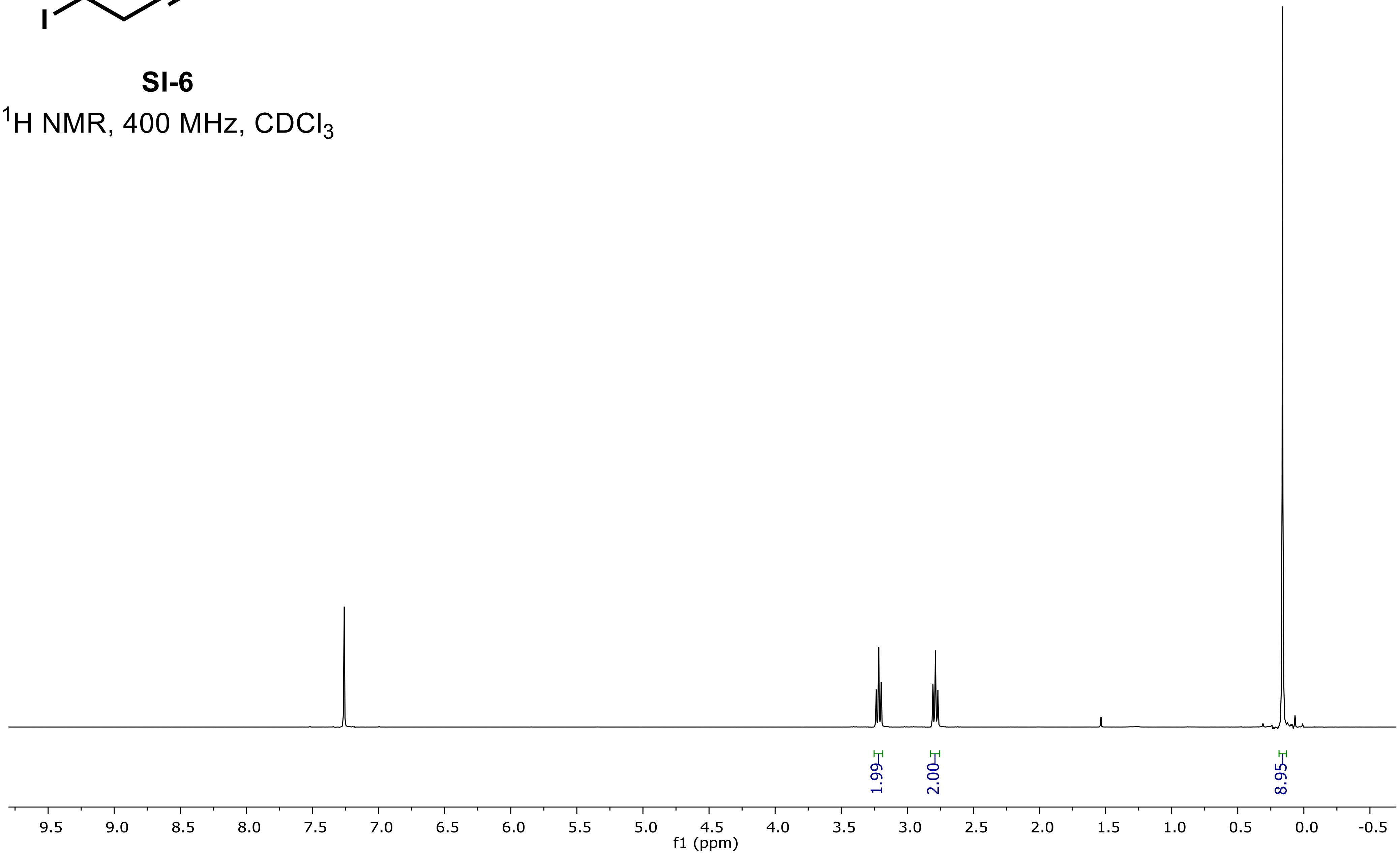

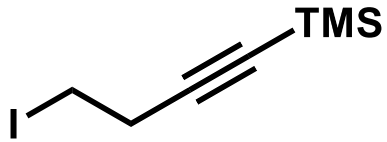

SI-6

^{13}C NMR, 101 MHz, $CDCl_3$

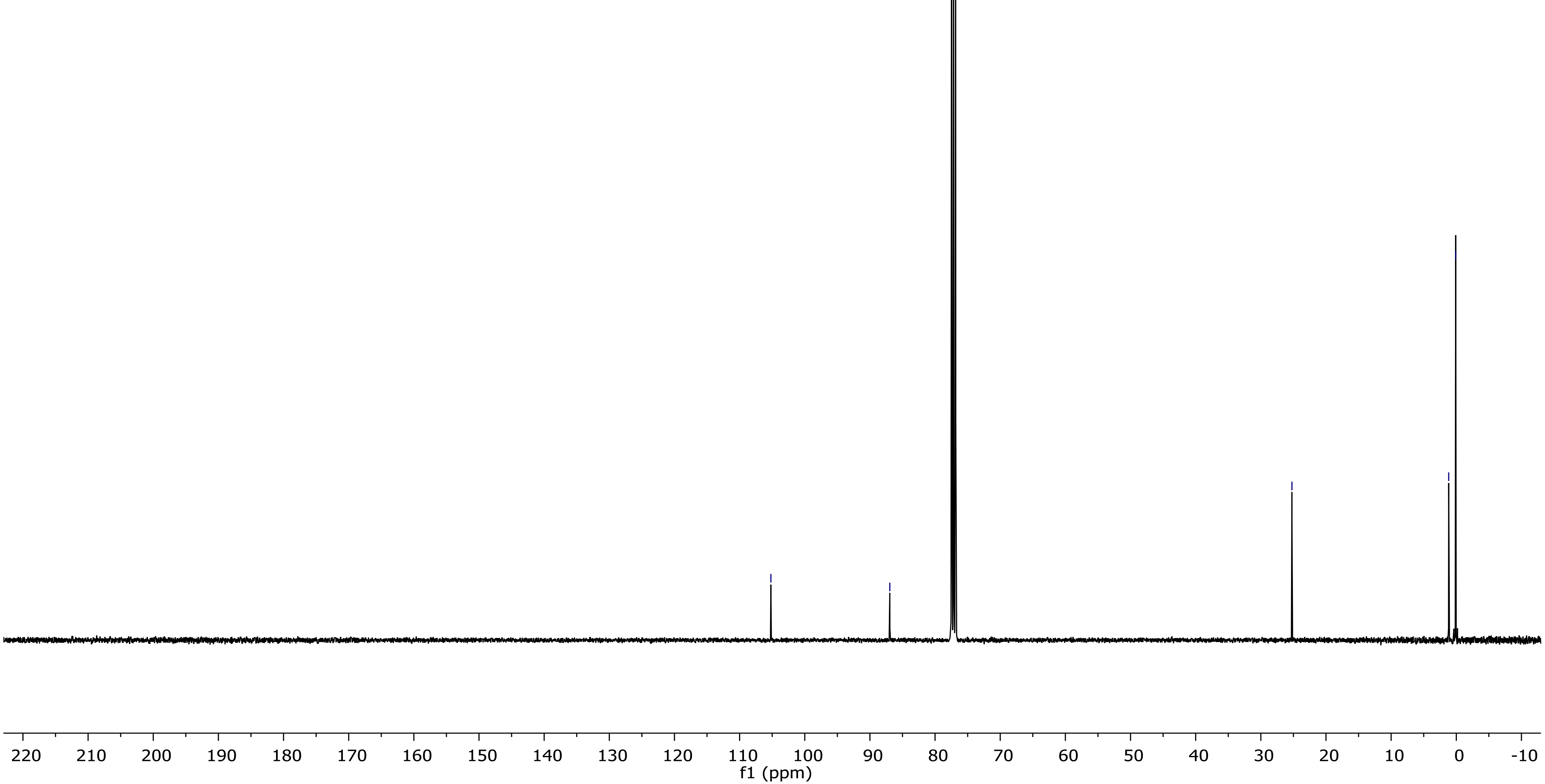

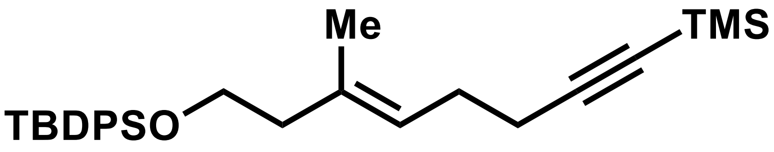

16

^{1}H NMR, 500 MHz, $CDCl_3$

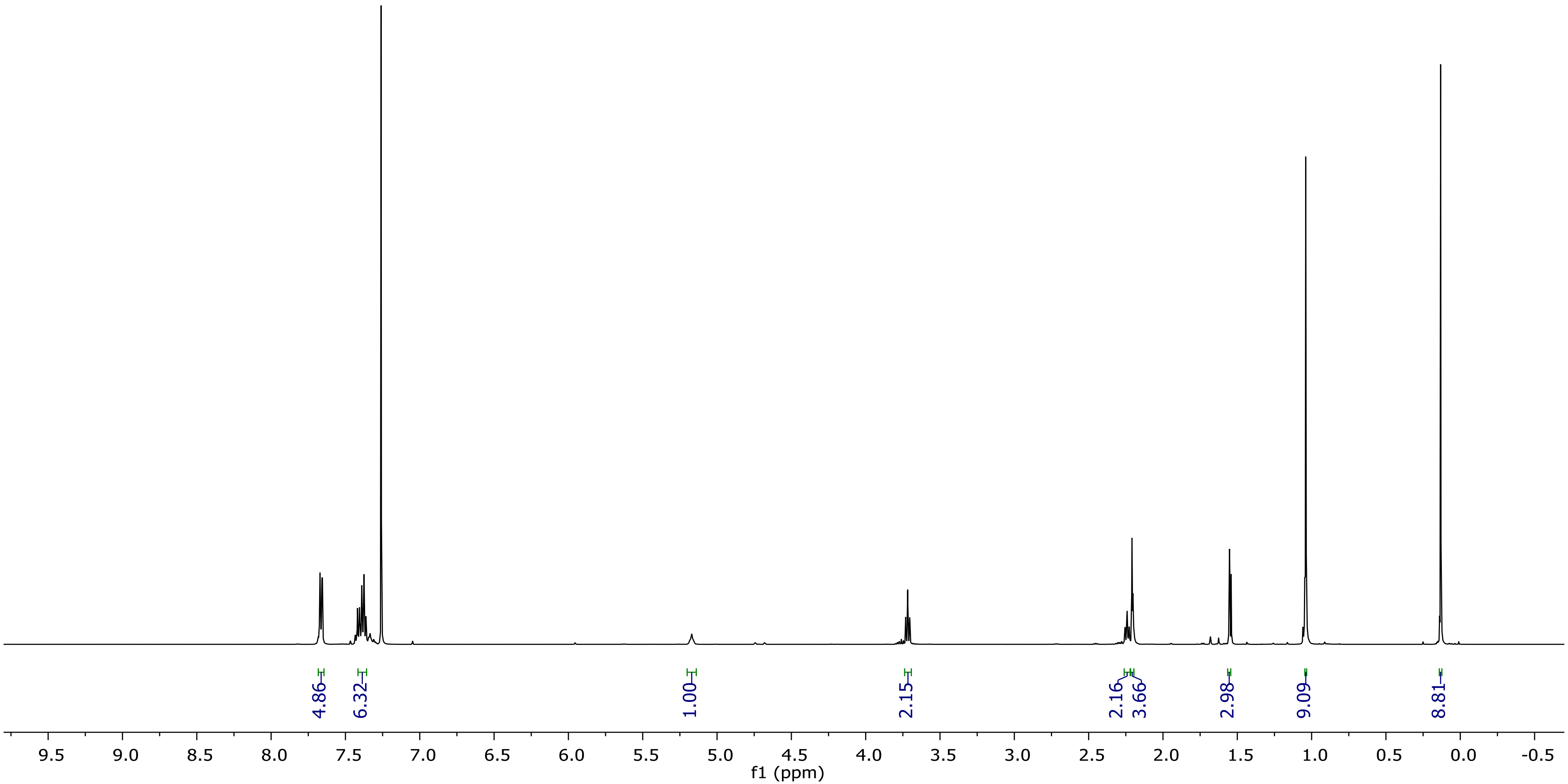

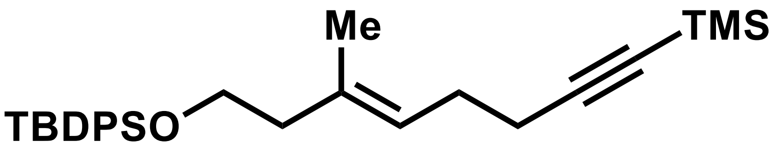

16

^{13}C NMR, 126 MHz, $CDCl_3$

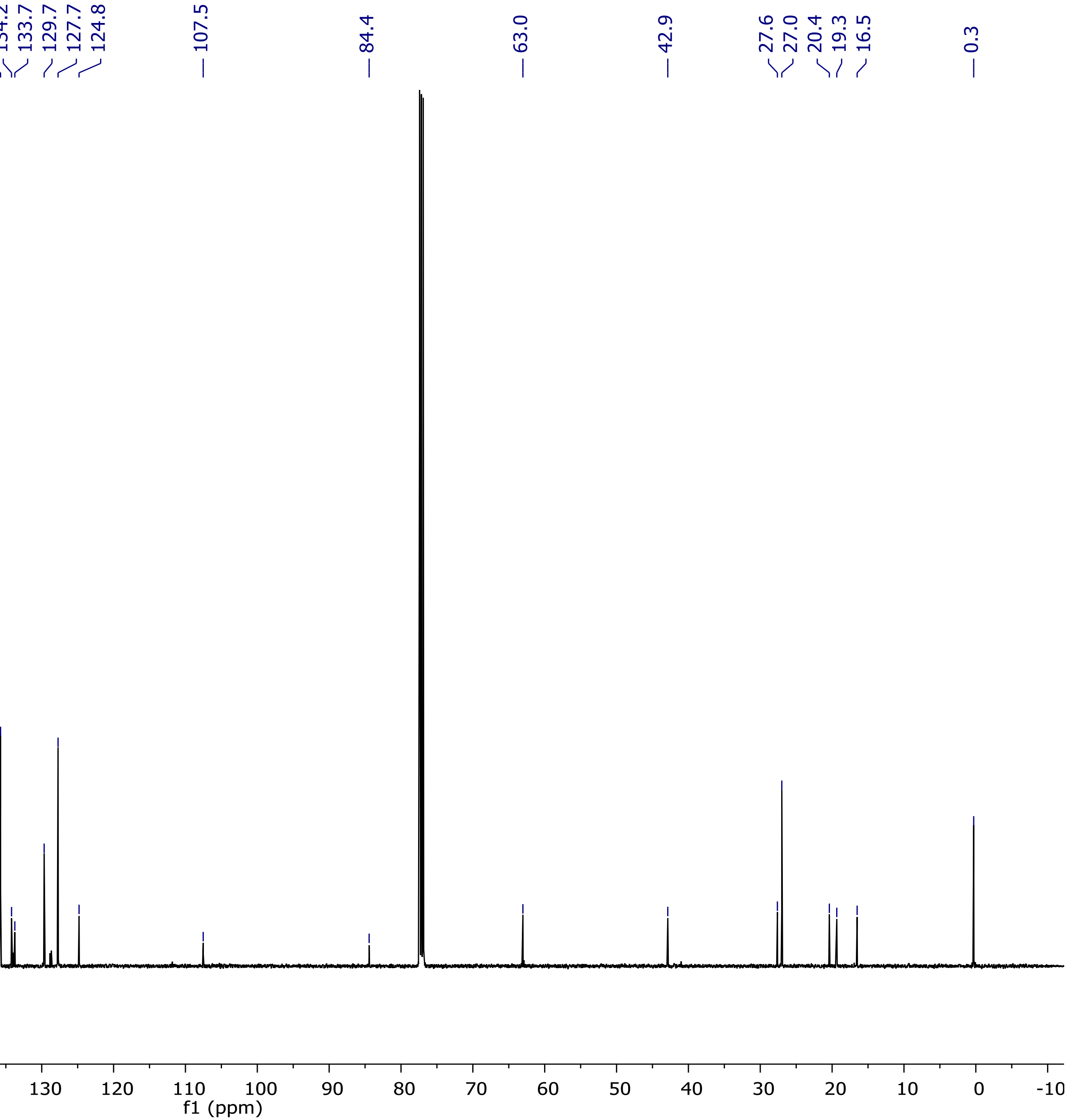

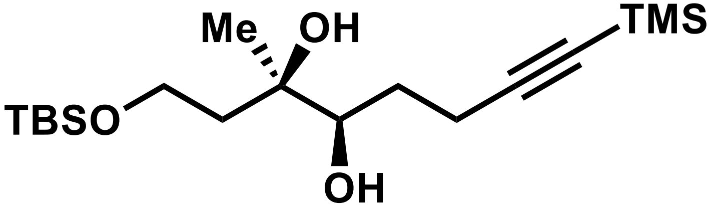

SI-8

^{1}H NMR, 400 MHz, $CDCl_3$

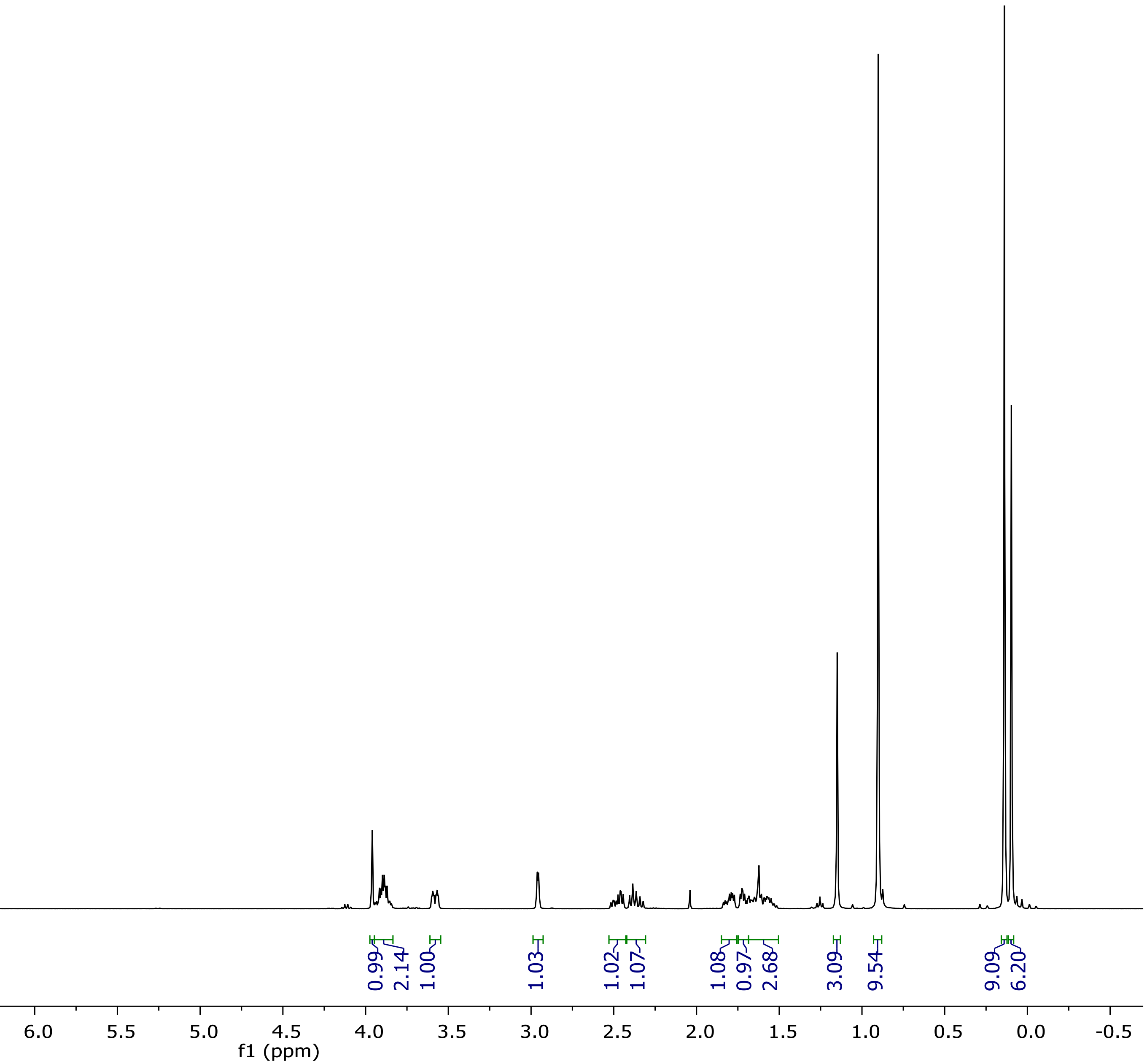

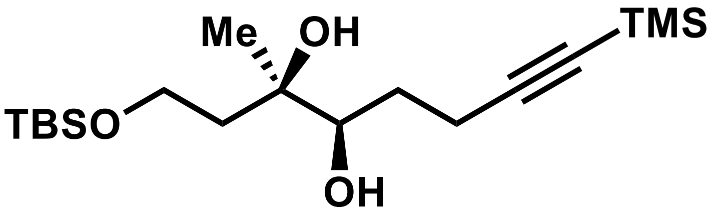

SI-8

^{13}C NMR, 101 MHz, $CDCl_3$

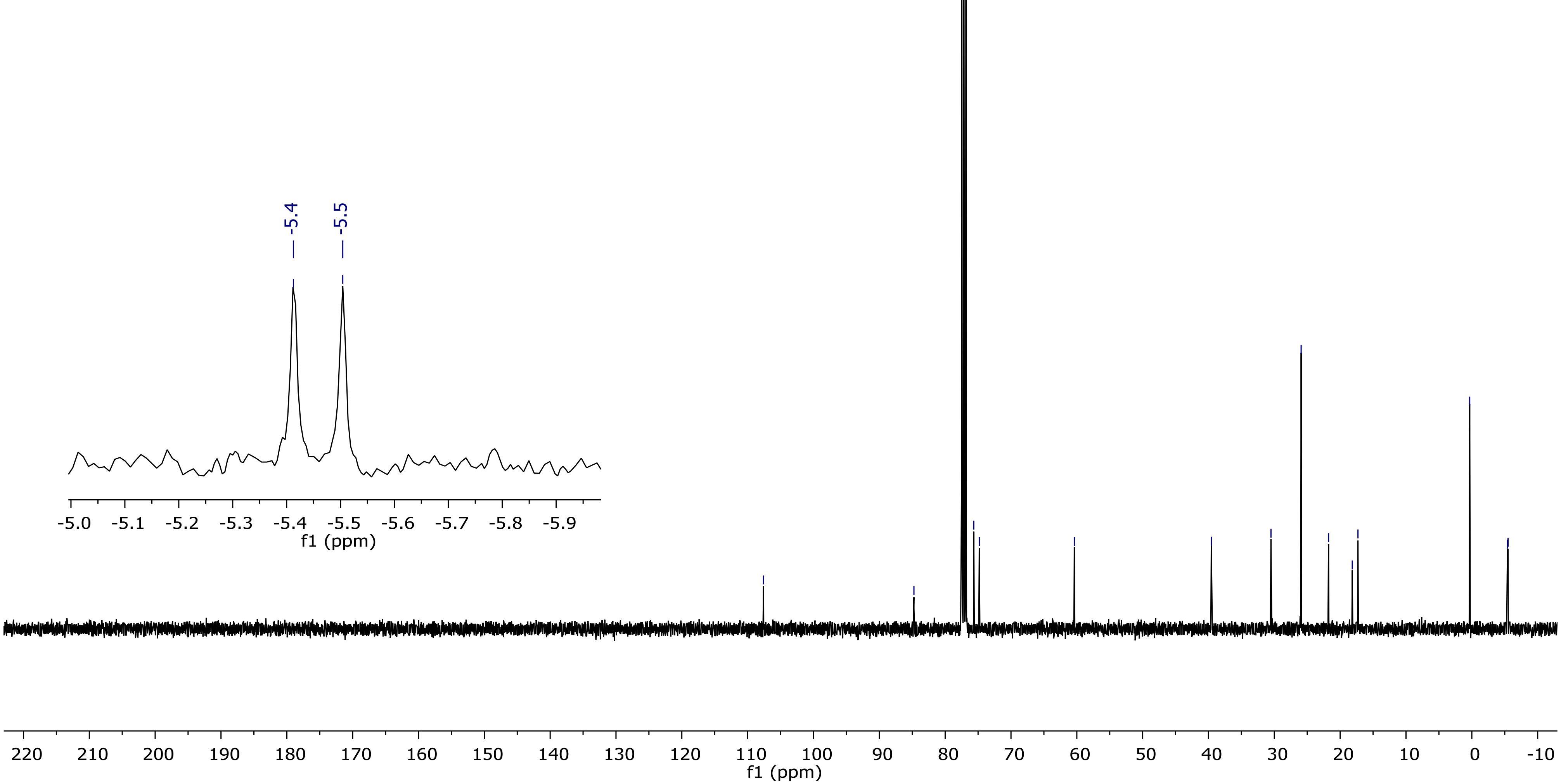

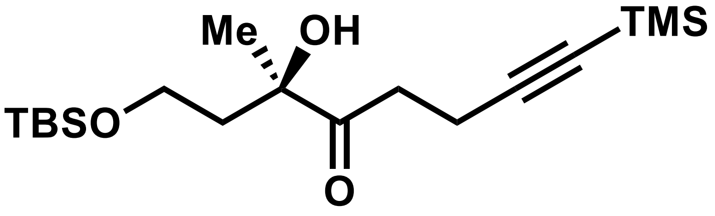

SI-9

^1H NMR, 400 MHz, $CDCl_3$

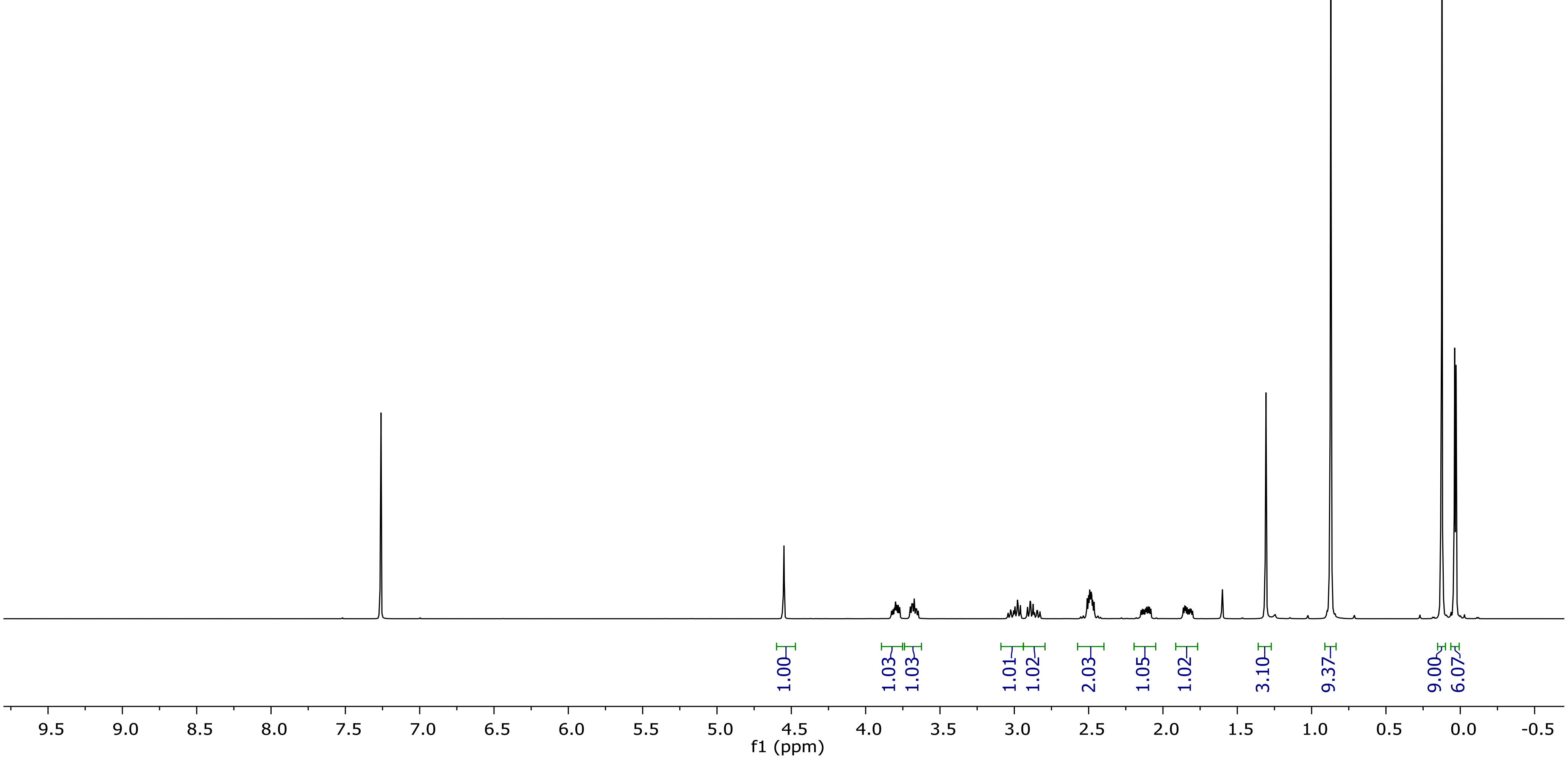

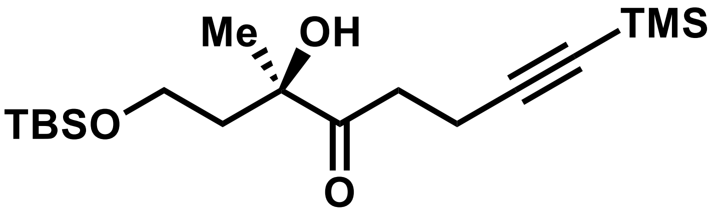

SI-9

^{13}C NMR, 101 MHz, $CDCl_3$

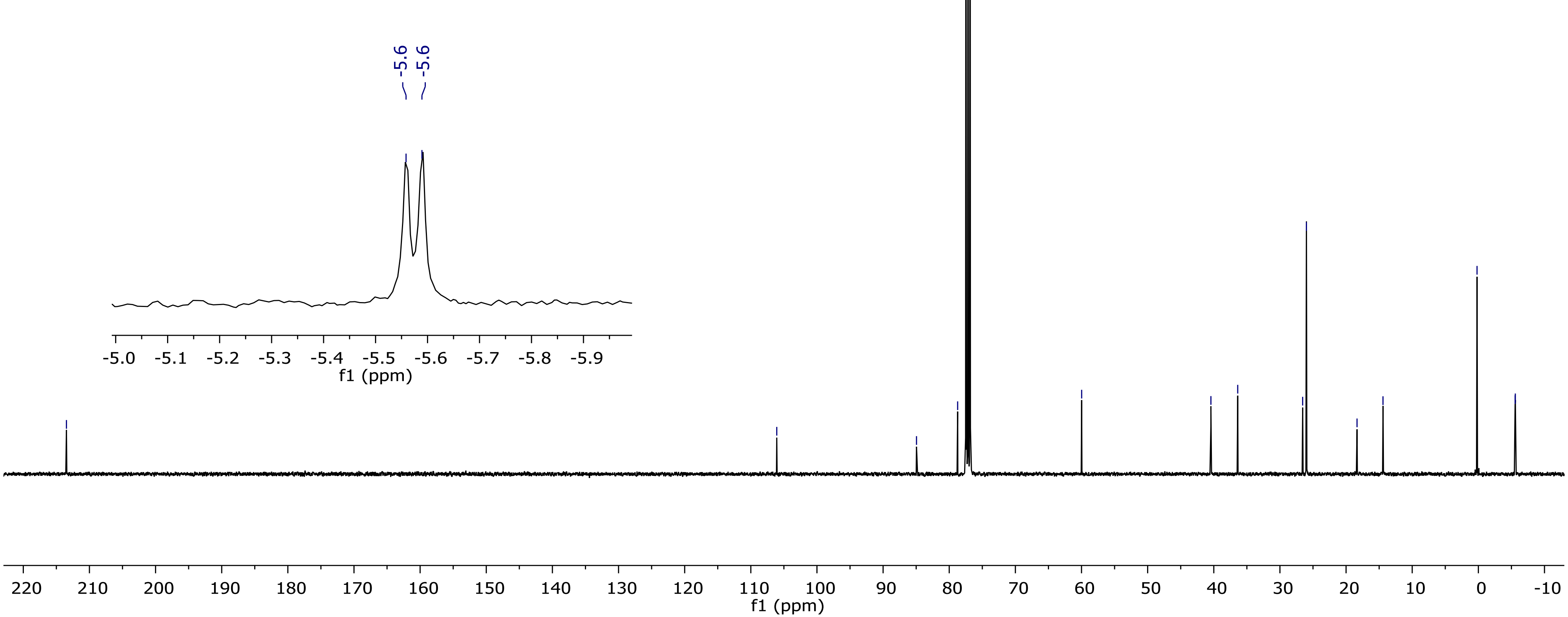

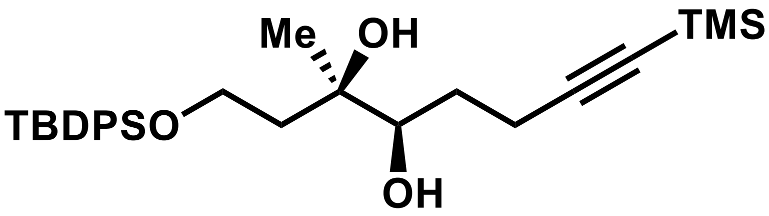

(+)-**17**

^{1}H NMR, 400 MHz, $CDCl_3$

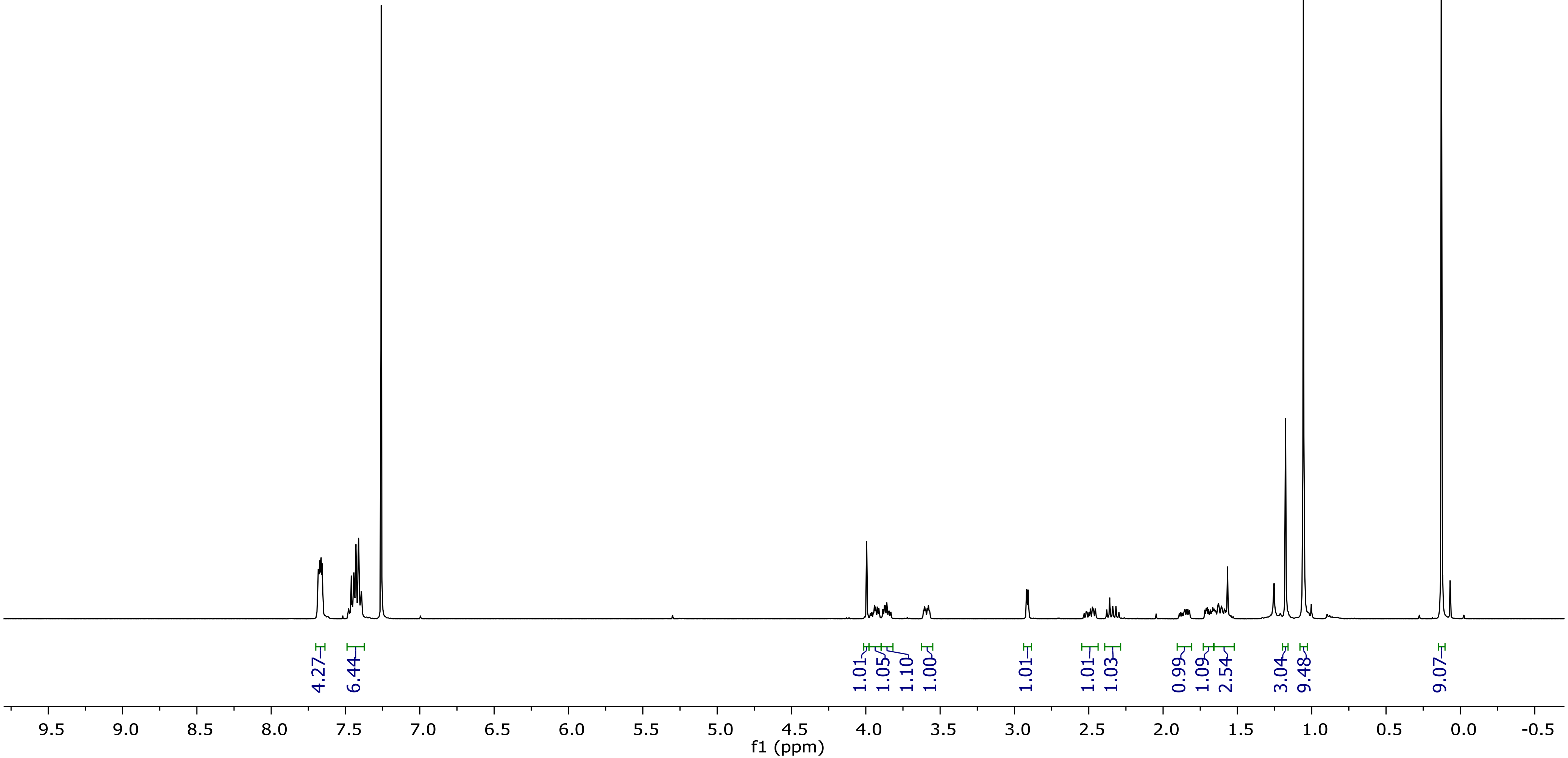

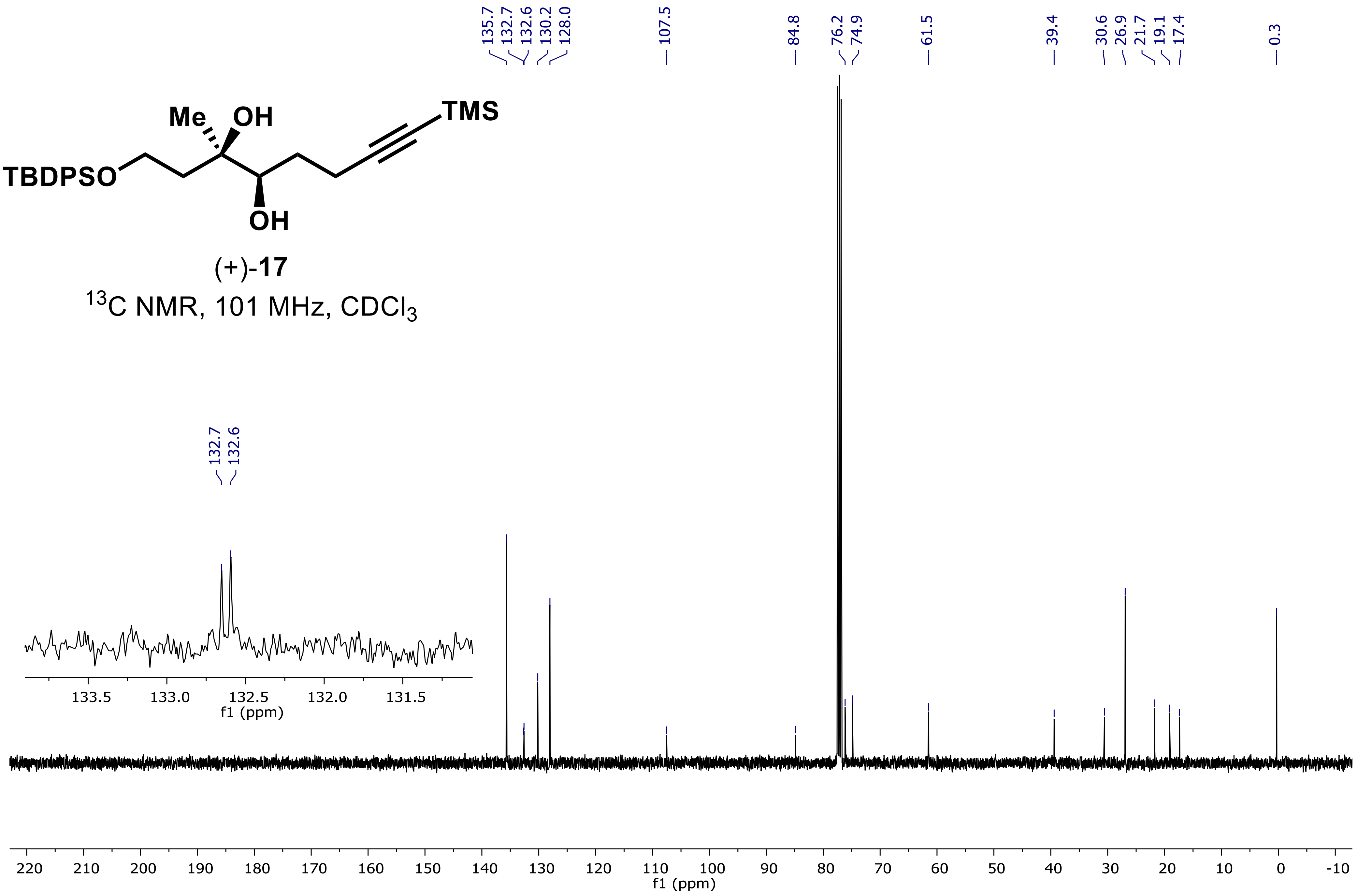

135.7
132.7
132.6
130.2
128.0
107.5
84.8
76.2
74.9
61.5
39.4
30.6
26.9
21.7
19.1
17.4
0.3
Me
OH
TMS
TBDPSO
OH
(+)-17
^{13}C NMR, 101 MHz, $CDCl_3$
132.7
132.6
133.5 133.0 132.5 132.0 131.5
f1 (ppm)
220 210 200 190 180 170 160 150 140 130 120 110 100 90 80 70 60 50 40 30 20 10 0 -10
f1 (ppm)

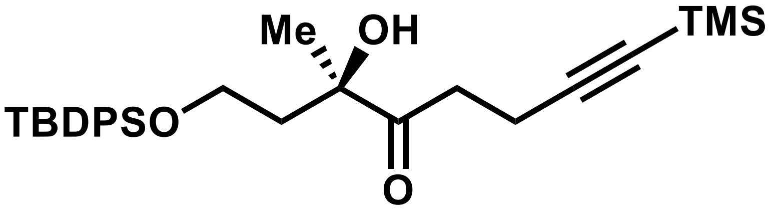

(+)-**18**

^{1}H NMR, 400 MHz, $CDCl_3$

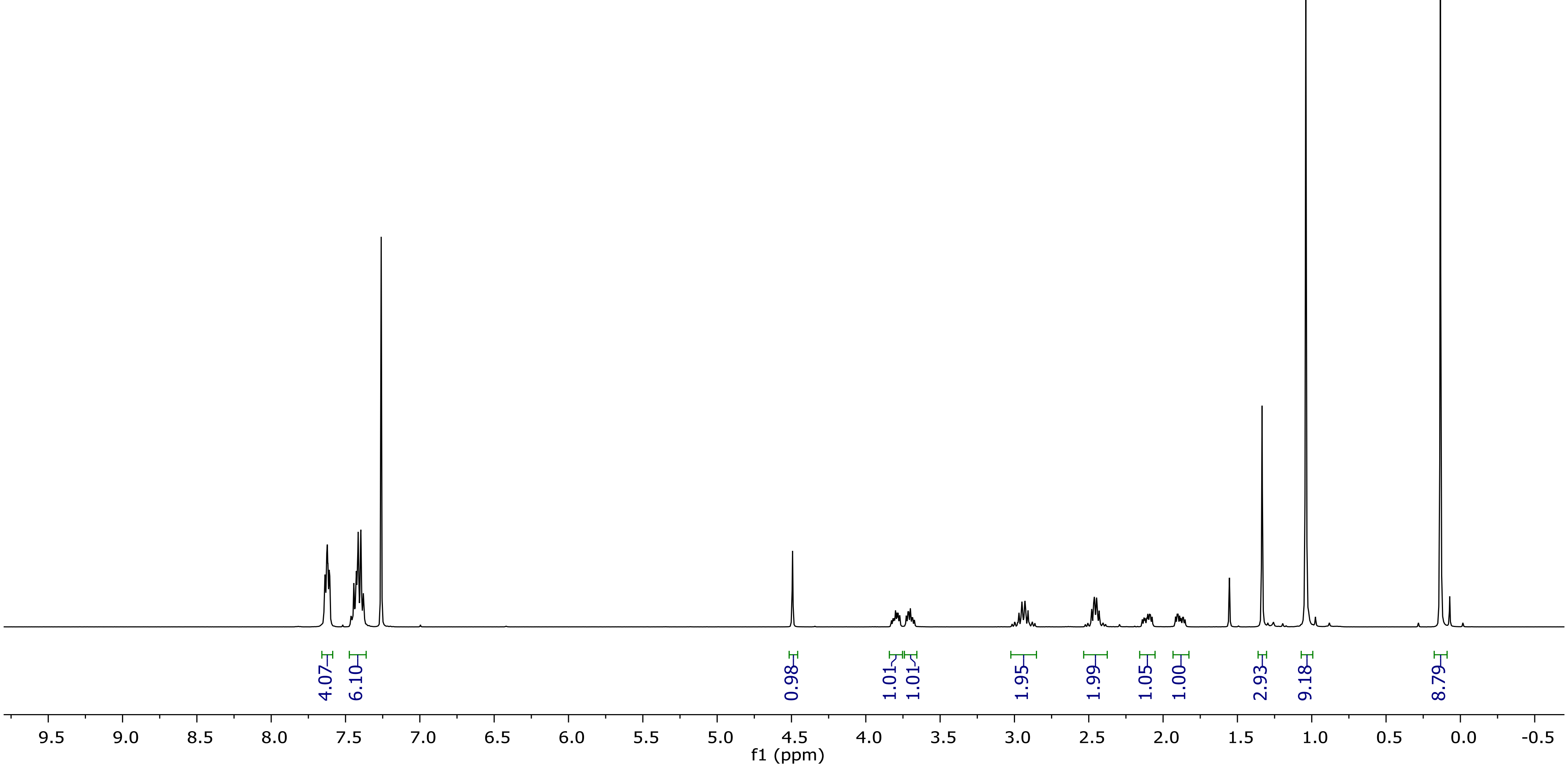

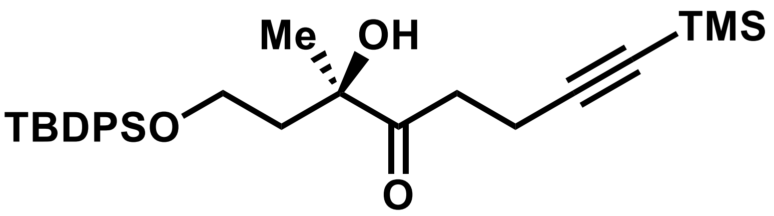

(+)-**18**

^{13}C NMR, 101 MHz, $CDCl_3$

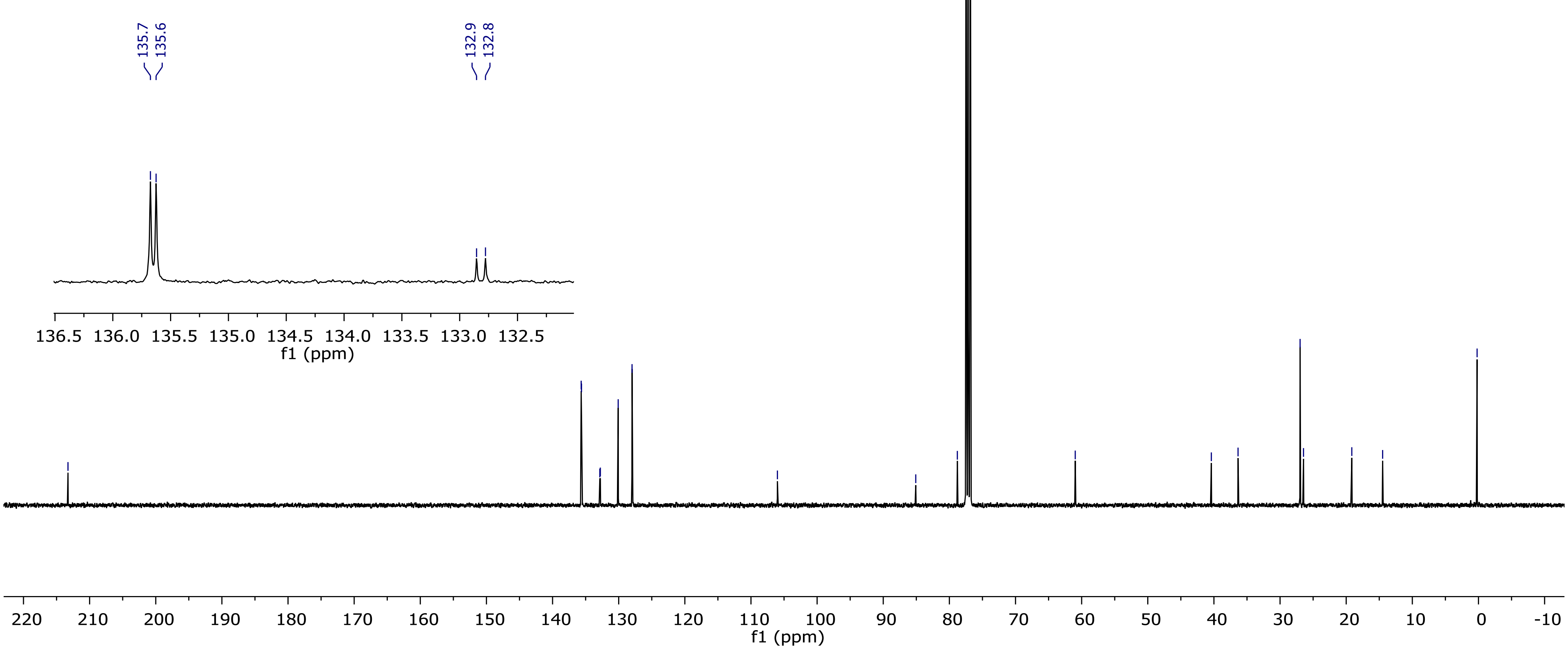

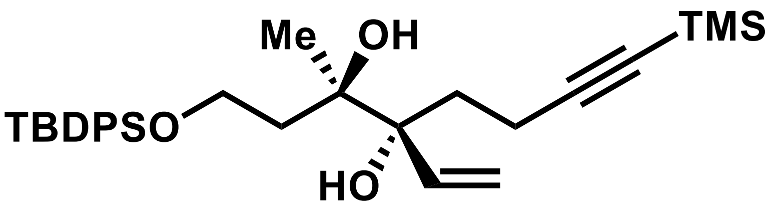

(–)-**19**

^{1}H NMR, 500 MHz, $CDCl_3$

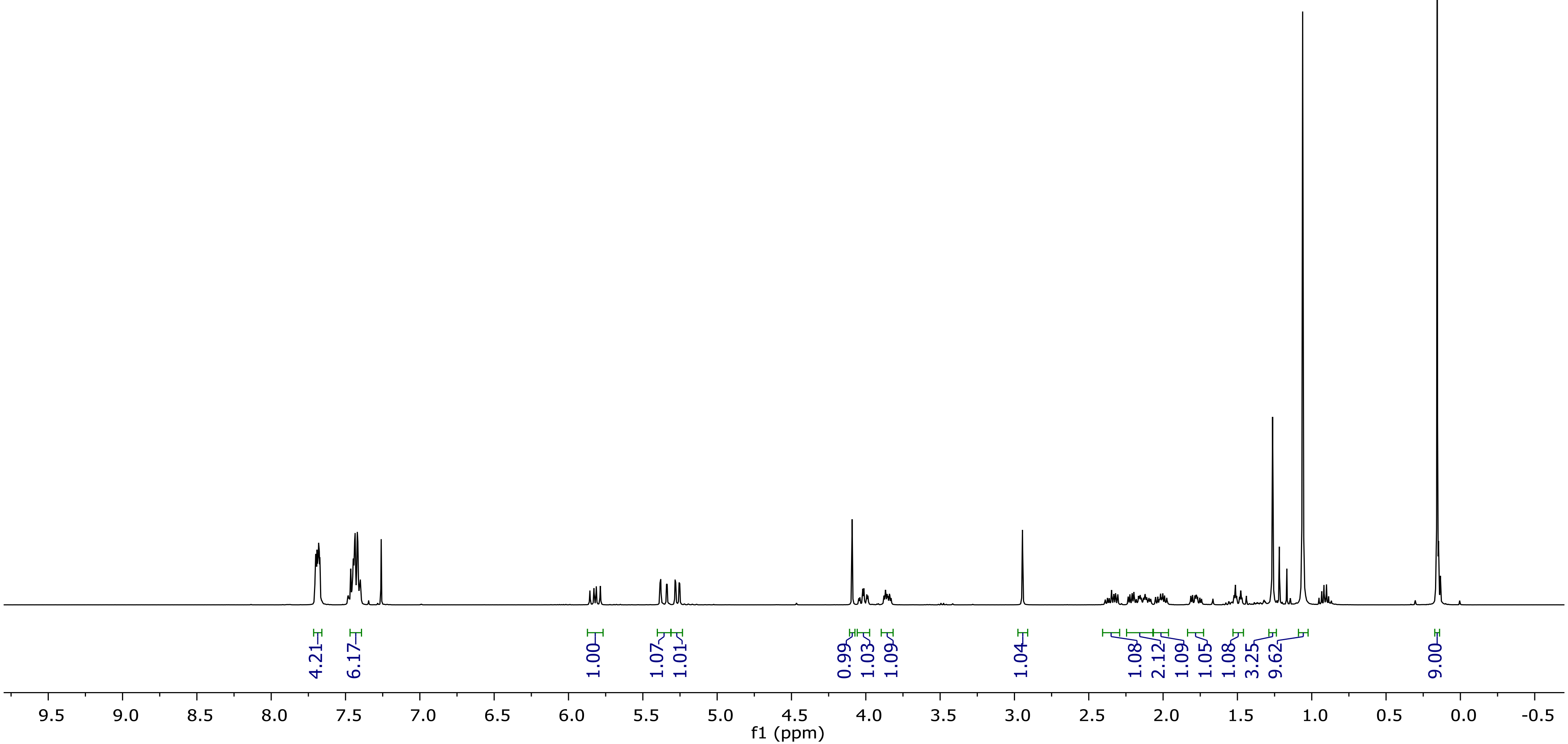

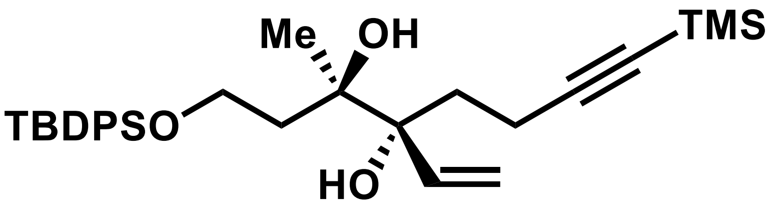

(–)-**19**

^{13}C NMR, 101 MHz, $CDCl_3$

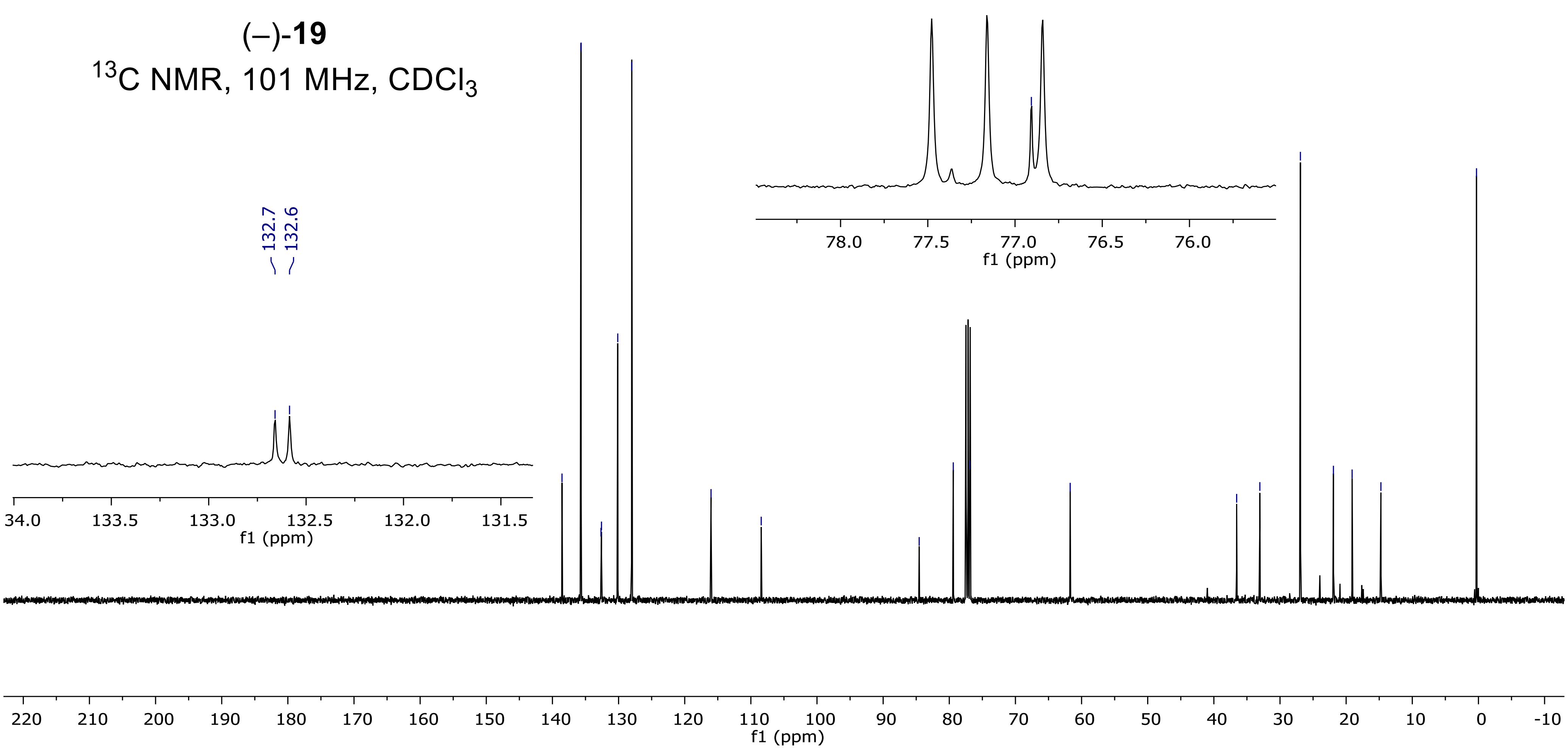

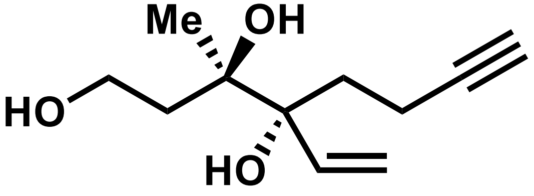

(–)-**11**

^{1}H NMR, 500 MHz, $CDCl_3$

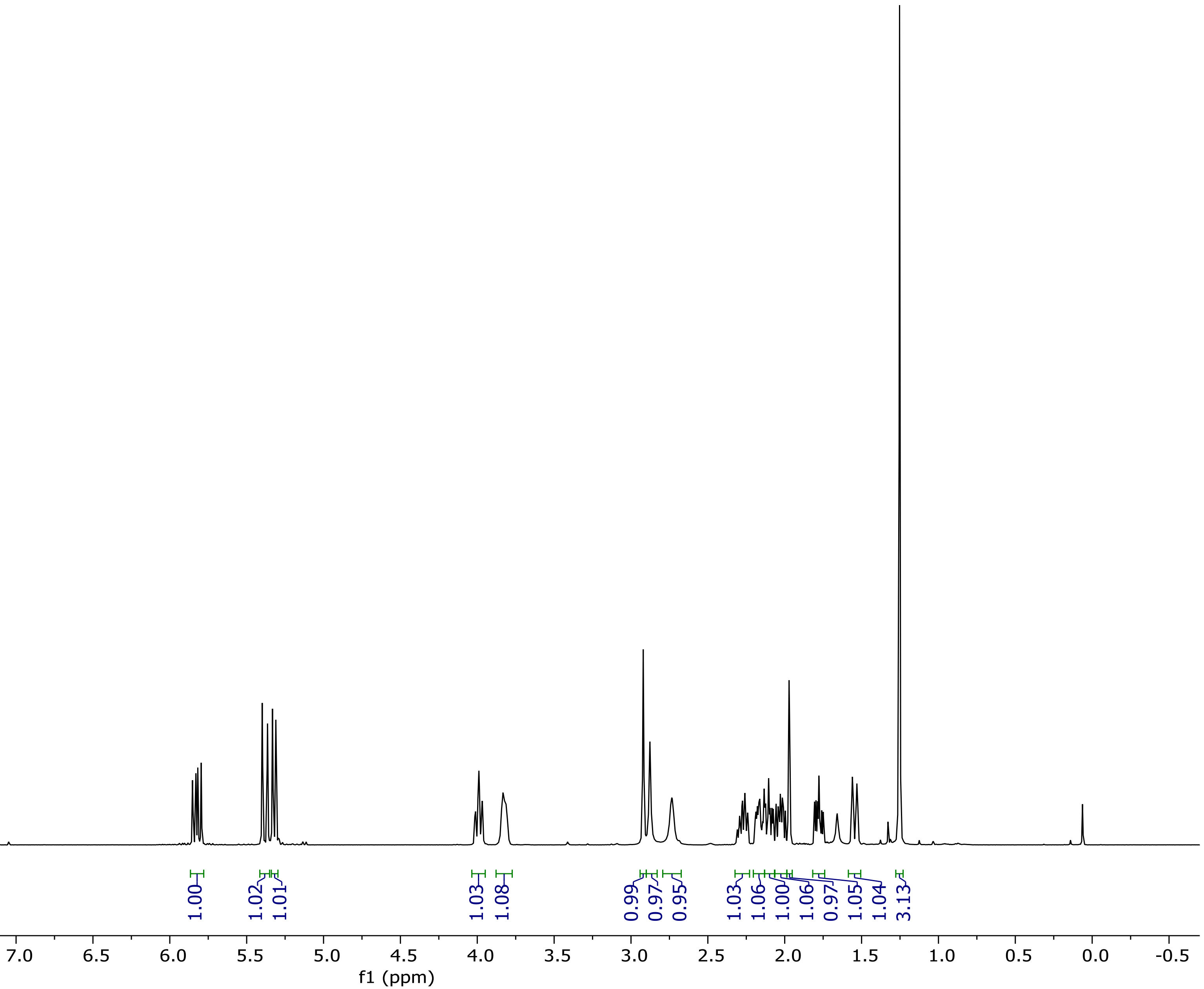

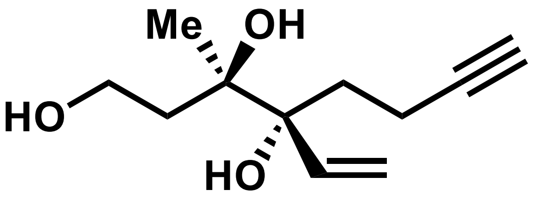

(–)-**11**

^{13}C NMR, 126 MHz, $CDCl_3$

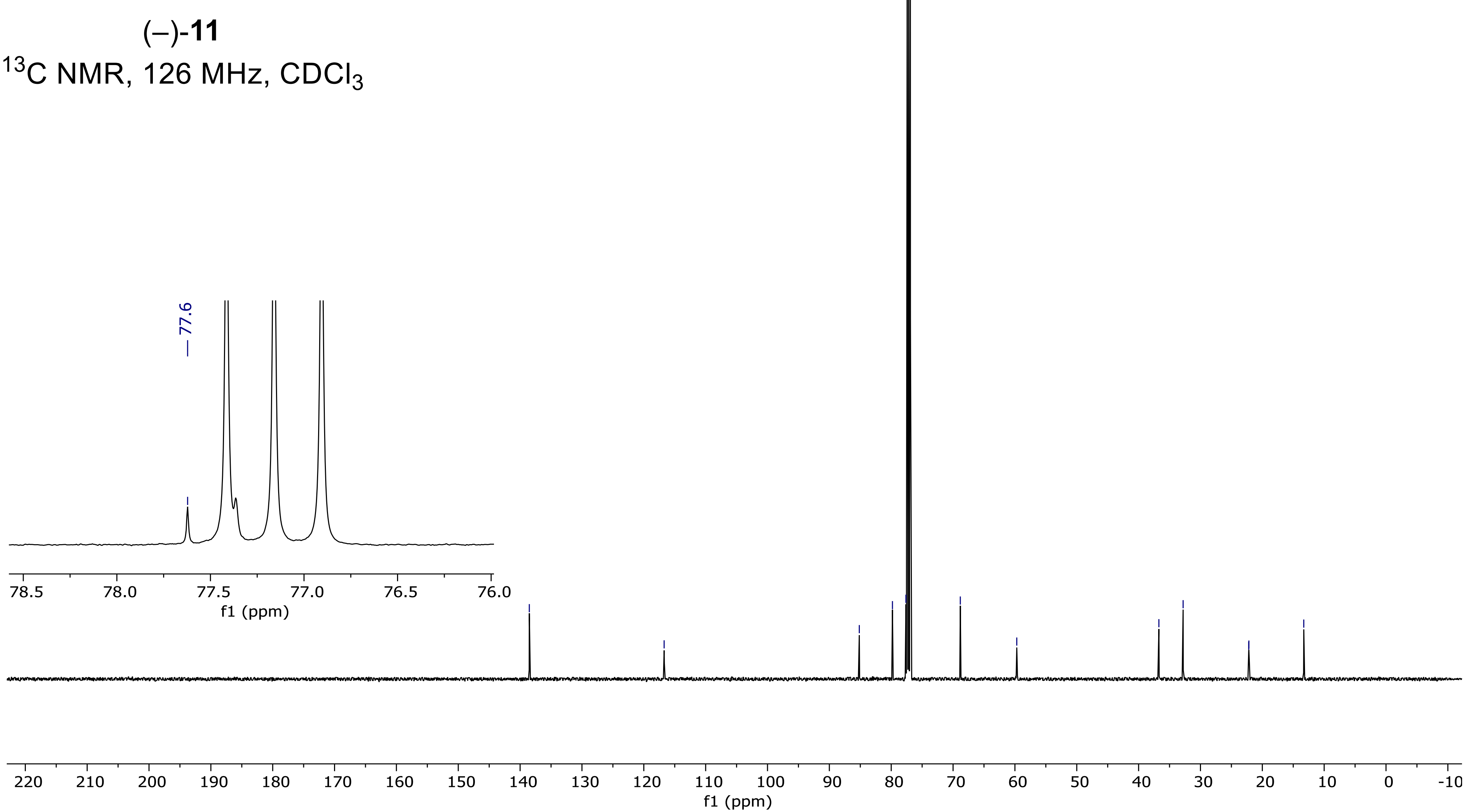

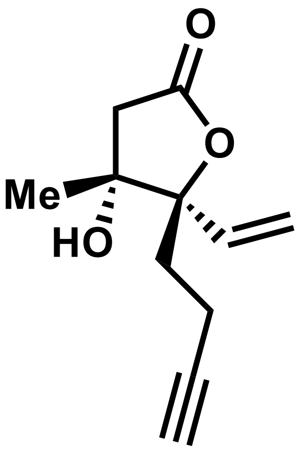

(+)-**20**

^{1}H NMR, 500 MHz, $CDCl_3$

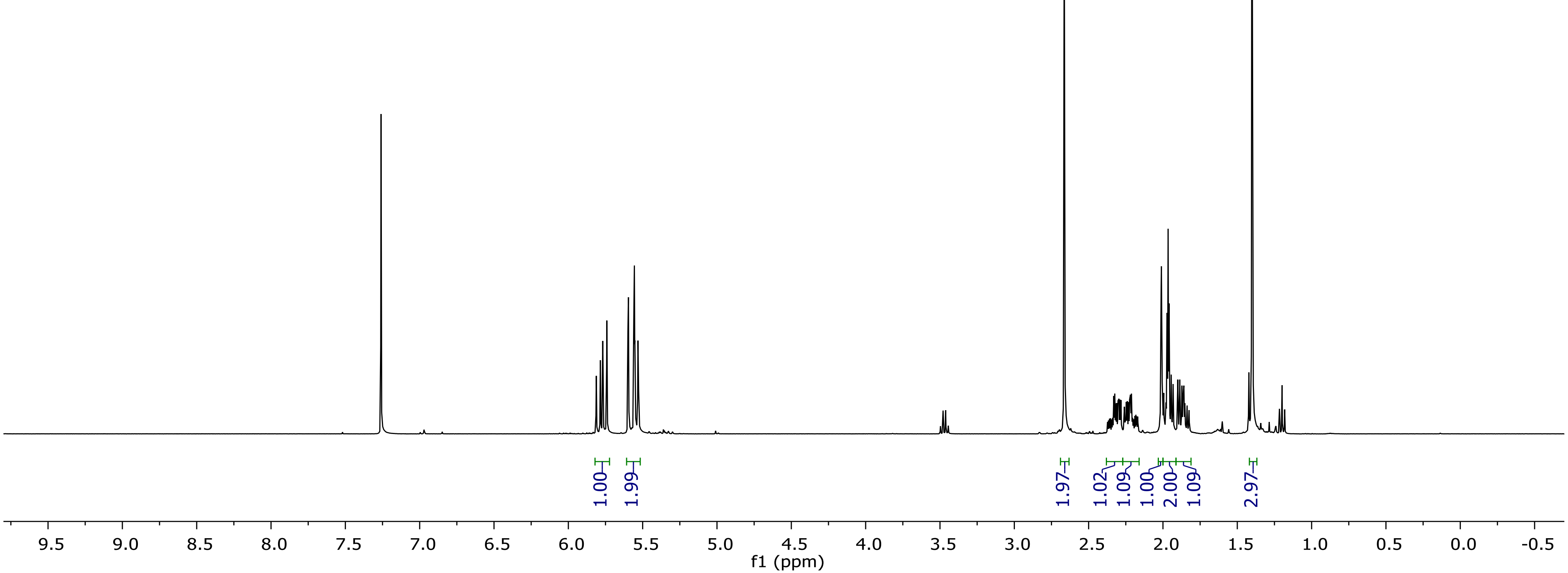

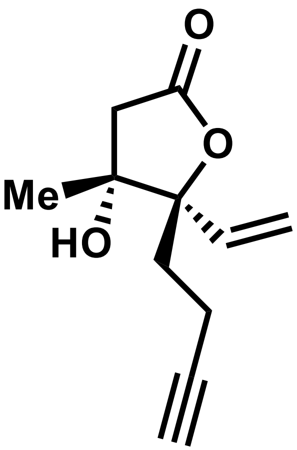

(+)-**20**

^{13}C NMR, 126 MHz, $CDCl_3$

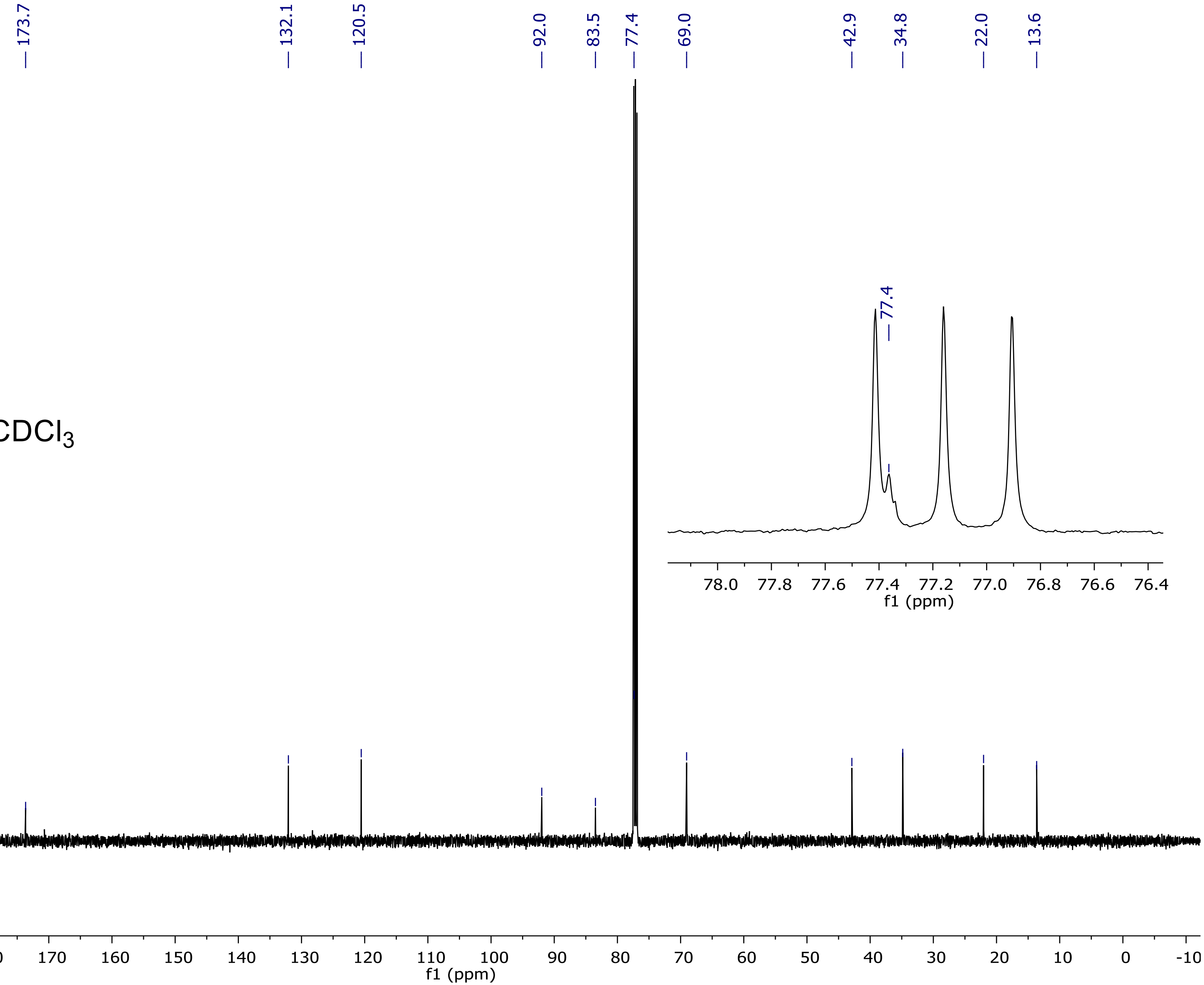

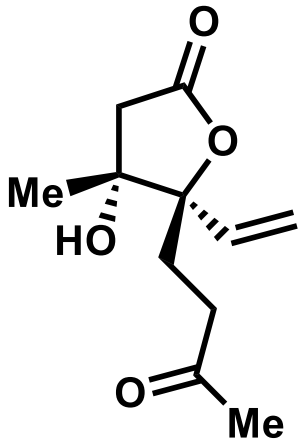

(+)-**9**

^{1}H NMR, 400 MHz, $CDCl_3$

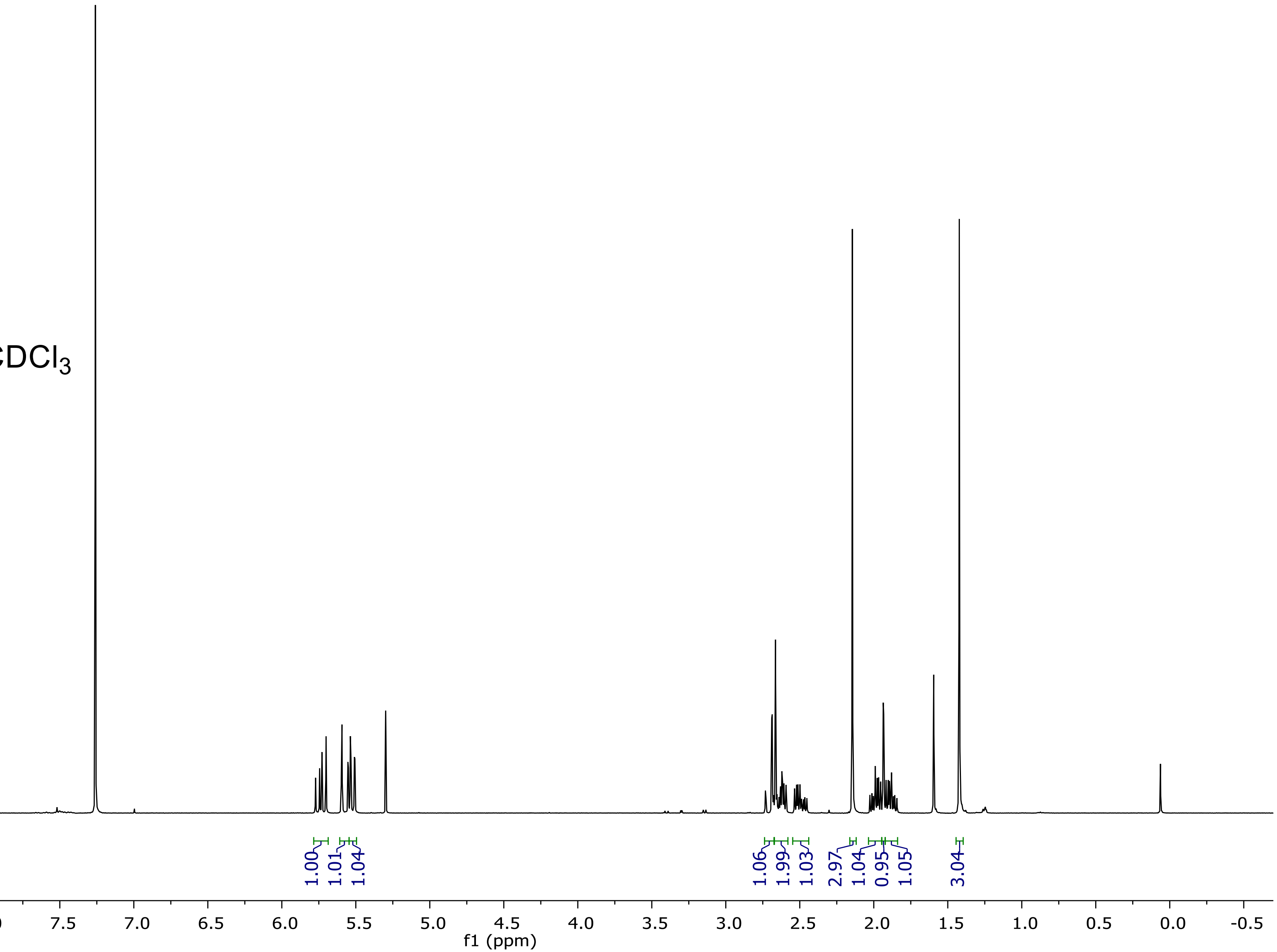

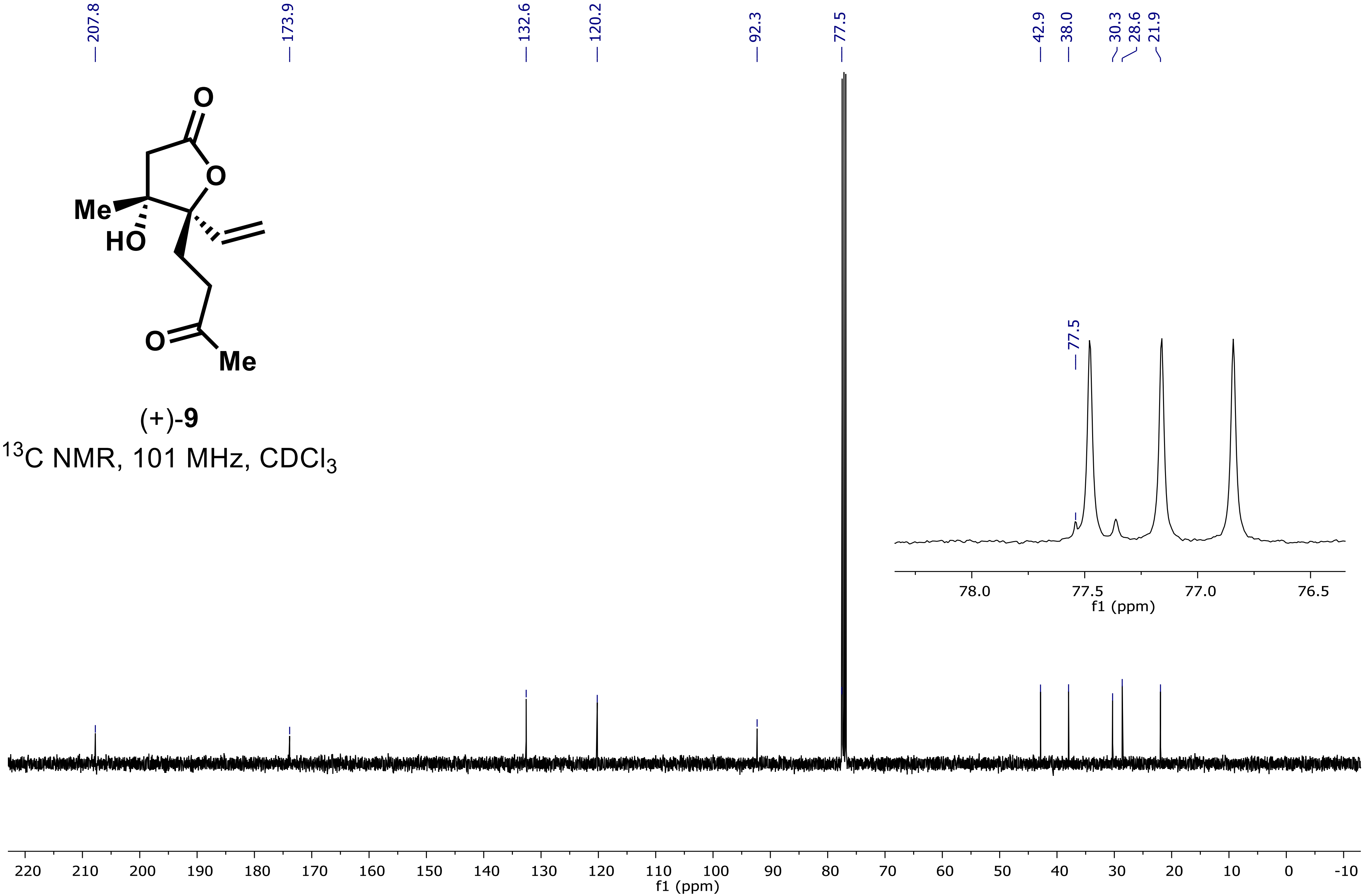

O
O
Me
HO
O
Me
(+)-9
13C NMR, 101 MHz, CDCl3
207.8
173.9
132.6
120.2
92.3
77.5
42.9
38.0
30.3
28.6
21.9
77.5
78.0
77.5
77.0
76.5
f1 (ppm)
220 210 200 190 180 170 160 150 140 130 120 110 100 90 80 70 60 50 40 30 20 10 0 -10
f1 (ppm)

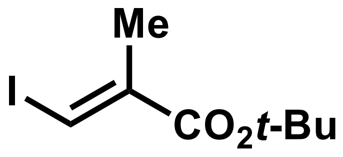

10 *t*-Bu ester

^{1}H NMR, 400 MHz, $CDCl_3$

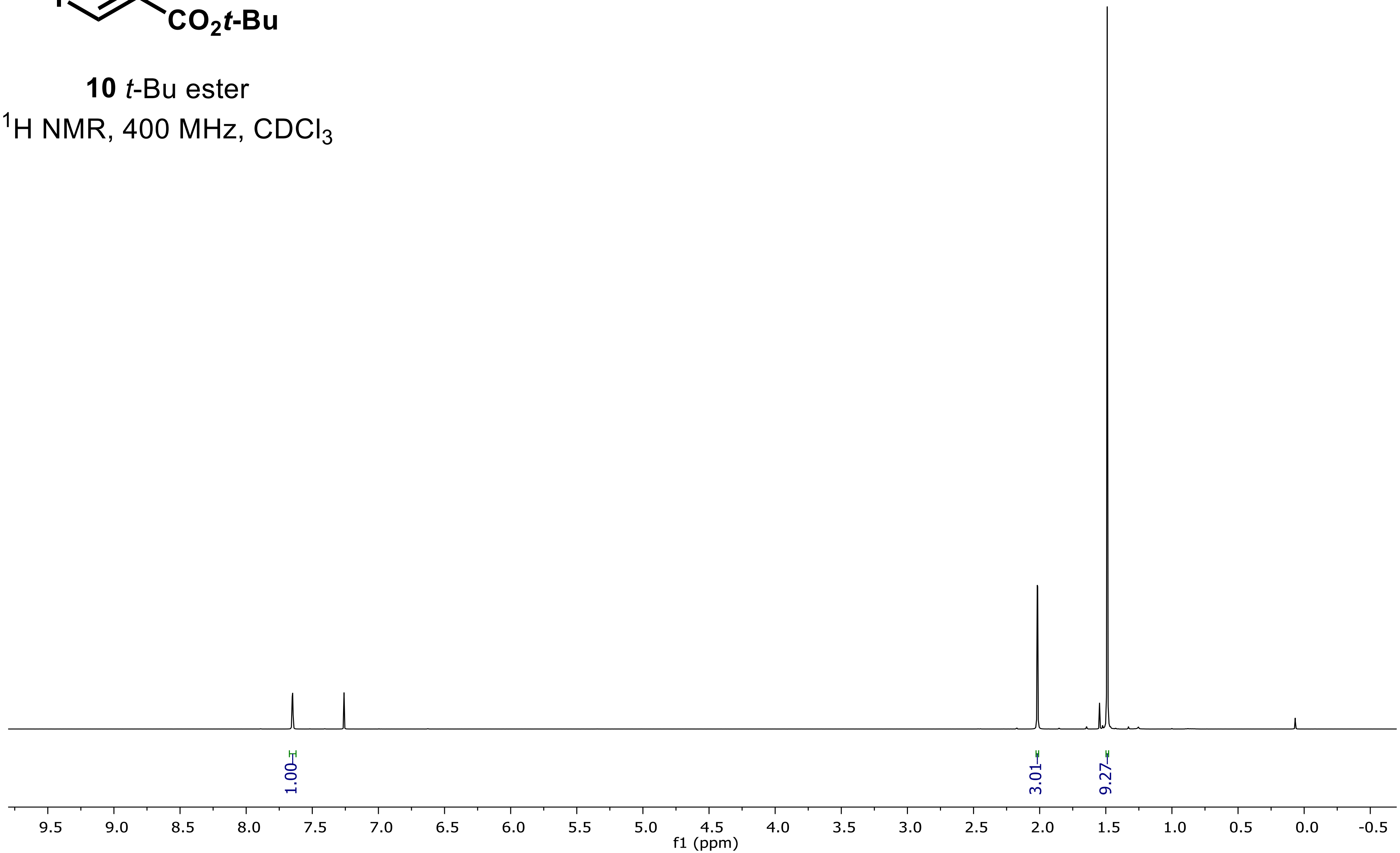

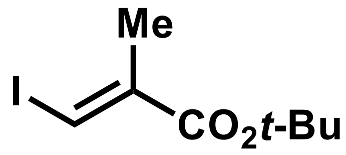

10 *t*-Bu ester

^{13}C NMR, 101 MHz, $CDCl_3$

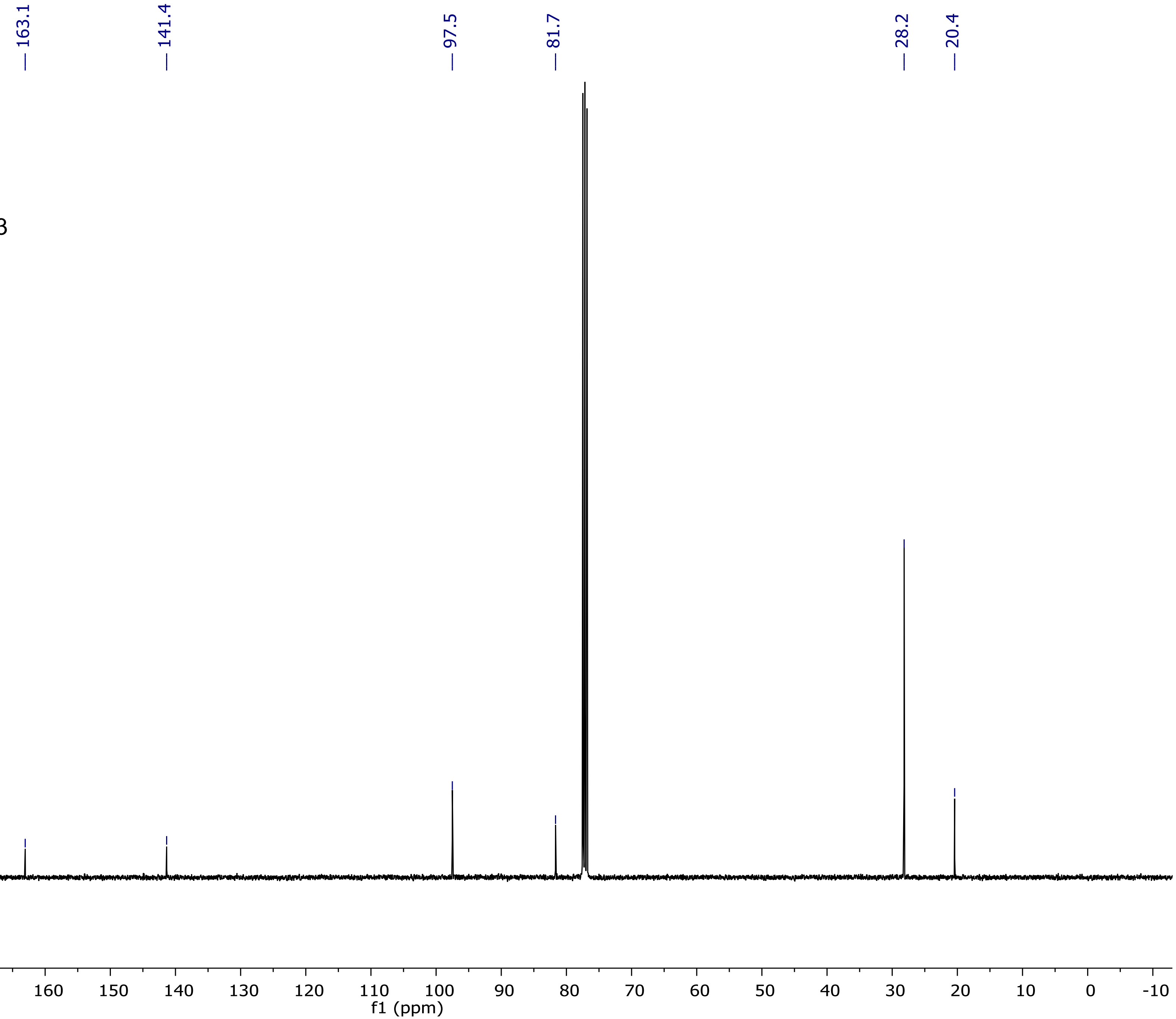

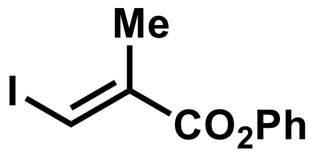

10 *t*-Bu ester

^{1}H NMR, 400 MHz, $CDCl_3$

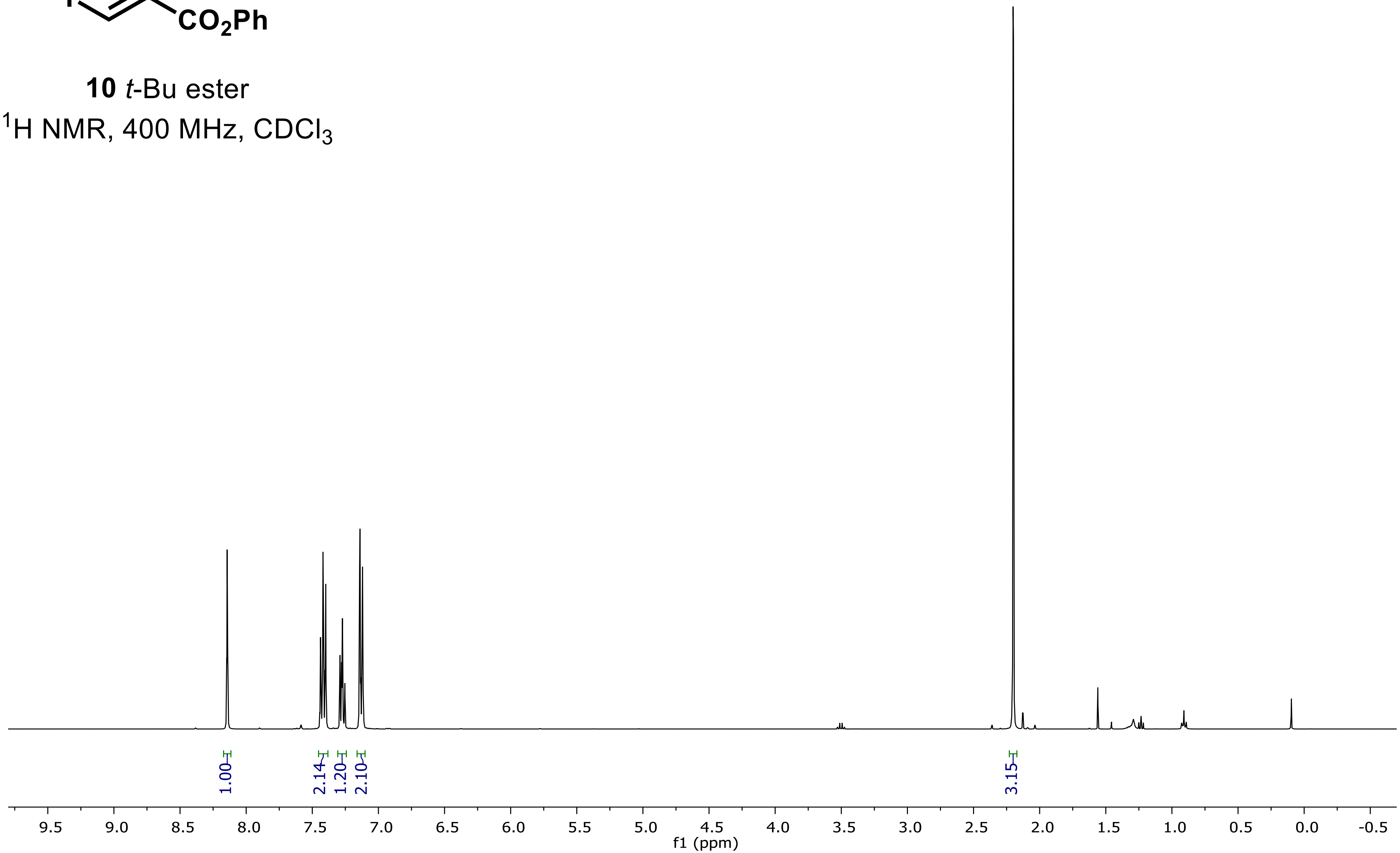

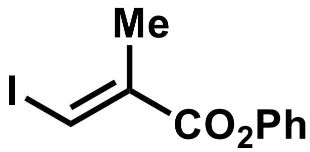

10 *t*-Bu ester

^{13}C NMR, 101 MHz, $CDCl_3$

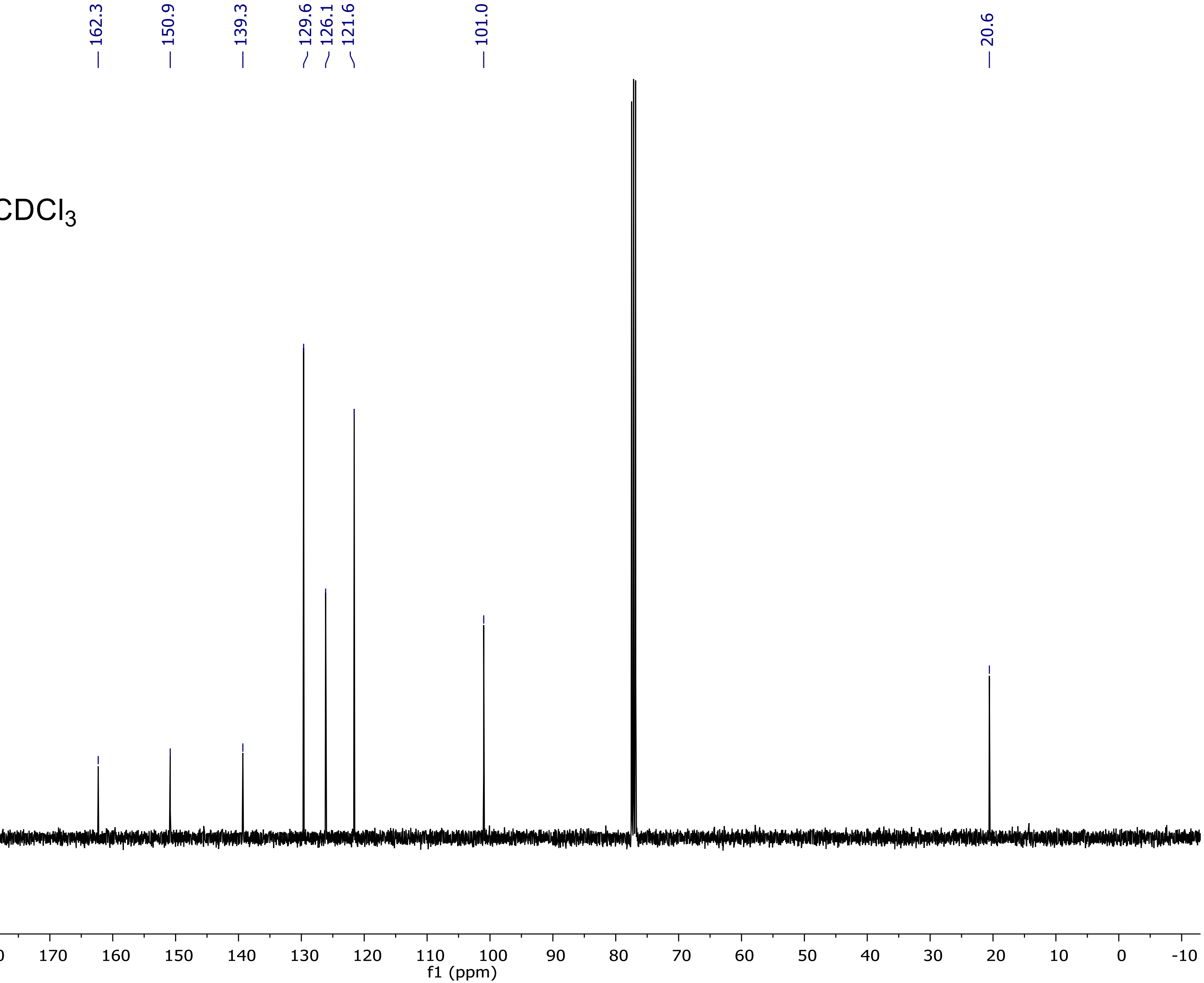

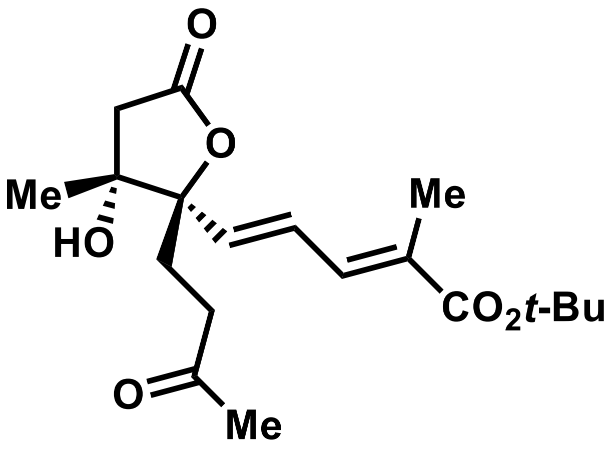

(+)-**8** *t*-Bu ester

^{1}H NMR, 400 MHz, $CDCl_3$

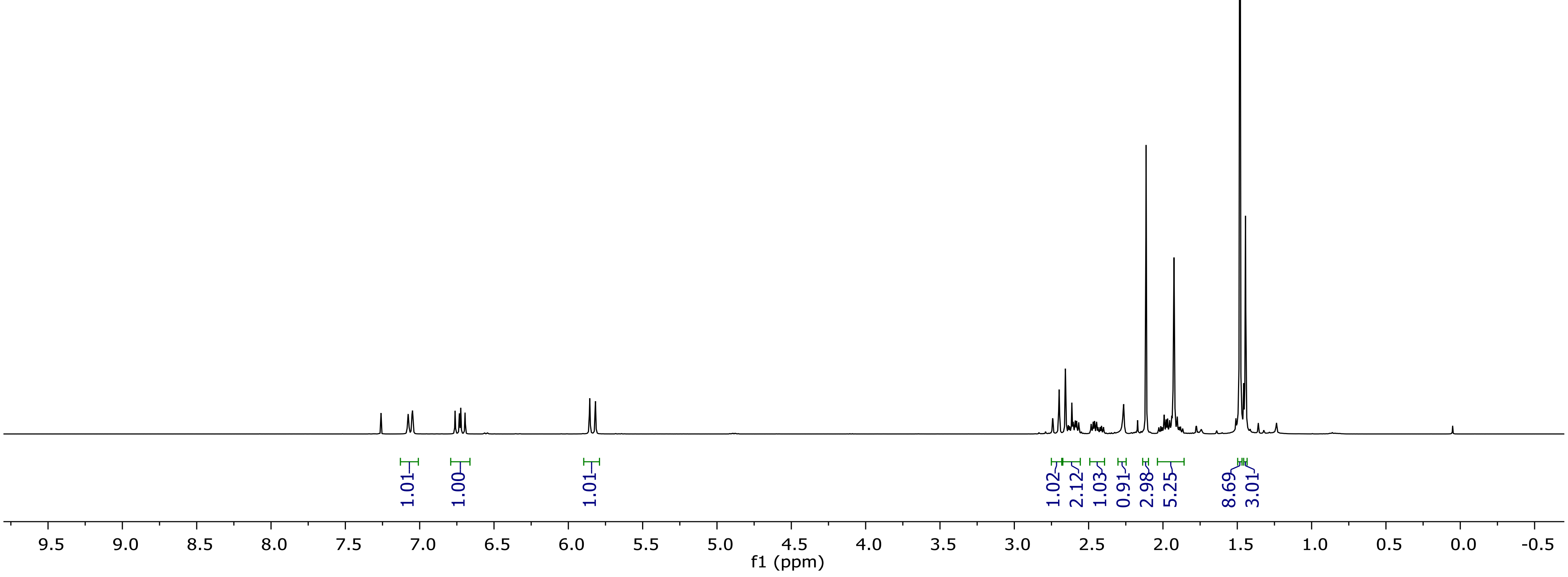

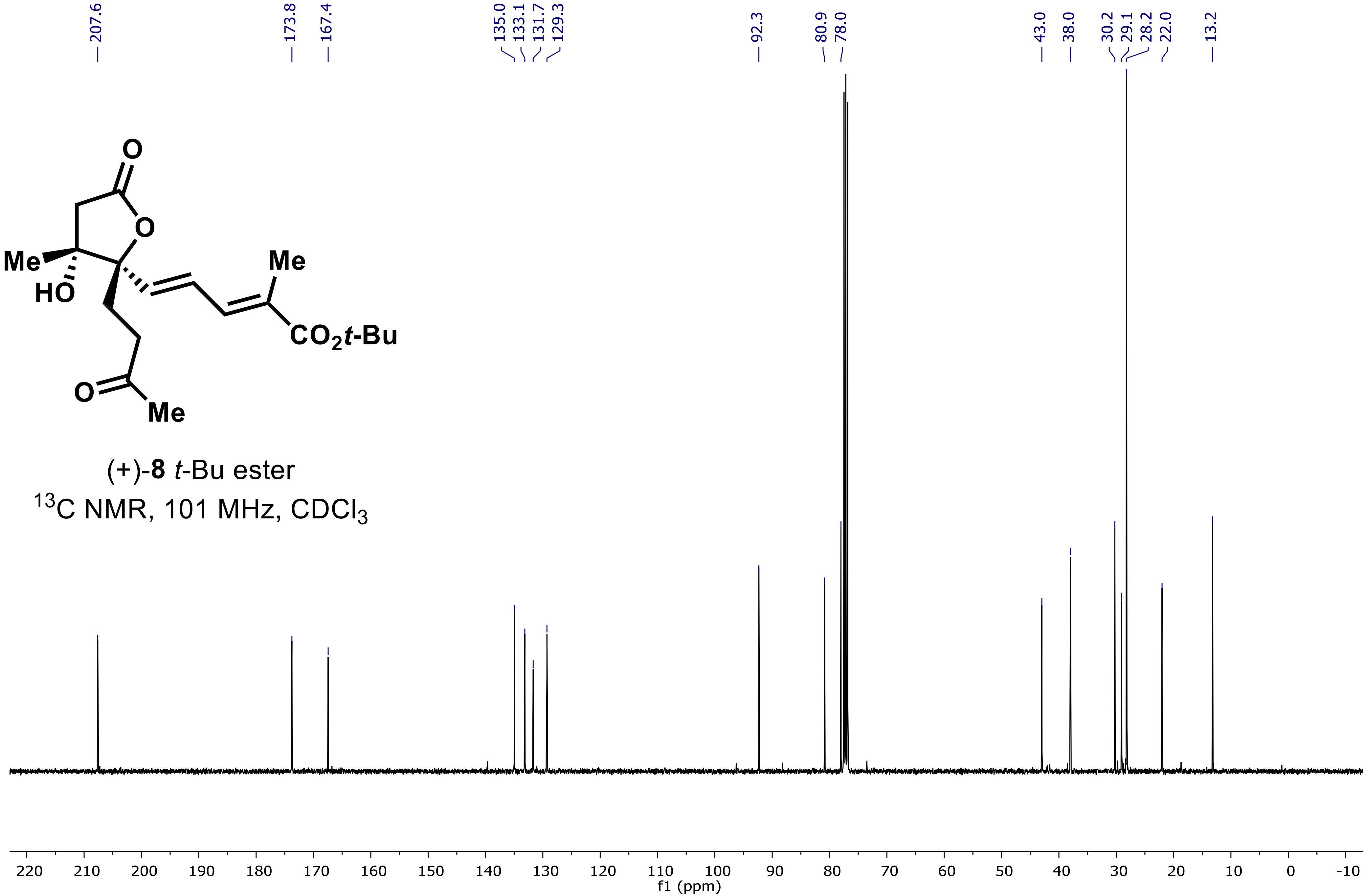

(+)-**8** *t*-Bu ester
^{13}C NMR, 101 MHz, $CDCl_3$

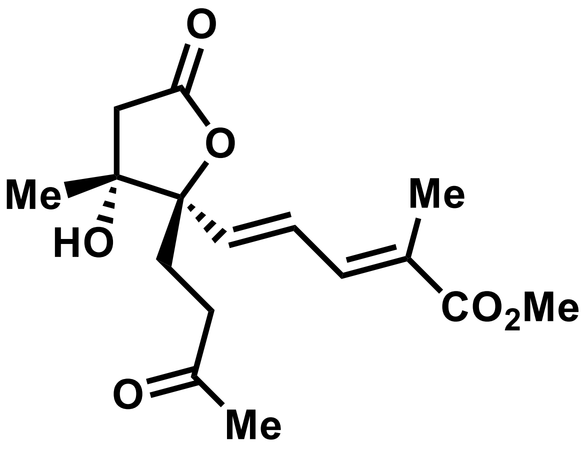

(+)-**8** Me ester

^{1}H NMR, 400 MHz, $CDCl_3$

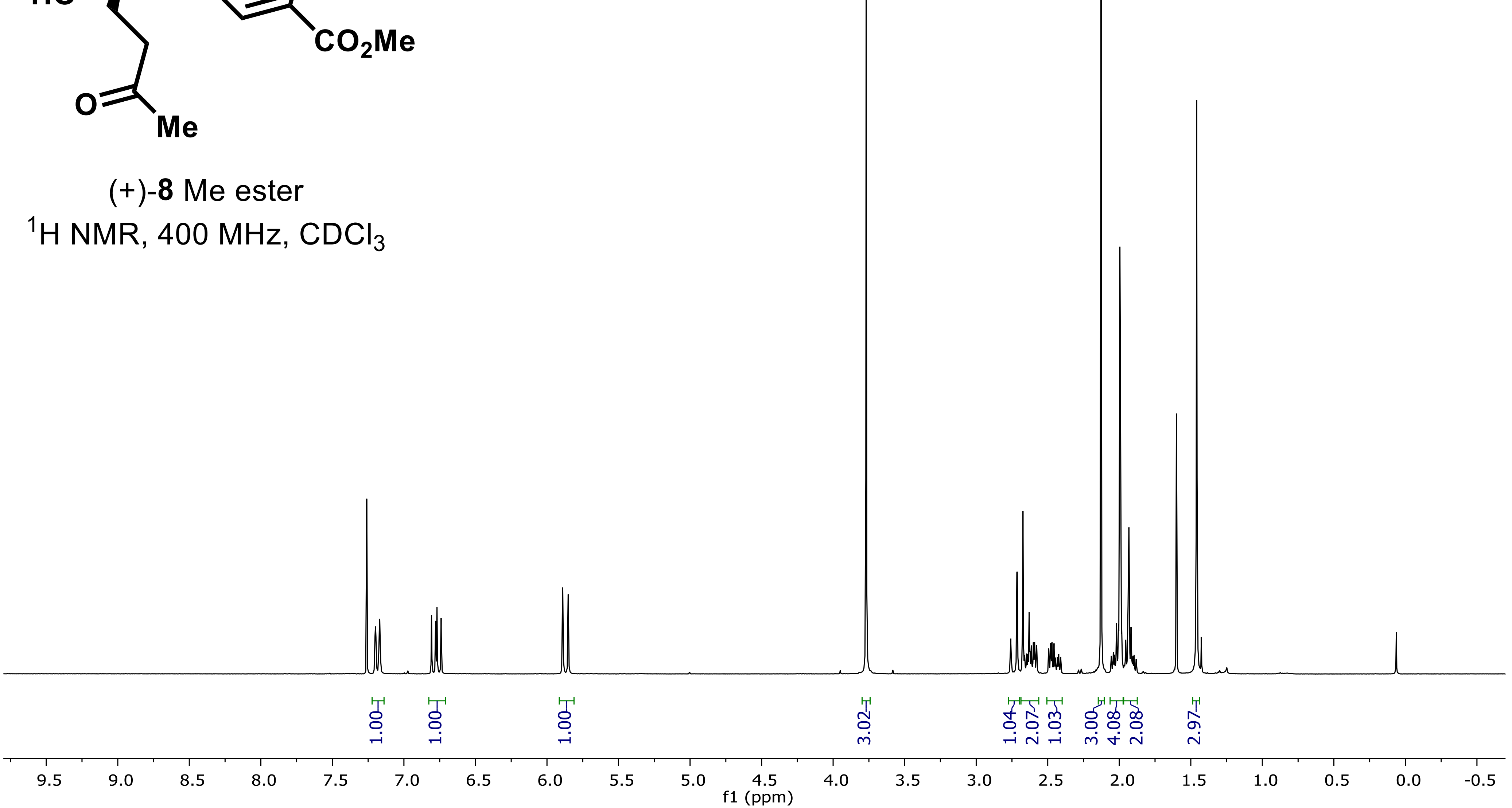

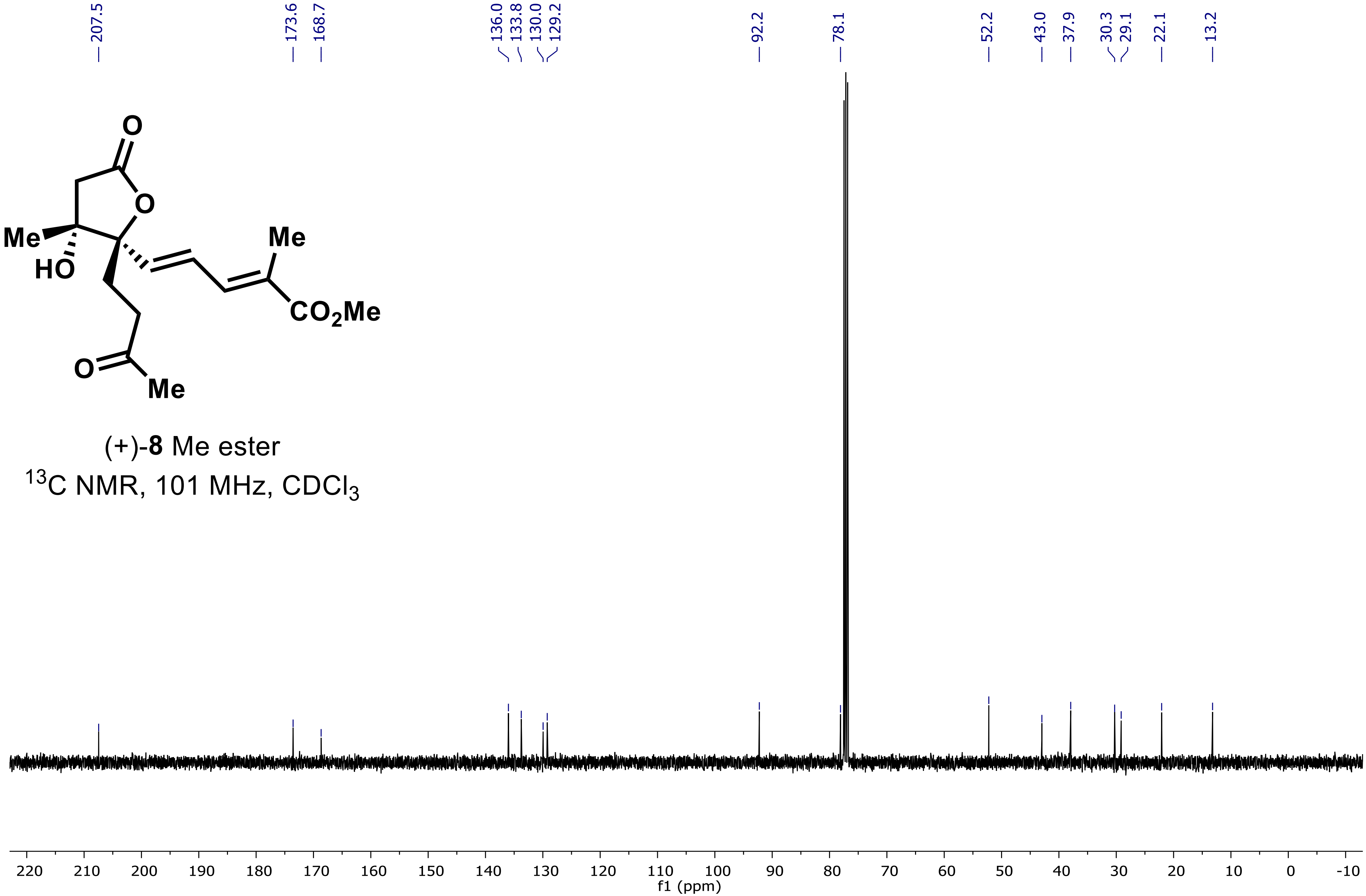

(+)-**8** Me ester

^{13}C NMR, 101 MHz, $CDCl_3$

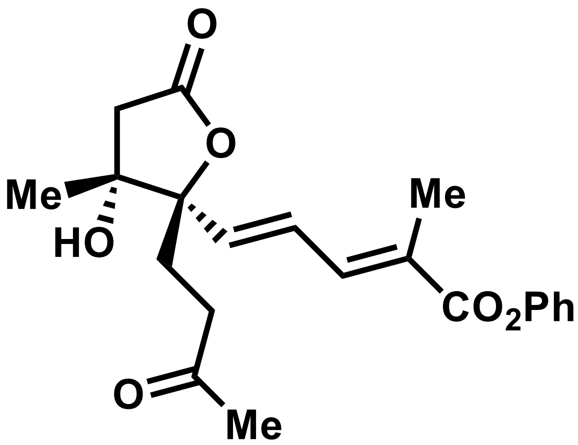

(+)-**8** Ph ester

^{1}H NMR, 400 MHz, $CDCl_3$

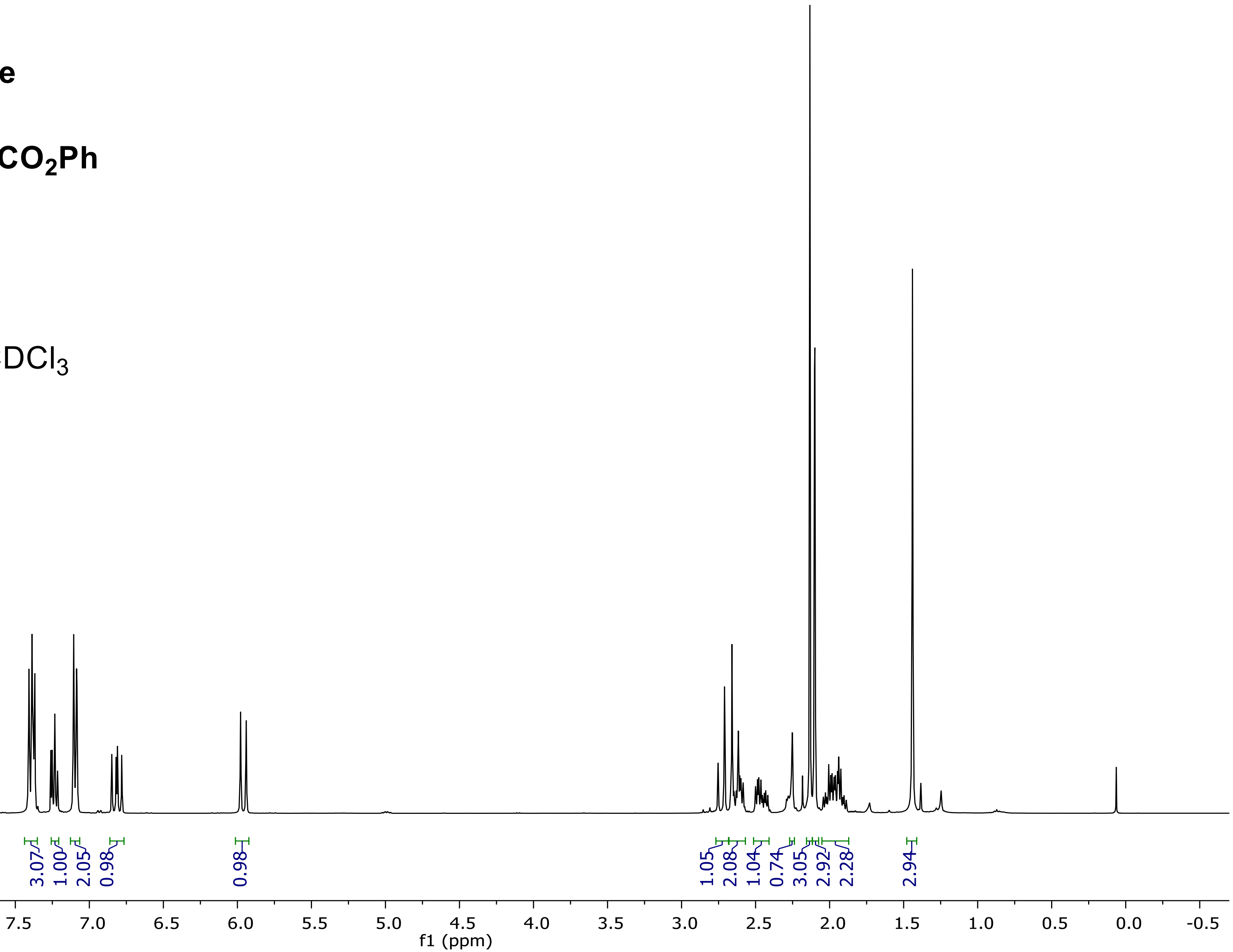

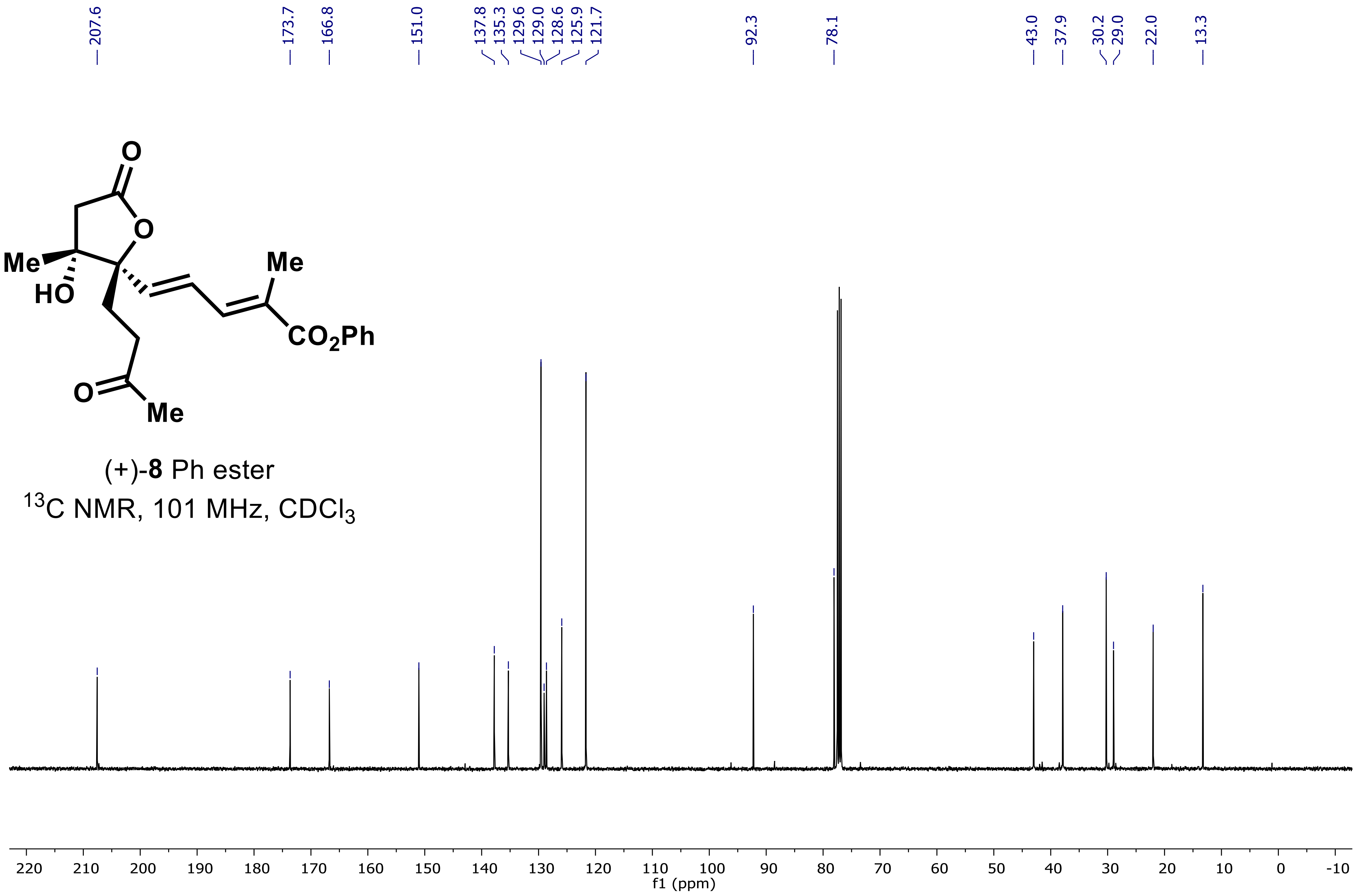

(+)-**8** Ph ester
^{13}C NMR, 101 MHz, $CDCl_3$

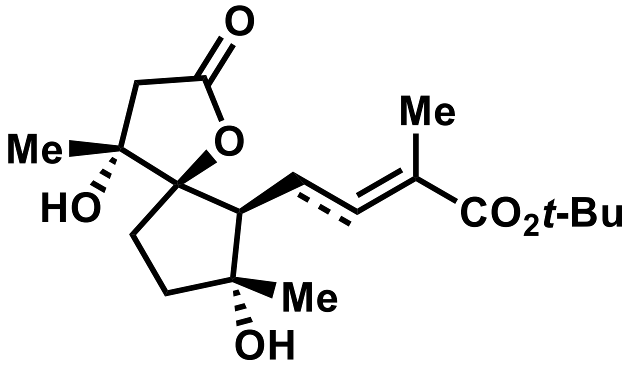

7 *t*-Bu ester:*iso*-**7** *t*-Bu ester (1.3:1)
^{1}H NMR, 400 MHz, $CDCl_3$

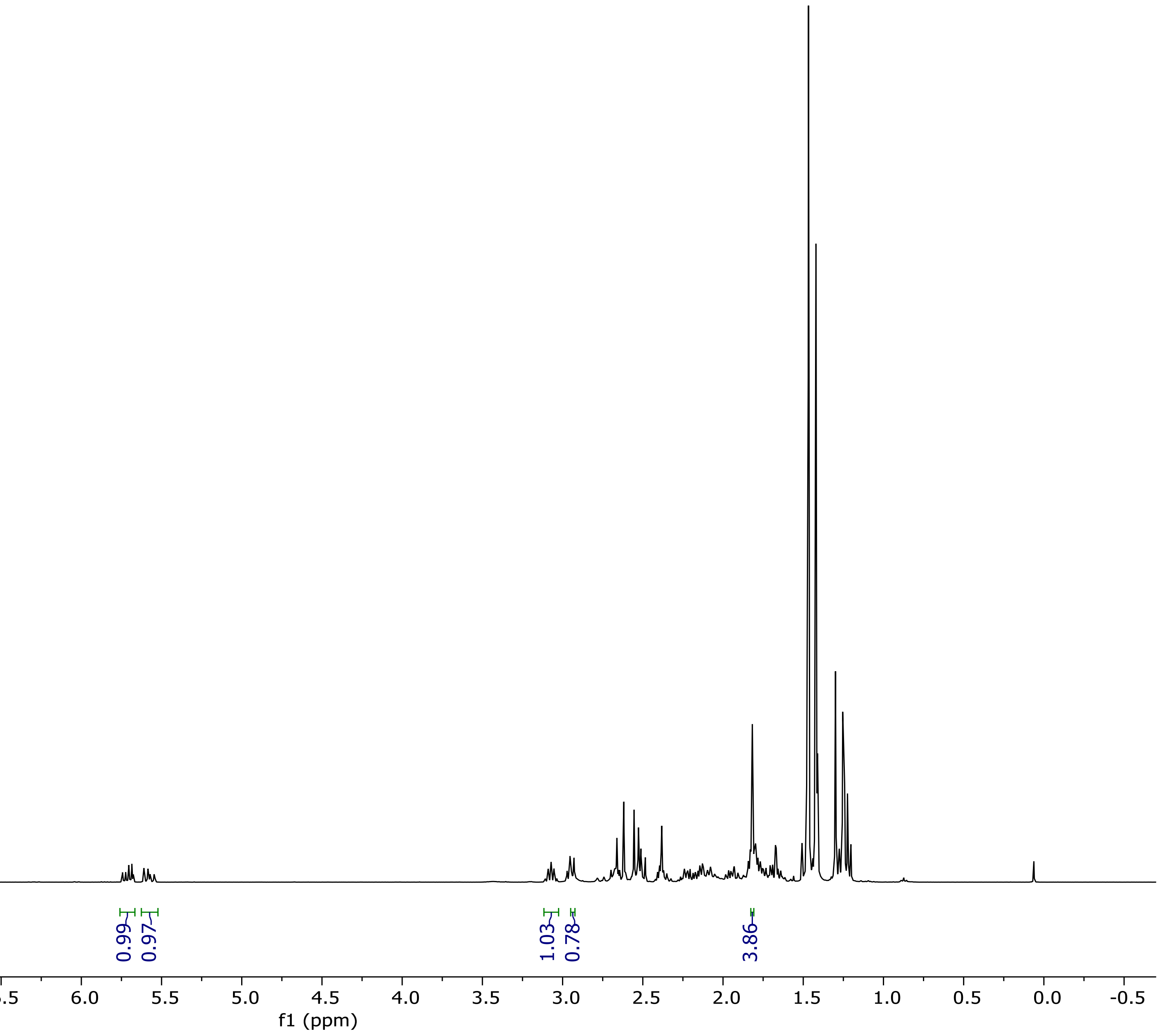

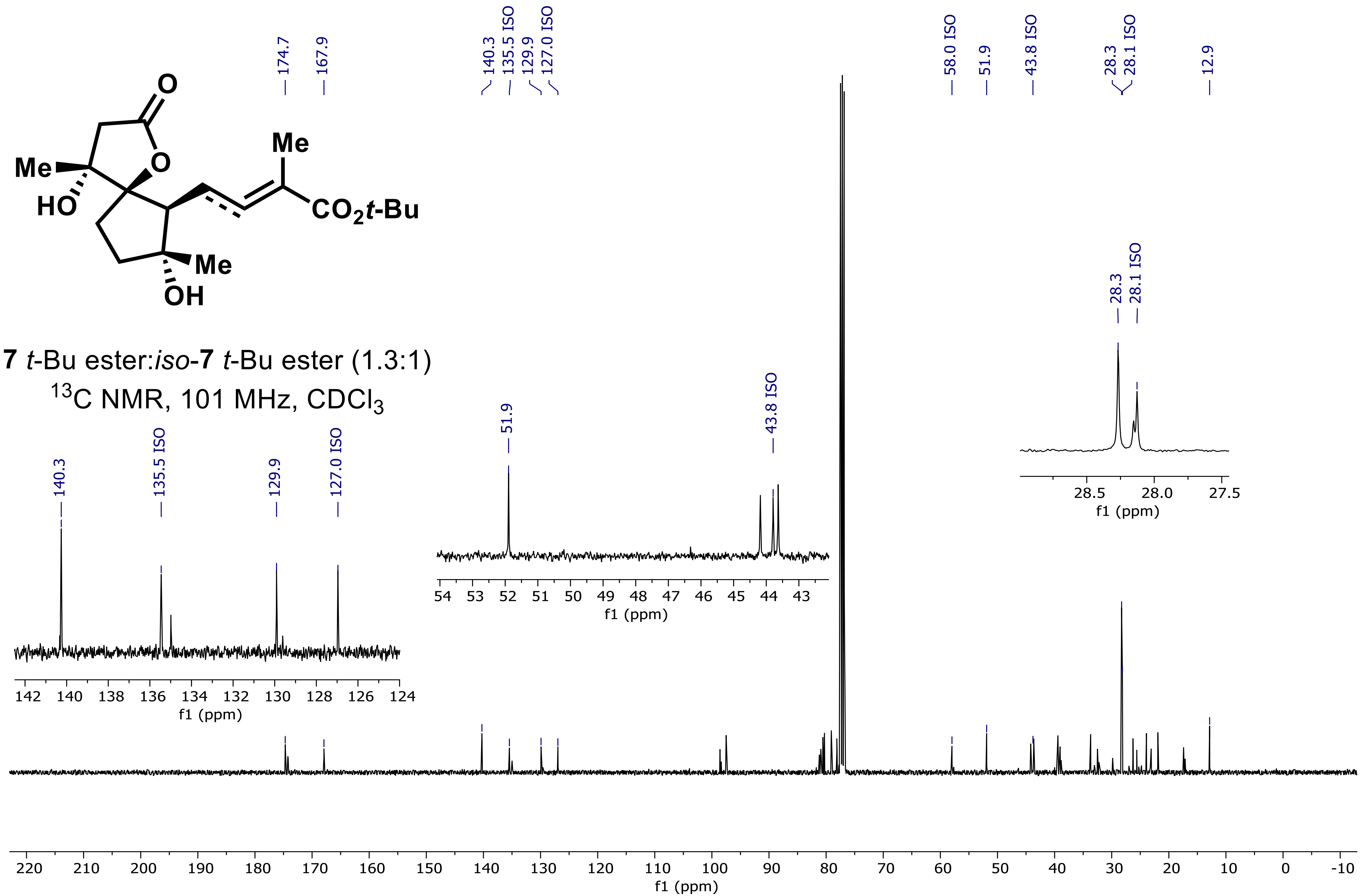
7 *t*-Bu ester:*iso*-7 *t*-Bu ester (1.3:1)
^{13}C NMR, 101 MHz, $CDCl_3$
174.7
167.9
140.3
135.5 ISO
129.9
127.0 ISO
58.0 ISO
51.9
43.8 ISO
28.3
28.1 ISO
12.9
f1 (ppm)

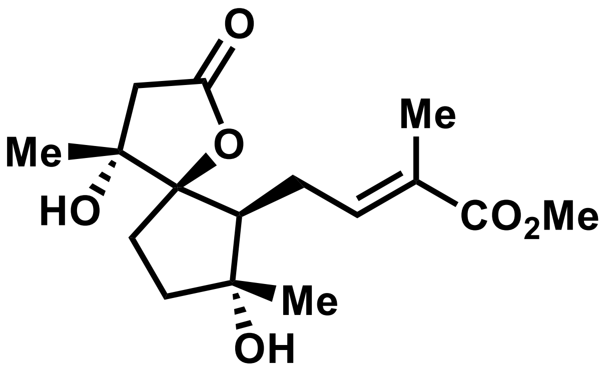

(+)-**7** Me ester

^{1}H NMR, 400 MHz, $CDCl_3$

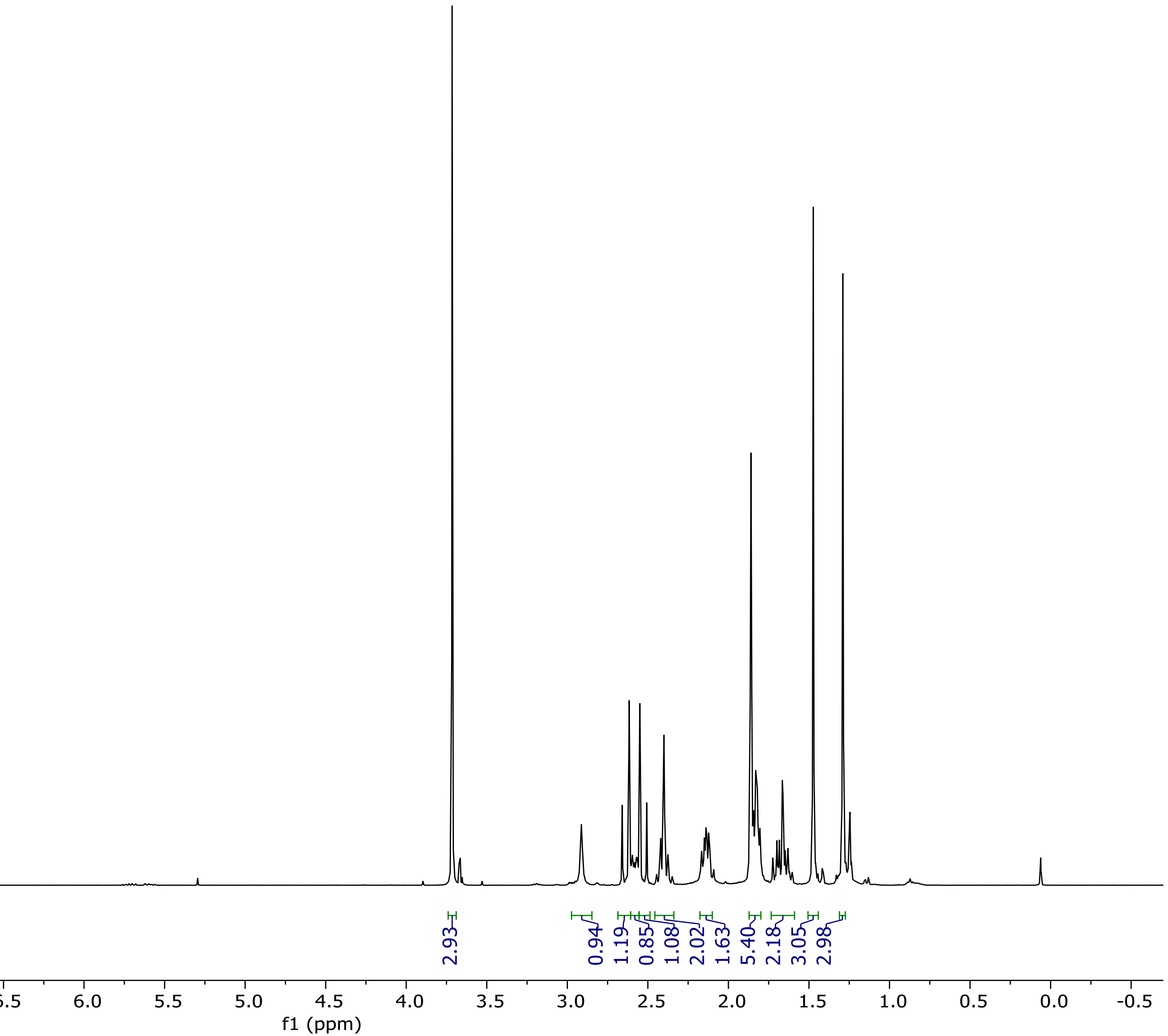

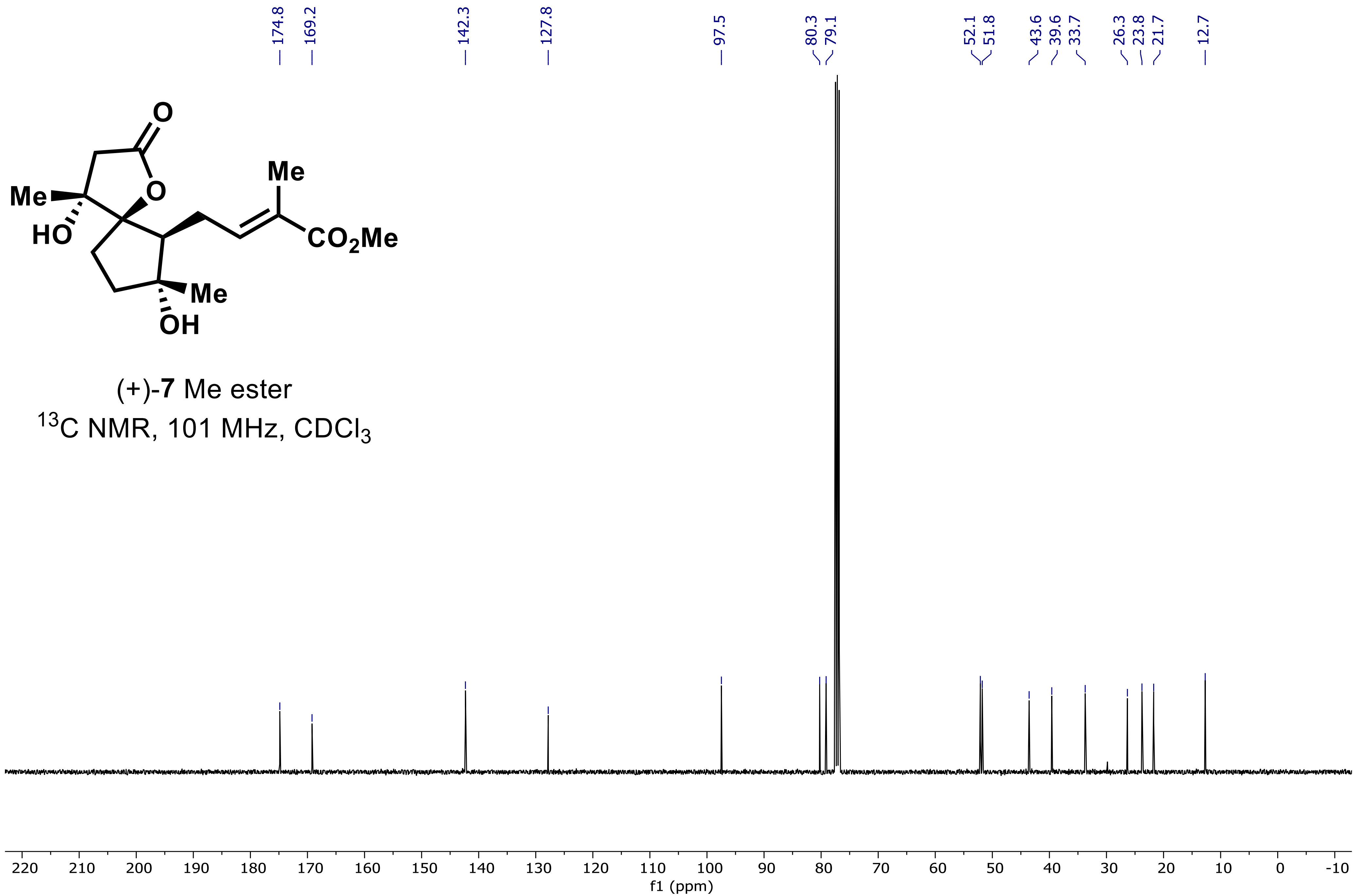

174.8
169.2
142.3
127.8
97.5
80.3
79.1
52.1
51.8
43.6
39.6
33.7
26.3
23.8
21.7
12.7
O
O
Me
Me
HO
CO2Me
Me
OH
(+)-7 Me ester
13C NMR, 101 MHz, CDCl3
220 210 200 190 180 170 160 150 140 130 120 110 100 90 80 70 60 50 40 30 20 10 0 -10
f1 (ppm)

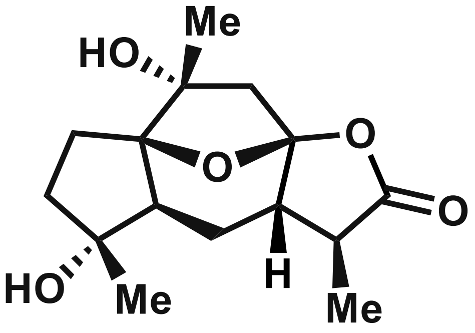

(–)-**6**

^{1}H NMR, 400 MHz, $CDCl_3$

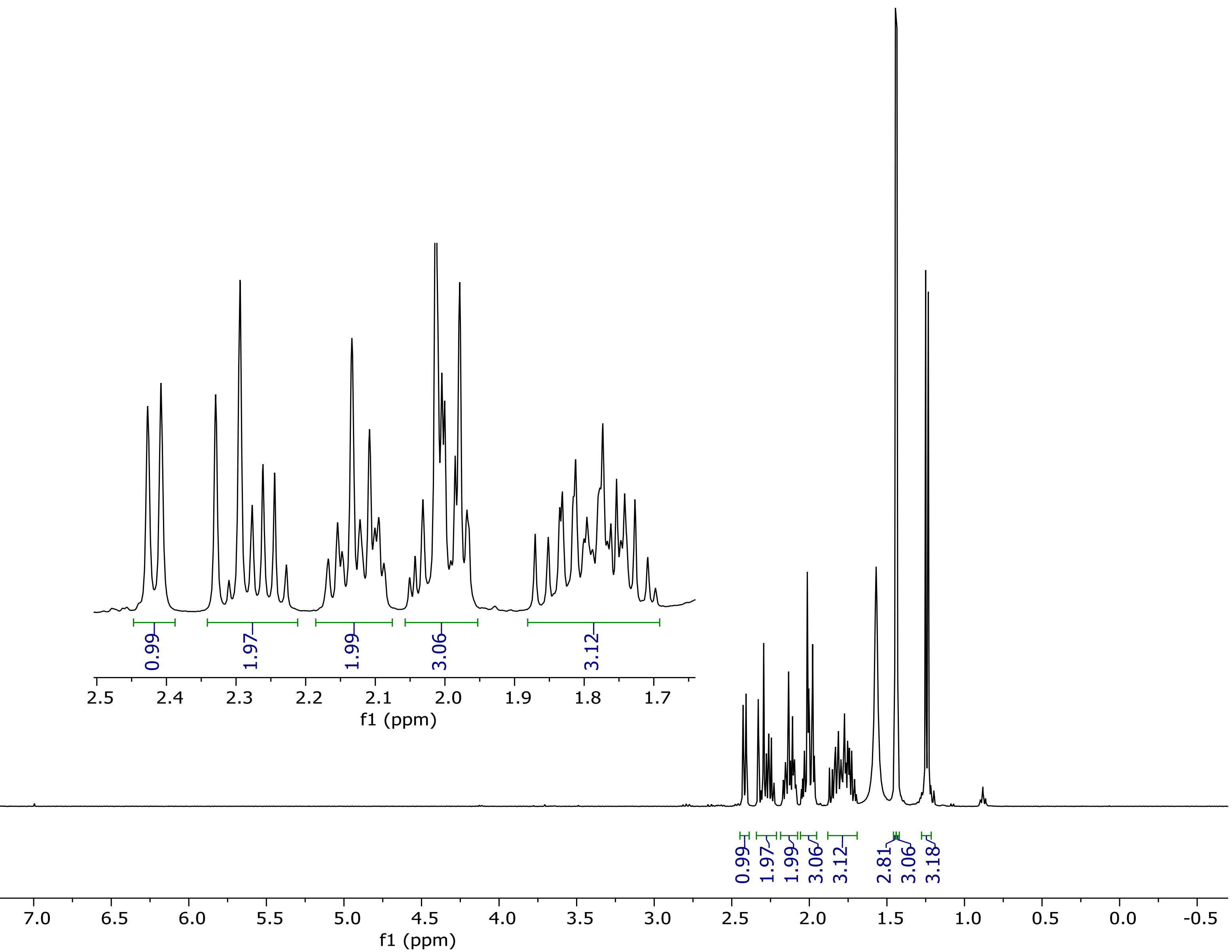

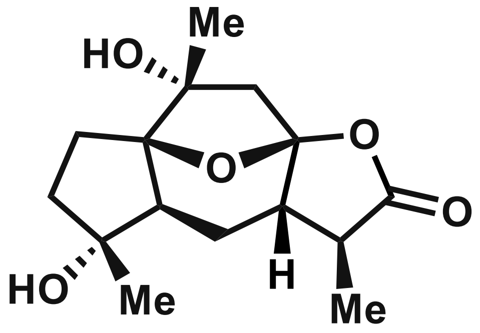

(–)-**6**

^{13}C NMR, 101 MHz, $CDCl_3$

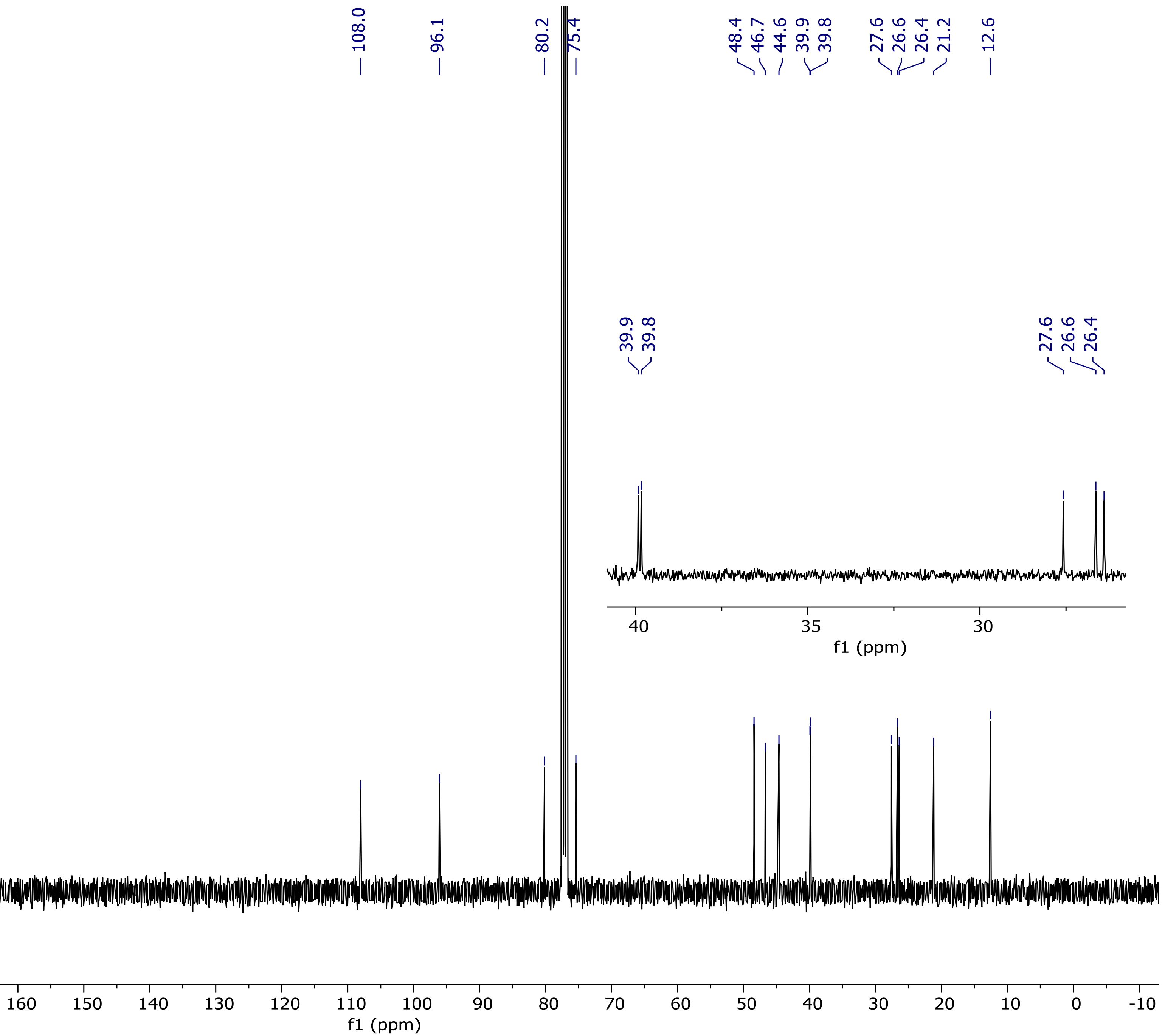

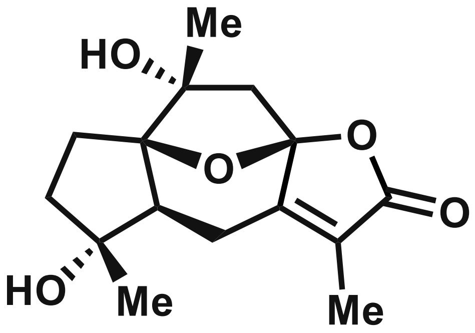

(–)-phaeocaulisin A (**1**)
^{1}H NMR, 500 MHz, CD_3OD

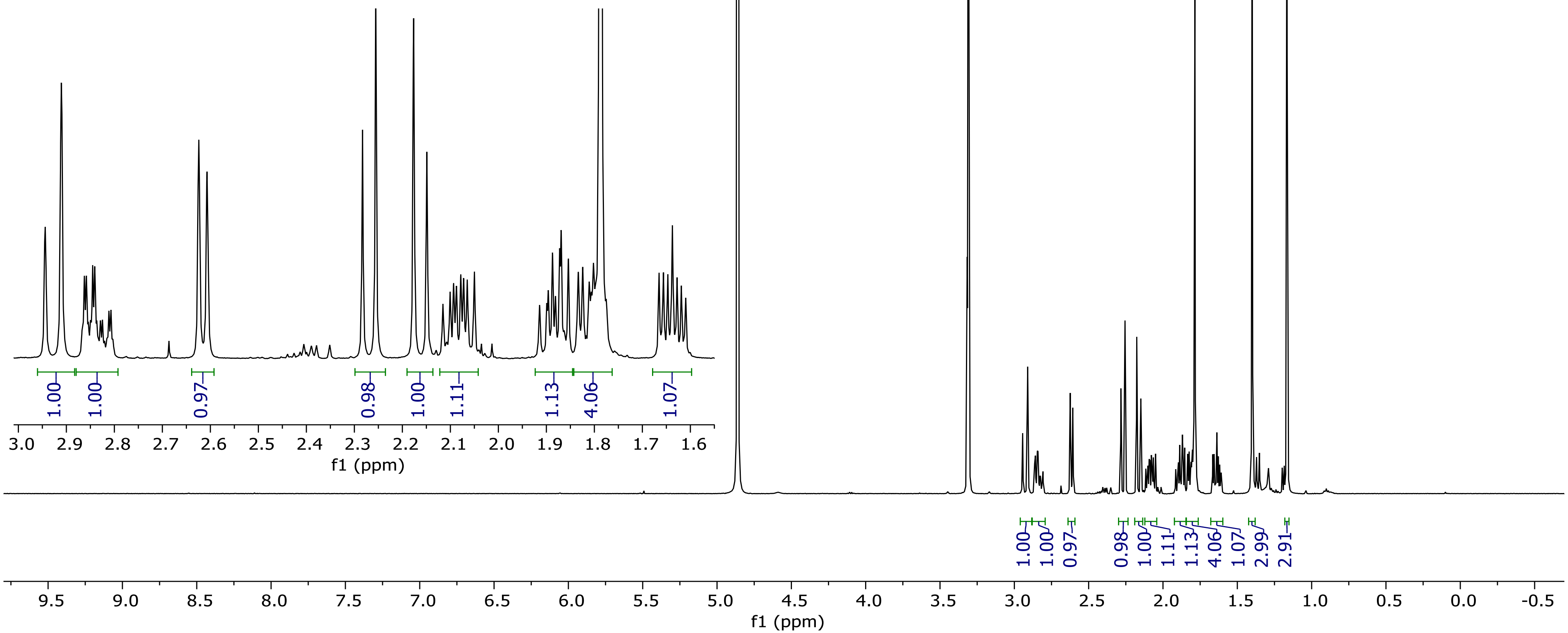

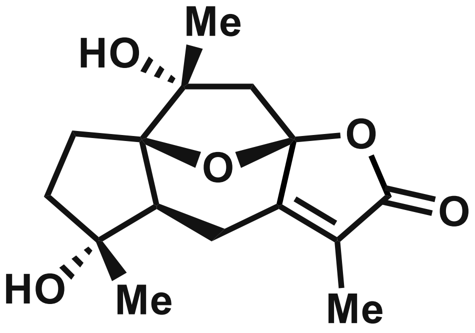

(–)-phaeocaulisin A (**1**)
^{13}C NMR, 151 MHz, CD_3OD

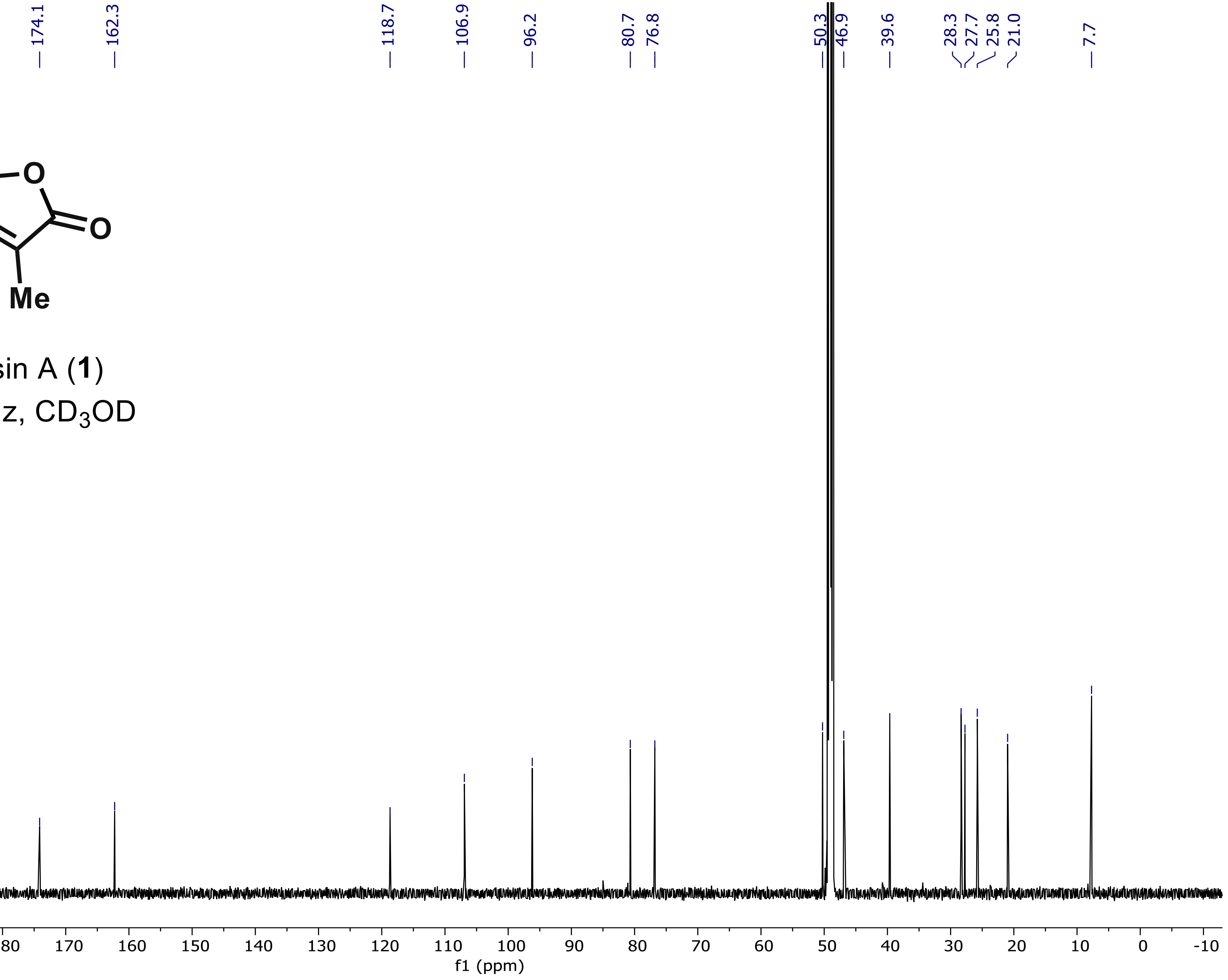

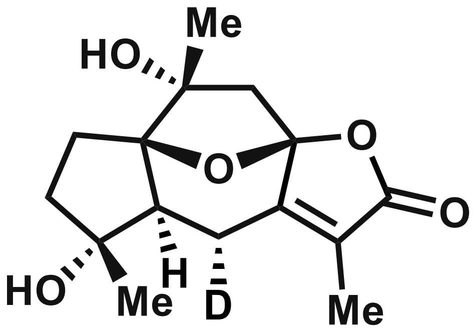

C6-D-phaeocaulisin A

^{1}H NMR, 500 MHz, CD_3OD

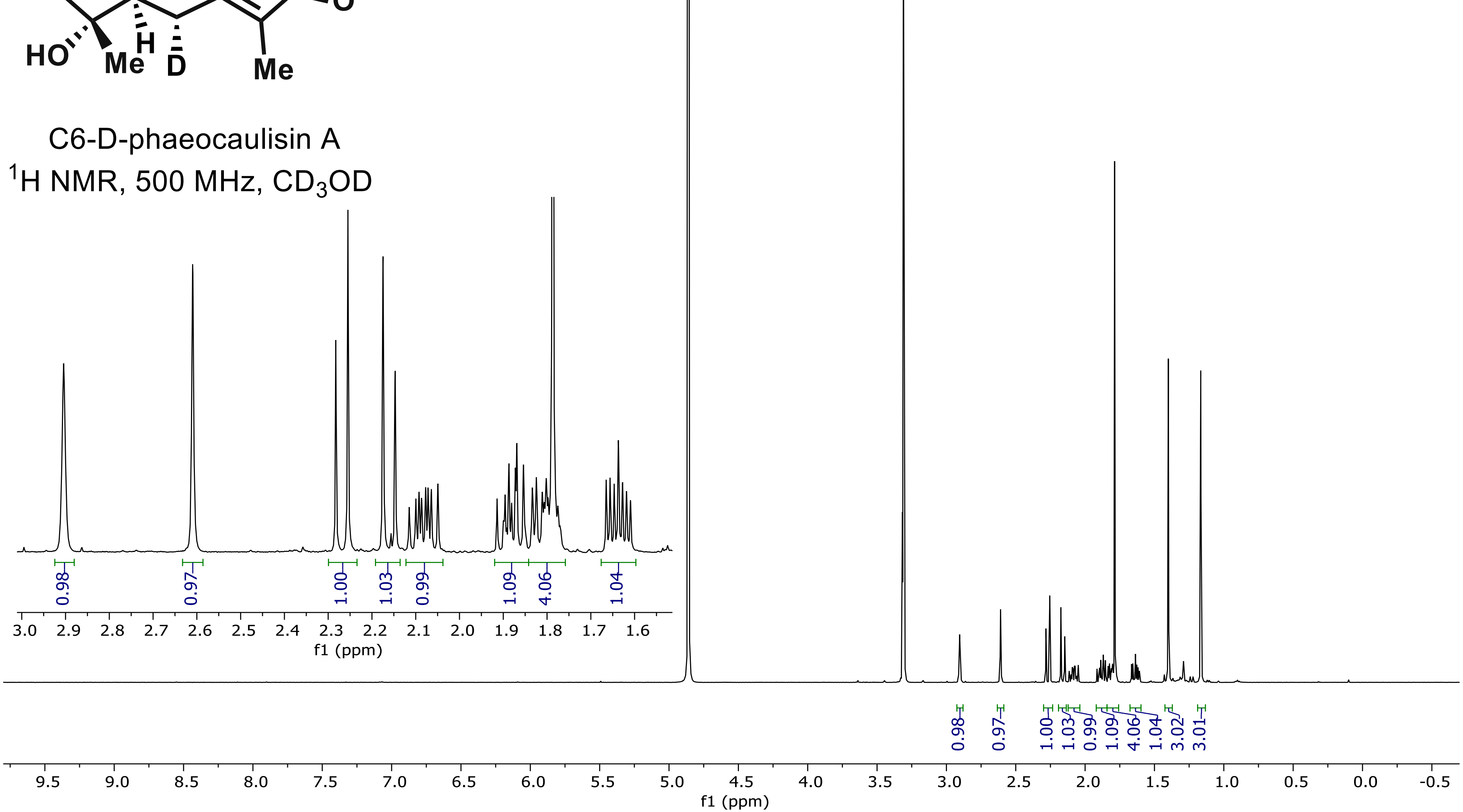

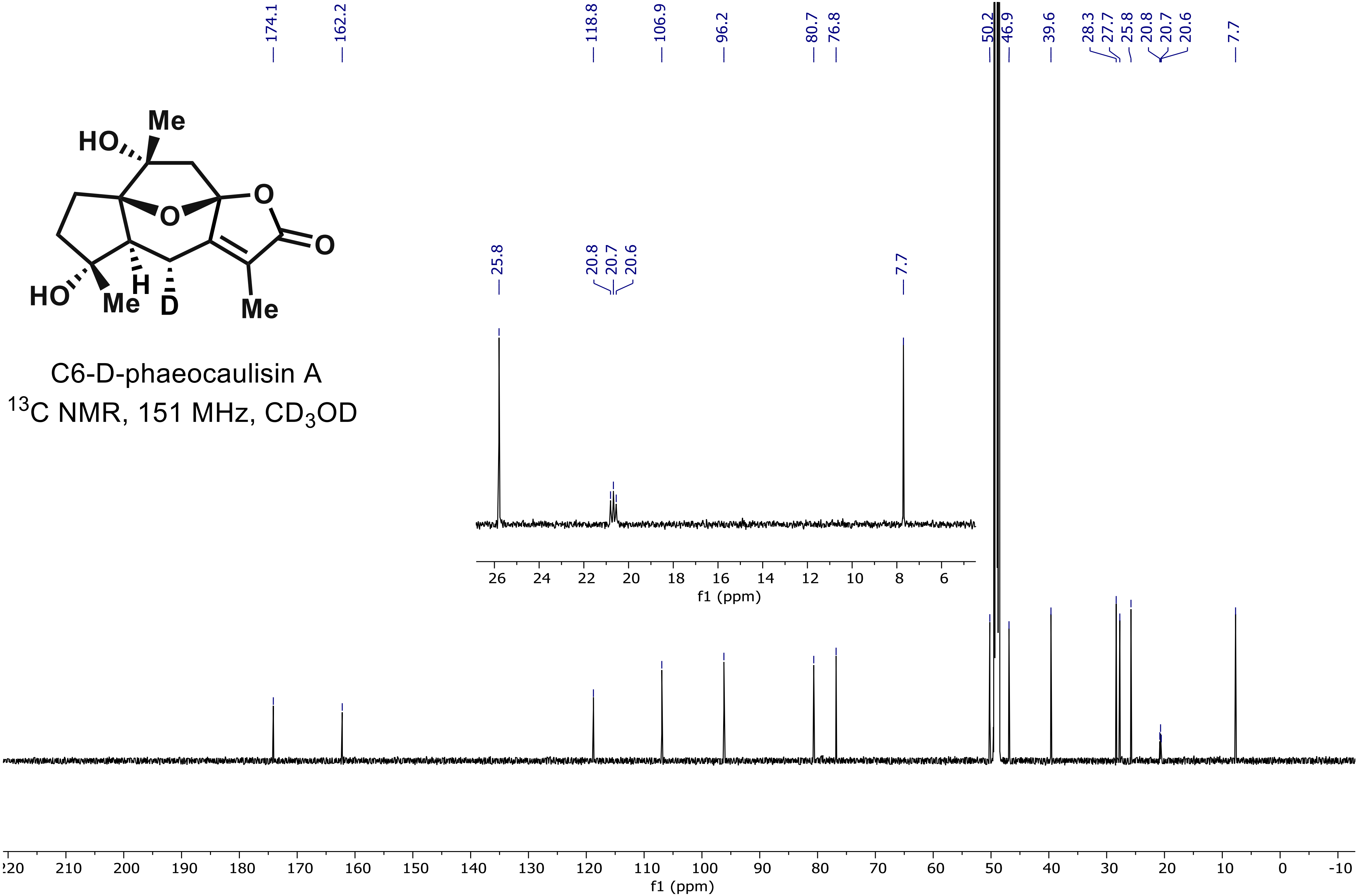

C6-D-phaeocaulisin A

^{13}C NMR, 151 MHz, CD_3OD

10. References

1. Szostak, M., Spain, M. & Procter, D. J. Preparation of samarium(II) iodide: Quantitative evaluation of the effect of water, oxygen, and peroxide content, preparative methods, and the activation of samarium metal. *J. Org. Chem.* **77**, 3049–3059 (2012).
2. Pedersen, D. & Rosenbohm, C. Dry Column Vacuum Chromatography. *Synthesis (Stuttg).* **2001**, (2004).
3. Sejer, D. Dry Column Vacuum Chromatography (DCVC) - The Movie! http://curlyarrow.blogspot.com/2017/02/dry-column-vacuum-chromatography-dcvc.html (2017).
4. Liu, Y. *et al.* Guaiane-type sesquiterpenes from Curcuma phaeocaulis and their inhibitory effects on nitric oxide production. *J. Nat. Prod.* **76**, 1150–1156 (2013).
5. Penner, M., Rauniyar, V., Kaspar, L. T. & Hall, D. G. Catalytic Asymmetric Synthesis of Palmerolide A via Organoboron Methodology. *J. Am. Chem. Soc.* **131**, 14216–14217 (2009).
6. Amans, D., Bellosta, V. & Cossy, J. An efficient and stereoselective synthesis of the monomeric counterpart of marinomycin A. *Org. Lett.* **9**, 1453–1456 (2007).
7. Hari, D. P. & Waser, J. Copper-Catalyzed Oxy-Alkynylation of Diazo Compounds with Hypervalent Iodine Reagents. *J. Am. Chem. Soc.* **138**, 2190–2193 (2016).
8. Foote, K. M., Hayes, C. J., John, M. P. & Pattenden, G. Synthetic studies towards the phomactins. Concise syntheses of the tricyclic furanochroman and the oxygenated bicyclo[9.3.1]pentadecane ring systems in phomactin A. *Org. Biomol. Chem.* **1**, 3917–3948 (2003).
9. Kolb, H. C., VanNieuwenhze, M. S. & Sharpless, K. B. Catalytic Asymmetric Dihydroxylation. *Chem. Rev.* **94**, 2483–2547 (1994).
10. Diaba, F., Ricou, E. & Bonjoch, J. Synthesis of enantiopure 1-azaspiro[4.5]dec-6-en-8-ones from L-proline derivatives. *Tetrahedron: Asymmetry* **17**, 1437–1443 (2006).
11. Lu, P. *et al.* Enantioselective Synthesis of (−)-Maoecrystal V by Enantiodetermining C − H Functionalization. *J. Am. Chem. Soc.* **136**, 17738–17749 (2014).
12. Shi, B., Wu, H., Yu, B. & Wu, J. 23-Oxa-Analogues of OSW-1: Efficient Synthesis and Extremely Potent Antitumor Activity. *Angew. Chemie Int. Ed.* **43**, 4324–4327 (2004).
13. Stivala, C. E., Gu, Z., Smith, L. L. & Zakarian, A. Studies toward the Synthesis of Spirolide C : Exploration into the Formation of the 23-Membered All-Carbon Macrocyclic Framework. *Org. Lett.* **14**, 804–807 (2012).
14. Fukaya, K. *et al.* Practical synthesis of the C-ring precursor of paclitaxel from 3-methoxytoluene. *J. Antibiot. (Tokyo).* **69**, 273–279 (2016).
15. Ooi, T., Otsuka, H., Miura, T., Ichikawa, H. & Maruoka, K. Practical Oppenauer (OPP) Oxidation of Alcohols with a Modified Aluminum Catalyst. *Org. Lett.* **4**, 40–43 (2002).
16. Frigerio, M. & Santagostino, M. A Mild Oxidizing Reagent for Alcohols and 1,2-Diols: o-Iodoxybenzoic Acid (IBX) in DMSO. *Tetrahedron Lett.* **35**, 8019–8022 (1994).
17. Hashimoto, T. *et al.* Phase-Transfer-Catalyzed Asymmetric Alkylation of α-Benzoyloxy-β-keto Esters: Stereoselective Construction of Congested 2,3-Dihydroxycarboxylic Acid Esters. *Chem. - An Asian J.* **5**, 562–570 (2010).
18. Imamoto, T., Takiyama, N., Nakamura, K., Hatajima, T. & Kamiya, Y. Reactions of carbonyl compounds with Grignard reagents in the presence of cerium chloride. *J. Am. Chem. Soc.* **111**, 4392–4398 (1989).
19. Krasovskiy, A., Kopp, F. & Knochel, P. Soluble Lanthanide Salts ($LnCl_3$•2LiCl) for the Improved Addition of Organomagnesium Reagents to Carbonyl Compounds. *Angew. Chem. Int. Ed.* **45**, 497–500 (2006).
20. Zong, H., Huang, H., Liu, J., Bian, G. & Song, L. Added-Metal-Free Catalytic Nucleophilic Addition of Grignard Reagents to Ketones. *J. Org. Chem.* **77**, 4645–4652 (2012).
21. Hughes, J. M. E. & Gleason, J. L. A Concise Enantioselective Total Synthesis of (−)-Virosaine A. *Angew. Chem. Int. Ed.* **56**, 10830–10834 (2017).
22. Fétizon, M., Li, Y. & Jiang, X. Silver(I) Carbonate on Celite. in *Encyclopedia of Reagents for Organic Synthesis* 1–19 (John Wiley & Sons, Ltd, 2017). doi:10.1002/047084289X.rs014.pub2.
23. Xie, X. & Stahl, S. S. Efficient and Selective Cu/Nitroxyl-Catalyzed Methods for Aerobic Oxidative Lactonization of Diols. *J. Am. Chem. Soc.* **137**, 3767–3770 (2015).
24. Hoover, J. M. & Stahl, S. S. Highly Practical Copper(I)/TEMPO Catalyst System for Chemoselective Aerobic Oxidation of Primary Alcohols. *J. Am. Chem. Soc.* **133**, 16901–16910 (2011).
25. Li, W., Silipo, A., Molinaro, A. & Yu, B. Synthesis of bradyrhizose, a unique inositol-fused monosaccharide relevant to a Nod-factor independent nitrogen fixation. *Chem. Commun.* **51**, 6964–6967 (2015).
26. Williams, D. R., Gladen, P. T. & Pinchman, J. R. Total Synthesis of Neodolastane Diterpenes Trichoaurantianolides C and D. *J. Org. Chem.* **80**, 5474–5493 (2015).
27. Yamamoto, Y., Suzuki, H. & Moro-oka, Y. Ruthenium-catalyzed oxidation of alcohols with sodium bromate. *Tetrahedron Lett.* **26**, 2107–2108 (1985).

28. Reddy, S. R. & Chadha, A. A simple and efficient method for mild and selective oxidation of propargylic alcohols using TEMPO and calcium hypochlorite. *RSC Adv.* **3**, 14929 (2013).
29. Vatèle, J.-M. Yb(OTf) 3 -Catalyzed Oxidation of Alcohols with Iodosylbenzene Mediated by TEMPO. *Synlett* **2006**, 2055–2058 (2006).
30. Shimojo, H., Moriyama, K. & Togo, H. Simple One-Pot Conversion of Alcohols into Nitriles. *Synthesis (Stuttg).* **45**, 2155–2164 (2013).
31. Bolm, C., Magnus, A. S. & Hildebrand, J. P. Catalytic Synthesis of Aldehydes and Ketones under Mild Conditions Using TEMPO/Oxone. *Org. Lett.* **2**, 1173–1175 (2000).
32. Giordano, C., Cavicchioli, S., Levi, S. & Villa, M. Direct conversion of (1S,2S)-2-amino-1-[(4-methylthio)phenyl]-1,3-propanediol into its enantiomer for efficient synthesis of thiamphenicol and florfenicol. *J. Org. Chem.* **56**, 6114–6118 (1991).
33. Semmelhack, M. F., Schmid, C. R., Cortes, D. A. & Chou, C. S. Oxidation of alcohols to aldehydes with oxygen and cupric ion, mediated by nitrosonium ion. *J. Am. Chem. Soc.* **106**, 3374–3376 (1984).
34. Karimi, B. & Golshani, B. Mild and Highly Efficient Method for the Silylation of Alcohols Using Hexamethyldisilazane Catalyzed by Iodine under Nearly Neutral Reaction Conditions. *J. Org. Chem.* **65**, 7228–7230 (2000).
35. Hintermann, L. & Labonne, A. Catalytic Hydration of Alkynes and Its Application in Synthesis. *Synthesis (Stuttg).* **2007**, 1121–1150 (2007).
36. Leyva, A. & Corma, A. Isolable Gold(I) Complexes Having One Low-Coordinating Ligand as Catalysts for the Selective Hydration of Substituted Alkynes at Room Temperature without Acidic Promoters. *J. Org. Chem.* **74**, 2067–2074 (2009).
37. Francais, A., Leyva, A., Etxebarria-Jardi, G. & Ley, S. V. Total Synthesis of the Anti-Apoptotic Agents Iso- and Bongkrekic Acids. *Org. Lett.* **12**, 340–343 (2010).
38. Baker, R. & Castro, J. L. Total synthesis of (+)-macbecin I. *J. Chem. Soc. Perkin Trans. 1* 47 (1990) doi:10.1039/p19900000047.
39. Knowles, J. P., O′Connor, V. E. & Whiting, A. Studies towards the synthesis of the northern polyene of viridenomycin and synthesis of Z-double bond analogues. *Org. Biomol. Chem.* **9**, 1876 (2011).
40. Beletskaya, I. P. & Cheprakov, A. V. Heck reaction as a sharpening stone of palladium catalysis. *Chem. Rev.* **100**, 3009–3066 (2000).
41. Péter, Á. Radical cyclization cascades for the construction of natural product-like architectures. (The University of Manchester, 2019, MSc thesis).
42. Hanamoto, T., Sugimoto, Y., Sugino, A. & Inanaga, J. Preparation and Reaction of Lanthanide(II) Trifluoromethanesulfonates. *Synlett* **1994**, 377–378 (1994).
43. Maisano, T., Tempest, K. E., Sadasivam, D. V. & Flowers, II, R. A. A convenient pathway to Sm(II)-mediated chemistry in acetonitrile. *Org. Biomol. Chem.* **9**, 1714 (2011).
44. Schäfer, G. & Bode, J. W. Synthesis of Sterically Hindered N -Acylated Amino Acids from N -Carboxyanhydrides. *Org. Lett.* **16**, 1526–1529 (2014).
45. Roger, M. *et al.* U(SMes*) n , (n = 3, 4) and Ln(SMes*) 3 (Ln = La, Ce, Pr, Nd): Lanthanide(III)/Actinide(III) Differentiation in Agostic Interactions and an Unprecedented η 3 Ligation Mode of the Arylthiolate Ligand, from X-ray Diffraction and DFT Analysis. *J. Am. Chem. Soc.* **128**, 8790–8802 (2006).
46. Kaul, R., Brouillette, Y., Sajjadi, Z., Hansford, K. A. & Lubell, W. D. Selective tert -Butyl Ester Deprotection in the Presence of Acid Labile Protecting Groups with Use of ZnBr 2. *J. Org. Chem.* **69**, 6131–6133 (2004).
47. Marcantoni, E. *et al.* Selective Deprotection of N-Boc-Protected tert -Butyl Ester Amino Acids by the $CeCl_3 \cdot 7H_2O$−NaI System in Acetonitrile. *J. Org. Chem.* **66**, 4430–4432 (2001).
48. Slade, M. C. & Johnson, J. S. Alternaric acid: formal synthesis and related studies. *Beilstein J. Org. Chem.* **9**, 166–172 (2013).
49. Otera, J. Transesterification. *Chem. Rev.* **93**, 1449–1470 (1993).
50. Trost, B. M., Taft, B. R., Masters, J. T. & Lumb, J.-P. A New Strategy for the Synthesis of Chiral β-Alkynyl Esters via Sequential Palladium and Copper Catalysis. *J. Am. Chem. Soc.* **133**, 8502–8505 (2011).
51. Ando, M. *et al.* Tropolone derivatives as synthetic intermediates. 1. A novel synthetic method of the octahydro-2H-cyclohepta[b]furan-2-one derivatives. *J. Org. Chem.* **52**, 1429–1437 (1987).
52. Crossley, S. W. M., Barabé, F. & Shenvi, R. A. Simple, Chemoselective, Catalytic Olefin Isomerization. *J. Am. Chem. Soc.* **136**, 16788–16791 (2014).
53. Crisp, G. T. & Meyer, A. G. Synthesis of optically active α-methylene γ-butyrolactones and (+)-mintlactone. *Tetrahedron* **51**, 5831–5846 (1995).
54. Park, B. R., Kim, K. H., Lim, J. W. & Kim, J. N. Facile synthesis of γ-alkylidenebutenolides from Morita–Baylis–Hillman adducts. *Tetrahedron Lett.* **53**, 36–40 (2012).
55. Vaz, B., Fontán, N., Castiñeira, M., Álvarez, R. & de Lera, Á. R. Synthesis of labile all-trans-7,8,7′,8′-bis-acetylenic carotenoids by bi-directional Horner–Wadsworth–Emmons condensation. *Org. Biomol. Chem.* **13**, 3024–3031 (2015).

56. Braun, M., Mroß, S. & Schwarz, I. Mild and Stereoconvergent Palladium-Catalyzed Carbonyl Alkenation Reaction of α,β-Unsaturated Aldehydes. *Synthesis (Stuttg).* **1998**, 83–88 (1998).
57. Dolomanov, O. V., Bourhis, L. J., Gildea, R. J., Howard, J. A. K. & Puschmann, H. OLEX2: A complete structure solution, refinement and analysis program. *J. Appl. Crystallogr.* **42**, 339–341 (2009).
58. Sheldrick, G. M. Crystal structure refinement with SHELXL. *Acta Crystallogr. Sect. C Struct. Chem.* **71**, 3–8 (2015).
